# Supplementary material for: Genome-wide identification and analysis of the CNGC gene family in maize
Source: PeerJ. 2018 Oct 17;6:e5816. doi: 10.7717/peerj.5816 (PMC6195792; doi:10.7717/peerj.5816)
Supplement: File S1 — The AKT and CNGC indicated maize AKT/KAT channel genes and CNGC genes. S1–S6 indicated six transmembrane (TM) regions with one P region of AKT/KAT or CNGC proteins. [file peerj-06-5816-s001.docx]

Supplemental File 1

**A ML phylogenetic tree, conserved regions and multiple sequences alignments between maize AKT/KAT channels genes and ZmCNGCs.**

The AKT and CNGC indicated maize AKT/KAT channel genes and CNGC genes. S1-S6 indicated six transmembrane (TM) regions with one P region of AKT/KAT or CNGC proteins.


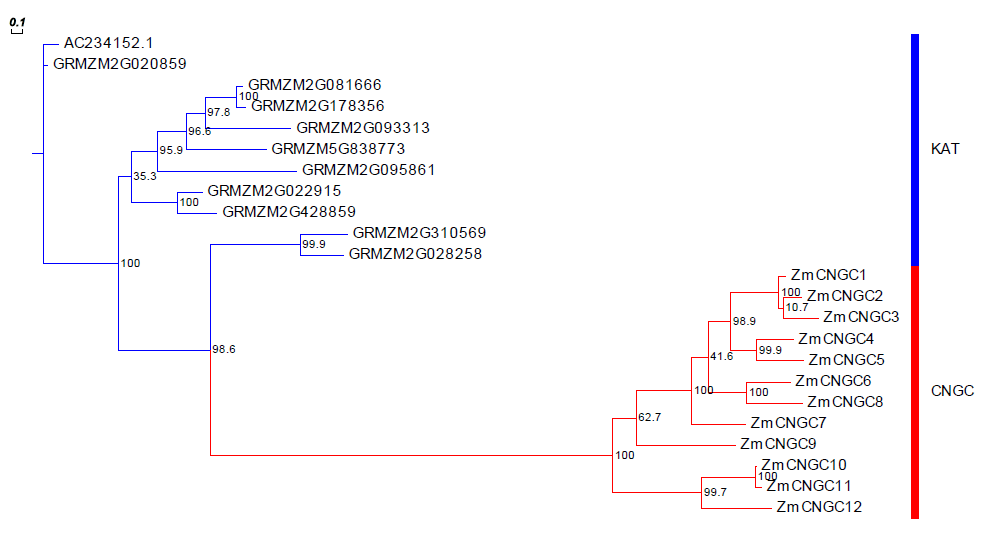


|  | AKT | CNGC |  | AKT | CNGC |
| --- | --- | --- | --- | --- | --- |
| S1 | 11[Y] | 0 | S2 | 12[F] | 6[Y] |
|  | 11[S] | 6[A],1[S] |  | 8[A] | 5[Y] |
|  | 9[A] | 6[L] |  | 6[V],3[I] | 3[A] |
|  | 10[W] | 4[A],4[F] |  | 8[L],4[V] | 4[L] |
|  | 0 | 8[D] |  | 10[F] | 7[F] |
|  | 11[P] | 8[P] |  | 11[A] | 7[A] |
|  |  |  |  | 10[Y] | 6[Y] |
|  |  |  |  | 9[T] | 8[S] |
|  |  |  |  | 7[L] | 8[L] |
| S3 | 9[F] | 5[F],3[L] | S4 | 10[L] | 9[Q] |
|  | 6[I],5[A] | 2[A] |  | 11[R] | 8[Y] |
|  | 12[D] | 8[D] |  | 11[L] | 5[L] |
|  | 9[V] | 4[L] |  | 9[W] | 9[P] |
|  | 9[S] | 0 |  | 11[R] | 7[R] |
|  | 9[T] | 3[A],3[I] |  | 9[L] | 4[L] |
|  | 0 | 7[L] |  | 10[R] | 4[R] |
|  | 11[P] | 8[P] |  | 11[V] | 6[I] |
|  | 8[F] | 6[L] |  | 6[S] | 0 |
|  | 6[Q] | 8[P] |  | 8[L] | 5[L] |
|  | 3[V] | 9[Q] |  | 11[F] | 0 |
|  | 7[I] | 6[V] |  | 7[A] | 0 |
|  | 7[L] | 8[W] |  | 9[R] | 0 |
|  |  |  |  | 9[L] | 4[I] |
|  |  |  |  | 10[E] | 4[Q] |
|  |  |  |  | 10[K] | 4[K] |
| S5 | 8[I] | 9[L] | P | 4[L] | 4[L],3[I] |
|  | 11[V] | 3[L],3[A] |  | 11[Y] | 6[W],4[F] |
|  | 8[T] | 7[Y] |  | 9[W] | 10[W] |
|  | 11[L] | 5[M] |  | 9[S] | 10[G] |
|  | 7[A] | 10[A] |  | 9[I] | 9[L] |
|  | 7[V] | 7[S] |  | 9[T] | 4[Q],4[M] |
|  | 9[H] | 10[H] |  | 11[T] | 5[N],3[T] |
|  | 9[A] | 2[A] |  | 6[L] | 10[L] |
|  | 7[G] | 10[G] |  | 9[T] | 10[S] |
|  | 11[C] | 6[A] |  | 11[T] | 6[T] |
|  | 5[F] | 4[C] |  | 11[G] | 9[G] |
|  | 10[Y] | 10[Y] |  |  |  |
|  | 6[I],5[L] | 8[L] |  |  |  |
| S6 | 11[E] | 10[E] | PBC | 7[G] | 12[G] |
|  | 11[M] | 4[V] |  | 13[G] | 12[G] |
|  | 4[L],4[I] | 5[L] |  | 12[E] | 8[E] |
|  | 11[F] | 9[F] | Hinge | 4[E] | 10[E] |
|  | 7[I] | 8[I] |  | 2[A],8[I] | 11[A] |
|  | 9[N] | 9[G] |  | 5[M] | 12[F] |
|  | 9[L] | 10[L] |  | 13[D] | 9[D] |
|  | 8[G] | 4[I] |  | 9[I] | 10[V],2[I] |
|  | 11[L] | 11[L] |  | 4[L],3[M] | 8[A],4[T] |
|  | 8[T] | 6[F] |  |  |  |
|  | 11[A] | 6[A] |  |  |  |
|  | 11[Y] | 6[L] |  |  |  |
|  | 11[L] | 10[L] |  |  |  |
|  | 9[I] | 10[I] |  |  |  |
|  | 12[G] | 10[G] |  |  |  |
|  | 11[N] | 9[N] |  |  |  |
|  | 11[M] | 4[M],4[I] |  |  |  |
|  | 11[T] | 11[Q] |  |  |  |
|  | 10[N] | 6[T] |  |  |  |
|  | 12[L] | 8[Y],4[F] |  |  |  |
|  | 8[V] | 12[L] |  |  |  |

....|....| ....|....| ....|....| ....|....| ....|....|

10 20 30 40 50

**AC234152.1** -------LVL DQHWAMRVGI A--------- ---------- ---GLVFGET

**GRMZM2G020** WWDTLMVVLV AYSAWVYPFE VAFMNA---- ---------- SPKGGLEVAD

**GRMZM2G081** YWELFLIVLV IYSAWICPFE LAFLR----- ---------- DLPSKLLLAE

**GRMZM2G178** YWELFLTVLV IYSAWICPFE LAFLR----- ---------- DLSSKLLLVE

**GRMZM2G310** LWTKFILGWA VYSSFFTPLE FGFFR----- ---------- GLPENLFFLD

**GRMZM2G093** TWETFLLVLV VYSAWICPFE LAFLR----- ---------- HLSWVLFLVE

**GRMZM2G022** CWETFLIILV IYSAWVSPFE FGFIR----- ---------- KPAGALAAVD

**GRMZM2G028** IWSSAVFLWS IYSTFFTPFE FAFFR----- ---------- GLPDHLLDLE

**GRMZM2G428** AWEHSLVALV AYSAWVAPFE FGFVP----- ---------- DPRGALAVAD

**GRMZM5G838** VWETFLILLV VYSAWICPLE FAFLR----- ---------- YLPRAPFVVD

**GRMZM2G095** CWQAFLVAPV LYSAWASPFE LAVER----- ---------- AVTFPLLVAD

**ZmCNGC1**  RWNKIFVISC LFAVFVDPLF LYVPVI---D GGNNCLYLDK KLETTASILR

**ZmCNGC2**  ---------- ---------- ---------- ---------- ----------

**ZmCNGC3**  ---------- ---------- ---------- ---------- ----------

**ZmCNGC4**  RMNWVFFSSC LFAVAVDPLF FFLPIIN--D SN--CIGIDK KLAVTSTIIR

**ZmCNGC5**  RLNRAFFISC IVAIAVDPMF FYLPMVT--D EGNLCVGIDR WLAISTTVVR

**ZmCNGC6**  TWNRIFLFSC FVALFIDPLY FYVPKIS-YG SPKFCIGTDT RFAVGVTFFR

**ZmCNGC7**  LWNKIFLSAC LLSLFVDPLF LYLTGTQ--- -RNTCVEFKD SLALTLSMVR

**ZmCNGC8**  RWNRVYLVAC LFALFIDPFF YYLPLIRQNG NGSSCVAKDQ GLSIRITVLR

**ZmCNGC9**  ---------- ---------- ---------- ---------- ----------

**ZmCNGC10**  RWNRWILLGR AAALALDPLF FYALSIG--R AGRPCLYLDA GLAAAVTALR

**ZmCNGC11**  RWNRWILLGR AAALAVDPLF FYALSIG--R AGQPCLYMDA GLASAVTALR

**ZmCNGC12**  ---------- ---------- ---------- ---------- ----------

....|....| ....|....| ....|....| ....|....| ....|....|

60 70 80 90 100

**AC234152.1** LLGWYQAGDD --GFSTSFLP ---------- ---LEGAAQK TSSLYLSTF-

**GRMZM2G020** MVVDLFFAVD IVLTFFVAYI DP-------- RTQLLVRDRK KITLRYLSTF

**GRMZM2G081** NIVNGFFAVD IVLTFFVAYV DS-------- KTHLLVDDQK RIAVRYLSTW

**GRMZM2G178** NIVNSVFAID IVLTFFVAYV DS-------- KTHLLVDDRK RIAVRYLSTW

**GRMZM2G310** TVGQAAFLID IVVKFFVAYR DP-------- DTYRIVYSPT AIALRYCKSS

**GRMZM2G093** NIVNSFFAID IILTFFLAYL DK-------- KSYLLVDNPK RIAASMYA--

**GRMZM2G022** NVVNAFFAVD IILTFFVAYL DR-------- MTYLLEDDPK RIAWRYTTSW

**GRMZM2G028** CV-QLIFLAD VAVHFLLAYR DA-------- RTYRMVYDKR RIALRYIKGS

**GRMZM2G428** NAVNAAFAVD IALTFFVAYA DG-------- GTYLLQDDPR RIAWRYARSW

**GRMZM5G838** DVVNGFFAVD IVLTFFVPYV DS-------- KSCLVVDDHR KIAARYLSTW

**GRMZM2G095** LVADAFFFVD IAVSIVVAWL PLRG---SSC ATNLFYDDRK KAYVRNLRPW

**ZmCNGC1**  FFTDIFYLLH ILFQFRTGFI APSS--RVFG RGALVKDTFA IAKRYLSTLF

**ZmCNGC2**  ---------- ---------- ---------- ---------- ----------

**ZmCNGC3**  ---------- ---------- ---------- ---------- ----------

**ZmCNGC4**  TVIDFVYLIR VCLQFRTAYV APSS--RVFG TGELVIDPML IAKRYIKSYF

**ZmCNGC5**  CVVDLFFLGR IALQFRTAYI KPSS--RVFG RGELVIDTAL IARRYMRRFF

**ZmCNGC6**  SIADLLYVLH IIIKFRTAYI NPSSTLRVFG RGDLVTNPKE IAWKYIRSDL

**ZmCNGC7**  SLLDLFYAAH ILFRFRTAFI APSS--RVFG RGELVIQPYE IARRYLGRTF

**ZmCNGC8**  SLADLFYMLN IAIKFHTAYV DPKS--RVLG KGELVVDIKK IQRRYIRTDF

**ZmCNGC9**  ---------- ---------- ---------- ---------- ----------

**ZmCNGC10**  TCADVAHLAH VLLQFRLAYV SRESLVVGCG KLVWDARAIA AHYARSVKGL

**ZmCNGC11**  TCADVAHLAH VLLQLRLAYV SRESLVVGCG KLVWDARAVA AHYARSVKGL

**ZmCNGC12**  ---------- ---------- ---------- -------MHA ACVTISVR--

....|....| ....|....| ....|....| ....|....| ....|....|

110 120 130 140 150

**AC234152.1** FIMDVASTIP FQGLAYLITG ---------- EVRENAVYSM LGVLRLWRLR

**GRMZM2G020** -FIMDVASTI PFQSLAYLIT G--------- -EVRENAAYS MLGVLRLWRL

**GRMZM2G081** -FIFDVCSTA PFQPISLLFT R--------- --KGNGLAFK ILNMLRLWRL

**GRMZM2G178** -FIFDVCSTA PFQSISLLFT H--------- --KGNDLAFK ILNMLRLWRL

**GRMZM2G310** -FIFDLLGCF PWDAIYRACG S--------- -----KEEVR YLLWIRLTRV

**GRMZM2G093** ----DISPLG SFLTFAQQFH I--------- --K------R LVYYLKSMQM

**GRMZM2G022** -FILDVASTI PSEFARKILP P--------- -NLRS---YG FFNMLRLWRL

**GRMZM2G028** -FALDILGCF PWDSIYKATG R--------- -----AEAVR CLVWLRLYRA

**GRMZM2G428** -LALDVASTV PTEVYRRVLP R--------- -QARS---YN FFGMLRLWRL

**GRMZM5G838** -FAFDVCSTF PFQSISLLFD E--------- --HEHSLGLK FLNVLRLWRL

**GRMZM2G095** TLAMDVASTI PFQVIYLLGT SW-------G AAAAWLSPCR YLSLLRLWRL

**ZmCNGC1**  LVDFLAVLPL PQVFVLVVLP KL-QGPEIMK AKIVLLVIII CQYVPRLLRI

**ZmCNGC2**  ---------- ---------- ---------- ---------- ----------

**ZmCNGC3**  ---------- ---------- ---------- ---------- ----------

**ZmCNGC4**  AMDFVALLPL PQIVVWRYLH IP-DGPDVLT TKTALVWVVL IQYIPRLLRI

**ZmCNGC5**  SADLMSVLPL PQVVIWKFLH RS-KGTAVLD TKNSLLFIVF IQYVPRVVRI

**ZmCNGC6**  AVDVAAALPL PQIIVWFVIP AI-KYSSAEH NNNILVLIVL AQYLPRLYLI

**ZmCNGC7**  WFDLVTALPL PQFVIWIVIP RLNEYSRTAN TKNILRFSII FQYLPRLFQI

**ZmCNGC8**  FVDILAAVPL PQVTVWLIMP AI-KSSDYNI RNTTFALVIV IQYVIRMYLI

**ZmCNGC9**  ---------- ----ILLVVP KV-GLSAANY AKNLLRVTVL LQYVPRIIRF

**ZmCNGC10**  CFDLFVILPI PQVIFWLVIP KLIREERVRL IMTILLLMFI FQFLPKVYHS

**ZmCNGC11**  CFDLFVILPI PQVIFWLVIP KLIREEQVKL IMTILLLMFI FQFLPKVYHS

**ZmCNGC12**  ---------- SQVVVWVATP AMIRAGSTTD VMIVLLTAFL LEYLPKIYHA

....|....| ....|....| ....|....| ....|....| ....|....|

160 170 180 190 200

**AC234152.1** RVKQFFTRLE KDIRFSYFWI RSARLVAVTL FLVHCAGCLY YLIADRYPDR

**GRMZM2G020** RRVKQLFTRL EKDIRFSYFW IRSARLIAVT LFLVHCAGCL YYLIADRYPD

**GRMZM2G081** HRVSSLFARL EKDIRFNYFW TRCSKLISVT LFAVHCAGCF NYMIADRYPD

**GRMZM2G178** HRVSSLFARL EKDIRFNYFW TRCSKLISVT LFAVHCAGCF NYMIADRYPD

**GRMZM2G310** TKVTEFFWRL EKDIRINYLF TRIVKLIVVE LYCTHTAACI FYYLATTLPE

**GRMZM2G093** ASLTEFLT-- ---------- --CYGYGAFV ASVIYLPGCF NYLIADRYPN

**GRMZM2G022** RRVSSLFARL EKDRHFNYFW VRCAKLICVT LFAVHCSACF YYLLADRYPD

**GRMZM2G028** RNILAFFRRM EKDIRISYLF TRVVKLVTVE LHFTHTAACA FYYLATTLPP

**GRMZM2G428** HRVGTLFAQL EKDRKFRYFW VRCTRLICVT LFAVHCAGCF YYLLADRYPD

**GRMZM5G838** RRVSSLFARL EKDIRFNYAV IRCTKLISVT LFAIHCAGCI NYLIADRYPD

**GRMZM2G095** RRVSELFAKL EKDVRLNYYW TRLVKLVGVT LLAVHAAACV LLWMASHYSG

**ZmCNGC1**  IPLYLQITRS AGILTETAWA GAAFNLIIYM LASHGFGALW YILSIQREDT

**ZmCNGC2**  ---------- ---------- ---------- ---------- ----------

**ZmCNGC3**  ---------- ---------- ---------- ---------- ----------

**ZmCNGC4**  FPVITDLKRT AGVFIETAWA GAAYYLLWFM LAGHNVGTLW YFLTIEREDD

**ZmCNGC5**  YPISSELKRT SGVFAETAYA GAAYYLLWYM LASHIVGAFW YLLSIERVSD

**ZmCNGC6**  FPLTYEIVKA TGVVAKTAWE GAAYNMVLYL IASHVLGALW YLLSVDRQTF

**ZmCNGC7**  FPLSGRIVMA TGVMTETAWA GAAYNLILYM LASHVLGALW YLFSVQRQEA

**ZmCNGC8**  IPLSNQIIKA VGVVAKSAWG GAAYNLLLYM LASHITGAIY YLLSIERQIT

**ZmCNGC9**  VPLLDGQS-T NGFIFESAWA NFVINLLMFI LAGHVVGSCW YLFGLQRVNQ

**ZmCNGC10**  IHIMRKMQKV TGYIFGSIWW GFGLNLFAYF IASHIAGGCW YVLAIQRIAS

**ZmCNGC11**  IHIMRKMQKV TGYIFGSIWW GFGLNLFAYF IASHIAGGCW YVLAIQRIAS

**ZmCNGC12**  VRVLRRMQGV SGYLFGTIWW GIALNLMAYF VAAHAVGACW YLLGAQRATK

....|....| ....|....| ....|....| ....|....| ....|....|

210 220 230 240 250

**AC234152.1** QKTWIGA--- ---------- ---------- ---------- ----------

**GRMZM2G020** REKTWIGA-- ---------- ---------- ---------- ----------

**GRMZM2G081** PEKTWIGA-- ---------- ---------- ---------- ----------

**GRMZM2G178** PEKTWIGA-- ---------- ---------- ---------- ----------

**GRMZM2G310** SMEGYTWIGS L--------- ---------- ---------- ----------

**GRMZM2G093** PARTWIGA-- ---------- ---------- ---------- ----------

**GRMZM2G022** PTDTWIGN-- ---------- ---------- ---------- ----------

**GRMZM2G028** AREGGTWIGS L--------- ---------- ---------- ----------

**GRMZM2G428** PGHTWLSS-- ---------- ---------- ---------- ----------

**GRMZM5G838** PRRTWIGA-- ---------- ---------- ---------- ----------

**GRMZM2G095** PKERTW---- ---------- ---------- ---------- ----------

**ZmCNGC1**  CWRQACINQT ---------- GCDPTSLYCG YHS------- -LANNSFLQN

**ZmCNGC2**  ---------- ---------- ---------- ---------- ----------

**ZmCNGC3**  ---------- ---------- ---------- ---------- ----------

**ZmCNGC4**  CWHLYCDDPN F-------GL GCNSSYLYCN NHHHGSYDSW LTNNSAQVFN

**ZmCNGC5**  CWRNACDEFP ---------- GCNQIYMYCG NDRQLGFLEW RTITRQVINE

**ZmCNGC6**  CWKTNCL--- N-------ET GCDLKYLDCD TTPN---ATW ANTTAVFSN-

**ZmCNGC7**  CWREACLLVS ---------P TSQTMFFDCK ALSSN----- RTIWYELSNI

**ZmCNGC8**  CWDQQCVAEY N-------DT HCNFSFISCE NNGSNDYSVW ANKTKVFAN-

**ZmCNGC9**  CLRDACSIST ---------I PYCDSFIDCG RGIGSG--LY RQQWFNDSGA

**ZmCNGC10**  CLQEECKKNN S-CDLISLAC SKEICFHPPW SSN-VNGFAC DTNMTSFSQR

**ZmCNGC11**  CLQEECKRNN S-CDLISLAC SKEICFHPPW SSN-VNGFAC DTNMTSFSQQ

**ZmCNGC12**  CLREQCAQAG SGCAPWALAC AEPLYYGATA SSVGAARLAW AGNATARG--

....|....| ....|....| ....|....| ....|....| ....|....|

260 270 280 290 300

**AC234152.1** ---------- ---------- VIPNFRQASL RIRYISSIYW SITTMTTVGY

**GRMZM2G020** ---------- ---------- -VNPNFRQAS LRIRYISSVY WSITTMTTVG

**GRMZM2G081** ---------- ---------- -VMPTFRSES LWARYVTALY WSITTLTTTG

**GRMZM2G178** ---------- ---------- -VMPTFRSES LWTRYVTALY WSITTLTTTG

**GRMZM2G310** ---------- ------QLGD YKFTHFREID LAKRYITSLY FAIVTMATVG

**GRMZM2G093** ---------- ---------- -ARPDYRSES LWVRYVTSIY WSITTLTTTG

**GRMZM2G022** ---------- ---------- -SMPDFHQRG LWIRYVVSVY WSITTLTTVG

**GRMZM2G028** ---------- ------ALGD ARYAGFREVG LLTRYVTSLY LAVVTMATVG

**GRMZM2G428** ---------- ---------- -SMPDFKQQS VWKRYAASMY WSITTLTTVG

**GRMZM5G838** ---------- ---------- -AMPDFREAG LWIRYVTSMY WSITTMTTTG

**GRMZM2G095** ---------- ---------- -LSRGFETSS VWAGYTRAVY WSLTTLTTVG

**ZmCNGC1**  ACPTNSTANP DPIFGIFLPA LQ-NVSQSTS FFEKLFYCFW WGLQNLSSLG

**ZmCNGC2**  ---------- ---------- ---------- ---------- ----------

**ZmCNGC3**  ---------- ---------- ---------- ---------- ----------

**ZmCNGC4**  MCNGGQDN-- PFNFGIYEQA LVSKILSPGN FISKLCYCFW WGLQNLSTLG

**ZmCNGC5**  TCEPKRDGSI PFNYGIYSPA VVSDVLKSKD TTSKLLFCLW WGLANLSTLG

**ZmCNGC6**  --CNASDTNI SFDFGMFEPA LSNQAPAQS- FAMKYFYSLW WGLQNLSCYG

**ZmCNGC7**  TTSRCTPGNG FYPFGIYEEA LYAKLTSSS- FTQKYFYCFW WGLKNLSSLG

**ZmCNGC8**  --CDATNSSI SFNYGMFSSA LSKGAVSSP- FLEKYFFCLW WGLLQLSSSG

**ZmCNGC9**  EACFNTGNDA TFQYGIYEQA VLLTTEDSA- -VKRYIYSLF WGFQQISTLA

**ZmCNGC10**  NVSTCLSGKG SFAYGIYLGA LPVISSNSL- -AVKILYPIF WGLMTLSTFG

**ZmCNGC11**  NVSTCLSGKG SFAYGIYLGA LPVISSNSL- -AVKILYPIF WGLMTLSTFG

**ZmCNGC12**  ---TCLDSAD NYQYGAYQWT VMLVANPSR- -VERVLLPIF WGLMTLSTFG

....|....| ....|....| ....|....| ....|....| ....|....|

310 320 330 340 350

**AC234152.1** GDLHAQNNVE MIFNIFYMLF NLGLTAYLIG NMTNLVVEGT RRTMEFRNSI

**GRMZM2G020** YGDLHAQNTV EMIFNIFYML FNLGLTAYLI GNMTNLVVEG TRRTMEFRNS

**GRMZM2G081** YGDLHAENPR EMLFDICYML FNLGLTAYLI GNMTNLVVHG TSRTRSFRDS

**GRMZM2G178** YGDLHAENPR EMLFDICYML FNLGLTAYLI GNMTNLVVHG TSRTRNFRDS

**GRMZM2G310** YGDIHAVNIR EMIFIMIYVS FDMILGAYLI GNMTALIVKG SRTER-FRDK

**GRMZM2G093** YGDLHAENPR EMLFSICYML FNLGLTAYLI GNMTNLVVQG SCRTRNFRDT

**GRMZM2G022** YGDLHAENTR EMIFNIFYML FNLGLTAYLI GNMTNLVVHG TSRTRKYRDT

**GRMZM2G028** YGDIHAVNPR EMAFTAVYIS FSILLSAYLV GNMTALIVRG SRTER-FRDR

**GRMZM2G428** YGDMHAVNTG EMVFTTFYML FNLGLTAYLI GNMTNLVVHG TSRTRKYRDA

**GRMZM5G838** YGDLHAENSR EMLFGIAFML FNLWLTAYLI GNMTNLVVHS TGRTRDFRDM

**GRMZM2G095** YGDLHPANPG EMAFAVFYML FNLGLAAYIV GNMTNLAVSD STALLALRDT

**ZmCNGC1**  QNMKTSTNTL ENLFAVFVST SGLVLFALLI GNVQTYLQSA SVRIEEMRVK

**ZmCNGC2**  ---------- ---------Y VCFLLLTMFH PYLQTYLQSA SLRVEEMRVK

**ZmCNGC3**  ---------- ------VSKF NCFLFLGWIE LLLQTYLQSA SGHIEEMRVR

**ZmCNGC4**  QGLLTSTYPG EVLFSIAICV LGLILFALLI GNMQSYLQSV AIRLEEMRVK

**ZmCNGC5**  QGLKTSIYTG EALFSIALAI FGLILMAMLI GNIQTYLQSL TVRLEEMRVK

**ZmCNGC6**  QTLTVSTYLG ETLYCIFLAV LGLVLFAHLI GNVQTYLQSI TVRVEEWRLK

**ZmCNGC7**  QNLSTSLFIG EITFAIVVGV LGLVLFGLLI GNMQSYLQAT MVRLEEWRTK

**ZmCNGC8**  NPLVTSAFIT ENAFAIAIGA ISLILFAQLI GKMQTYLQSI SKRLEEWRLR

**ZmCNGC9**  GNLVPSYFIW EVLFTMAIIG LGLLLFALLI GNMQNFLQAL GRRRLEMQLR

**ZmCNGC10**  NDLAPTSNGI EVIFSIINVL SGLMLFTLLI GNIQVFLHAV LARKRKMQLR

**ZmCNGC11**  NDLAPTSNGI EVIFSIINVL SGLMLFTLLI GNIQVFLHAV LARKRKMQLR

**ZmCNGC12**  N-LESTTEWL EIVFNIVTIT GGLILVTMLI GNIKVFLNAT TSKKQAMHTR

....|....| ....|....| ....|....| ....|....| ....|....|

360 370 380 390 400

**AC234152.1** RAASSFVGRN HLPPRLKQQI LAYMCLKFRA ES--LNQQQL MDQLPKSICK

**GRMZM2G020** VRTASSFVGR NHLPPRLKQQ ILAYMCLKFR AES--LNQQQ LMDQLPKSIC

**GRMZM2G081** IQSASEFASR NQLPDKIKQQ MLSHFCLQF- K-TEGLSQQA MLNCLPKGIR

**GRMZM2G178** IQSASEFAAR NQLPEKIKQQ MLSHFCLQF- K-TEGLNQQA MLNCLPKGIR

**GRMZM2G310** MKEVIRYMNR NKLGKEIREQ IKGHLRLQYE S---SYTEAS VLQDIPISIR

**GRMZM2G093** IHAASQFASR NQLPEYIRDE MLSHICLRY- K-TESLKQKE TLDSLPKGIR

**GRMZM2G022** IQAATSFALR NQLPSRLQDQ MISHLSLKFR TDSEGLQQQE TLDALPKAIR

**GRMZM2G028** MADLIRYMNR NKLGAGVRSQ VKDHLLLQYE S---SYTRDR IVDDIPVAVR

**GRMZM2G428** IKAATSFAVR HQLPPRLQEQ MVSHLSLKFR TDSEGLQQQE TLDALPKAIR

**GRMZM5G838** VQVASEFAAR NQLPQQIEEQ MLNHICLRF- R-TEGLKQQE TLDMLPKAMR

**GRMZM2G095** LRGVSMFGAV NRLPEALTEQ MAETVRLNF- DMTEQLLQQQ LLSEMPRAVR

**ZmCNGC1**  RRDTEQWMAH RLLPENLKDR IMRHEQYRWQ ETR-GVDEEG LLKNLPKDLR

**ZmCNGC2**  SRDTDQWMSY RHLPENLKER IRRYEQYRWQ ETS-GVDEEQ LLMNLPKDLR

**ZmCNGC3**  RRDMEQWMSY RLLPEHIKER ILRHHQYRWQ ETQ-GVDEEG LLVNLPKDLR

**ZmCNGC4**  KRDAEQWMHH RSLPLDIRHR VRKYERYRWL ETR-GVDEET LVQTLPKDLR

**ZmCNGC5**  QRDSEQWMHH RLLPPELRER VRRYDQYKWL NTH-GVDEEA LVQNLPKDLR

**ZmCNGC6**  QRDTEEWMRH RQLPCELRER VRRFIQYKWL ATR-GVNEES ILHALPADLR

**ZmCNGC7**  RTDMERWMHH RQIPQPLKQC VRRYHQYQWV ATR-GVDEEA LLQDLPMDIR

**ZmCNGC8**  QRDMDEWMRH HQLPSHLQER VRRFVQVKWL ATR-GVEEES ILQALPADIR

**ZmCNGC9**  RRDVEKWMSH RRLPEDLRRR VRRAERFTWA ATQ-GVNEEE LLSNLPEDIQ

**ZmCNGC10**  FRDMEWWMRR RQLPSRLRQR VRKYERERWA AVT-GDEEME MIKDLPEGLR

**ZmCNGC11**  FRDMEWWMRR RQLPSRLRQR VRKYERERWA AVT-GDEEME MIKDLPEGLR

**ZmCNGC12**  LRSVELWMKR KDLPRSYRHR VRQYERQRWA ATR-GVDECR IVRDLPEGLR

....|....| ....|....| ....|....| ....|....| ....|....|

410 420 430 440 450

**AC234152.1** SICEHLFVPV VKDVYLFRGV SREMLLSLVT KMKPEYIPPK EDVIVQNEAP

**GRMZM2G020** KSICEHLFVP VVKDVYLFKG VSREMLLSLA TKMKPEYIPP KEDVIVQNEA

**GRMZM2G081** SGIAYNLFFT IIRKAYLFHG VSNSFIAELV MEVQAEYFPP MEDIMLQNEA

**GRMZM2G178** SSIAYNLFFT IIRQAYLFHG VSNDFIAELV MEVQAEYFPP KEDIMLQNEG

**GRMZM2G310** AKISQTLYKP YVESIPLFKG CSAEFIQQIV IRLQEEFFLP GEVILEQGSA

**GRMZM2G093** SGIAYHLFFP VIEKVYLFRG VSYTCMLQLV TAMEAEYFPP RELVILQNEA

**GRMZM2G022** SGISQYLFFN LVQKVYLFEG VSNDLIFQLV SEMKAEYFPP REDVILQNEA

**GRMZM2G028** SKMSQTLYLD MVSRVHLFKG CSEDFLSQIV VKLHEEFFLP GEVILEQGTV

**GRMZM2G342** ---AHQAMRR DVAGAALLG- ------VHLP VDG----VPP RRQGAH----

**GRMZM2G428** SGISHYLFFA LVQGVYLFQG VSNDLIFQLV SEMNAEYFAP REDVILQNEA

**GRMZM5G838** SSISLYLFFP VVQGSYLFKG VSSGFIQQLA TEMQPEYFAP KEDIMLQNDK

**GRMZM2G095** SGIAQHLFRD TVEGAYLFRG VSEGLVVDLV ADVTPQFFPP KADIVQQNET

**ZmCNGC1**  REIKRHLCLS LLMKVPMFEN MDEQLLDAMC DRLKPMLYTE GSCIIREGDP

**ZmCNGC2**  RDIKRHLCLK LLMRVPLFEN MDEQLLDAMC DCLKPILYTE GSCVIREGDP

**ZmCNGC3**  RDIKRHLCLS LLKRVPMFEN MDDQLLDAMC DRVKPMLYTE GSHIVREGDP

**ZmCNGC4**  RDIKRHLCLG LVKRVPLFEN MDERLLDAIC ERLRPALYTE NEFILREGDP

**ZmCNGC5**  RDIKRHLCLG LVRRVPLFAN MDERLLDAIC ERLKPSLCTE HTYITREGDP

**ZmCNGC6**  RDIKRHLCLG LVRRVPFFSQ MDDQLLDAIC ERLVSSLCTK GTYIVREGDP

**ZmCNGC7**  RDIKRHLCLD LVRRVPLFDE MDERMLDAIC ERLRPALYTR GTRLMRELDP

**ZmCNGC8**  RDVQRHLCLD LVRRVPFFSE MDNQLLDAIC ERLVSFLCPE NTYISREGDP

**ZmCNGC9**  RDIRRHFFR- FLNKVRLFTL MDWPILDAIC DKLRQNLYIS GSDILYQGGT

**ZmCNGC10**  RDIKRYLCLE LVKQVPLFHG MDDLILDNIC DRLRPLVFSS GEKVIREGDP

**ZmCNGC11**  RDIKRYLCLE LVKQVPLFHG MDDLILDNIC DRLRPLVLSS GEKVIREGDP

**ZmCNGC12**  RDIKYHLCLG LVRQVPLFQH MDDLVLENIC DRVKSLIFPK GEVIVREGDP

....|....| ....|....| ....|....| ....|....| ....|....|

460 470 480 490 500

**AC234152.1** DDVYVVVSGE VEVILFDGIY EQ---VQATL GARDIFGEVS ALSDRAQA-F

**GRMZM2G020** PDDVYVVVSG EVEVVLFDGV DER---VEAT LGTRNIFGEV SALSDRPQAP

**GRMZM2G081** AADIYIIVSG VANLITTANG NE---QVYEK VEEGDMFGEV GALCDIPQP-

**GRMZM2G178** AADIYVIVSG SVNLITTVNG NE---QVFGK VEERDMFGEV GALCDIPQP-

**GRMZM2G310** VDQLYFVCHG ALEGVGIGED GQE--ETLLM LEPESSFGEI SILCNIPQP-

**GRMZM2G093** PTDVYILVSG AVEERFVIDG VENKMQVQGV MYAGEIFGEI GALCSVPQP-

**GRMZM2G022** PTDFYILVTG SAELIELRNG GEQ---MAGV AKAGDVVGEI GVLCYRPQL-

**GRMZM2G028** VDQIYIVAHG CLEEVATGEG GSE--DIISE LLPYDIVGDV SVVCNVPQP-

**GRMZM2G428** PSDFYILVTG ---------- -SV---VAGV ARAGDVVGEI GVLCYKPQL-

**GRMZM5G838** PSDMYLLVSG AVDILTFLDG TE---QVYGK AAEGELLGEV GVMSNKPQP-

**GRMZM2G095** PTDFYIIVSG SVDVLATAPD GTE--TVVSR ACRRGMAGEI GVMLNIPQP-

**ZmCNGC1**  VNEMLFIMRG TLESTTTNGG QTG-FFNSNV LKGGDFCGEE LLTWALDPT-

**ZmCNGC2**  VNEMLFVMRG NLMSMTTNGG RTG-FFNSDV LKAGDFCGEE LLTWALDPT-

**ZmCNGC3**  VNEMFFIMRG RLESTTTDGG RAG-FFNSNV LEGGDFCGEE LLTWALDPA-

**ZmCNGC4**  VDEMHFILHG CLESVTTDGG RSG-FFNKVQ LKEGSFCGDE LLTWALDPK-

**ZmCNGC5**  VDQMVFIIRG SLESITTDGG RTG-FYNRSL LEEGDFCGEE LLTWALDPK-

**ZmCNGC6**  VTEMLFIIRG KLESSTTNGG RTG-FFNSIT LKPGDFCGEE LLGWALVPR-

**ZmCNGC7**  VDSMLFIIRG YLDSYTTQGG RSG-FFNSCR IGAGEFCGEE LLTWALDPR-

**ZmCNGC8**  VNEMLFIIRG KLESSTTNGG RSN-FFNSII LRPGDFAGEE LLTWALLPK-

**ZmCNGC9**  VEKMVFIVRG KLESISADGS K-------AP LHDGDVCGEE LLTWYLEHS-

**ZmCNGC10**  VQRMVFILQG KLR--STQPL TKG-VVATCM LGAGNFLGDE LLSWCLRRP-

**ZmCNGC11**  VQRMVFILQG KLR--STQPL TKG-VVATCM LGAGSFLGDE LLSWCLRRP-

**ZmCNGC12**  VKRMLFIVRG HLQ--SSQVL RNG-AESCCM LGPGNFSGDE LLSWCLRRP-

....|....| ....|....| ....|....| ....|....| ....|....|

510 520 530 540 550

**AC234152.1** TFRTRTLSQL LRLKQATLKE AMQSRPEDSV VVIKNFLKHQ VEMHG-----

**GRMZM2G020** FTFRTRTLSQ LLRLKQATLK EAMQSWPDDS VIIIKNYVKH QVEMHG----

**GRMZM2G081** FTCRTTTLSQ LLRIRKIRLT EIMQEHKEDS KILMNNLFQK LKLHESLPEL

**GRMZM2G178** FTCRTSTLSQ LLRIRKIRLT EIMQEHREDS NIILNNLFQK LKLQENLPEL

**GRMZM2G310** YTVRVCELCR LLRLDKQSFT NILEIYFVDG RRILSNLSES EYG--GRVKQ

**GRMZM2G093** FTICTTKISQ LLRKGELILV LIIWQKTAQD --------QR FSTEVSGKFL

**GRMZM2G022** FTVRTKSLCQ LLRMNRTAFL SLVQSNVADG TIIMNNLMQL LKQQKDNSVM

**GRMZM2G028** HTVRVCDLCS LLRIDKQSLT SILQIYSKDS RQILSNLLKG RGGTESRGKQ

**GRMZM2G428** FTARTRSLCH LLRMERSAFL RIVQANVGDG TIIINNLIQY LKEKRDSGAI

**GRMZM5G838** FTFRTTRLSQ ILRIARSKLM DIMRENGEDG --------QI IRSNFQQAST

**GRMZM2G095** FTVRCRRLTQ AVRVSQGHLL RVVRPHTADA --------DR VFCNFVRQHL

**ZmCNGC1**  SASN------ ----LPGSTR TVKTLSEVEA FALRADDLKF VATQFRR---

**ZmCNGC2**  STSS------ ----LPSSTR TVKTMSEVEA FALRAEDLRF VATQFRR---

**ZmCNGC3**  SGSN------ ----LPSSTR TARTLSEVEG FSLRARHLRF VASQYRR---

**ZmCNGC4**  SAAN------ ----FPVSSR TVQALTEVEA FALCAEELKF VASQFRR---

**ZmCNGC5**  AGAC------ ----LPSSTR TVMALSEVEA FALHAEELKF VAGQFRR---

**ZmCNGC6**  PTTN------ ----LPSSTR TVKALIEVEA FALQAEDLKF VASQFRR---

**ZmCNGC7**  PAAK------ ----LPLSTR TVRAVSEVEA FALVADDLRF VASQFRR---

**ZmCNGC8**  TNVH------ ----FPLSTR TVRSHTEVEA FALRAEDLKF VANQFRR---

**ZmCNGC9**  SANRDGGKIK FQGMRLVAIR TVRCLTNVEA FVLRASDLEE VTSQFARF--

**ZmCNGC10**  FVDR------ ----LPASSA TFECVEAAQA FCLDAPDLRF ITEHFRYK--

**ZmCNGC11**  FVDR------ ----LPASSA TFECVEAAQA FCLGAPDLRF ITEHFRYN--

**ZmCNGC12**  FLER------ ----LPGSSS TLATLESTEA FGLDAADVKY VTQHFRYT--

....|....| ....|....| ....|....| ....|....| ....|....|

560 570 580 590 600

**AC234152.1** ---MKVEDLL GDNTGEHDDD ANVLTVAAMG NSGLLEDLLR AGKDADVGDA

**GRMZM2G020** ----MKADDS LGDNTSEHDD DANVLTVAAM GNSGLLEDLL RAGKDADVGD

**GRMZM2G081** KQLDRRFMHK YELFHAPQEA WLLPQPYLQY T-EHKLEDIG KKIPTFCGDH

**GRMZM2G178** NQLDRRFMHK YELFHATREA WLLPQPYLQY T-EHKFEDIS KKAPAFGGDH

**GRMZM2G310** LESDITFHIG KQE----AEL TLRVNSAAFY GDLHQLKSLI RAGADPKNTD

**GRMZM2G093** GKLNQEFRK- ---------- ---PNNYSAF N-QVGQENES E-------AK

**GRMZM2G022** VGVLKEIENM LAR--GRLDL PITLCFAVNK GDDLMLHQLL KRGLDPNESD

**GRMZM2G028** LESDIAYLIS RQE----AEL VLGVNNAAYH GDLSRLKGLV SAGADPSKPD

**GRMZM2G428** AGVAEEIEYL LAR--GQLEL PVTLCYAASR GDDFLMHQLL KRGVDPNESD

**GRMZM5G838** GYVGARFCHA PSSAIRVSTA ILAPAPLSIE EGRTPFQGLP HHS----RPR

**GRMZM2G095** GSP------- ---------- ---------- ---------- ----------

**ZmCNGC1**  ---------- ---------- ---------- ----LHSKQL QHTFRFYSQQ

**ZmCNGC2**  ---------- ---------- ---------- ----LHSKQL QHTFRFYSQQ

**ZmCNGC3**  ---------- ---------- ---------- ----LHSKQL RHTFRFYSHQ

**ZmCNGC4**  ---------- ---------- ---------- ----LHSRQV QHTFRFYSQQ

**ZmCNGC5**  ---------- ---------- ---------- ----MHSKAV QHTFRFYSQQ

**ZmCNGC6**  ---------- ---------- ---------- ----LHSKKL QHTFRYYSHH

**ZmCNGC7**  ---------- ---------- ---------- ----LHSARI RHRFRFYSHQ

**ZmCNGC8**  ---------- ---------- ---------- ----LHSKKL QHTFRFYSHH

**ZmCNGC9**  ---------- ---------- ---------- ----LRNPRV QGAIRYESPY

**ZmCNGC10**  ---------- ---------- ---------- ----FANEKL RRTARYYSSN

**ZmCNGC11**  ---------- ---------- ---------- ----FANEKL KRTARYYSSN

**ZmCNGC12**  ---------- ---------- ---------- ----FTNDKV RRSARYYSPG

....|....| ....|....| ....|....| ....|....| ....|....|

610 620 630 640 650

**AC234152.1** KGRTALHIAA SKGYEDCVLV LLKHACNVNI R--------- ----------

**GRMZM2G020** AMGRTALHIA ASKGYEDCVL VLLKHACNAN IR-------- ----------

**GRMZM2G081** GSTKLAAETN QMRLTQQGNS HDHGNYMATD GM-------- ----------

**GRMZM2G178** GSTKLAAETI QLRVPQQGNS HDHGNFGATD RM-------- ----------

**GRMZM2G310** YDGRSPLHLA ASRGYEDVVQ FLVNEGVDMD LT-------- ----------

**GRMZM2G093** GRVTSCCRNE RCKELNESER CNHVTIHKTA KQ-------- ----------

**GRMZM2G022** NNGHTALHIA ASKGDEQCVK LLLDYGADPN AR-------- ----------

**GRMZM2G028** HDGRTALHVA ALRGYEDIVR FLVQRGANVN SI-------- ----------

**GRMZM2G428** NYWHTALHVS ASGGHEQCIK LLLEHGADPN ASGACDWGHQ PNLALIVIQC

**GRMZM5G838** PLSGILAPTR PRHPCPPTRK SSAPLTLPTS SR-------- ----------

**GRMZM2G095** ---------- ---------- ---------- ---------- ----------

**ZmCNGC1**  WRTWAACFIQ AAWHRYCRKK LEEALYEKEK RLQ------- ----------

**ZmCNGC2**  WRTWAACFIQ AAWHRYCRKK IEDSLREKEK RLQ------- ----------

**ZmCNGC3**  WRTWAACFVQ AAWHRYCRRR LEEGVREKER MFR------- ----------

**ZmCNGC4**  WRTWAACFIQ AAWRRYYKRK MAEQRRKEEE ---------- ----------

**ZmCNGC5**  WRTWAATYIQ AAWRRHLKRR AAELRRREDE ---------- ----------

**ZmCNGC6**  WRTWASCFIQ AAWRRYKRRK MAKDLSMRES FNS------- ----------

**ZmCNGC7**  WRTWAACFIQ AAWRRYKRRR ASMELRVREV R--------- ----------

**ZmCNGC8**  WRTWAACFIQ AAWRQHQRRK LAESLSRWES YSW------- ----------

**ZmCNGC9**  WRTIAATRIQ VAWR-YRKRR LKR------- ---------- ----------

**ZmCNGC10**  WRTWAAVNIQ LAWRRYRARA STDLAAMAAP ---------- ----------

**ZmCNGC11**  WRTWAAVNIQ LAWRRYRART S---ADLAAP ---------- ----------

**ZmCNGC12**  WRTWAAVAVQ LAWRRYKHRK TLASLSFIRP RR-------- ----------

....|....| ....|....| ....|....| ....|....| ....|....|

660 670 680 690 700

**AC234152.1** DAQGNTAMWN AIAAGHHKTF NLLYQFGRAS NPRAGGDVMC LAARRGHLGA

**GRMZM2G020** -DAQGNTAMW NAIAAGHHKI FNILYHSARA SNPHAGGDVM CLATRRGDLD

**GRMZM2G081** ---------- ---------- ---------- --AGEEGERN EVHINCET--

**GRMZM2G178** ---------- ---------- ---------- --ASGDDKRN DLHINCETKK

**GRMZM2G310** -DQFGNTPLL EAVKQGQERV AALLFAKGAK LSLKNAGSHL CTAVAKGDSD

**GRMZM2G093** ---------- ---------- ---------- --D-DFNIIN SFPAKGES--

**GRMZM2G022** -DSEGKVPLW EALCEKHNAV IELLVESGAE LSSGDTALYA CIAVEENDAE

**GRMZM2G028** -DKFGNSPLL LALKSGHERI TSLLAKHGAA LNLEDAGGYL CRVVTDGKVD

**GRMZM2G428** PDAQGRVPLW EALSRGHRGA AQLLADGGAD LASGDAALYA RAAVEAGDVA

**GRMZM5G838** ---------- ---------- ---------- --KGAPSPAP VVSAPSLDPW

**GRMZM2G095** ---------- ---------- ---------- ---------- ----------

**ZmCNGC1**  ---------- ---------- -----AAIVS DGTTSLSLGA ALYASR----

**ZmCNGC2**  ---------- ---------- -----FAIAN DSSTSLSFMA ALYASR----

**ZmCNGC3**  ---------- ---------- -----AAAVT DISSSRSLGA ALYAAH----

**ZmCNGC4**  ---------- ---------- ------AASR PSSSHPSLGA TIYASR----

**ZmCNGC5**  ---------- ---------- ------ELEE DEGKSNRIRT TILVSR----

**ZmCNGC6**  ---------- ---------- -----VRLDE VDNEDDDSPP KNS-------

**ZmCNGC7**  ---------- ---------- ---------- ---------- ----------

**ZmCNGC8**  ---------- ---------- -----WSAED HPTGDKPRQE GTSSGGGGTR

**ZmCNGC9**  ---------- ---------- ---------- ---------- ----------

**ZmCNGC10**  ---------- ---------- ---------- ---------- ----------

**ZmCNGC11**  ---------- ---------- ---------- ---------- ----------

**ZmCNGC12**  ---------- ---------- ---------- ---------- ----------

....|....| ....|....| ....|....| ....|....| ....

710 720 730 740

**AC234152.1** LQELLKLGLD VD-SEDHDGA TALRVAMAEG HADAARFLIL NGAS

**GRMZM2G020** ALRELLKLGL DVD-SEDHDG ATALRVAMAE GHADAARFLI TNGA

**GRMZM2G081** --KEVSIHIN SEDCDAASHW QTDHETVRLG SSHNTSDGIT MREN

**GRMZM2G178** GTKEFCIQIK SKDCEAASYW QTSPETVKLG SSHSTLDAIT TREN

**GRMZM2G310** FIRRTLAYGA DPN-CRDYDH RTPLHIAAAE GLYLIAKMLV EAGA

**GRMZM2G093** --KEKHILTN LMFMESVYRG EADVHRQILP DSS------L TRSE

**GRMZM2G022** LLENVIRYGG NIN-NPTKDG TTPLHRAVCD GNVQMVELLL EHGA

**GRMZM2G028** LLSRLLRSGV DPN-CRNYDQ RTPLHVAAAE GLHLVASMLV GFGA

**GRMZM2G428** LLEDVARHGG DVTVACCDDG GTALHRAVLQ GDVGMARALL EHGA

**GRMZM5G838** FTAPSLDPGK RRRVAARGDW KMTPFVVDLG FGEESILRGL TEAN

**GRMZM2G095** -------VWK VARKETPLFG EIADELEVGA ATAASMSRRR SEMF

**ZmCNGC1**  -FAGNMMRIL RRNATRKARL Q-ERVPARLL QKPAEPNFFA EDS-

**ZmCNGC2**  -FAGNMIRIL RRNATRKARL Q-ERVPARLL QKPAEPNFSA EEQ-

**ZmCNGC3**  -FARNMVRTL RRNAARKARL L-DTVSSRLL QKPAEPNFFA EED-

**ZmCNGC4**  -FAANAMRGV HRLRSKAV-- ---PTIVRLP KP-PEPDFGV DDAD

**ZmCNGC5**  -FAANAMRGV HRQRSRRA-- ---VAVSELL MPMPKPR--- ----

**ZmCNGC6**  -LALKFIART RKVPQNMK-- -----ELPKI TKPDEPDFSA EPED

**ZmCNGC7**  --AGGSLLRS RRHSIEGK-- -------ASI RKPMEPDFTV EEED

**ZmCNGC8**  TIAEGAIAHM HKLASASRRF RTEDVAIRRL QKPDEPDFSA DHFD

**ZmCNGC9**  --AEKSRLSE ETYASLGS-- ---------- ---------- ----

**ZmCNGC10**  PLAGGPDDGD RRLRHYAA-- ------MFMS LRP-HDHLE- ----

**ZmCNGC11**  PLVGGPDDGD RRLRHYAA-- ------MFMS LRP-HDHLE- ----

**ZmCNGC12**  PLSRCSSLGE EKLRLYTA-- ------LLTS PKPNQDDLL- ----
