# Supplementary material for: Genome-wide identification and analysis of the CNGC gene family in maize
Source: PeerJ. 2018 Oct 17;6:e5816. doi: 10.7717/peerj.5816 (PMC6195792; doi:10.7717/peerj.5816)
Supplement: File S2 — Residues in highlighted indicate most conserved among these CNGCs. [file peerj-06-5816-s002.pdf]

Supplemental File 2

Multiple sequences alignments among maize, *Arabidopsis* and rice CNGCs by using T-COFFEE method.

Residues in highlighted indicate most conserved among these CNGCs

```
                1                                                                     10
AT5G53130       MNF.R.............................................QEKF............V...RF..QD..............
AT3G48010       MSN.LHL.YT...........................................S............A...RF..RNF.............
AT2G23980       MFD.TCGPKGV...KSQ...V........I.........SGQ........RENF............V...RL..DS..............
AT2G24610       MEF.KR.............................................DNT............V...RF..YGDEKQT.I....EVG
AT5G54250       MAT.EQE.F.TRA.SRF............SRDSSSVGYYSEE..........D..NTEEEDEEEEME...EIEEEE..............
AT2G28260       MGY.G.............................................NSRS............V...RF..QE..............
AT5G15410       MPS.HPN.FIFR....W............I.........GLF........SDKFRRQTTGIDENSNL...QI..NG..............
AT1G15990       MYK.SQY......................I.........SGQ........REKF............V...RL..DD..............
AT4G30560       MLD.CGK.KAV...KSQ...V........I.........SGR........LEKF............V...RL..DS..............
AT5G57940       MAG.K.............................................RENF............V...RV..DD..............
AT3G17700       MAS.HNE.NDD.I.PMLPISD........P.........SSRTRARAFTSRSRS...V............SL..SNPTS........SI.
AT1G01340       MIL.F.............................................R....................F..KD..............
AT1G19780       MYK.SQY......................I.........SGH........REKF............V...RL..DD..............
AT2G46450       MNH.R.............................................RSKF............A...RI..DS..............
AT4G30360       MEL.RK.............................................DKL............L...MF..YSEGKESKEAKWAVN.
AT5G14870       MNK.IRS.LR...........................................C............L...LP..ETI.............
AT4G01010       MAF.G.............................................RNNR............V...RF..RD..............
AT3G17690       MAH.TRT.FTS.......................................RNRS...V............SL..SNPSF........SI.
AT2G46430       MMNPQ.............................................RNKF............V...RF..NG..............
AT2G46440       MNL.Q.............................................RRKF............V...RL..DS..............
LOC_Os06g33570  MMMGR.............................................EDKY............V...RF..ED..............
LOC_Os06g33610  ..........................................................................................
LOC_Os03g44440  MSY.ASG.GGG...............GGEL.........ATK........RSAF....................................
LOC_Os12g28260  MFG.SCG.GGY...RTQ...T........I.........NGR........KGTF............V...RL..EQ..............
LOC_Os04g55080  MFD.SAH.KA......Q...Y........I.........DGQ........REMF............K...RL..DE..............
LOC_Os02g41710  MAC.N.............................................GSRA............V...RF..QN..............
LOC_Os12g06570  ..........................................................................................
LOC_Os09g38580  MFG.SRV.QD..EVEMQ.................................RRTT...N.............................RIF
LOC_Os02g54760  MFG.AGK.VDD.E................M.........ALK........RQRT............V...RF..YD..............
LOC_Os06g08850  MFG.SRR.VKD.E................M.........ELR........KQRT............V...RF..HEE.............
LOC_Os02g53340  MSD.QER.DD..I.PMLLR......................NVELPTFPP..RS...T............SM..CIPVR.........D.
LOC_Os06g10580  MSG.QER.DD..V.PML..........................ELQRFPT..RS...V............SM..CIPVR.........D.
LOC_Os03g55100  MPS.LSF...LR....F............L.........SGR................................................
LOC_Os01g57370  MAS.SSA.A..AA.SS.....................AH......................G.VGVVQ..RLWLEE..............
LOC_Os05g42250  MSG.ELS.T..RA.ST......................S...................................................
GRMZM2G005791   MDC.DLF.AAW.W..........................SSS........TRLV............S...RI..FRGSAD........AP
GRMZM2G023037   MFD.STQ.KA......Q...Y........M.........DGH........RERF............I...RL..DE..............
GRMZM2G066269   V.........................................................................................
GRMZM2G068904   MAS.G.............................................ASRN............V...RF..QN..............
GRMZM2G074317   MPR.LAF...LRR...S............L.........PAR................................................
GRMZM2G077828   MSY.DQS.AFQ....VD...YMGVGAGAGV.........SAS........RRRF....................................
GRMZM2G090528   MHA.......................................................................................
GRMZM2G129375   ..........................................................................................
GRMZM2G135651   MYY.SSI.VTT.LVRNH...T........I.........STQ........NHWF...L...LYLYFLRFCYF..SNF.LFI.....FRIF
GRMZM2G141642   ..........................................................................................
GRMZM2G148118   MAG.R.............................................EERY............V...RF..HD..............
GRMZM5G858887   MPP.LAF...LRR...Y............L.........PAR.....................NR.V...SV..ID..............
consensus>70    m.........................................................................................

                                                                        20
AT5G53130       ..WK..SD.K..............................................................TSS...............
AT3G48010       ...P..TT.F..............................................................SLR...............
AT2G23980       ..MD..SR.Y..............................................................SQS...............
AT2G24610       E........K..............................................................RVP...............
AT5G54250       ..EE..EE.E................................................................................
AT2G28260       ..DQ..EV.V....HG..................................................GES...GVK...............
AT5G15410       ..GD..SS.S..............................................................SGS...............
AT1G15990       ..ID..SS.S..............................................................SPA...............
AT4G30560       ..MD..SR.Y..............................................................SQT...............
AT5G57940       ..LD..SR.L..............................................................PSS...............
AT3G17700       EGFD..TS.TVVLGYTGPLRTQR.RPPLVQMSGPLTSTRKHEPLFLPHPS....SDSVGVSSQPERYPSFAALE................
AT1G01340       ..EG..KPLS..............................................................SE................
AT1G19780       ..TD..SR.V..............................................................SMS...............
AT2G46450       ..MG..VD.G................................................................................
AT4G30360       DPMSKSYK.L..............................................................SLP...............
AT5G14870       ...T..SA.S..............................................................TAA...............
AT4G01010       ..WI..SE.G..............................................................TE................
AT3G17690       DGFD..NS.TVTLGYTGPLRTQRIRPPLVQMSGPIHSTRRTEPLFSPSPQ....ESPDS.............SST...............
AT2G46430       ..ND..DE.F..............................................................ST................
AT2G46440       ..TG..VD.G................................................................................
LOC_Os06g33570  ..WR..SE.Q..............................................................SVM...............
LOC_Os06g33610  ..........................................................................................
LOC_Os03g44440  ..........................................................................................
LOC_Os12g28260  ..QE..DQ.E..............................................................RQP...............
LOC_Os04g55080  ..SS..PR.S..............................................................SVP...............
LOC_Os02g41710  ..DM..EL.P....HW...................................................KT..SSVP...............
LOC_Os12g06570  ..........................................................................................
LOC_Os09g38580  PDER.QDQ.F..............................................................KLP...............
LOC_Os02g54760  ..EK..AK.P..............................................................TIP...............
LOC_Os06g08850  ...R..AK.P..............................................................TIP...............
LOC_Os02g53340  DEYE..ED.TF.VPHTGPLFVQP...PTQTAAAGIPFTNTP..DMPPRPPQGKQVNKPH............AIMP...............
LOC_Os06g10580  DIYE...D.SI.ISHSGPIFTPA...PTQYTSVAIPSGN.R..DMLDKLPRPKVKSKPH............VVTP...............
LOC_Os03g55100  ................................................................................SLADVCDGVK
LOC_Os01g57370  ..Q...ER.K..............................................................PPP...............
LOC_Os05g42250  ..S...SS.S..............................................................SPP...............
GRMZM2G005791   GPSP..AR.P..............................................................AMP...............
GRMZM2G023037   ..SS..PR.S..............................................................SVP...............
GRMZM2G066269   ..........................................................................................
GRMZM2G068904   ..EI..EV.Q....SF..................................................RTSPLQSLS...............
GRMZM2G074317   ................................................................................LLARACGGGG
GRMZM2G077828   ..........................................................................................
GRMZM2G090528   ..........................................................................................
GRMZM2G129375   ..........................................................................................
GRMZM2G135651   PDER.QNQ................................................................SKS...............
GRMZM2G141642   ..........................................................................................
GRMZM2G148118   ..WK..SE.Q..............................................................SVS...............
GRMZM5G858887   ..PE..LR.L..............................................................SVCFPEQRLLARACDGGV
consensus>70    ..........................................................................................
```

```
                                                            30          40
AT5G53130       ............DVEY...............SG..K..NEIQTGIFQRTISSI..............................SDK
AT3G48010       ............H..........................................................................
AT2G23980       ............SETGL..............N..K..C...........TLN.........I..QGGPKRFA..QGSKA..SSGS
AT2G24610       ............LFK..............................................................S.......TTA
AT5G54250       ...........................................................................................ED
AT2G28260       ..........................................................................................L.K
AT5G15410       ............DETP.......V.....LSS.VECYA...........CTQ........V...GV.PAFH...STSCD..QAHA
AT1G15990       ............TGM..............MMQ..R..N...........CFG.........F..NL.KNRG...GEKKK..ASKS
AT4G30560       ............SDTGL..............N..R..C...........TLN.........L..QG.PTRGGGAQGNNV..SSGS
AT5G57940       ............SVAFQ..............Q..N..Y...........ASN.........F..SG.QLHPI..HASNE..TSRS
AT3G17700       ............HKNS..................SEDEFVLKHANLLR..SGQLGMCNDPYCTTC.PSYY...N.........
AT1G01340       ..............YGY..............G.....R...........KAR........PS...L.D......RV......FKN
AT1G19780       ............SNATG.............MKK..R..S...........CFG........LF..NV.TSRGG..GKTKN..TSKS
AT2G46450       .............................................................................K......LKS
AT4G30360       ............SA............................................................................LRP
AT5G14870       ............S..............................................................................
AT4G01010       ..............YGY..............G..R..N...........KAR........PS...L.N......TV......LKN
AT3G17690       ............VDVP..................PEDDFVFKNANLLR..SGQLGMCNDPYCTTC.PSYY...N.........
AT2G46430       ......................................K...........TTR........PS...V.S......SV......MKT
AT2G46440       .............................................................................K......LKS
LOC_Os06g33570  ............SPR......................RH.......NA.......................................
LOC_Os06g33610  ...............................................................................................
LOC_Os03g44440  ................H..............I.................D.........Y.......GGG.VSLRRLAQPEA
LOC_Os12g28260  ............AATYT..............M.................D.........G.......SGGG.GRVQH..VMDS
LOC_Os04g55080  ............SEVG...............G..R..S...........TLK.........F...SM.PSFG...YDSFN..PVRS
LOC_Os02g41710  ............ECTS...............S..S.....R........STK.........H...GK.AQHQ...QQQHH..DPRK
LOC_Os12g06570  ...............................................................................................
LOC_Os09g38580  ..............FQ.........................................................A.......ARA
LOC_Os02g54760  ............THQ...............................................................................KQ
LOC_Os06g08850  ............THQ...............................................................................KQ
LOC_Os02g53340  ............EEIG...............G..NRWSYSGNVPKNEHLMM..SGPLGQCDDPDCVNC.PPAC...K.........
LOC_Os06g10580  ............EEVG...............I..SNWPYDQHVPKNKHLMM.YSEPLGLCDNPDCVDC.PRAC...K.........
LOC_Os03g55100  RRLG.LGDDEGRDEEAGLAGGSSRPAAAAAVAGPP.GECYA...........CTQ........P...GV.PSFH...STTCD..QVHS
LOC_Os01g57370  ...................................K...................................R.............GG.
LOC_Os05g42250  ...................................GDARG..................................PEH............GGT
GRMZM2G005791   ............LHQ...............................................................................KQ
GRMZM2G023037   ............SEVG...............G..R..S...........TLR.........S...SM.PGFG...YGPFN..ALRS
GRMZM2G066269   ...............................................................................................
GRMZM2G068904   ............RKH..................................................GK.A..........H..DPRK
GRMZM2G074317   GGDQGSPDQVARDEEAGGSGGMSG...RSSAGGPSGGECYA...........CTQ........P...GV.PAFH...STTCD..QVHS
GRMZM2G077828   ..........................................................................................MPSES
GRMZM2G090528   ...............................................................................................
GRMZM2G129375   ...............................................................................................
GRMZM2G135651   ............LYQ..........................................................T.......TRA
GRMZM2G141642   ...............................................................................................
GRMZM2G148118   ............VIS................D..RVVSEKGH........NI.......................................
GRMZM5G858887   RG...SP.GVARDEEAGGSGGLSG...RSA.GAPS.GECYA...........CTQ........P...GV.PAFH...STACD..QVHS
consensus>70    ...............................................................................................

                               50          60                     70                    80          90
AT5G53130       F..Y........RS.FE.SSSAR.I..KLFKRS..YKS............YSFK.E..AVS....KGI......GSTHKILDPQ..GPFL
AT3G48010       ..........................................................H.HNDPNNQRRRSIFS......KLRDKTLDPG..GDLI
AT2G23980       F..K........KG.FR.KGSEG.L..WSIGRS..IGL............GVS.RA..VFPE...DLE......VSEKKIFDPQ..DKFL
AT2G24610       PFMK........QEVLP.KK.......SKTRLK..IPR............FGR.FK..VFPE...NFE......IERDKILDPG..GDAV
AT5G54250       PR...................I.G..LT..C.................GG.RRN..GSSN...NNK.....WMMLGRILDPR..SKWV
AT2G28260       F.KI........NG.TQ.INNVK.M..MSKGKF..LKA............KVL.SR..VFSE...DLE......RVKTKILDPR..GQTI
AT5G15410       PE..........WR.AS.AGSSL.V..PIQEGS..VP.............NP..AR..TRFR...RLK......GPFGEVLDPR..SKRV
AT1G15990       F..R........EG.VKKIRSEG.L..ITIGK..................SVT.RA..VFPE...DLR......ITEKKIFDPQ..DKTL
AT4G30560       F..K........KG.FR.KGSKG.L..WSIGRS..IGL............GVS.RA..VFPE...DLK......VSEKKIFDPQ..DKFL
AT5G57940       F..K........KG.IQ.KGSKG.L..KSIGRS..LGF............GVY.RA..VFPE...DLK......VSEKKIFDPQ..DKFL
AT3G17700       ..R.........KA.......AQ.I..PTSRVS..ALFDSTFHNALYDDAKGW.AR..RFAS...SVN......RYLPGIMNPH..AKEV
AT1G01340       V..K............WG.........FKKP..LSF............PSHKDP..DHKE...TSS......VTRKNIINPQ..DSFL
AT1G19780       F..R........EG.VK.IGSEG.L..KTIGKS..FTS............GVT.RA..VFPE...DLR......VSEKKIFDPQ..DKTL
AT2G46450       V..R...........................................................................GR.LKKVYGK..MKTL
AT4G30360       ............DNLLP.............GNRLR..YT............DA.SK...SKS...SKV......SWYKTILDPG..SEIV
AT5G14870       ..................................................NR.GS..DGSQ...FSV......LWRHQILDPD..SNIV
AT4G01010       V..R...............RG.........LKKP..LSF............GSHNKK..RDSN...SST......TTQKNIINPQ..GSFL
AT3G17690       ..R.........QA.......AQ.L..HTSRVS.....ASRFRTVLYGDARGW.AK..RFAS...SVR......RCLPGIMNPH..SKFV
AT2G46430       V..R........RS.FE.KGSEK.I..RTFKRP..LSV............HS..NK..NKEN...NKK......KKILRVMNPN..DSYL
AT2G46440       V..R...........................................................................GR.LKKVYGK..MKTL
LOC_Os06g33570  ..L.........SS.LK.ERTAGVF..AFLGNL..VHS............ETLK.R..LVL....HER......KLTTRTLHPQ..GPFL
LOC_Os06g33610  ..............................................................................................
LOC_Os03g44440  L..........ARGMIT.QGSAQ.L..RTLGRS..LRT............GAAMAV..VFQE...DLK......NTSRKIFDPQ..DRLL
LOC_Os12g28260  Y..FSSAPKIRTRS.VR.MAAAG.V..MSIGGYRAERL.............KS.IGR..VFQE...DLT......NMSQKIFDPQ..DAFL
LOC_Os04g55080  F..L........SG.VR.KGSGR.L..KSLRQS..LTS............GAP.KT..AFAE...DLK......SFKKTIFDPQ..EKFL
LOC_Os02g41710  W..R........RG.GG.G............GGS..LKD............RVL.SR..AFSE...ELESLMS.SGANHLFFDPR..GQLI
LOC_Os12g06570  ..............................................................................................
LOC_Os09g38580  D............RF...............GVNRID..AKT............TEK.IK..VISE...GNI......PWHRRILDPG..SSMV
LOC_Os02g54760  A...........GF................AASKLG..VAS............SGK.NK..IFVP...GEE......LWYKRILDPS..SDFI
LOC_Os06g08850  A...........GL................ATSKLG..LGI............SEK.NK..IFLA...GNE......LWYKKIIDPS..SDFI
LOC_Os02g53340  ..NK........RH................FHRGS..STLDSKFHNFLCEHGGGW.KK..E..I...ERF......LSRIPVMNPH..AKVV
LOC_Os06g10580  ..NK........RH................FQRSL..APFDNKFHNILYGYGDRW.KK..K..A...GHY......LSYIPIMKPH..DKAV
LOC_Os03g55100  PD..........WD.AD.AGSSL.V..PVQAQP..S..............AA..HH..AAAA...AAR......WVFGPVLDPR..SKRV
LOC_Os01g57370  .........................................................G...K.......RRWAWAPLEPRRAGWWA
LOC_Os05g42250  PR..........GE.VS.SKRRL.V..LR..R...................RQ.RWR..RLGG...G.......AAASWAAADPR..ARWV
GRMZM2G005791   A...........GL................AASKLG..VGT............SKK.HR..AFVA...SDE......QWYNKIFDPS..SDFI
GRMZM2G023037   F..L.............S.GGSGR.L..KSLRQS..LTS............GAP.KT..AFAE...DLK......SYKRTIFDPQ..DKLL
GRMZM2G066269   ..............................................................................................
GRMZM2G068904   C..R........LG.FR.GGCLE.KACRNRKPM..LKD............RVL.SR..AFSE...ELESLMHAAGGSHLFFDPR..GQLI
GRMZM2G074317   PD..........WD.AD.AGSSL.V..PVQGQA..QAA............AAAAAP..RQRH...AAR......WLLGPVLDPR..SRRV
GRMZM2G077828   L..........ARGVIT.HGSAQ.L..RTIGRS..LRA............GATMAA..VFQE...DLK......NTSRRIFDPQ..DPVL
GRMZM2G090528   ..............................................................................................
GRMZM2G129375   ..............................................................................................
GRMZM2G135651   D............RF...............GANRID..LKN............PEK.LK..VLNE...SNK......PWHQRILDPG..SNIV
GRMZM2G141642   ..............................................................................................
GRMZM2G148118   ...F........GL.LK.DRTAGAF..SFLGNS..SHS............EALN.K..L.G....LGE......KSKTKVLDPQ..GPFL
GRMZM5G858887   PD..........WD.AD.AGSSL.V..PVQAQQ..QAQ............PA..AA..AAQH...AAR......WLFGPVLDPR..SKRV
consensus>70    ...............................................................................i.dp......
```

```
                     100       110       120            130       140       150       160              170
AT5G53130       QRWNKIFVLACIIAVSLDPLFFYVPIIDD.....AKKCLGIDKKMEITASVLRSFTDVFYVLHIIFQFRTGFIAPSS..R.....VFGRG
AT3G48010       TRWNHIFLITCLLALFLDPLYFYLPIVQ.....AGTACMSIDVRFGIFVTCFRNLADLSFLIHILLKFKTAFVSKSS..R.....VFGRG
AT2G23980       LLCNKLFVASCILAVSVDPLFLYLPFIND.....KAKCVGIDRKLAIIVTTIRTVIDSFYLFHMALRFRTAYVAPSS..R.....VFGRG
AT2G24610       LQWNRVFLFWCLVALYVDPLFFFLSSVKR....IGRSSCMTTDLKLGIVITFFRTLADLFYVLHIVIKFRTAYVSRT..SR.....VFGRG
AT5G54250       REWNKVFLLVCATGLFVDPLFLYTLSVS.....DTCMCLLVDGWLALTVTALRSMTDLLHLWNIWIQFKIARRWPYP..GGDSDGDTNKG
AT2G28260       RRWNKIFLIACLVSLFVDPLFFFLPVMR......NEACITIGVRLEVVLTLIRSLADAFYIAQILIRFRTAYIAPPS..R.....VFGRG
AT5G15410       QRWNRALLLARGMALAVDPLFFYALSIGR...TTGPACLYMDGAFAAVVTVLRTCLDAVHLWHVWLQFRLAYVSRES..L.....VVGCG
AT1G15990       LVWNRLFVISCILAVSVDPLFFYLPIVDN....SGSSCIGIDTKLAVTTTLRTIVDVFYLTRMALQFRTAYIAPSS..R.....VFGRG
AT4G30560       LLCNKLFVTSCILAVSVDPLFLYLPFVKD.....NEKCIGIDRKLAIIATTLRTVIDAFYLFHMALRFRTAFVAPSS..R.....VFGRG
AT5G57940       LYCNKLFVASCILSVFVDPFFFYLPVINA.....ESKCLGIDRKLAITASTLRTFIDVFYLAHMALQLRTAYIAPSS..R.....VFGRG
AT3G17700       QTWTKFFALSCLLAIFIDPLFFFLIKVQE.....QNKCIMIDWPMTKAFVAVRSVTDVIFTMNILLQFRLAYVARES..T.....VVGAG
AT1G01340       QNWNKIFLFACVVALAIDPLFFYIPIVDS.....ARHCLTLDSKLEIAASLLRTLIDAFYIIHIVFQFRTAYIAPSS..R.....VFGRG
AT1G19780       LLWNRMFVISCILAVSVDPLFFYLPIVDN.....SKNCIGIDSKLAVTTTLRTIIDVFYLTRMALQFRTAYIAPSS..R.....VFGRG
AT2G46450       ENWRKTVLLACVVALAIDPLFLFIPLIDS.....QRFCFTFDKTLVAVVCVIRTFIDTFYVIHIIYYLITETIAPRS..Q.....ASLRG
AT4G30360       LKWNWVFIVSCMVALFIDPLYFFVPAIGG...DKNYPCARTDTSLSILVTFFRTIADLFYLLHIFIKFRTGFIAPNSSTR.....VFGRG
AT5G14870       TYWNHVFLITSILALFLDPFYFYVPYVG......GPACLSIDISLAATVTFFRTVADIFHLLHIFMKFRTAFVARSS..R.....VFGRG
AT4G01010       QNWNKIFLFASVIALAIDPLFFYIPIVDG.....ERHCLNLHRNLEIAASVLRTFIDAFYIIHIVFQFRTAYISPSS..R.....VFGRG
AT3G17690       QVWTRVLAFSSLVAIFIDPLFFLLLIQQ......DNKCIAIDWRATKVLVSLRSITDLIFFINILLQFRLAYVAPES..R.....IVGAG
AT2G46430       QSWNKIFLLLSVVALAFDPLFFYIPYVKP.....ERFCLNLDKKLQTIACVFRTFIDAFYVVHMLFQFHTGFITPSS..S.....GFGRG
AT2G46440       ENWRKTVLLACVVALAIDPLFLFIPLIDS.....QRFCFTFDKTLVAVVCVIRTFIDTFYVIHIIYYLITETIAPRS..Q.....ASLRG
LOC_Os06g33570  QSWNKIFVLSCIFAVSVDPLFFYIPVIND.....NNTCWYLDKKLEITASVLRFFTDIFYILHIIFQFRTGYIASSL..T.....TFGRG
LOC_Os06g33610  ..........................................................................................
LOC_Os03g44440  VRLNRSFVVSCIVSIAVDPVFFYAPQVTA...NGGNLCVGISRDLAISASVVRTVVDLFFAARIVLQFRTAYIAPSS..R.....VFGRG
LOC_Os12g28260  VRMNRLFVMACIVSVAVDPLFFYLPAVTA...TDSNTCIGFDRGLATGATAVRSAIDLFYLARIALQFRTAYIAPSS..R.....VFGRG
LOC_Os04g55080  FQMNWFCFLSCVFAVAVDPLFFFLPIIDG...DDKSSCIGIDKKLAVTSTIIRTILDLVYLIRVFLQFRTAYVAPSS..R.....VFGTG
LOC_Os02g41710  HLWSKIFLAACLASLFVDPLFLYLTGTR......QNMCIELKYSLAFTLSMIRSLLDLFYAAHIFFRFRTAFIAPSS..R.....VFGRG
LOC_Os12g06570  ........................................MDMQIGVGVTAVRTVADLFYLAHMILKFRTAFVAPSS..R.....VFGRG
LOC_Os09g38580  LMWNRVFLGSCLFALFIDPFFYYLPLVHVLDESTNRSCIAKDRRLSITITVLRTFADLFYMLNIMVKFHTAYVDPKS..R.....VLGKG
LOC_Os02g54760  LTWNHIFLFSCFVALFIDPLYFYVPKISY...GTPNSCIGTDRHLAITVTFFRSISDLLYFTHIIIKFRTAYINPSSTMR.....VFGRG
LOC_Os06g08850  LTWNYVLRIACFVALFMDPLYFYVPKIYY...GTPNSCIGRDTRLAIIVTVFRSITDLFYVLQIIIKFRTAYINPSSTLG.....VFSRG
LOC_Os02g53340  QQWNQFFVISCLVAIFIDPLFFFLLSVQK.....DNKCIVLNWHFATALAVVRSVTDAIYFLHMLLQFRLAYVAPES..R.....VVGAG
LOC_Os06g10580  HRWNQFFVISCLLAIFNDPLFFFLLSVDK.....DYKCIVFNWNFAIALAVGRSVTDAIYFLHMLLQFRLAYVAPES..R.....VVGTG
LOC_Os03g55100  QRWNRWILLARAAALAVDPLFFYALSIGR....AGQPCVYMDAGLAAAVTALRTAADLAHLAHVLLQFRVAYVSRES..L.....VVGCG
LOC_Os01g57370  REWDRAYLLACAAGLMVDPLFLYAVSVS.....GPLMCVFLDGWFAAAVTVLRCTVDAMHAWNLLMRLRAAVRPPEE........DDGAD
LOC_Os05g42250  REWNRAYLLACAAGLMVDPLFLYAVSLS.....GPLMCVFLDGWLAAAVTALRCMVDAMHAWNIVTQLRVSRAGRER........ACAAG
GRMZM2G005791   LTWNRIFLFSCFVALFIDPLYFYVPKISY...GSPKFCIGTDTRFAVGVTFFRSIADLLYVLHIIIKFRTAYINPSSTLR.....VFGRG
GRMZM2G023037   FRMNWVFFSSCLFAVAVDPLFFFLPIIND......SNCIGIDKKLAVTSTIIRTVIDFVYLIRVCLQFRTAYVAPSS..R.....VFGTG
GRMZM2G066269   SKFN......C...............................................................................
GRMZM2G068904   HLWNKIFLSACLLSLFVDPLFLYLTGTQ......RNTCVEFKDSLALTLSMVRSLLDLFYAAHILFRFRTAFIAPSS..R.....VFGRG
GRMZM2G074317   QRWNRWILLGRAAALAVDPLFFYALSIGR....AGQPCLYMDAGLASAVTALRTCADVAHLAHVLLQLRLAYVSRES..L.....VVGCG
GRMZM2G077828   VRLNRAFFISCIVAIAVDPMFFYLPMVTD....EGNLCVGIDRWLAISTTVVRCVVDLFFLGRIALQFRTAYIKPSS..R.....VFGRG
GRMZM2G090528   ....................................ACVT..................................................
GRMZM2G129375   ........Y.................................................................................
GRMZM2G135651   LRWNRVYLVACLFALFIDPFFYYLPLIRQ..NGNGSSCVAKDQGLSIRITVLRSLADLFYMLNIAIKFHTAYVDPKS..R.....VLGKG
GRMZM2G141642   ..........................................................................................
GRMZM2G148118   QRWNKIFVISCLFAVFVDPLFLYVPVIDG.....GNNCLYLDKKLETTASILRFFTDIFYLLHILFQFRTGFIAPSS..R.....VFGRG
GRMZM5G858887   QRWNRWILLGRAAALALDPLFFYALSIGR....AGRPCLYLDAGLAAAVTALRTCADVAHLAHVLLQFRLAYVSRES..L.....VVGCG
consensus>70    ..wn......c.....vdplf.y...i..........c...d..l.......r...d..y.......fr.ayi...s........v.g.g
```

```
                                   180                             190       200       210         220
AT5G53130       VLV............EDKREIA.....................KRYLSS..HFIIDILAVLPLPQMVILIIIPHMRG.SS.SLNTKNMLKF
AT3G48010       ELV............MDRREIA.....................IRYLKS..EFVIDLAATLPLPQIMIWFVIPNAGE.FRYAAHQNHTLSL
AT2G23980       ELV............IDPAQIA.....................KRYLQQ..YFIIDLLSVLPVPQIIVWRFLYTSRG.AN.VLATKQALRY
AT2G24610       ELV............KDPKLIA.....................RRYLRS..DFIVDLIACLPLPQIVSWFILPSIRS.SH.SDHTTNALVL
AT5G54250       GGT............RGSTRVA.....................PPYVKK.NGFFFDLFVILPLPQVVLWVVIPSLLKRGS.VTLVVSVLLV
AT2G28260       ELV............IDSRKIA.....................WRYLHK..SFWIHLVAALPLPQVLIWIIIPNLRG.SP.MTNTKNVLRF
AT5G15410       KLV............WDPRAIA.....................SHYARSLTGFWFDVIVILPVPQAVFWLVVPKLIREEK.VKLIMTILLL
AT1G15990       ELV............IDPAKIA.....................ERYLTR..YFVVDFLAVLPLPQIAVWKFLHGSKG.SD.VLPTKTALLN
AT4G30560       ELV............IDPAQIA.....................KRYLQQ..YFIIDFLSVLPLPQIVVWRFLYISKG.AS.VLATKRALRS
AT5G57940       ELV............IDPAQIA.....................KRYLQR..WFIIDFLSVLPLPQIVVWRFLQSSNG.SD.VLATKQALLF
AT3G17700       QLV............SHPKKIA.....................LHYLKG..KFFLDLFIVMPLPQILILWIIPAHLGASG.ANYAKNLLRA
AT1G01340       ELV............DDAKAIA.....................LKYLSS..YFIIDLLSILPLPQIVVLAVIPSVNQ.PV.SLLTKDYLKF
AT1G19780       ELV............IDPAKIA.....................ERYLTR..YFIVDFLAVLPLPQIAVWKFLHGSKG.TD.VLPTKQALLH
AT2G46450       EIV............VHSKATL.....................KTRLLF..HFIVDIISVLPIPQVVVLTLIPLS...A.SLVSERILKW
AT4G30360       ELV............MDPKAIA.....................WRYIKS..DFIIDLIATLPLPQIVIWFVISTTKS.YR.FDHNNNAIAL
AT5G14870       ELV............MDSREIA.....................MRYLKT..DFLIDVAAMLPLPQLVIWLVIPAATN.GT.ANHANSTLAL
AT4G01010       ELV............DDPKAIA.....................IKYLSS..YFIIDLLSILPLPQLVVLAVIPNVNK.PV.SLITKDYLIT
AT3G17690       QLV............DHPRKIA.....................RHYFRG..KFLLDMFIVFPIPQIMILRIIPLHLGTRR.EESEKQILRA
AT2G46430       ELN............EKHKDIA.....................LRYLGS..YFLIDLLSILPIPQVVVLAIVPRMRR.PA.SLVAKELLKW
AT2G46440       EIV............VHSKATL.....................KTRLLF..HFIVDIISVLPIPQVVVLTLIPLS...A.SLVSERILKW
LOC_Os06g33570  VLV............EDRYAIA.....................KRYLST..YFLIDVFAVLPLPQVVILVVLPNLGG.SE.VTKAKNILMF
LOC_Os06g33610  ...........................................................................................
LOC_Os03g44440  ELV............IDTAQIA.....................ARYFRR..FFAADLLSVLPLPQIVIWKFLHRSKG.AA.VLSTKDALLI
LOC_Os12g28260  ELV............IDPAAIA.....................RRYVRR..FFVVDLLSVLPLPQIPIWNFLHRPKG.AD.LLPTKNALLF
LOC_Os04g55080  ELV............IDPMRIA.....................IRYLKS..YFVMDFFALLPLPQIVVWRYLHTLDG.PD.VPSTKNALVW
LOC_Os02g41710  ELV............IQPCKIA.....................RRYLAG..TFWFDLVTALPLPQFVIWIVIPKLKE.SA.TANRKNILRF
LOC_Os12g06570  ELV............RDPDQIA.....................IRYLKN..DFIIDLAAMLPIPQVIIWFVIPAVNN.SS.ANHTNNTLSM
LOC_Os09g38580  ELV............LDLKKIQ.....................RRYLRT..DFFIDLLATIPLPQVTVWIIMPSIKN.SD.YNIRNTTFAL
LOC_Os02g54760  DLI............TDPKEIA.....................WQYLRS..DFVVDAVAALPLPQILIWFVIPAIKY.ST.DEHNNNILVL
LOC_Os06g08850  DLV............TDPGNIA.....................KHYLRS..SFVVDLVASLPLPQIIIWSVIPSVKY.SL.SEHDDDILLL
LOC_Os02g53340  DLV............DEPKKIA.....................VRYLRG..YFLLDFFVVLPLPQVMILLVIPKYVGLST.ANYAKNLLRI
LOC_Os06g10580  DLV............DEPMKIA.....................MRYLRG..FFVLDLFVVLPLPQVMILLVIPKYVGLSS.ANYAKNLLRA
LOC_Os03g55100  KLV............WDPRAIA.....................AHYARSLKGLWFDLFVILPIPQVIFWLVIPKLIREEQ.IKLIMTMLLL
LOC_Os01g57370  EEVAAERGAGGNGGGPAPAQVA.....................RPVSRK..GLMLDMFVILPVMQVIVWVAAPAMIRAGS.TTAVMTVLLV
LOC_Os05g42250  PDE............EQPEAEAAAPAPAADADAAASNKLRDHGRY.RK..WLVLDFFVILPVMQVVVWVAAPAMIRAGS.TTAVMTVMLV
GRMZM2G005791   DLV............TNPKEIA.....................WKYIRS..DLAVDVAAALPLPQIIVWFVIPAIKY.SS.AEHNNNILVL
GRMZM2G023037   ELV............IDPMLIA.....................KRYIKS..YFAMDFVALLPLPQIVVWRYLHIPDG.PD.VLTTKTALVW
GRMZM2G066269   ...........................................................................................
GRMZM2G068904   ELV............IQPYEIA.....................RRYLGR..TFWFDLVTALPLPQFVIWIVIPRLNEYSR.TANTKNILRF
GRMZM2G074317   KLV............WDARAVA.....................AHYARSVKGLCFDLFVILPIPQVIFWLVIPKLIREEQ.VKLIMTILLL
GRMZM2G077828   ELV............IDTALIA.....................RRYMRR..FFSADLMSVLPLPQVVIWKFLHRSKG.TA.VLDTKNSLLF
GRMZM2G090528   ...........................................................ISVRSQVVVWVATPAMIRAGS.TTDVMIVLLT
GRMZM2G129375   ...........................................................................................
GRMZM2G135651   ELV............VDIKKIQ.....................RRYIRT..DFFVDILAAVPLPQVTVWLIMPAIKS.SD.YNIRNTTFAL
GRMZM2G141642   ...................................................................ILLVVPKVGL.SA.ANYAKNLLRV
GRMZM2G148118   ALV............KDTFAIA.....................KRYLST..LFLVDFLAVLPLPQVFVLVVLPKLQG.PE.IMKAKIVLLV
GRMZM5G858887   KLV............WDARAIA.....................AHYARSVKGLCFDLFVILPIPQVIFWLVIPKLIREER.VRLIMTILLL
consensus>70    elv.............d...ia.......................y......f..d....lp.pqv.i....p...............l..
```

```
                230       240       250       260       270       280       290       300
AT5G53130       IVFFQYIPRFIRIYPLYKEVT..RTSGILTETAWAGAAFNLFLYMLASHVFGAFWYLFSIERETVCWKQACERN...N......PP..CI
AT3G48010       IVLIQYVPRFLVMLPLNRRII..KATGVAAKTAWSGAAYNLILYLLVSHVLGSVWYVLSIQRQHECWRRECIKE...MNAT.HSPS..CS
AT2G23980       IVLVQYIPRFLRMYPLSSELK..RTAGVFAETAWAGAAYYLLLYMLASHIVGALWYLLALERNNDCWSKACHNN...Q.......N..CT
AT2G24610       IVLVQYIPRLYLIFPLSAEII..KATGVVTTTAWAGAAYNLLQYMLASHILGSAWYLLSIERQATCWKAECHKE...SV....PLQ..CV
AT5G54250       TFLFQYLPKIYHSIRHLRRNA..TLSGYIFGTVWWGIALNMIAYFVAAHAAGACWYLLGVQRSAKCLKEQCENT..........IG..CD
AT2G28260       IIIFQYVPRMFLIFPLSRQII..KATGVVTETAWAGAAYNLMLYMLASHVLGACWYLLAVERQEACWRHACNIE...K......QI..CQ
AT5G15410       IFLFQFLPKIYHCICLMRRMQ..KVTGYIFGTIWWGFALNLIAYFIASHVAGGCWYVLAIQRVASCIRQQCMRT..........GN..CN
AT1G15990       IVIVQYIPRFVRFIPLTSELK..KTAGAFAEGAWAGAAYYLLWYMLASHITGAFWYMLSVERNDTCWRFACKVQ...P.......DPRLC
AT4G30560       IILVQYIPRFIRLYPLSSELK..RTAGVFAETAWAGAAYYLLLYMLASHIVGAIWYLLALERYNGCWTKVCSNSS..L.......D..CH
AT5G57940       IVLVQYIPRFLRVLPLTSELK..RTAGVFAETAWAGAAYYLLLYMLASHIVGAFWYLLALERNDACWQEACIDA...G.......N..CS
AT3G17700       AVLFQYIPKLYRLLPFLA.GQ..TPTGFIFESAWANFVINLLTFMLAGHVVGSCWYLFGLQRVNQCLRNACGNF...GR......E..C.
AT1G01340       SIIAQYVPRILRMYPLYTEVT..RTSGIVTETAWAGAAWNLSLYMLASHVFGALWYLISVEREDRCWQEACEKT...K.......G..CN
AT1G19780       IVITQYIPRFVRFIPLTSELK..KTAGAFAEGAWAGAAYYLLWYMLASHITGAFWYMLSVERNDTCLRSACKVQ...P.......DPKVC
AT2G46450       IILSQYVPRIIRMYPLYKEVT..RAFGTVAESKWAGAALNLFLYMLHSYVFGAFWYLSSIERKSKCWRAACART...S.......D..CN
AT4G30360       IVLLQYIPRFYLIIPLSSQIV..KATGVVTKTAWAGAAYNLLLYMLASHVLGAAWYILSVDRYTSCWKSRCNGE...AG....QVN..CQ
AT5G14870       IVLVQYIPRSFIIFPLNQRII..KTTGFIAKTAWAGAAYNLLLYILASHVLGAMWYLSSIGRQFSCWSNVCKKD...NAL..RVLD..CL
AT4G01010       VIFTQYIPRILRIYPLYTEVT..RTSGIVTETAWAGAAWNLSLYMLASHVFGALWYLISVEREDRCWREACEKI...P......EV..CN
AT3G17690       TVLFQYIPKLYRLLPLLA.GQ..TSTGFIFESAWANFVINLLTFMLAGHAVGSCWYLSALQRVKKCMLNAWNIS...AD......E..R.
AT2G46430       VIFCQYVPRIARIYPLFKEVT..RTSGLVTETAWAGAALNLFLYMLASHVFGSFWYLISIERKDRCWREACAKI...Q.......N..CT
AT2G46440       IILSQYVPRIIRMYPLYKEVT..RAFGTVAESKRVGAALNFFLYMLHSYVCGAFWYLSSIERKSTCWRAACART...S.......D..CN
LOC_Os06g33570  IVICQYVPRLIRIRPLYLQIT..RSAGVITETPWAGAVLNLLIYLLASHVLGALWYLLSIERKDACWRDMCSNN...S......TV..CN
LOC_Os06g33610  ..........................................................................................
LOC_Os03g44440  IVFLQYIPRVVRIYPLSSELK..RTSGAFAESAYAGAAYYLLWYMLASHIVGASWYLLSIERVSDCWKKACNEF...P.......G..CN
LOC_Os12g28260  IVLVQYIPRLVRFYPITSELK..RTTGVFAETAFAGAAYYLLLYMLASHMVGAFWYLLSIERLDDCWRENCRVL...KF.....HQ..C.
LOC_Os04g55080  VVLFQYIPRLLRIFPVTKDLK..RTAGVFIETAWLGAAYYLLWFMLAGHNVGTLWYFLTIEREDSCWRSNCHSN...D.......G..CN
LOC_Os02g41710  SIIFQYLPRLFQIFPLSRQIV..MATGVMTETAWAGAAYNLILYMLASHVLGALWYLFSVQRQEACWREACHVE...G......PS..CQ
LOC_Os12g06570  IVLIQYIPRVFLIVSLNSKIV..KSSGVVTRTAWAGAAYNLLLYTLASHVLGALWYLLSIERQYTCWMDVCTRE...NGTNPAIPK..CY
LOC_Os09g38580  VIMIQYIFRMYLIVPLSNQII..KAAGVVAKSAWLGAAYNLLYYMLASHITGAIYYLLSIERQITCWNQQCLNE...........S..CS
LOC_Os02g54760  IVLAQYFPRLYLIFPLTYEIV..KTTGVVAKTAWQGAAYNMLLYMIASHVLGALWYLLSVDRQTACWKSNCKNE...T.......G..CD
LOC_Os06g08850  IALFQYVLRLYLVFSLNSKIV..EVTGAFSKTAWQGAAYNLLLYMIASHVLGALWYLLSVDRQTACWEKYCSKE...A.......G..CQ
LOC_Os02g53340  TVLLQYVPRIIRFVPLLGGQSDSSANGFIFESAWANFVINLLMFVLAGHVVGSCWYLFGLQRVNQCLRNACSAS...KI.....PS..C.
LOC_Os06g10580  TVLLQYVPRIIRFVPLLG.GQ..STNGFIFESAWSTFVINLLMFVLAGHVVGSCWYLFGLQRVNQCLRDSCAAS...NIS...KAL..C.
LOC_Os03g55100  LFLLQFLPKVYHSIYIMRKMQ..KVTGYIFGTIWWGFGLNLFAYFIASHIAGGCWYVLAIQRVASCLQEECKIK..........NT..CN
LOC_Os01g57370  SFLFEYLPKIYHAVRLLRRMQ..N..TYVFGTIWWGIALNLMAYFVAAHAVGACWYLLGAQRATKCLKEQCAQG...G......SG..CA
LOC_Os05g42250  AFMLEYLPKIYHSVVFLRRMQ..NQSGHIFGTIWWGIALNLIAYFVAAHAVGACWYLLGVQRATKCLKEQCLLA...GL.....PA..CA
GRMZM2G005791   IVLAQYLPRLYLIFPLTYEIV..KATGVVAKTAWEGAAYNMVLYLIASHVLGALWYLLSVDRQTFCWKTNCLNE...T.......G..CD
GRMZM2G023037   VVLIQYIPRLLRIFPVITDLK..RTAGVFIETAWAGAAYYLLWFMLAGHNVGTLWYFLTIEREDDCWHLYCDDPNFGL.......G..CN
GRMZM2G066269   ..........................................................................................
GRMZM2G068904   SIIFQYLPRLFQIFPLSGRIV..MATGVMTETAWAGAAYNLILYMLASHVLGALWYLFSVQRQEACWREACLLV...S.......PT.SQ
GRMZM2G074317   MFIFQFLPKVYHSIHIMRKMQ..KVTGYIFGSIWWGFGLNLFAYFIASHIAGGCWYVLAIQRIASCLQEECKRN..........NS..CD
GRMZM2G077828   IVFIQYVPRVVRIYPISSELK..RTSGVFAETAYAGAAYYLLWYMLASHIVGAFWYLLSIERVSDCWRNACDEF...P.......G..CN
GRMZM2G090528   AFLLEYLPKIYHAVRVLRRMQ..GVSGYLFGTIWWGIALNLMAYFVAAHAVGACWYLLGAQRATKCLREQCAQA...G......SG..CA
GRMZM2G129375   ..........................................................................................
GRMZM2G135651   VIVIQYVIRMYLIIPLSNQII..KAVGVVAKSAWGGAAYNLLLYMLASHITGAIYYLLSIERQITCWDQQCVAE...YN....DTH..CN
GRMZM2G141642   TVLLQYVPRIIRFVPLLD.GQ..STNGFIFESAWANFVINLLMFILAGHVVGSCWYLFGLQRVNQCLRDACSIS...TI.....PY..C.
GRMZM2G148118   IIICQYVPRLLRIIPLYLQIT..RSAGILTETAWAGAAFNLIIYMLASHGFGALWYILSIQREDTCWRQACINQ..........TG..CD
GRMZM5G858887   MFIFQFLPKVYHSIHIMRKMQ..KVTGYIFGSIWWGFGLNLFAYFIASHIAGGCWYVLAIQRIASCLQEECKKN..........NS..CD
consensus>70    .v..qy.pr.....pl..........g.....aw.g.a.nl..y.lashv.g..wyl..ier...c....c.................c.

                         310                 320                                330       340
AT5G53130       S..K..LLYCDPETA.........GG.NAFLNE...............S.CP.....I..QT...PNTTLFDFGIFLDALQSGV..VES
AT3G48010       L..L..FLDCGSLHD.........PGRQAWMRI...............T.RV.LSNCDA..RN...DDDQHFQFGMFGDAFTNDV...TS
AT2G23980       R..N..FLFCGNQNM.........KGYAAWDNI...............KVSYLQLKCPVNVPE...DEEPPFDFGIYLRALSSGI..VSS
AT2G24610       T..D..FFDCGTLHR.........DDRNNWQNT...............T.VV.FSNCDP..S.....NNIQFTFGIFADALTKNV...VS
AT5G54250       L..R..MLSCKEPVYYGTTVMVLDRARLAWAQN...............H.QA.RSVCL........DINTNYTYGAYQWTIQL.V...SS
AT2G28260       Y..R..FFECRRLED.........PQRNSWFEW...............S.NI.TTIC.....K...PASKFYEFGIFGDAVTSTV...TS
AT5G15410       L.....SLACKEEVCY..........QFVSPTSTVGYPCLSGNLTS..V.VN.KPMCL........DSNGPFRYGIYRWALPV.I...SS
AT1G15990       V..Q..ILYCGTKFV........SSGETEWIKT...............VPELLKSNCSA...K...ADDSKFNYGIYGQAISSGI..VSS
AT4G30560       R..N..FLFCGNEKM.........DGYAAWTTI...............KDSVLQLNCPV..NT...TDNPPFDFGIYLRALSSGI..VSS
AT5G57940       T..D..FLYCGNQNM.........DGYAVWNRA...............KESVLKSKCRAD.LD...DNNPPFDFGIYTQALSSGI..VSS
AT3G17700       Q..D..LIDCGNGNS.S......VLVRATWKDN...............A.S..ANACF.........QEDGFPYGIYLKAVNL.T...NH
AT1G01340       M..K..FLYCENDRN.........VS.NNFLTT...............S.CPFLDP..G..DI...TNSTIFNFGIFTDALKSGV..VES
AT1G19780       V..Q..ILYCGSKLM........SSRETDWIKS...............VPDLFKNNCSA...K...SDESKFNYGIYSQAVSSGI..VSS
AT2G46450       L..TVTDLLCKRAGS.........DN.IRFLNT...............S.CPLIDP..A..QI...TNSTDFDFGMYIDALKSGVLEVKP
AT4G30360       L..Y..YLDCDSMYD.........NNQMTWANV...............T.KV.FKLCDA........RNGEFKYGIFGNAITKNV...VS
AT5G14870       P..S..FLDCKSLEQ.........PERQYWQNV...............T.QV.LSHCDA..T....SSTTNFKFGMFAEAFTTQV...AT
AT4G01010       F..R..FLYCDGNSS.........VR.NDFLTT...............S.CPFINP..D..DI...TNSTVFNFGIFTDALKSGI..VES
AT3G17690       R..N..LIDCARGSYAS......KSQRDLWRDN...............A.S..VNACF.........QENGYTYGIYLKAVNL.T...NE
AT2G46430       H..A..YLYCSPTGE.........DN.RLFLNG...............S.CPLIDP..E..EI...TNSTVFNFGIFADALQSGV..VES
AT2G46440       L..TVTDLLCKRAGS.........DN.IRFLNT...............S.CPLIDP..A..QI...TNSTDFDFGMYIDALKSGVLEVKP
LOC_Os06g33570  Q..A..YLYCGDKE.............NSILRT...............A.CL......P..ID...SNDIDPNFGIYVPALNN.V..SQS
LOC_Os06g33610  ..........................................................................................
LOC_Os03g44440  K..I..YMYCGND.........HQKGFLEWRTI...............TRQYINETCEP..R....DGVMPFNYGIYTPAVRSDV..IKS
LOC_Os12g28260  K..K..YMYCGGGNL.......GQSGFLEWRTM...............IRQVLVMECAP..AD...EAGTGFQYGIFTTAIQSGV..VST
LOC_Os04g55080  K..S..YLYCSDNHT.........GNYTSWLSK................RTELLSAC..........STNSFQFGIFEQALVSGI..LRP
LOC_Os02g41710  T..L..FFDCKTVS..........SNRTMWYEL...............S.NI.TSLC.....T...PSNGFYQFGIYGEALDNGL...TS
LOC_Os12g06570  M..S..YLDCKTLED.........PIRMDWHSR...............S.EI.DHQCLL........PEATYVYGLFADALNLDV...AK
LOC_Os09g38580  F..N..FISCDNTGS.........SSYLTWGKN...............T.SI.FDNCDP..NRNSSANPPPFNYGMFSTALSKGA...VS
LOC_Os02g54760  I..K..FLDCDVIPN............QNWASK...............T.AI.FNTCDA..T....NTSISFDYGMFQPALFNQA...PG
LOC_Os06g08850  N..R..YLACDIQSD............SNWKIS...............T.AI.FNKCDA..T....NKTIDFDFGMFTPLLSNQA...PD
LOC_Os02g53340  D..G..FIDCGRGINIG...KQNQLSRQQWFND...............S.A..STACFD.......TGDNGFHYGIYEQAVLL.T...TE
LOC_Os06g10580  N..N..CTDCGITG..........INRTNWLNN...............S.D..LTGCFD.......TKSGNFPYGIYQQAVLL.....TT
LOC_Os03g55100  L..T..SLACSKEMCF..........HLPWSDKNG.LAC...NLTSFGQ.QN.IPDCL........SGNGPFAYGIYKGALPV.I...SS
LOC_Os01g57370  P..G..ALACAAPLYYGGAVGGVGADRLAWALD...............A.SA.RGTCL........DSGDNYQYGAYKWTVML.V...AN
LOC_Os05g42250  SSTA..AVACVDPLYYGAAVASVGGDRLAWGGN...............A.TA.RNVCL........SSGDNYQYGAYKWTVML.V...SN
GRMZM2G005791   L..K..YLDCDTTPN............ATWANT...............T.AV.FSNCNA..S....DTNISFDFGMFEPALSNQA...PA
GRMZM2G023037   S..S..YLYCNNHHH.........GSYDSWLTN...............NSAQVFNMCNG.......GQDNPFNFGIYEQALVSKI..LSP
GRMZM2G066269   ..........................................................................................
GRMZM2G068904   T..M..FFDCKALS..........SNRTIWYEL...............S.NITTSRC.....T...PGNGFYPFGIYEEALYAKL...TS
GRMZM2G074317   L..I..SLACSKEICF..........HPPWSSNVNGFACD.TNMTSFSQ.QN.VSTCL........SGKGSFAYGIYLGALPV.I...SS
GRMZM2G077828   Q..I..YMYCGND.........RQLGFLEWRTI...............TRQVINETCEP..KR...DGSIPFNYGIYSPAVVSDV..LKS
GRMZM2G090528   P..W..ALACAEPLYYGATASSVGAARLAWAGN...............A.TA.RGTCL........DSADNYQYGAYQWTVML.V...AN
GRMZM2G129375   ..........................................................................................
GRMZM2G135651   F..S..FISCENNGS.........NDYSVWANK...............T.KV.FANCDA..T....NSSISFNYGMFSSALSKGA...VS
GRMZM2G141642   D..S..FIDCGRGIGSG.......LYRQQWFND...............S.G..AEACFN..T....GNDATFQYGIYEQAVLL.T...TE
GRMZM2G148118   P..T..SLYCGYHSL.........AN.NSFLQN...............A.CP......T..NS...TANPDPIFGIFLPALQN.V..SQS
GRMZM5G858887   L..I..SLACSKEICF..........HPPWSSNVNGFACD.TNMTSFSQ.RN.VSTCL........SGKGSFAYGIYLGALPV.I...SS
consensus>70    .........c...................w..........................c..............f.yg.y..a..........
```

```
                    350       360       370       380       390       400       410       420       430
AT5G53130      QDFPQKFFYCFWWGLQNLSSLGQNLKTSTYIWEICFAVFISIAGLVLFSFLIGNMQTYLQSTTTRLEEMRVKRRDAEQWMSHRLLPENLR
AT3G48010      SPFFDKYFYCLWWGLRNLSSYGQSLAASTLSSETIFSCFICVAGLVFFSHLIGNVQNYLQSTTARLDEWRVRRRDTEEWMRHRQLPDELQ
AT2G23980      KNFVSKYFFCLWWGLQNLSTLGQGLETSTYPGEVIFSITLAIAGLLLFALLIGNMQTYLQSLTIRLEEMRVKRRDSEQWMHHRMLPPELR
AT2G24610      SPFLEKYLYCLWFGLQNLSSYGQNLSTSTSVLETMFAILVAIFGLVLFALLIGNMQTYLQSITVRLEEWRLKRRDTEEWMGHRLLPQNLR
AT5G54250      ESRLEKILFPIFWGLMTLSTFGN.LESTTEWSEVVFNIIVLTSGLLLVTMLIGNIKVFLHATTSKKQAMHLKMRNIEWWMKKRHLPIGFR
AT2G28260      SKFINKYFYCLWWGLKNLSSLGQNLATSTYAGEILFAIIIATLGLVLFALLIGNMQTYLQSTTMRLEEWRIRRTDTEQWMHHRQLPPELR
AT5G15410      NSLAVKILYPIFWGLMTLSTFANDLEPTSNWLEVIFSIVMVLSGLLLFTLLIGNIQVFLHAVMAKKRKMQIRCRDMEWWMKRRQLPSRLR
AT1G15990      TTFFSKFCYCLWWGLQNLSTLGQGLQTSTFPGEVLFSIAIAIAGLLLFALLIGNMQTYLQSLTVRLEEMRIKRRDSEQWMHHRSLPQNLR
AT4G30560      KSFVSKYFFCLWWGLQNLSTLGQGLETSTYPGEVIFSIALAIAGLLLFALLIGNMQTYLQSLTIRLEEMRVKRRDSEQWMHHRMLPPELR
AT5G57940      QNFIVKYCYCLWWGLQNLSTLGQGLETSTYPMEIIFSISLAISGLILFALLIGNMQTYLQSLTIRLEEMRVKRRDSEQWMHHRMLPQDLR
AT3G17700      SNLFTRYSYSLFWGFQQISTLAGNQVPSYFLGEVFFTMGIIGLGLLLFALLIGNMQNFLQALGKRNLEMTLRRRDVEQWMSHRRLPDGIR
AT1G01340      HDFWKKFFYCFWWGLRNLSALGQNLQTSKFVGEIIFAISICISGLVLFALLIGNMQKYLESTTVREEEMRVRKRDAEQWMSHRMLPEDLR
AT1G19780      TTFFSKFCYCLWWGLQNLSTLGQGLQTSTYPGEVLFSIAIAVAGLLLFALLIGNMQTYLQSLTVRLEEMRIKRRDSEQWMHHRSLPQNLR
AT2G46450      KDFPRKFVYCFWWGLRNISALGQNLETSNSAGEIFFAIIICVSGLLLFAVLIGNVQKYLQSSTTRVDEMEEKRRDTEKWMSYRVIPEYLK
AT4G30360      SQFFERYFYCLWWGLQQLSSYGQNLSTTMFMGETTFAVLIAIFGLVLFAHLIGNMQTYLQSLTVRLEEWRLKKRDTEEWMRHRQLPEELR
AT5G14870      TDFVSKYLYCLWWGLRNLSSYGQNITTSVYLGETLFCITICIFGLILFTLLIGNMQSSLQSMSVRVEEWRVKRRDTEEWMRHRQLPPELQ
AT4G01010      DDFWKKFFYCFWWGLRNLSALGQNLNTSKFVGEIIFAVSICISGLVLFALLIGNMQKYLESTTVREEEMRVRKRDAEQWMSHRMLPDDLR
AT3G17690      SSFFTRFSYSLYWGFQQISTLAGNLSPSYSVGEVFFTMGIIGLGLLLFARLIGNMHNFLQSLDRRRMEMMLRKRDVEQWMSHRRLPEDIR
AT2G46430      RDFPKKFFYCFWWGLRNLSALGQNLKTSAFEGEIIFAIVICISGLVLFALLIGNMQKYLQSTTVRVEEMRVKRRDAEQWMSHRMLPDDLR
AT2G46440      KDFPRKFVYCFWWGLRNISALGQNLETSNSAGEIFFAIIICVSGLLLFAVLIGNVQKYLQSSTTRVDEMEEKRRDTEKWMSYREIPEYLK
LOC_Os06g33570 TNFLAKLFYCVWWGLQNLSSLGQNLKTSTYAWENLFAVFVSISGLVLFALLIGNVQTYLQSAHLREEEMRVKSRDTDQWMSYRLLPENLK
LOC_Os06g33610 ....................................................................MRVKSRDTDQWMSYRLLPENLK
LOC_Os03g44440 NDFTSKLLYCLWWGLANLSTLGQGLQTSIYTGEALFSIFLATFGLILMAMLIGNIQTYLQSMTVRLEEMRVKRRDSEQWMHHRLLPQELR
LOC_Os12g28260 TNLVAKVLFCLWWGLQNLSTVGQGLKTTHYKGEALFAIFLAVFGLILMALLIGNMQTYLQSMTLRLEEMRLRRRDSEQWMRHRVLPVDLQ
LOC_Os04g55080 GNFISKICYCFWWGLQNLSTLGQGLQTSIYPGEVLFSIAICVIGLILFALLIGNMQTYLQSVAIRLEEMRVKKRDAEQWMHHRSLPPQIR
LOC_Os02g41710 SSFTQKYFYCFWWGLKNLSCLGQNLSTSLFIGEITFATVIGVLGLVLFALLIGNMQ....ATMVRLEEWRTKRTDMERWMNHRQIPQPLK
LOC_Os12g06570 VNFWDKYLYCLWWGFRNLSSYGQNLENSTYRGETIFCILICIMGLVFFSHLIGNMQTYLQSMTVRLEEWRVKRRDIEEWMRHRQLPLELQ
LOC_Os09g38580 APFLEKYFFCLWWGLLQLSSSGNPLQTSAYIAENTFAIAIGALSLVLFAQLIGNMQTYLQSISKRLEEWRLRQRDMEEWMRHHQLPDELQ
LOC_Os02g54760 QRFLMKYFYSLWWGLQNLSCYGQTITVSTYIGETLYCIFLAVLGLVLFAHLIGNVQTYLQSITVRVEEWRLKQRDTEEWMRHRQLPHELR
LOC_Os06g08850 QGFLKKFFYCLWWGLQNLSCYGQTLTVSTYIGETLYAIFLAVLGLVLFAHLIGNVQTYLQSITARVEEWRIKQRDTEEWMRHRQLPQKLR
LOC_Os02g53340 DNAVKRYIYSLFWGFQQISTLAGNLVPSYFAWEVLFTMAIIGLGLLLFALLIGNMQNFLQALGRRRLEMQLRRRDVEQWMSHRRLPEDLR
LOC_Os06g10580 EPGLKRYIYSLFWGFQQISTLAGNLIPSYFVWEVIFTMAIIGLGLLLFALLIGSMQNFLQALGKRRLEMQLRRRDVEQWMSHRRLPEDLR
LOC_Os03g55100 NSLAVKILYPIFWGLMTLSTFGNDLEPTSNWLEVIFSIINVLSGLMLFTLLIGNIQVFLHAVLARKRKMQLRFRDMEWWMRRRQLPSRLR
LOC_Os01g57370 PSRLEKILLPIFWGLMTLSTFGN.LASTTEWLEIVFNIITITGGLILVTMLIGNIKVFLNAATSKKQAMQTRLRGVEWWMKRKKLPQSFR
LOC_Os05g42250 PSRLEKMLLPIFWGLMTLSTFGN.LESTTEWVEIVFNIMTITGGLILVTMLIGNIKVFLNATTSKKQAMQTRLRGLEWWMEHKGVPHGFR
GRMZM2G005791  QSFAMKYFYSLWWGLQNLSCYGQTLTVSTYLGETLYCIFLAVLGLVLFAHLIGNVQTYLQSITVRVEEWRLKQRDTEEWMRHRQLPCELR
GRMZM2G023037  GNFISKLCYCFWWGLQNLSTLGQGLLTSTYPGEVLFSIAICVLGLILFALLIGNMQSYLQSVAIRLEEMRVKKRDAEQWMHHRSLPLDIR
GRMZM2G066269  ............................................FLFLGWIELLLQTYLQSASGHIEEMRVRRRDMEQWMSYRLLPEHIK
GRMZM2G068904  SSFTQKYFYCFWWGLKNLSSLGQNLSTSLFIGEITFAIVVGVLGLVLFGLLIGNMQSYLQATMVRLEEWRTKRTDMERWMHHRQIPQPLK
GRMZM2G074317  NSLAVKILYPIFWGLMTLSTFGNDLAPTSNGIEVIFSIINVLSGLMLFTLLIGNIQVFLHAVLARKRKMQLRFRDMEWWMRRRQLPSRLR
GRMZM2G077828  KDTTSKLLFCLWWGLANLSTLGQGLKTSIYTGEALFSIALAIFGLILMAMLIGNIQTYLQSLTVRLEEMRVKQRDSEQWMHHRLLPPELR
GRMZM2G090528  PSRVERVLLPIFWGLMTLSTFGN.LESTTEWLEIVFNIVTITGGLILVTMLIGNIKVFLNATTSKKQAMHTRLRSVELWMKRKDLPRSYR
GRMZM2G129375  ..........................................VCFLLLTMFHPYLQTYLQSASLRVEEMRVKSRDTDQWMSYRHLPENLK
GRMZM2G135651  SPFLEKYFFCLWWGLLQLSSSGNPLVTSAFITENAFAIAIGAISLILFAQLIGKMQTYLQSISKRLEEWRLRQRDMDEWMRHHQLPSHLQ
GRMZM2G141642  DSAVKRYIYSLFWGFQQISTLAGNLVPSYFIWEVLFTMAIIGLGLLLFALLIGNMQNFLQALGRRRLEMQLRRRDVEKWMSHRRLPEDLR
GRMZM2G148118  TSFFEKLFYCFWWGLQNLSSLGQNMKTSTNTLENLFAVFVSTSGLVLFALLIGNVQTYLQSASVRIEEMRVKRRDTEQWMAHRLLPENLK
GRMZM5G858887  NSLAVKILYPIFWGLMTLSTFGNDLAPTSNGIEVIFSIINVLSGLMLFTLLIGNIQVFLHAVLARKRKMQLRFRDMEWWMRRRQLPSRLR
consensus>70   .....k..y...wgl.nls..gq.l..s....e..f.i.....gl.lf.llign.q.ylq....r.eem....rd.#qWM.hr.lP..l.
```

```
                    440       450       460       470       480       490       500       510       520
AT5G53130      KRIRRYEQYKWQETRGVDEENLLSNLPKDLRRDIKRHLCLALLMRVPMFEKMDE.QLLDALCDRLQPVLYTEESYIVREGDPVDEMLFIM
AT3G48010      ERVRRFVQYKWLTTRGVDEEAILRALPLDLRRQIQRHLCLALVRRVPFFAQMDD.QLLDAICERLVPSLNTKDTYVIREGDPVNEMLFII
AT2G23980      ERVRRYDQYKWLETRGVDEENLVQNLPKDLRRDIKRHLCLALVRRVPLFENMDE.RLLDAICERLKPCLFTEKSYLVREGDPVNEMLFII
AT2G24610      ERVRRFVQYKWLATRGVDEETILHSLPADLRRDIQRHLCLDLVRRVPLFAQMDD.QLLDAICERLASSLSTQGNYIVREGDPVTEMLFII
AT5G54250      QRVRNYERQRWAAMRGVDECEMVQNLPEGLRRDIKYHLCLDLVRQVPLFQHMDD.LVLENICDRVKSLIFTKGETIQKEGDAVQRMLFVV
AT2G28260      QAVRKYDQYKWLATRGVDEEALLISLPLDLRRDIKRHLCFDLVRRVPLFDQMDE.RMLDAICERLKPALCTEGTFLVREGDPVNEMLFII
AT5G15410      QRVRRFERQRWNALGGEDELELIHDLPPGLRRDIKRYLCFDLINKVPLFRGMDD.LILDNICDRAKPRVFSKDEKIIREGDPVQRMIFIM
AT1G15990      ERVRRYDQYKWLETRGVDEENIVQSLPKDLRRDIKRHLCLNLVRRVPLFANMDE.RLLDAICERLKPSLFTESTYIVREGDPVNEMMFII
AT4G30560      ERVRRYDQYKWLETRGVDEENLVQNLPKDLRRDIKRHLCLALVRRVPLFENMDE.RLLDAICERLKPCLYTESSYLVREGDPVNEMLFII
AT5G57940      ERVRRYDQYKWLETRGVDEEYLVQNLPKDLRRDIKRHLCLALVRRVPLFKSMDD.KLLDAICMRLKPCLFTESTYLVREGDPVDEMLFII
AT3G17700      RRVREAERFNWAATRGVNEELLFENMPDDLQRDIRRHL.FKFLKKVRIFSLMDE.PILDAIRERLKQRTYIGSSTVLHRGGLVEKMVFIV
AT1G01340      KRIRRYEQYRWQETRGVEEETLLRNLPKDLRRDIKRHLCLDLLKKVPLFEIMDE.QLLDAVCDRLRPVLYTENSYVIREGDPVGEMLFVM
AT1G19780      ERVRRYDQYKWLETRGVDEENIVQSLPKDLRRDIKRHLCLNLVRRVPLFANMDE.RLLDAICERLKPSLYTESTYIVREGDPVNEMLFII
AT2G46450      ERIRRFEDYKWRETKGTEEEALLRSLPKDLRLETKRYLYLDMLKRVPWLNIMDDGWLLEAVCDRVKSVFYLANSFIVREGHPVEEMLIVT
AT4G30360      NRVRRYEQYKWLATRGVDEEVLLQSLPTDLRRDIQRHLCLDLVRRVPFFSQMDD.QLLDAICERLVSSLCTEGTYLVREGDLISEMLFII
AT5G14870      ERVRRFVQYKWLATRGVDEESILHSLPTDLRREIQRHLCLSLVRRVPFFSQMDD.QLLDAICGCLVSSLSTAGTYIFREGDPVNEMLFVI
AT4G01010      KRIRRYEQYKWQETRGVEEENLLRNLPKDLRRDIKRHFCLDLLKKVPLFEIMDE.QLLDAVCDKLKPVLYTENSYAIREGDPVEEMLFVM
AT3G17690      KRVREVERYTWAATRGVNEELLFENMPDDLQRDIRRHL.FKFLKKVRIFSLMDE.SVLDSIRERLKQRTYIRSSTVLHRGGLVEKMVFIV
AT2G46430      KRIRKYEQYKWQETKGVEEEALLSSLPKDLRKDIKRHLCLKLLKKVPWFQAMDD.RLLDALCARLKTVLYTEKSYIVREGEPVEDMLFIM
AT2G46440      ERIRRFEDYKWRRTKGTEEEALLRSLPKDLRLETKRYLFLKLLKKVPLLQAMDD.QLLDALCARLKTVHYTEKSYIVREGEPVEDMLFIM
LOC_Os06g33570 ERIRRHEKYRWHQTSGVDEELLLMNLPKDLRRAIKRHLCLSLLMRVPMFENMDD.QLLNALCDRLKPVLYTEGSCIIREEDPVNEMLFIM
LOC_Os06g33610 ERIRRHEKYRWHQTSGVDEELLLMNLPKDLRRAIKRHLCLSLLMRVPMFENMDD.PLLDALCDHLKPVLYTEGSCIIREEDPVYEMLFIM
LOC_Os03g44440 ERVRRYDAYKWVNTRGVDEEVLVANLPKDLRRDIKRHLCLGLVRRVPLFANMDE.RLLDAICERLRPALYTERTFIIREGDPVDQMLFII
LOC_Os12g28260 ERVWRHDQYRWLETRGVDEDSLVRSLPKDLRRDVKRHLCLRLVRRVPLFANMDE.RLLDAICERLKPSLCTEATYILREGDPVDEMLFII
LOC_Os04g55080 ERVRRYERYRWLETRGVDEENLVQTLPKDLRRDIKRHLCLGLVKRVPLFENMDE.RLLDAICERLRPTLYTENEYILREGDPVDEMHFIL
LOC_Os02g41710 QCVRRYHQYKWLATRGVDEEALLEDLPMDIRRDIKRHLCLDLVRRVPLFDEMDE.RMLEAICERLRPALYTRGTRLVRELDPVDSMLFII
LOC_Os12g06570 ERVRRFQYKWLATRGVDEESILQSLPLDLRREIQRHLCLALVRRVPFFSQMDE.QLLDAICERLVSSLSTKDAYIVREGDPVSEMLFVI
LOC_Os09g38580 DRVRRFVQVKWLATRGVEEESILQALPADIRRDVQRHLCLDLVRRVPFFSEMDY.QLLDAICERLVSFLCPERTYISREGDPVNEMLFVI
LOC_Os02g54760 ERVRRFIQYKWLATRGVNEESILQALPADLRRDIKRHLCLGLVRRVPFFSQMDN.QLLDAICERLVSSLCTQGTYIVREGDPVTEMLFII
LOC_Os06g08850 ERVRRFVHYKWLATRGVDEESILKALPADLRRDIKRHLCLDLVCRVPFFSQMDG.QLLDAICERLVSSLSTVGTYIVREGDPVTEMLFII
LOC_Os02g53340 RRVRRAERFTWAATQGVNEEELLSNLPEDIQRDIRRHF.FRFLNKVRLFTLMDW.PILDAICDKLRQNLYISGSDILYQGGPVEKMVFIV
LOC_Os06g10580 RRVRSAERFSWVATRGVNEEELLSNLPEDIQRGIRRHF.FGFLKKVRLFNLMDN.ATWDAICDKLRQNLYITGSDILYQGGPVEKMVFIV
LOC_Os03g55100 QRVRKYERERWAAITGDEEMEMIKDLPEGLRRDIKRYLCLELVKQVPLFHGMDD.LILDNICDRLRPLVFSSGEKVIREGDPVQRMVFVL
LOC_Os01g57370 HRVRQHERQRWAATRGVDECRIVRDLPEGLRRDIKYHLCLDLVRQVPLFQHMDD.LVLENICDRVKSLVFPKGEIIVREGDPVQRMLFIV
LOC_Os05g42250 QRVRQFERQRWAATRGVDECQIVRDLPEGLRRDIKYHLCLDLVRQVPLFHHMDD.LVLENICDRVKSLIFPKGEIIVREGDPVQRMLFIV
GRMZM2G005791  ERVRRFIQYKWLATRGVNEESILHALPADLRRDIKRHLCLGLVRRVPFFSQMDD.QLLDAICERLVSSLCTKGTYIVREGDPVTEMLFII
GRMZM2G023037  HRVRKYERYRWLETRGVDEETLVQTLPKDLRRDIKRHLCLGLVKRVPLFENMDE.RLLDAICERLRPALYTENEFILREGDPVDEMHFIL
GRMZM2G066269  ERILRHHQYRWQETQGVDEEGLLVNLPKDLRRDIKRHLCLSLLKRVPMFENMDD.QLLDAMCDRVKPMLYTEGSHIVREGDPVNEMFFIM
GRMZM2G068904  QCVRRYHQYQWVATRGVDEEALLQDLPMDIRRDIKRHLCLDLVRRVPLFDEMDE.RMLDAICERLRPALYTRGTRLMRELDPVDSMLFII
GRMZM2G074317  QRVRKYERERWAAVTGDEEMEMIKDLPEGLRRDIKRYLCLELVKQVPLFHGMDD.LILDNICDRLRPLVLSSGEKVIREGDPVQRMVFIL
GRMZM2G077828  ERVRRYDQYKWLNTHGVDEEALVQNLPKDLRRDIKRHLCLGLVRRVPLFANMDE.RLLDAICERLKPSLCTEHTYITREGDPVDQMVFII
GRMZM2G090528  HRVRQYERQRWAATRGVDECRIVRDLPEGLRRDIKYHLCLGLVRQVPLFQHMDD.LVLENICDRVKSLIFPKGEVIVREGDPVKRMLFIV
GRMZM2G129375  ERIRRYEQYRWQETSGVDEEQLLMNLPKDLRRDIKRHLCLKLLMRVPLFENMDE.QLLDAMCDCLKPILYTEGSCVIREGDPVNEMLFVM
GRMZM2G135651  ERVRRFVQVKWLATRGVEEESILQALPADIRRDVQRHLCLDLVRRVPFFSEMDN.QLLDAICERLVSFLCPENTYISREGDPVNEMLFII
GRMZM2G141642  RRVRRAERFTWAATQGVNEEELLSNLPEDIQRDIRRHF.FRFLNKVRLFTLMDW.PILDAICDKLRQNLYISGSDILYQGGTVEKMVFIV
GRMZM2G148118  DRIMRHEQYRWQETRGVDEEGLLKNLPKDLRREIKRHLCLSLLMKVPMFENMDE.QLLDAMCDRLKPMLYTEGSCIIREGDPVNEMLFIM
GRMZM5G858887  QRVRKYERERWAAVTGDEEMEMIKDLPEGLRRDIKRYLCLELVKQVPLFHGMDD.LILDNICDRLRPLVFSSGEKVIREGDPVQRMVFIL
consensus>70   er!rrye.y.W..trGv#Ee.l...$P.dlrrdikrhlcl.l...Vplf..MDe..ll#aicerl...ly.....ivregdp!n.Mlf!.
```

```
                 530       540       550       560       570                 580       590       600
AT5G53130        RGKLLTITTNGGRTGF.LNSEYLGAGDFCGEELLTWALDPHSSSNL..........PISTRTVRALMEVEAFALKADDLKFVASQFRR.L
AT3G48010        RGQMESSTTDGGRSGF.FNSITLRPGDFCGEELLTWALVPNINHNL..........PLSTRTVRTLSEVEAFALRAEDLKFVANQFRR.L
AT2G23980        RGRLESVTTDGGRSGF.YNRSLLKEGDFCGDELLTWALDPKSGSNL..........PSSTRTVKALTEVEAFALIADELKFVASQFRR.L
AT2G24610        RGKLESSTTNGGRTGF.FNSITLRPGDFCGEELLAWALLPKSTVNL..........PSSTRTVRALEEVEAFALQAGDLKFVANQFRR.L
AT5G54250        RGHLQSSQL..LRDGV.KSCCMLGPGNFSGDELLSWCLRRPFVERL..........PPSSSTLVTLETTEAFGLDAEDVKYVTQHFRYTF
AT2G28260        RGHLDSYTTNGGRTGF.FNSCLIGPGDFCGEELLTWALDPRPVVIL..........PSSTRTVKAICEVEAFALKAEDLQFVASQFRR.L
AT5G15410        RGRVKRIQS..LSKGV.LATSTLEPGGYLGDELLSWCLRRPFLDRL..........PPSSATFVCLENIEAFSLGSEDLRYITDHFRYKF
AT1G15990        RGRLESVTTDGGRSGF.FNRGLLKEGDFCGEELLTWALDPKAGSNL..........PSSTRTVKALTEVEAFALEAEELKFVASQFRR.L
AT4G30560        RGRLESVTTDGGRSGF.FNRSLLKEGDFCGEELLTWALDPKSGSNL..........PSSTRTAKALTEVEAFALIADELKFVASQFRR.L
AT5G57940        RGRLESVTTDGGRSGF.FNRSLLKEGEFCGEELLTWALDPKSGVNL..........PSSTRTVKALTEVEAFALTSEELKFVASQFRR.L
AT3G17700        RGEMESIGEDGSV.......LPLYEGDVCGEELLTWCLERSSVNPDGTRIRMPSKGLLSSRNVRCVTNVEAFSLSVADLEDVTSLFSRFL
AT1G01340        RGRLVSATTNGGRSGF.FNAVNLKASDFCGEDLLPWALDPQSSSHF..........PISTRTVQALTEVEAFALTAEDLKSVASQFRR.L
AT1G19780        RGRLESVTTDGGRSGF.FNRGLLKEGDFCGEELLTWALDPKAGSNL..........PSSTRTVKALTEVEAFALEAEELKFVASQFRR.L
AT2G46450        RGKLKSTTGSH.EMGVRNNCCDLQDGDICGELLFNGS.......RL..........PTSTRTVMTLTEVEGFILLPDDIKFIASHLNV.F
AT4G30360        RGRLESSTTNGGRTGF.FNSIILRPGDFCGEELLSWALLPKSTLNL..........PSSTRTVRALVEVEAFALRAEDLKFVANQFRR.L
AT5G14870        RGQIESSTTNGGRSGF.FNSTTLRPGDFCGEELLTWALMPNSTLNL..........PSSTRSVRALSEVEAFALSAEDLKFVAHQFKR.L
AT4G01010        RGKLMSATTNGGRTGF.FNAVYLKPSDFCGEDLLTWALDPQSSSHF..........PISTRTVQALTEVEAFALAADDLKLVASQFRR.L
AT3G17690        RGEMESIGEDGSV.......LPLSEGDVCGEELLTWCLSSINPD..GTRIKMPPKGLVSNRNVRCVTNVEAFSLSVADLEDVTSLFSRFL
AT2G46430        RGNLISTTTYGGRTGF.FNSVDLVAGDFCGD.LLTWALDPLS.SQF..........PISSRTVQALTEVEGFLLSADDLKFVATQYRR.L
AT2G46440        RGNLISTTTYGGRTGF.FNSVDLIAGDSCGD.LLTWALYSLS.SQF..........PISSRTVQALTEVEGFVISADDLKFVATQYRR.L
LOC_Os06g33570   RGNLMSMTTNGGRTGF.FNSDVLKGGDFCGEELLTWALDPTSVSSL..........PSSTRTVKTMSEVEAFALRAEDLKFVATQFRR.L
LOC_Os06g33610   RGNLMSMTTDGGITGF.FKSDVLKGGDFCGEELLTWALDPTSVSRL..........PSSTRTVETMSEVEAFALTAEDLKFVATQFRR.L
LOC_Os03g44440   RGCLESITTDGGRSGF.FNRSLLEESDFCGEELLTWALDPKAGLSL..........PSSTRTVRALSEVEAFALHSDELKFVAGQFRR.M
LOC_Os12g28260   RGRLESSTTDGGRMGF.FNRGLLKEGDFCGEELLTWALDPKAAANL..........PLSTRTVKAISEVEAFALHADELKFVAGQFRR.L
LOC_Os04g55080   HGCLESETTDGGRSGF.FNKVQLKEGAFCGDELLTWALDPKSAANF..........PASTRTVKALTEVEAFALCAEELKFVASQFRR.L
LOC_Os02g41710   RGYLDSYTTQGGRSGF.FNSCRIGAGEFCGEELLPWALDPRPAASL..........PLSTRTVRAVSEVEAFALVADDLRFVASQFRR.L
LOC_Os12g06570   RGELESSTTDGGRTNF.FSSITLRPGDFCGEELLTWALMPNPSLNF..........PQSTRTVRSVTEVEAFALRAEDLKYVANQFKR.L
LOC_Os09g38580   RGKLESSTTNGGRSNF.FNSIILRPGDFAGEELLTWALLPKTNVHF..........PLSTRTVQSLTEVEAFALRAEDLKFVANQFRR.L
LOC_Os02g54760   RGKLESSTTNGGRTGF.FNSTTLKSGDFCGEELLGWALVPKPTVNL..........PSSTRTVKALIEVEAFALQAEDLKFVANQFRR.L
LOC_Os06g08850   RGKLESSTTDGGRTGF.FNSITLKTGDFCGEELLGWALVPKPTVNL..........PSSTRTVKTIVEVEAFALRAEDLKFVASQFRR.L
LOC_Os02g53340   RGKLESISADGSK.......APLHEGDVCGEELLTWYLEHSSANRDGGRMRFHGMRLVAIRTVRCLTNVEAFVLRASDLEEVTSQFSRFL
LOC_Os06g10580   RGRLESISADGNK.......SPLQEGDVCGEELLSWYLEQSSVNRDGGKIKLHGMRLVAIRTVRCLTNVEAFVLRARDLEEVTSQFSRFL
LOC_Os03g55100   QGKLRSTQP..LAKGV.VATCMLGAGNFLGDELLSWCLRRPSLDRL..........PASSATFECVETAQAFCLDAPDLRFITEQFRYKF
LOC_Os01g57370   RGHLQSSQV..LRTGA.TSCCTLGPGNFSGDELLSWCMRRPFLERL..........PASSSTLVTMESTEAFGLEAADVKYVTQHFRYTF
LOC_Os05g42250   RGHLQCSQV..MRNGA.TSWCTLGPGNFSGDELLSWCMRRPFMERL..........PASSSTLVTAESTEAFGLEAGDVKYVTQHFRYTF
GRMZM2G005791    RGKLESSTTNGGRTGF.FNSITLKPGDFCGEELLGWALVPRPTTNL..........PSSTRTVKALIEVEAFALQAEDLKFVASQFRR.L
GRMZM2G023037    HGCLESVTTDGGRSGF.FNKVQLKEGSFCGDELLTWALDPKSAANF..........PVSSRTVQALTEVEAFALCAEELKFVASQFRR.L
GRMZM2G066269    RGRLESTTTDGGRAGF.FNSNVLEGGDFCGEELLTWALDPASGSNL..........PSSTRTARTLSEVEGFSLRARHLRFVASQYRR.L
GRMZM2G068904    RGYLDSYTTQGGRSGF.FNSCRIGAGEFCGEELLTWALDPRPAAKL..........PLSTRTVRAVSEVEAFALVADDLRFVASQFRR.L
GRMZM2G074317    QGKLRSTQP..LTKGV.VATCMLGAGSFLGDELLSWCLRRPFVDRL..........PASSATFECVEAAQAFCLGAPDLRFITEHFRYNF
GRMZM2G077828    RGSLESITTDGGRTGF.YNRSLLEEGDFCGEELLTWALDPKAGACL..........PSSTRTVMALSEVEAFALHAEELKFVAGQFRR.M
GRMZM2G090528    RGHLQSSQV..LRNGA.ESCCMLGPGNFSGDELLSWCLRRPFLERL..........PGSSSTLATLESTEAFGLDAADVKYVTQHFRYTF
GRMZM2G129375    RGNLMSMTTNGGRTGF.FNSDVLKAGDFCGEELLTWALDPTSTSSL..........PSSTRTVKTMSEVEAFALRAEDLRFVATQFRR.L
GRMZM2G135651    RGKLESSTTNGGRSNF.FNSIILRPGDFAGEELLTWALLPKTNVHF..........PLSTRTVRSHTEVEAFALRAEDLKFVANQFRR.L
GRMZM2G141642    RGKLESISADGSK.......APLHDGDVCGEELLTWYLEHSSANRDGGKIKFQGMRLVAIRTVRCLTNVEAFVLRASDLEEVTSQFARFL
GRMZM2G148118    RGTLESTTTNGGQTGF.FNSNVLKGGDFCGEELLTWALDPTSASNL..........PGSTRTVKTLSEVEAFALRADDLKFVATQFRR.L
GRMZM5G858887    QGKLRSTQP..LTKGV.VATCMLGAGNFLGDELLSWCLRRPFVDRL..........PASSATFECVEAAQAFCLDAPDLRFITEHFRYKF
consensus>70     rG.les.ttdggr.gf......l..gdfcG#eLl.wal.......l..........p.s.rtv..l.ev#aF.l.aedlkf!a.qfrr.l
```

```
                  610       620       630       640       650
AT5G53130        HSKQLRHTFRYYSQQWKTWAACFIQAAWRRYIKKKLEESLKEEENRLQDA......LA..K.............................
AT3G48010        HSKKLQHAFRYYSHQWRAWGTCFIQAAWRRYMKRKLAMELARQEEEDDYF......YD..DDG..DY.QFEED.MPES......NNNNG.
AT2G23980        HSRQVQHTFRFYSQQWRTWAACFMQAAWRRYIKRKKLEQLRKEEEEEEAA......A......................AS.........
AT2G24610        HSKKLQHTFRYYSHQWRTWAACFVQVAWRRYKRKKLAKSLSLAESFSSYDEEEAVAVAATEEMSHEG.....E........AQSGAKAR.
AT5G54250        VNEKVKRSARYYSPGWRTWAAVAVQLAWRRYKHRLTLTSLSFIRPRRPLS......RC................................
AT2G28260        HTKQLRHKFRFYSHQWRTWAACFIQAAWRRHRKRKYKTELRAKEEFHYRF........................................
AT5G15410        ANERLKRTARYYSSNWRTWAAVNIQMAWRRRRKRTRGENIGGSMS..PVS......EN................................
AT1G15990        HSRQVQQTFRFYSQQWRTWASCFIQAAWRRYSRRKNAELRRIEEKEEELG......YE..DE...Y..D.DE..SDKRPMV.........
AT4G30560        HSRQVQHTFRFYSQQWRTWAAIFIQAAWRRYVKKKKLEQLRKEEEEGE..........................................
AT5G57940        HSRQVQHTFRFYSHQWRTWAACFIQAAWRRYCKRKKMEEAEAEAAAVS..........................................
AT3G17700        RSHRVQGAIRYDSPYWRLRAARQIQVAWRYRRRRL.HRLCTPQSSYS...........................................
AT1G01340        HSKQLQHTFRFYSVQWRTWSVSFIQAAWRRYCRRKLAKSLRDEEDRLREA......LA..SQ...DKEHN....................
AT1G19780        HSRQVQQTFRFYSQQWRTWAACFIQAAWRRHLRRKIAELRRKEEEEEEMD......YE..DD...E..YYDDNMGGMVTRS.........
AT2G46450        QRQKLQRTFRLYSQQWRSWAAFFIQAAWRKHCKRKLSKTRDN.ENI............................................
AT4G30360        HSKKLQHTFRFYSHHWRTWAACFIQAAWRRYKRRVMENNLTAIESMENEEGEVGEELVVVE....E......E.............ECV.
AT5G14870        QSKKLQHAFRYYSHQWRAWGACFVQSAWRRYKRRKLAKELSLHESSGYYY......PD...ET..GYNEEDEE..TREYY....YGSDE.
AT4G01010        HSKQLQHTFRFYSVQWRTWGASFIQAAWRRHCRRKLARSLTEEEDRFRNA......IT..KR............................
AT3G17690        RSHRVQGAIRYESPYWRLRAAMQIQVAWRYRKRQL.QRLNTAHSNSN...........................................
AT2G46430        HSKQLRHMFRFYSVQWQTWAACFIQAAWKRHCRRKLSKALREEEGKLHNT......LQ..N.............................
AT2G46440        HSKQLQHMFRFYSLQWQTWAACFIQAAWKRHCRRKLSKALREEEGKLHNT......LQ..N.............................
LOC_Os06g33570   HSKQLQHTFKFYSQHWRTWAACFIQAAWHRYCRKKIEDSLREKEKRLQFA......IV..N.............................
LOC_Os06g33610   YRKQLRHTFRAPLNCS....................SEIVEEQENTLF..........................................
LOC_Os03g44440   HSKQVQHTFRFYSQQWRTWAATYIQAAWRRHLKRRAAELRRREEEEEE..........................................
LOC_Os12g28260   HSKQLQQTFRFYSQQWRTWASCFIQAAWRRHLKRRAAEQRRREEEEEEE.........................................
LOC_Os04g55080   HSRQVQHTFRFYSQHWRTWAACFIQAAWRRYYKRKMAEQHRKEEEAANR.........................................
LOC_Os02g41710   HSARIRHRFRFYSHQWRTWAACFIQAAWRRNKRRRASMELRMREGGEARP........................................
LOC_Os12g06570   HSKRLQHAFRYYSHQWRSWGACFVQGAWRRYKKRKLARELSKQEELYYMQ......GQGGDDG..DG.HDDSDSAPLLGAGVGAGGDHR.
LOC_Os09g38580   HSKKLQHTFRFYSHHWRTWAACFIQAAWRQHQRRKLAESLSRWESYSWWP......EE..H.P..PA.....D............KPKQE
LOC_Os02g54760   HSKRLQHTFRYYSHHWRTWASCFIQAAWRRYKRRKMARDLSMRESFCSMR......SD...D........................SNG.
LOC_Os06g08850   HSRKLQHTFRYYSHHWRTWAACFIQAAWRRYKRRRLAKDLSIRESFFSRR......SF...E............................
LOC_Os02g53340   RNPRVQGAIRYESPYWRTIAATRIQVAWRYRNRRLKRAGMSKLNDQSYNS......A.................................
LOC_Os06g10580   RNPLVLGTIRYESPYWKNLAANRIQVAWRYRKRRLKRAEMQRLQ..............................................
LOC_Os03g55100   ANEKLKRTARYYSSNWRTWAAVNIQLAWRRYKARTTTDLASAAQP..PSA......GG................................
LOC_Os01g57370   TNDRVRRSARYYSHGWRTWAAVAVQLAWRRYKHRKTLASLSFIRPRRPLS......RC................................
LOC_Os05g42250   TSDKVRRSARYYSHGWRTWAAVAVQLAWRRYKHRKTLASLSFIRPRRPLS......RC................................
GRMZM2G005791    HSKKLQHTFRYYSHHWRTWASCFIQAAWRRYKRRKMAKDLSMRESFNSVR......LD...E.......................VDNE.
GRMZM2G023037    HSRQVQHTFRFYSQQWRTWAACFIQAAWRRYYKRKMAEQRRKEEEAASR.........................................
GRMZM2G066269    HSKQLRHTFRFYSHQWRTWAACFVQAAWHRYCRRRLEEGVREKERMFRAA......AV..T.............................
GRMZM2G068904    HSARIRHRFRFYSHQWRTWAACFIQAAWRRYKRRRASMELRVREVRA...........................................
GRMZM2G074317    ANEKLKRTARYYSSNWRTWAAVNIQLAWRRYRARTSADLAAP.....PLV......GG................................
GRMZM2G077828    HSKAVQHTFRFYSQQWRTWAATYIQAAWRRHLKRRAAELRRREDEELE..........................................
GRMZM2G090528    TNDKVRRSARYYSPGWRTWAAVAVQLAWRRYKHRKTLASLSFIRPRRPLS......RC................................
GRMZM2G129375    HSKQLQHTFRFYSQQWRTWAACFIQAAWHRYCRKKIEDSLREKEKRLQFA......IA..N.............................
GRMZM2G135651    HSKKLQHTFRFYSHHWRTWAACFIQAAWRQHQRRKLAESLSRWESYSWWS......AE..DHP..TG.....D............KPRQE
GRMZM2G141642    RNPRVQGAIRYESPYWRTIAATRIQVAWRYRKRRLKRAEKSRLSEETYAS......L.................................
GRMZM2G148118    HSKQLQHTFRFYSQQWRTWAACFIQAAWHRYCRKKLEEALYEKEKRLQAA......IV..S.............................
GRMZM5G858887    ANEKLRRTARYYSSNWRTWAAVNIQLAWRRYRARASTDLAAMAAP..PLA......GG................................
consensus>70     .s......frfys..wrtwaa.fiq.awrr...r.........e..............................................
```

```
                    660       670       680       690       700       710
AT5G53130       ..EA.CGSSPSL..GATIYASRFAANILRTIRRSGSVRKPR...MP...ERMPPMLLQK..PAEPDFNSD....D.
AT3G48010       .DEN.SSNNQNL..SATILASKFAANTKRGVLGNQR.GS...TRIDPDHPTLKMPKMFK..PEDPGF........F
AT2G23980       .VIA.GGSPYSI..RATFLASKFAANALRSVHKNRT.AKST...LL.LSSTKELVKFQK..PPEPDFSAED....H
AT2G24610       .HHT.SNVKPHF..AATILASRFAKNTRRTAHK...........L....KDVEIPMLPK..PDEPDFSVD.....D
AT5G54250       .ASL.G.....................E..DKLR...............LYAAILTSPK..PNPDDFDD......Y
AT2G28260       .E.A.............ATARLAVNGG...KYTRS.GS...........DSGMMSSIQK..PVEPDFSS......E
AT5G15410       .SIE.G...................NSE..RRLL...............QYAAMFMSIRPH.....DHL......E
AT1G15990       .ITR.SESSSRL..RSTIFASRFAANALKGH.RLRS.SE..........SSKTLINLQK..PPEPDFDA......E
AT4G30560       ......GSVTSI..RATFLASKFAANALRKVHKNRI.EA.........KSTIELVKYQK..PSEPDFSADDT...S
AT5G57940       .SST.AGPSYSI..GAAFLATKFAANALRTIHRNRN.TK...........IRDLVKLQK..PPEPDFTA......D
AT3G17700       .....................................................L......................
AT1G01340       .AAT.VSSSLSL..GGALYASRFASNALHNLRHNIS..NLP...PR....YTLPLLPQK..PTEPDFTANHTTDP.
AT1G19780       .DSS.VGSSSTL..RSTVFASRFAANALKGH.KLRV.TE..........SSKSLMNLTK..PSEPDFEALDTDDLN
AT2G46450       ...P.QGTQLNL..ASTLYVSRFVSKALQNRRKDTA..DCS...SS...PDMSPPVPHK..PADLEFAKAE...A.
AT4G30360       .EES.PRTKMNL..GVMVLASRFAANTRRGVAAQ.........RV....KDVELPRFKK..PEEPDFSAEHD...D
AT5G14870       .EGG.SMDNTNL..GATILASKFAANTRRGTNQKAS.SSSTGKKD.GSSTSLKMPQLFK..PDEPDFSIDKED..V
AT4G01010       .ERN.AASSSSL..VATLYASRFASNALRNLRTN................NLPLLPPK..PSEPDFSLRN...P.
AT3G17690       .....................................................R......................
AT2G46430       .DDS.GGNKLNL..GAAIYASRFASHALRNLRANAA.ARNS...RF...PHMLTLLPQK..PADPEFPMDE...T.
AT2G46440       .DDS.GGNKLNL..GAAIYA........................................................
LOC_Os06g33570  ...D.GATTLSF..RAAIYASRFAGNMMRILRRNAT.RKAR...LK...ESVPARLLQK..PAEPNFAAEE...Q.
LOC_Os06g33610  ............................................................................
LOC_Os03g44440  .AAA.IRSSTGL..KTTMLVSRFAANAMRGVHRQRS.RRAD.......E..VLMMPMPK..PSEPDFGAD.Y....
LOC_Os12g28260  .AAS.ASSSCQI..TTTVLVSRFAKNAMRGAQRQRS.RRDA...........NLIVLPK..PPEPDFQTMEY....
LOC_Os04g55080  .QSS.SSHHPSL..AATIYASRFAANALRGVHRLRS.RA...........SPTIVRLPK..PPEPDFAVDEA...D
LOC_Os02g41710  .G.....................GS.....VRCRR..H...........SCDGKALIKK..PMEPDFTVEEE...D
LOC_Os12g06570  .DGA.AAGAAHL..GATFLASKFAKNTKKSAAAHH..GK...ARM.EDVSSIKFPKLAK..PDEPDFSLSSDD.VL
LOC_Os09g38580  GTSS...STKTIAESAIAQMHKFASASRRFRA.................DDTAIRRLQK..PDEPDFSADHF...D
LOC_Os02g54760  .EDD.SPPKQN.......LAMKIMSGSRKGPQ.................NMKELPKLRK..PDEPDFSAEPC...E
LOC_Os06g08850  ..DD.GSPEHS.......LVL...NAVRKGAH.................IIKELPKFRK..PSEPDFSAEHD...D
LOC_Os02g53340  .....L.........E..........RGARECDA.RQ...........HGRV......................
LOC_Os06g10580  ............................................................................
LOC_Os03g55100  .PDD.G....................D...RRLR...............HYAAMFMSLRPH.....DHL......E
LOC_Os01g57370  .SSL.G....................E...EKLR...............LYTAILTSPKPNPNQDDL........V
LOC_Os05g42250  .SSL.G....................E...EKLR...............LYTAILTSPK..PNQDDD........F
GRMZM2G005791   .DDD.SPPKNS.......LALKFIARTRKVPQ.................NMKELPKITK..PDEPDFSAEPE...D
GRMZM2G023037   .P...SSSHPSL..GATIYASRFAANAMRGVHRLRS.KA...........VPTIVRLPK..PPEPDFGVDDA...D
GRMZM2G066269   ...D.ISSSRSL..GAALYAAHFARNMVRTLRRNAA.RKAR...LL...DTVSSRLLQK..PAEPNFFAEE...D.
GRMZM2G068904   .G.....................GSL....LRSRR..H...........SIEGKASIRK..PMEPDFTVEEE...D
GRMZM2G074317   .PDD.G....................D...RRLR...............HYAAMFMSLRPH.....DHL......E
GRMZM2G077828   .E.D.EGKSNRI..RTTILVSRFAANAMRGVHRQRS.RRAV.......AVSELLMPMPK..PR.............
GRMZM2G090528   .SSL.G....................E...EKLR...............LYTALLTSPK..PNQDDL........L
GRMZM2G129375   ...D.SSTSLSF..MAALYASRFAGNMIRILRRNAT.RKAR...LQ...ERVPARLLQK..PAEPNFSAEE...Q.
GRMZM2G135651   GTSSGGGGTRTIAEGAIAHMHKLASASRRFRT.................EDVAIRRLQK..PDEPDFSADHF...D
GRMZM2G141642   .....G.........S............................................................
GRMZM2G148118   ...D.GTTSLSL..GAALYASRFAGNMMRILRRNAT.RKAR...LQ...ERVPARLLQK..PAEPNFFAED...S.
GRMZM5G858887   .PDD.G....................D...RRLR...............HYAAMFMSLRPH.....DHL......E
consensus>70    ..........................................................k..p.e.d.........
```
