# Supplementary material for: Genome-wide identification and analysis of the CNGC gene family in maize
Source: PeerJ. 2018 Oct 17;6:e5816. doi: 10.7717/peerj.5816 (PMC6195792; doi:10.7717/peerj.5816)
Supplement: File S3 — The multiple alignment was performed by ClustalX program. MEGA 7.0 was used to create the neighbor joining (NJ) under the poisson model. The tree was constructed with 1,000 bootstrap replications. [file peerj-06-5816-s003.pdf]

Supplemental File 3

The un-root neighbor joining (NJ) tree based on the maize, rice and *Arabidopsis* CNGCs protein sequences by using MEGA7.

The multiple alignment was performed by ClustalX program. MEGA 7.0 was used to create the neighbor joining (NJ) under the poisson model. The tree was constructed with 1000 bootstrap replications.

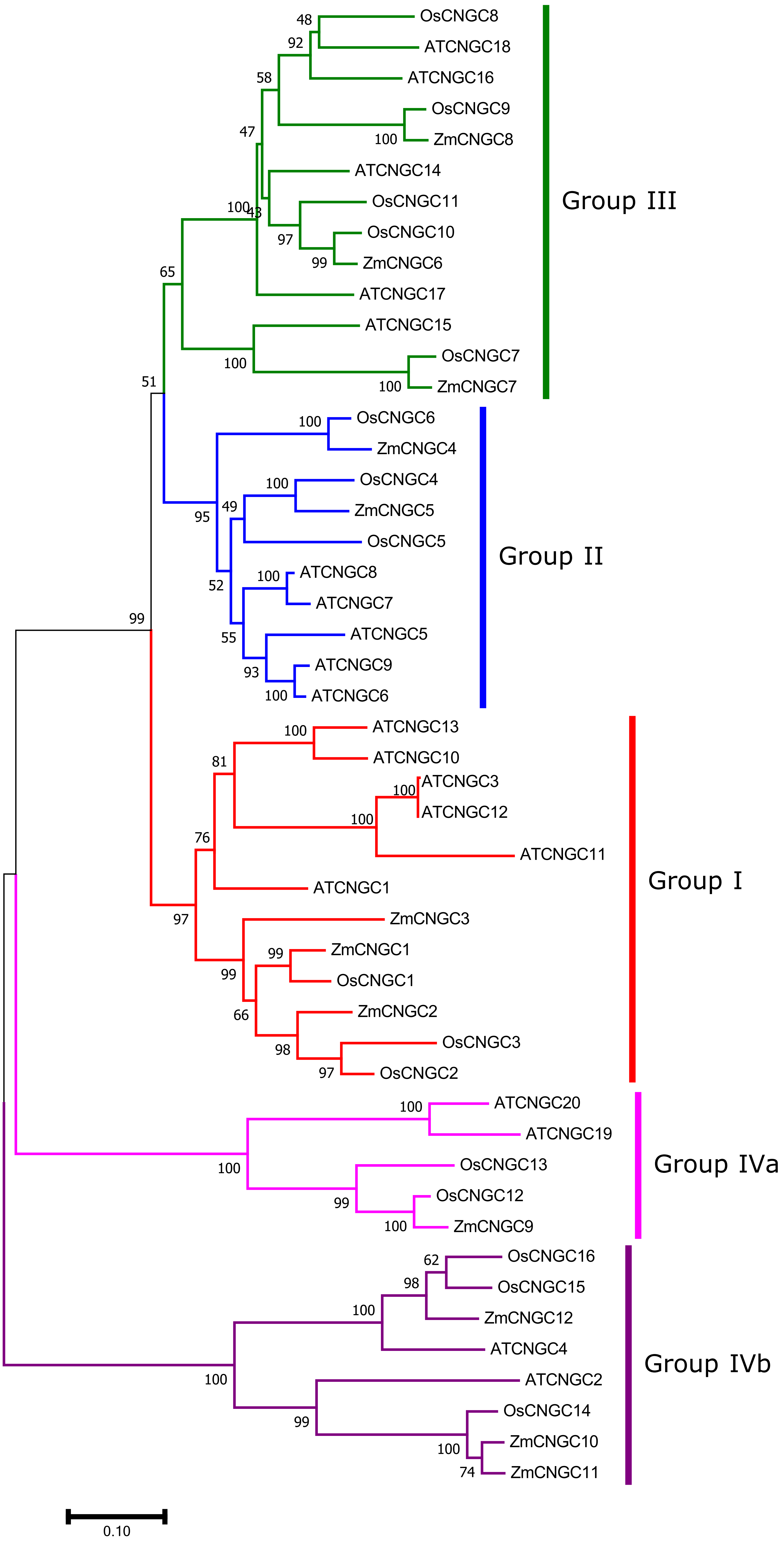

OsCNGC8
ATCNGC18
ATCNGC16
OsCNGC9
ZmCNGC8
ATCNGC14
OsCNGC11
OsCNGC10
ZmCNGC6
ATCNGC17
ATCNGC15
OsCNGC7
ZmCNGC7
Group III
OsCNGC6
ZmCNGC4
OsCNGC4
ZmCNGC5
OsCNGC5
ATCNGC8
ATCNGC7
ATCNGC5
ATCNGC9
ATCNGC6
Group II
ATCNGC13
ATCNGC10
ATCNGC3
ATCNGC12
ATCNGC11
ATCNGC1
ZmCNGC3
ZmCNGC1
OsCNGC1
ZmCNGC2
OsCNGC3
OsCNGC2
Group I
ATCNGC20
ATCNGC19
OsCNGC13
OsCNGC12
ZmCNGC9
Group IVa
OsCNGC16
OsCNGC15
ZmCNGC12
ATCNGC4
ATCNGC2
OsCNGC14
ZmCNGC10
ZmCNGC11
Group IVb
0.10
