# Supplementary material for: Genome-wide identification and analysis of the CNGC gene family in maize
Source: PeerJ. 2018 Oct 17;6:e5816. doi: 10.7717/peerj.5816 (PMC6195792; doi:10.7717/peerj.5816)
Supplement: File S4 — The multiple alignment was performed by ClustalX program. IQTREE was used to create the Maximum likelihood (ML) with JTT+LG4 model. The tree was constructed with 1,000 bootstrap replications. [file peerj-06-5816-s004.pdf]

Supplemental File 4

The Maximum likelihood (ML) tree based on the maize and other 11 gramineous plants CNGCs protein sequences by using IQTREE.

The multiple alignment was performed by ClustalX program. IQTREE was used to create the Maximum likelihood (ML) with JTT+LG4 model. The tree was constructed with 1000 bootstrap replications.

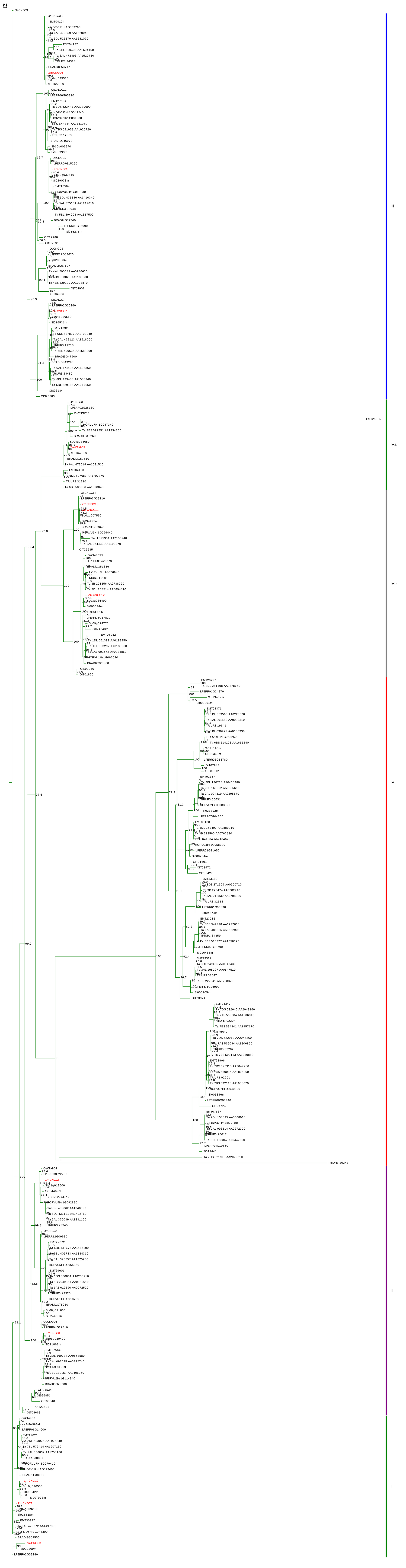
III
IVa
IVb
IV
II
I
