# Supplementary material for: Genome-wide identification and analysis of the CNGC gene family in maize
Source: PeerJ. 2018 Oct 17;6:e5816. doi: 10.7717/peerj.5816 (PMC6195792; doi:10.7717/peerj.5816)

Supplementary file 6

**The logo of ten motifs in study.**

The conserved motifs of ZmCNGC proteins were using the MEME Suite web server (http://meme-suite.org/index.html) with the maximum number of motif sets at 10 and optimum width of motifs from 6 to 200 amino acids.

Motif 1


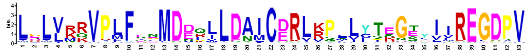


Motif2


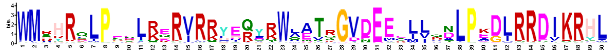


Motif 3


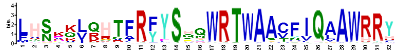


Motif 4


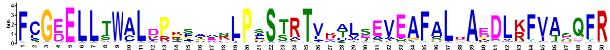


Motif5


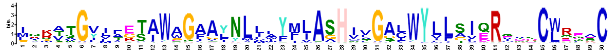


Motif6


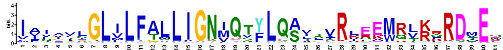


Motif7


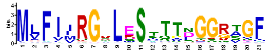


Motif 8


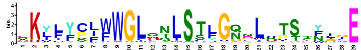


Motif 9


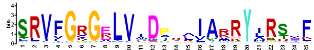


Motif10


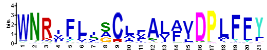

Supplement: File S6 — The conserved motifs of ZmCNGC proteins were using the MEME Suite web server (http://meme-suite.org/index.html) with the maximum number of motif sets at 10 and optimum width of motifs from 6 to 200 amino acids. [file peerj-06-5816-s006.docx]
