# Supplementary material for: Genome-wide identification and analysis of the CNGC gene family in maize
Source: PeerJ. 2018 Oct 17;6:e5816. doi: 10.7717/peerj.5816 (PMC6195792; doi:10.7717/peerj.5816)
Supplement: File S7 — Including DNA, CDS, Cdna, protein sequences of ZmCNGCS, and protein sequences of maize AKT/KAT channel genes and other plant CNGC gens. [file peerj-06-5816-s007.docx]

Supplemental File 7

**The gene sequences used in this research.**

Including DNA, CDS, Cdna, protein sequences of ZmCNGCS, and protein sequences of maize AKT/KAT channel genes and other plant CNGC gens.

**1.DNA sequences**

>ZMCNGC6

CCACTTCAAATATTTTTTAGGCTGCTTCAAAAAACATGGTTTAGAAGTGCTCTAAATACTTCAAGTCAACTCCGTTCTAGATGAACGCTCGTTTCCCTCCCGGCGACTACTTGCATCCATTACCGGCTATTGCTTCCATTACCGTCCGCGCTTAGCACTAACCACAGTGAAAACTGCGCCACTAACCGCAGCTTTAACCCCAATTCCATCAATTAGCGGCGACTGATAAAGTAATTAAGCCGGTGGCGAAGCATCCTCGTCCGCGTCCGCATCCGCACCGATCCGGTACGGGAGTTCTCGGCTCCGACGCCCCACGCGGCCCCGCCTCGCGCTCCTTCCTCGAACACCTCGCCGCCGGCTCTCCTCAGCTCCGCCGACTCGCTTCGCTGTCCTCCTCGCCCCCAGCCTCCTCCTGATCCCAGATTCGCCTCGCCGGCCCCCGGCGCCCGCCACGAGCGCCGTCAAGCGAAGCCGTTCTTCCCTGGCGCGCGCCGCTGGCGTAGCTGCCAGGCTCCTTCCGCCGAAGCACATCGCGGCCAGCTGGCGCTCGTCGTCCACCGTCGTCGCGGCGGACTTCGTCCTCCTCCCGGAAGCACAGATCCGCACAGGCTCGGCTGCATTTCTCCACTTGCTGGTAAGGAAGCTCGCTCCGATGGATTACTCCGAGGGTTAGTTCCGCCCTGGTCTTCCGTTTCTACGCTGCCAGCAATCAAGTTCCAACAAGTCGTCGATGGACTGCGATTTGTTCGCTGCGTGGTGGAGCAGCAGCACCAGATTAGTCTCCAGGATTTTTCGGGGGTCAGCTGATGCCCCGGGCCCGTCGCCAGCGAGGCCAGCCATGCCGCTTCACCAGAAGCAGGCGGGGCTCGCTGCTAGCAAGCTGGGCGTGGGGACCTCGAAGAAACACAGGGCTTTTGTCGCAAGCGATGAGCAGTGGTACAACAAGATTTTTGATCCGTCAAGCGACTTCATCTTGACATGGAACCGCATTTTCCTCTTCTCCTGCTTCGTCGCGCTATTTATAGACCCCCTCTATTTCTATGTGCCCAAGATCAGCTACGGCAGCCCCAAATTCTGTATCGGAACAGACACCCGTTTCGCCGTCGGTGTTACATTCTTCAGATCGATTGCGGATTTATTGTATGTCCTGCACATCATAATAAAGTTCAGAACAGCATATATCAATCCAAGCTCGACTCTGAGGGTGTTTGGAAGAGGGGATCTTGTCACAAATCCCAAGGAAATTGCGTGGAAATATATCAGATCTGACTTAGCTGTTGATGTGGCAGCTGCTTTGCCTTTGCCACAGGTAAATTGGTACTTCTATTGTTTTTTGTTCTTATTAATTATATAGTATACTACTTATGACCCTTGCTGATAAGCTGGTTTGTCATGAGTATTTGCTATTCTTGATCTTATTTTAGGCAATTATTGTCTACATACAACCAAACCACCAAAATTCACCGGGCAGACTATGCTAATAATTCTAGGTACACATATATATGTTGAACTTGAACCTGGGTTTGTAATTACTCTTTGCTGTTCTGATGCAATTCAAACAGGCTGTCGGGGCAGGTCCCTTTATACAACTTCTAGAAAACGTGTTATAGATATTTGTTAATGCTGCTCAGGTAACACTTAAAGACAACAATTACAACCATTTCCATGTTCAATCATCCTAGCCTGATTATCTGTGAAGAAGGCATTCCATAAATAAACAGTGCCATTTTCTTCTGTGATCTATGTTCAACTTCATCTTAAAAAATCGTAGAGCTGCGTTCCATTTCATTAAGAAAAATAAGTAGCAGGGGAAAGAACCCCAGAGTACAGCACGCCTAAAGCCTATTACATGAAAGAGAGAGGGAATACATGACCAGGACAGCCAAGAGTTATCCTCTCCTGCCTACTGCCCCCTCCAGCAGGGAGCGGAGACCCTTAGCTCCAGCCTCACAAGTCACACCACAGCGAGCCCTCATTGGCAATATTTTGGAGCACCACCGAAGCGTCCGGTGCAGAGCTCTCAAAGGTGCAGGCATTTCGATGCTTCCAAATCTCCCAAGCTACAAGAAAAACTAGAGTGTTGAGGCCCTTTTGTAGTTCCTTTGGTGTCAATCTGATGGAACTACTCCACCACTTCATAAAGCTGCCATTCCAGGGTTGAGGCAAAACAGACCCAAGTCAAAGAGTAGTAGAAACCAAACTTGTTTGGCAAAGACACAGGAGACCAACAGGTGTTGTATTGATTCAGCCTCTTGATCACAGAGAGGGCAAGCTGCAGCATGCGGGAGGCCTCTCATCGCAAGGCGGTCTGCATATTTCTTTTAGTTTGTGCACTTCTAGTTTGTGTATCAAAAAGTATGTGTACATACATATTTCTTTTGGAAACAAGTAACTGCATCCATTTCAATACGGTTTCATTAGTATCGTTGTTACATCATTGACAAACACATGTCATTGTGTGTCCTGTACTGTACCTATGTTAAACTTTATTTCCTTCGTCCCAAACTATAAGCCACTTTAATATGTATTTAGACATAATATATATCTAGATGCGTAGCAAAAGCTACGAATCTAAAAAAGCCAAAATGTCTTATAATTTGGAACAGAGGGGTAACTTCCTATTGCTTCTTCATTTCCTTTTGTGTTCCAGTACTGAACATCGTTGAAACATCAGTTTCATGTTAGTTTGTCAGTTGATGAGCTTACTAGTTACTGCAATTGGCTTTTAAATATGAAACTTCTATCATGTGCATAACATCTGTCTACTGGAAGCCTAGGACCTTTTATTTTGCTCCCATTTAATGAATGCCATCGCCCCTGCCAGTTTTGGTTAAAACACCTAGTTTTTCCTGGTTCCCTTTTATTGAGCTATATACGCCTTAGAGGCCAAAATAATAAAGGAAAAACATTTTCCTGTGCTCTCTTTGGGAACTCCACCTATGTTGTTTGAAATGGTCCATAAAGCCCAGATGAAGGCGGAGCCAGTCATATGGACATAGGGGTGACAAAGGGGGGTTCTAAATTTTACACTACAAAATTTAAGGATCGAATCGGATTAGGATCGGACCCTATTTCTATTCATTTTCGAACTAAAATTTATTTAGAGTCCTACTATTTTGTGAAGAAGCATTTGGATCGCGATCCGTTACCACCCCTATATGGACATGGAACCCAATAGAAGATTGTTGGCATGTAACCCTCCTTTCGGTTATTATCTGTTTCCAGTTTGGATTGTACCTTTTATTTACACATCTTCCACTGTTTGTGCAGATTATTGTCTGGTTTGTGATACCAGCTATAAAGTATTCCTCTGCTGAGCACAACAATAACATTCTGGTGCTCATAGTTCTTGCTCAGTATCTTCCAAGATTGTATCTCATATTCCCCTTAACTTATGAAATTGTCAAAGCTACTGGAGTTGTTGCAAAGACTGCTTGGGAAGGAGCTGCATACAACATGGTGCTCTATCTGATAGCTAGTCATGTATGACACTCATATGTACTTAAGTGGTTTCATCATTCCGTATCTTGATGTAACTAATTGTTAGATGTGATGCAAATCATTCATCTTTCCCCCCTTTTCTTTCAGGTGCTAGGTGCACTGTGGTATTTGCTATCTGTTGATCGCCAGACATTCTGCTGGAAGACGAACTGCCTGAATGAAACTGGTTGTGATCTTAAGTACCTAGATTGTGACACGACACCAAATGCTACATGGGCGAATACGACTGCTGTCTTCAGTAATTGTAATGCTAGCGATACCAATATAAGTTTTGATTTTGGCATGTTCGAGCCTGCATTGTCTAATCAAGCCCCTGCTCAAAGTTTTGCGATGAAGTATTTCTATTCCCTCTGGTGGGGATTGCAGAATTTAAGGTATGTCCATAGCAGGCATTTTTGTAACTATAGTTCAAATTAGAGTGCTATATGGAAACTTATCTTGTGTGTTCCTTTCATAAACCAGCTGTTGTTTACACTGTATGGGTTAGGGACCTTGACAAGAACAACATACTGCATCTTACTCTTGATTTTGCAATCGCACTTTCACATTTAAGCTTTGAAAGTTTAACCTAATTCAGTATTTCTTTAGTGTGTTCCTCTCTTTTTAACTTTTACAGTTCTAAGCTAATAACTTTATATATGTGGATTTGCTAATGTCCTATATATTCTTTCATTCTGAAACAGCTGCTACGGTCAGACTCTTACTGTGAGCACCTATCTTGGCGAGACGCTGTATTGTATATTCTTGGCGGTACTTGGTCTTGTCTTGTTTGCGCATTTGATTGGAAATGTGCAGGTACTTTTTATCCACTCTACTGCATACTTTATATTGTACAGGCTTTTTATATGTGAACAATAATACTCGCTTTGGATAATTTATTAATTTTGACATAATTTGGAATTTTCTGTCTTACAACATGATGATTATTATCAAGACCTACCTGCAATCTATTACTGTGAGGGTTGAGGAATGGAGATTAAAGCAAAGAGATACTGAGGAATGGATGAGACATCGTCAGCTTCCTTGTGAACTGCGGGAAAGGGTGAGACGATTTATCCAGTACAAGTGGCTTGCAACAAGAGGAGTGAACGAAGAGTCAATATTGCATGCTCTGCCTGCAGACCTTCGACGTGACATTAAGCGCCACCTTTGCCTGGGTCTTGTTCGACGGGTAAATACTTCTACATGCTTTTGTCAATGTACATATGAAATAAACTTTTGAAACTTCTATGGTTGGATGATGGTTACCACCATTTCATTTCTGCATATACCAAACTTGTCTATCACCATTATAGCCATGAATGTATATGTATCCTTTTTATGTTAGAAGTTGACATCATATTTCTAGTGTCGCTAGAGGAATTTGAGGTGATATGCTTCTCATGTTGAGGATACACTTGTAGTCAGGAAATGTCTTGGTACAGCTACAATGTGTGTTATGGCCCTGTTTTCACTATAAAAAGGAATTTTTTTCAAGTACACCACTCAAATGTTGCTGAGAATCCAGAGTAACATATTTTAGCAATACAAGTATATATCCTTTGCCCCCTGAAAAAGATTGCATCGTAAATTTGAACTACACATTGCTTTCTACATAATTGCATGGTAAATCTCGCTTTGTATCGCAATGTTGCAGGTTCCTTTTTTCTCCCAGATGGATGATCAGCTTCTTGATGCCATCTGTGAGCGTCTTGTATCATCACTGTGCACAAAAGGCACATACATTGTCCGTGAGGGTGATCCGGTGACAGAGATGCTCTTCATCATCCGTGGAAAACTGGAAAGCTCCACAACAAATGGTGGCCGCACTGGCTTCTTCAATTCAATCACCCTGAAACCCGGTGATTTCTGTGGCGAGGAGCTTCTTGGATGGGCTCTTGTCCCCAGGCCTACTACAAATTTGCCGTCATCCACTCGGACAGTGAAGGCACTGATAGAAGTAGAGGCCTTTGCGCTCCAGGCGGAGGATCTCAAGTTTGTTGCCAGCCAGTTCAGGCGGCTGCACAGCAAGAAACTGCAGCACACTTTCCGGTACTACTCGCACCACTGGAGGACGTGGGCCTCATGCTTCATCCAAGCTGCCTGGAGACGGTACAAGCGAAGGAAGATGGCAAAGGACCTGAGTATGAGGGAGTCATTCAACTCCGTTAGATTAGACGAAGTGGATAACGAAGATGACGATTCTCCGCCCAAGAATAGCCTTGCTCTAAAATTCATAGCTAGGACTAGAAAAGTGCCTCAGAACATGAAAGAGTTGCCGAAGATAACGAAGCCAGACGAGCCAGATTTCTCAGCTGAACCCGAAGACTAAAGTTGTCTTTGCTTATGCTATCATGTACAGTGGTCATAGCTGACTATTGTGAATTGCTAGTCCTATTTGGTTCGTGATGGAGGTTACAAGCCAACTAGGTATGGTGGGTGAATTGAACTTGGGTTGACGGTGCCATCTGTGGATGTGAGGAAGGTAACTCAGAATATCAATGAAAGCGTAATGAAGCTTCTGACTGGTATATAGACTGAATGGTTGTAAGTTAACTAGTTGCTACGTTCATGTTGTATATTCAGACATCTCTGTGCCTATAGATTTCTTGAAAAAAAAAGGAAAGAAAAAACAAGAATACATCATTACATAGACTGTAATCTTAAACATCTGACATAAGGGTGTGGCTACGTTGTCCGTGGCCGTGGAGGACTGTAAATCATGAGGGTGTGGCTTCTGGTAAAATGGCTAGCACCACGCAGTGATGAGAGATATCTCGTTGAGTCATCAGGGCGCAGATTCGAACAGGGGCGGAGCTAGGCCGCGGATTCGAACAGGCGCAGAGCTAGGCCTGTTTATCGGTGTCAGCTGACACCGGCGGCTTTTTGCAAAATCAATGAGAAATATTTGATATGCGTTGTTTATCCATAGAGCTGATATCAGTGTCTAGTGGAATCGGCTCCTTCAGCTGGCTTCGCTCCGCATTTGTGAGACAAAGGTTT

>ZMCNGC4

TTCTGGTACAACCGAACAGGCCCTTAGTCGTTGACCGTCTCGCTCGCCTCCCCGTCCGCTCCGCTCCCGTGCTTTCCCCTTTGCTCGCTCGCGGACCTCCGTCCCGGACTCCGACTCCCGGCGGCGCTCTCGCGCCAGCAAGCTCGAATCTGCACGCCGCCGCCGCCGCCGCCGCCGCCCCACGCCCACGGCGCCTAGGTAACCGTCTTACCCTCTTGGTTCCTCTAAGTACTCCTCGGACCCCTTCCACCGCGTGGAGTTCGTCAGGAAACCGTCCGTTCGATTCTGGGAGCTATGATTCGCGCCCTCTTCCTTGACGAGTAGATCTGGATTAGCCTGCGAGAGGACGACAACTCCACTAGTTCGGGCGCTACCGCTGTTATTAGTCGCGCGGGGAAACGGAGGCGTAGCCCTGCGTCTTCTTCTCCCGCGGCACTCCTAATCGTGGAAGTCAAACAGTGACGACGATTACCCAGCGCCGCTCCTGCAGAGCTTAGCTGTAGCTTTACTCGCTACAGCTACGGCTAAGCTCTGCAAATTTAGAGTATTTGCTTATTGCTGGCTGTTGTGGAACTCTATATTTAGGATGTTACTGTCATGCAAATGTCAGTGTTTCCTGAACTGGCTAGTTTTCCTGTATGGCACCTACAGTACTTGCCATTTCTGTATGCCCATGCTTTGTCCGTGAGGTGGTGTATTACTTTTTTTGGTCGATGTTGATTGTGCTTGTTGTTGGTCAGTGTATTATTGTTTTTTTTGCGGGTATTGATTGTGCTTGTTGGTAAATTGTATCAGAACCAGGGTCTTTCTGGTGTGCAGGGGTGGGCGCTTGGCTTCTGAGAACATGTAACGGGTGTTGATACGAAAATAGGTTCCTGGAATAGCGGGTTCATTAGCAGTTTGGCACAACTTTGAAGCTGCAGGGGATTTCAAATGCCCTTTGTCATCAGGTCCACGGTTCCACTGAGCCAGAGGTATGCTGCTGTGCTGGTTTTCTTTGACATCGATTTCATCATGGCTTTAGTTTCAAAATTTCTTCAGTATCTGTAAAATGTGGTTCTTGAATGCTTCACTTCGGGTCTGTATCCCTATTTCAAATGCCCCATTTTCTTCTAGCTTTCTTCAATCTGGTGAGCCTTGTGTACAAGAAAAAAGAAATGTTTTATGCATGCGATGGAAATGTCAGTTCGATGTCATGATTTTTATGATGCAGTCTGCCCTTATATGATTCTGGATGTTTCCATAGGACGCTTATCTTATATTCCTGAATCTTCAGCTTTACTTGAATGAAAAACTAATTAGAAGAAAATGTCATTGCTCTGGCTGAAGAAAATGTCCTTGTTTGACATATTAGCTGTTCGAAGCCTGAAAAATTATATTAATATTTGAAATTGTATAACCAAACTATCAAAAGGTTTCATATCTGTAAGGTTATATAAAATATTTATATATTAGTAACTTCCAGCATTATTATTTCTACTTCACTCACTTAGTGTGTCAAAGTTACTGATTTACTACTATTTCCATTCCTATGATATGTACTTATGTACCGTCTGTGTCTGTTATCCTTTCTGTGTAATTCTGGCAACTAACTATTTCATTGTCATATCTGGCTGAATTTCCAGGATTTTTTATTGTATTTTCAACTTCCCTGAGGATGTTTGACAGTACTCAAAAAGCTCAATACATGGATGGGCACAGAGAGAGGTTTATTAGGTTGGTTTATTTATCTTAAAGGCACTTCTAGTCTTCTACAATGTTTTTACATCTATTCTTATTCGCTCTGGGAGTATGACATGTTTTATGTAGTATTGCCCATTTCTCTGTGTATATTGGAAATCCTATTTCTGGAAGATAATATTTTTTGTACAATCCACGGCTGCAACAGTATCCCTAAATTGTTCTTCCTATAGTTTATAATTTGTCCTCTTCAGCTTTAACAAGTTCAAAATTGTTTACTATTTTTGTTTTGTGGTACCCTAGAAATCTGATTTTTATCCTAGTACTCTTTCATTGTTCGAATTGGTCTTTGCTGCTTTACAAGCTAATAATTTTTGTCAGACCTATGAGAGTCTTTTCCATGTTGAGCGTTCAGGAGTGTTAAAGTAATAAATGCTTTTATCAAATGTCTTATAGACCTGAAACTTCTTGCACTGCATAGAATTTCCATGTTTTTTGTGTTGAACCCTTAGAGTTTTTAAGTTGTATCACCGATCACCATATTTTCTGTAGGCTGGATGAGTCAAGTCCTAGGTCATCTGTACCTTCTGAAGTGGGAGGCAGGAGTACATTGAGGTCCAGCATGCCTGGGTTTGGTTATGGTCCATTTAATGCACTAAGGTCTTTCTTGTCTGGGGGCTCTGGAAGACTGAAGTCACTTAGACAGTCACTTACTTCTGGTGCTCCTAAGACAGCTTTTGCGGAAGATCTTAAATCATATAAGAGAACTATATTTGATCCCCAGGACAAACTTCTTTTCCGAATGAACTGGGTTTTCTTCTCGTCTTGTCTTTTTGCTGTTGCAGTGGACCCACTATTCTTCTTCCTACCCATCATCAACGATTCAAACTGCATTGGTATTGATAAAAAGTTGGCAGTGACATCAACAATAATACGTACGGTTATTGATTTTGTCTACCTTATACGCGTGTGTCTTCAATTCCGCACTGCTTATGTTGCTCCATCTTCACGCGTGTTTGGGACTGGTGAGCTTGTGATTGATCCGATGCTAATTGCAAAGCGATACATTAAAAGTTACTTTGCAATGGACTTTGTTGCATTGCTACCACTTCCACAGGTACATCCCCTTTCTCTTGGTGTCAGATAATGCATTAACTGCTGCACCAGTTCGTTTGACATTTTTTTAGTATGCCACTTGCCAGCATTTCTAATATCTACTTCATTTGGTTACCTTAGTACTTAGAAGTGGATGTTGGCAGTAATGGCAGATGTTCAGCAACCAAAGCTCTATCCTGTTTTCCAGAATCAGTAGAAGTTAGTTTCATTCGCAGAGAAAAAGCTGTGCTTTATAAACTTCCCTACTTGCATTTTTCTTAAGGAGGTTTTCTGACAATGCTTTTGAAGTTCAAGTTCTGTTAAAATAAGTTCTGTTCCTCTAGTTAGGAGGTTTTCTGATGATGCTTTCGAGGTTCAAGTTCTGTTAAAATAAGTTTTGTTCTCCATATGATAGCCAGAAGCCCAGGACAACAATAAGTTGTAATATGCATAAAGAATTTCATTGTAGTCAACACATTCCTTTGTTCTCCAAATTTTGAATTCCCACAATCTAATTGTTCATGTGCGCACTGAATGTATACCTCACTGTAATTTACTACTCCCTCCGTTTCTTTTTAGTTGTCGCTGGATAGTTCAATTTTGCACTATCCAGCGACAACTAAAACGAAACGGAGGGAGTACCTCCGTTCCAAAGTGTCAGATGTTTTGAGTTTTCTAGATACATTGCTTTTACTATGTATTTAGACATAGTGTATATCTAAATGCATAATAAAATCTATGTACCTAGAAAAGCCAAAACATATTACAATTTAGAATGGAGGTATTACTACATTAAGTTACAGTCACCTTTAATCTCCTTCCTCTTCATCAACAAAGAATATTTACATTTCTATGCAATATGCATTTAGTTCATATCTGAAAGGTAAATTATTGCTTCACTACCTTGCGTCCTTGTGCAGTGTTACTTGATTAAGCCTTGACACTGTTATATGTATGCATAAGACTATCCCAATTGCTAGCTGAACTTATTATATTCTTGTTCACATTCAGAAGCTTTCTTGGTTCTAATAATTTCCCAGCTTACCTTAAACTATCTGTTGGTGGTTGACAGATTGTTGTATGGAGATACCTCCATATTCCAGATGGCCCAGATGTACTGACTACGAAAACTGCACTGGTTTGGGTTGTTTTGATCCAATACATTCCAAGGTTGCTTCGAATATTCCCTGTGATCACAGATTTGAAAAGGACAGCTGGTGTTTTTATTGAAACTGCTTGGGCTGGTGCTGCTTATTATCTTCTATGGTTTATGCTGGCTGGGCATGTAAGTGCTTCCATAAGCTATGTACTATCAATTATATTTTCCACATTCTTTACTCATTAGTGAAGGACAGGCCTGGCACAGTGGTGAGAAGCCTTTCCACTGAGCCACAAAGGTCCTAGGTCGGCACAGCCTCTTTGCAAAACGCAGGGGGTAAGACTTGCCTCGGCTATTCTTTTTCTAGACCCCACTCATGTGGGAGCCGCTAGCACTGGGTCTGTCCTTTTCTTTACTCATCAGAAGCAGGCAGCAGTAACGAGGTCGATCTTTTGACTACAATATGTCAATATCAGTGATATGTACCTGAGGTTCTCTGATATTCTAAATTACTGATGACTTTAAACTCTTTGTTAAAAAATGTGATACATAGTCCAGTTGAAACTGTTTGAAACTTTGAATCCTTCAGCCATTAAGTTAGTTCTTAGATGTAAAATGAGATGTTTTGTATATATTTTAAACAGCATCATCTTTGTTTAATTGATCTTCAAAGATTTTCCTTTGTTGACTTATGCAAAAGAAATACTCGCTTTCAGCACTATACATCATGGTTGTTTTCCATAAGTTTATATCAGAAATTATGTTTCACATGGCTTATGCTGTACCTTTTATACATGATATCTATGGCACAGGAAAATTCATTAGTTTCCATTTGTTCATTGAAACAGCCTCTTGTAATTTTCATTTAGTGTTTTCATATCACCCATGACTTATGCTGTGGTTTCTATATGTATTATTTGCTGCAGAATGTTGGTACTTTATGGTACTTTTTAACCATAGAACGTGAAGATGATTGCTGGCATCTATACTGTGACGATCCCAACTTTGGCTTGGGATGTAATAGCAGCTACTTGTATTGCAATAATCATCATCATGGCAGCTATGATAGTTGGCTTACGAATAATAGTGCCCAAGTATTCAACATGTGCAATGGCGGTCAAGACAATCCTTTCAACTTTGGCATTTATGAGCAAGCACTGGTCTCTAAAATACTTAGTCCAGGAAATTTCATCTCAAAATTATGTTATTGCTTCTGGTGGGGATTGCAAAATCTAAGGTACTTTATTGCTGTTGTTTATGATATTTTTCATAACACAGAATTTTAATGTGTTTGGATAATCCTGAAGCTGAACGATGTCCATAAAATGAGCATGGCTTGCTAGGAATACAAGCTTGCTTTTCATCCTGTTTTAATACGATATTAAATTCAATCTGTCACCTTTCTAAGAAAAAAATATTCTGTGTAGCTAATGCAGCTATTGTTGTGTACACAATGGTCAATTGGAAAAAAAATATCTCGACCTGTGGGGGTAAGACAACCCCCGAGCATTATTATAAGAAGAAGACTTTCTCAGTCGTGAAAACCTTCGAACCCTTGCCCCACCCATGCACAGCTACATCGTAGCCCATGTGAACGACCGCGAACAGACGAGAGGATTTTTTTAACCATAACCTGAAATTCGCTTCCACGGGAAGTGAACCTAGGACTTGAGGAGTGTAGAATAATTGAAGTGCTGTATTGCATAGAGAATGGGTACAATATATAGACTCAACCTAACCCTAATGGGCCCTAACCATAATGGGCCGGCAACCCAACAGTGGTGCCGACCCACACACTCACACACACAGTCTAACATCCCCCCGCAGTCGCAACGGGGGCACCACACACGATGAGACTGGAGTAGAGGCCGAAGGTAGGAGCCGACGGGTTGAAATCCCCCCGCAGTCGCAGCGTCGTGATGGTGCGAATGTTGCGGCTAGAGTAGAGACCGTTGCAGCTGGATAGCCCCTAGATGTCGAGGTAGCCGAAGTCGAGGTGGTCGCGGTCGGAAGACGCGCAGCAAAAGCCTGATCTTCGGGAGGGGTCGACGTTCGAGCGTCAACGATCGGCAGGGCGACACAACAAAAGGGCACCAGCAGGCCGACCTTCCTGCTTCTTCGATCGTCCAGGCGTCAAGGAGACTCGCCAGGGAGGCCGACGGCAGCGCACGCGTCTGCGCCGGTCAGGGTGTCCGCGCCCGCTGCAGAGTAGAAGGGGTATCGGTGGATCCGGCCGGGAAGGCCACGGCAGCGACGAATCCAGCCATGAAGACGGCGATCCGGCCAAAGGGATGGCGGATCTAGCCGAGAAGGCCGCAGCAACGTGGATCCGGCCGGGGAGGCCGCGGCAGCGACAGAACCAACGATGACGACGATGAAGGGGGAGGGCGACGGGGCGGGTCCAGCCGAGCAGGGGCCTGCGCCGGACCTGGCAAGGGGTGTTGGCAGCACCGGCGACGGGAGAAGTTCGAAGGGTGGTGCTACTGGGTGCTGCTGGTCGAGGTGATCGACACTAGGCTCGCGAATCTAGCGACGCAGACACAAGGGCGCGCGGATCCGGTGGCGACGGCTTGGGAGGCCGGGAAGGGGAGGGCCGCGCCCACGGCCAGTGAGGCGGCATGCGTGCAACCAGGGAAGGGGAGGGGCTGGCCGACATGAGGGAAAAGGGGAGGGCAGCGGACCGACTGCGGCGGCTTGGGGGCGCCGCGCCAGGGAGGGTCGCCGGCAGGGGCCGCGCAGCAGGGGGAGCCGGACGAGCAGGAGCTGGAGTGGAGGAGCTCGGGCGCCGAGCGAGGGAGAGTAGGAGAGAGCGCGCGAGCAGAGGGGCTGCTCCGGCGCGCCATGGGGAACCGCGCCGCCGGGAGAGGCCGCAACCGCTGGGGAGGGGCTGCGCAGGGGGCGCGGACGCCGGAGAGGGGGGAAGAGGGCTGGCCGCAAGGGAGCCGGACGGGTGGAGGAGAGCGTGGCGGTCTTCGGCGGGCACGACCGCACGCCGGGCTTCCTGGCCTCCTCCCGCCGCAGGAAGAAGCCGCAGCACCGGGCAACGCGCGGAGCTTCCGCCGGGCTGTTGCCCGCGTCGGGGCCGGGGTGGGCACGCCCGCGCAAGGGACACGCGCACCCGCGCCGCGCGCCGTCGGAGCCGGGGGCTGCCGCGCCGAGGCTGGGCAGCCACGCCCGCAGCTCCCGAGCCTTCTCCTCCGTCATCACCCCCACGAACGGCCTCCGCGACGCCTCGTCCTGCGCCGCCGCCGGGGCCGGGGCCTCCAGGTAGTCGGCCAAGGGAGCTTGAGGGAGACCGACGACCATGGCTGGGACGAACGGAGGAGCTGCTCTGCCTGCTGCGTCGAGGGGAGGAGAGGAGCGGCTGGAGGGAGACCGTGCTCCCTGGAGGAGCTGCTCGCAGGGGAGGTGCCATGGGAGAGATGGTGTACGGTGGCTGGATGGAGGTGGGAGGCCGCCCCCGGGAGGGAAGGCAGGGCCTCCCGGGGGCAGCGATGGCAGGTGGCGGCTGTGGGGTGGGAGGAAGCCGACGGCGGCTGCAGTGGGAGGTGGGAGAAAAAACCCTAAACCTAATCCTTTGATACCATGTAGAATAATTGAAGTGCTGTATTGCATAGAGAATGGGTACAATATATAGACTCAACCTAACCCTAATGGGCCGGCAACCCAACAGTGGTGCCGACCCACACACTCACACACACAGTCTAACAAGGAGTGCTACTCAGACCACCTAACCAAGTCAGCTAGAGGCCCTTTCACCACAATGGTCAATTGAGATACAACTGTAGTAAATATGTGGAAGTAATTGGGAATCTTAAGGCCCTATGAAGGAGGTTATGGTGTTCACAATACATTGTATTTCACGTGCATTGTAGTAGATGAAGTTGCACATATAAAACCAACATTCCAATATAGATCAATTTTGTATCTTCAAGGTGCATAAAAAGGTCATGTGGTTCTATTTAGGAAGATATCGAGAACCACATGGGTTGCAGCCAAATTGCTGATGATATCTGTAGATATACTGAAGAACACGTGCTGTAGGATTGGTAACTGCGAGTAAAATATGTTTTTATAGTTAAAATATGTTCAATGTATATAACCATATATCTATTTTTTCAGCATTATCTTTTTTGAGTTAAAATTAATAATAAAGCTGCAAAATACTACATCGATGTGGTAACTTACATCATGAGGCTGCCCTTTCTTTTAATAGGCTAACCCTTTGCAGGGTTCACGAAAACTAAATCTTTAGCCCTAAACCCTCTACAAGTCTACAGGGTTCCATGTTTTCCAAACTAGTGGTGGAGCTATAGTAACAGCTGGAAAGTGGTGGTTATATGTTGTTTATGTCTAACAGAATGGTATCATCAATGCTGACACAAGAGTGAACCACAGATGAGTGGTCAATCTGGGTTAGAACCAGAAACTGATGGGTTTGCAGCTCCACTTCTTAGGTTCAGGACTTCACATTTAGGTTGTTCTGTGAATTGGGTTCCTGCACTTGGCTGTTGAAATATGCATTCTAGCTGTTGTAGGTTCACAAGATTGGCTGTCATCATTATATGTTCTACTTCAGAGCGTGTTATGCCTGCCATCTTAATTTCCATTTCCATTCGACAGTACACTTGGTCAAGGGCTTTTGACAAGCACATACCCTGGAGAAGTCCTGTTCTCTATAGCAATATGTGTCCTTGGACTAATTCTTTTTGCTCTCCTCATTGGTAACATGCAGGTAGAGTGATATATTGTTTCCCTTTTGTTCTTTTAAGCCTTTTCCTCTTTCTTTGCTTGATTCCAAGTTGGATTTATATAGAGCTACCTACAATCTGTTGCTATACGCCTTGAAGAGATGAGAGTTAAGAAACGCGATGCTGAGCAGTGGATGCATCACCGTTCACTGCCACTGGACATCAGACATAGGGTGAGGAAGTATGAACGTTATCGGTGGTTGGAAACCAGGGGAGTAGACGAAGAAACTTTGGTTCAAACTCTTCCAAAAGATCTTAGGAGGGATATCAAGCGGCATCTTTGTTTGGGCTTAGTGAAAAGGGTACGTTCTGGTTGCTCAAACTATTGTTTTGTGCTTTGTTTAGTGATTTCCAGTTATTTAATATCCTCATTTTGTTTTTGCTACTGATAGGTGCCTTTGTTTGAAAATATGGATGAACGATTGTTGGATGCAATATGTGAGCGGTTAAGACCTGCACTCTACACAGAAAATGAGTTCATTTTGAGGGAAGGTGACCCGGTGGATGAGATGCACTTTATTCTTCATGGTTGTTTGGAGAGTGTAACCACTGATGGTGGACGGAGTGGGTTCTTTAACAAGGTTCAGCTAAAGGAAGGGTCGTTCTGTGGCGATGAGTTGCTCACTTGGGCATTGGATCCCAAGTCAGCTGCTAATTTTCCAGTTTCGAGCAGGACTGTCCAGGCACTCACCGAGGTTGAGGCGTTTGCCCTATGTGCAGAAGAGCTGAAATTCGTGGCCAGTCAGTTCAGGAGGCTGCACAGCAGGCAAGTGCAACACACATTCCGATTCTATTCCCAGCAGTGGAGGACTTGGGCAGCCTGCTTCATCCAAGCAGCATGGCGCCGCTACTACAAGAGGAAGATGGCAGAGCAGCGGCGCAAAGAAGAAGAGGCGGCAAGCCGGCCAAGTAGTAGCCACCCTAGCCTTGGGGCGACTATCTATGCATCTCGTTTCGCTGCCAACGCCATGCGAGGGGTTCACAGGCTAAGAAGCAAGGCTGTCCCTACCATTGTCAGGCTACCGAAACCCCCAGAACCAGATTTTGGTGTCGATGATGCTGACTAACGAGACAATCACACTGTACTCGCTTGTAATTGTACTTTACCTCATCTGTAGATTCTGTACAAGATTCAACGACAGCTGAAGAAGATAGTGTCTGTAAAGGTTTGTAACCACGCACAGACAGACCATAACGGAGGTATATCCTGCTTGACGTCTATGGAAATGCAAATCTACTTCCTTAAAACCTGAAGTGTTTTGGAGCCACCTCCGCGCTTCCGGGGTACGGGCGTACGGCATCAGTGTCACCTGCGGACGAGTGGCGGGATCGGATCTAAAAAATTTATCCACTGGAGTTGTGGGAGATGTCGTGTCTGGTGCCCTCTCCCTCCGTCCCGTGCTGCACTGCGCTTTCTTCTTTGTGATTGCTTCGCTCGCCTCCTCCACCACCAGACACCAGCCCAAACTACCTCGCCAGCTCCCTCTGCCTCCCCCTCCCTCCTCCCGTCGCCGCAGCACGCAGCTCCGCCGGTTTTCACGGCTTGGAGCAGATCTGCCACGCCACCGCCGCGTAGACCAACTTGGTGGAGGGATCCCGTCCGAATCTGATTCCTATTCTCCAGCTCGACCGCTGTAGCGAGAGGAGAGAGAGAAAGGAGCAGTGGCGACGAGGTCTATGGCATCGATGGCAGCGTACCTGGCCTGGCTCGGCGGGCGGCGCAGAAGAAGTGGGTGCACCTGCTTTACCACACGGCGCTCAACTTCCACCGCCACCTCTTCTACCAGGAAATCAGTTCCCCCAATCCTCCCCTCTTCGCCTCCCTTTTGATCTCGATCTGCCTTCCTGCTCGCGGACGGGACTGGCCTTGTGTTGTCTCTCCCGCGCAGGCGTCAGATCTCCTCGACAAGTTTGAGACCAACTGCCACGTGGTAAGGCCTCCTCCTGCTGATCCCTTTTCGCACACCTCCTCTTCCCATTTGTCTGTTAGTGGGGAGTGAATTCATGAGTTACATTTGGTGATGATGGTCTCTATGTGGTGCCGTGATTGTGAGCTAGAACAAGTTTTTTCTATCATGTTTAATTGTTAAAATGCCTCCGTTGAGACGCCAAGACTCAGCACCGTTGTGGTGTCGTCACATCCATCTCTAGCCTCCCGAGTCACCAGCGCTGCCCAAGTTAGCTGAGCTCCGTCTGTTCTGCTTTCGCGCCGCACAACACCATTGAGCTCCTCCCCGACGCCACGTCCCGAGGTCTGCCGAGCGGCCGGCTCTGCCACGCTGAGATGTGCCCTGTCCCCTGCCTAGGTCGAGCGCCCAAGCCCTGCCACGTTCCGCCCCTACCGAGTACCGAAACAGATGAGGCAATGGCTAACTCGTATGGTGAATTGGAGGTTATGGCTCTGTGTCCGACGGAGGAGAGAGAGATAGGGGATGCGGCTTCACATCGCATTAAGGGATGTGGTGCTTACTGCTGAGTCATACCCGAGAGAGATGAGCGTGAGTGCTGCTTCTGTAATATACAAAAGCCAAGCAAAAAATTGTCTGAGACACACACATACACAGAGATAGAGAGGGGGAGGGAAAAGCTGAATAAAAATCTAATTGATGCGTGTGTGTCTGGATTTTGAGCAAGTCTTTATTTCACTAGACTCTTTTTTATTTCACTTGTTTAACAAACTTTTTACTTTATTTTGTGATTATTAACTCATTTTCTCTTTCTTAGATTTTATTCGCTATTATGAATAAGATTTTTTTTAATTTCCTTCACTTGTTTTGTAGCTATGTTTAGACTGTGTTGTAAATTTTTTTCATTGTTGCGTTATCAACAGCTAAATTTTGGCCTGTCCTCCGCCACTGCTCCTGTCTCCTCCTAGGATCCCACTCGTCCCAAAAACCTAGCCGCCGCCTCCATCCCCGTCCCAGAACCCCGAAAATGGGCATGTGTTGAACCTGGCCATCCAGCAACAAAAAAGTGACCCTCTTCTCGAGACCGTTGAAGGCATTGATCATACAATTATACTTATGCATGTGAGAACGATGATTATGATGTCTTGGATGAATCATTGTGTTCTGGATTTTTTTTGGTTTTGTATTTTGTTACTGACTGCTTCAGCTGCCTCCTATGATTCTCAATTTGCATAACTAATTGAGGAGCATCAGGTCTTTAAAAGAATGTAAGACAAGAATTCAAAGGCCTATTTTCGGTGTTGATTTCAAATCAAACCATTAGTTTTTACCATAGTACCATAATTGCCTTTAGGTTGATTCCTAATAGATTGGGGATTGTTTACTATAGATTGATTCCTAAACCCTAAACCATAAGCTTCAGCTGCCTCCTATGATTATCATAGATTGGGAAACCATAAGCTTGTGTTTGGAACTAATAGATTGATTCCTTGCATTGTTCAATATTCTTCTGGCTGTTCATTAGTAGTTGGGGATTGTTTACTATATATTATGTGTTCTTCAAGGAAGATTATAGTGCTAAGAATGTGCAGGAGTTCATATGTACTGAAACATAAGCTCATAAAGATTTATTCATTTTATGTGGTGATATTATTTTTTTGAAGTTGTTTAAACTATCTCCAGGTTTCTTTAGGGGTATTCACTGAAGGCGACAGCTGATCGTCATTCATAGATTGCAAGCAATAGGGGAAAGTCACTTCATCCAAATAATGGTCATTGCCTTTTCTTCAGAATGCTCTATTTACCTATTTGTAATTACTCCCCTCTCATCTGACATGTAGAGTATTTGACATATTCGTTGTATTAGAGAGTTTGACATGTTCGTTGTATAATATGATTTTATCGATCTTGATATATATTTGAATGGTTGAGCGCCCGATAGGGCGCTTTCTATTCT

>ZMCNGC3

TGTCTCCAAATTTAATTGTTTTCTTTTTCTTGGCTGGATTGAATTATTGTTGCAGACCTATCTACAATCAGCCTCGGGGCATATAGAGGAAATGAGAGTGCGAAGACGTGACATGGAGCAATGGATGTCATACAGACTACTTCCAGAGCATATCAAGGAACGAATACTGCGTCACCATCAATATCGGTGGCAAGAAACACAAGGCGTGGATGAAGAGGGCCTTCTTGTAAACCTTCCCAAGGACCTCAGGAGGGATATCAAGCGTCATCTTTGTCTGTCACTTCTCAAGAGGGTACTACCCTATACCGTACCTTAAGCGTTAGTTTAGAAACTCAGATTCTCCAATATCCCTGTTTTTTTCCTAGAGCAAGGAATCTTAAGAGGTAGATCCGTTCTTTTTTATTTGTCGCGTTTTAGTTCAAAAATGAACTAACCGACAACAAATATTCGAGAACGAACATAGTATCATTTAAGTAAGATATTTATGACTTGTTTGAGAGCTAGAGGATTGGAGAAGATTGAGAGCTAAAATCCTTGCTATTCAAAATATAAGCCTTTAGTTATTTATGGTAGGTTTTTTTTTCTCTCACGGTTTATCTCCCATTGCTTTTGAACTAGTCCTTAAGACATGTTTAGTAGCAACTGAATTGGAGGGAATGGAAAATGCTAAAATCCTTTCTATTCAATTTTGAATATTATGAGATTTTAGCTCTCTCAATCCCCTCCAACACTCTTATACTCAAAATAAACTATTAAGACTAGTTTGGAAACTCAAATCCCTTTTGGGATTGGAGGGCACCTCAATCCCCTCTAATCCCGAAAGAGATTTGAGGTTCCTAAAGTAGCCCTCAAGGGCTAGTGTAGAAACTTAGATCTCCTTCAGAATTAGAGGAGAAATTTTTACCTCAACCCCTTCAATTCAAAAGAGAATTTGAGTTTTCAAACTAGCCCACGCTTTATTTATTGACGTTCATAAGGAATTACTTTGCTCTTTGTACCATTCAATACGTGAGAGAAAGACTCGACCCTATCATGATTCCGATATGACTTACACGCCGTTTACAATGCAGGTTCCAATGTTCGAAAACATGGACGACCAACTCCTGGACGCAATGTGCGACCGTGTGAAACCCATGCTGTACACAGAAGGAAGCCACATCGTTCGCGAAGGCGACCCAGTGAACGAGATGTTCTTCATCATGAGAGGGAGGCTAGAGAGCACGACAACGGACGGGGGGCGAGCGGGCTTCTTCAACTCCAACGTCCTCGAAGGCGGCGACTTCTGCGGCGAGGAGCTCCTCACGTGGGCCCTGGACCCAGCCTCGGGCTCCAACCTTCCAAGCTCGACGAGGACGGCGAGGACGCTGTCGGAGGTGGAAGGCTTCTCCCTGAGGGCTCGCCACCTGAGGTTCGTGGCCAGCCAGTACAGGCGGCTCCACAGCAAGCAGCTCCGGCACACCTTCAGGTTCTACTCCCACCAGTGGCGGACCTGGGCCGCGTGCTTCGTACAGGCGGCTTGGCACAGGTACTGCCGGAGGAGGCTGGAGGAGGGCGTGCGCGAGAAGGAGAGGATGTTCCGGGCAGCGGCCGTGACCGACATCTCCAGCTCCCGCAGCCTCGGCGCCGCGCTCTACGCTGCCCACTTCGCTCGCAACATGGTAAGGACGCTGCGGAGGAACGCCGCACGGAAGGCCCGTCTGCTGGATACAGTGTCTTCGAGGCTGTTGCAGAAGCCAGCGGAACCCAACTTTTTCGCTGAAGAAGACTGACCTTTCTTTAATTTCTACCTTGATACTGAGATGTAATTGGAGCGGATAAGGAGGTATATATTTGTTTTTATATTTTCTCTTCGGCTAATTTCGTTAATTAATCTCGTGTTC

>ZMCNGC7

CCCGTTTTTACCTAGTCTCGACGAATACCTTAACGTTTGTGACCAGAAGCTGTGAGAGATCACCATGGCATCCGGTGCTTCACGAAATGTCAGGTGAGCGTCTTCCCGCGGGAACACCTCTTCTCTTATCTTCTATCTAGCTTACATGTTGCAACTCAGGATAACTTCCACTACAGTTTATATGGCCACTTTCAGATAACTGAAAAAGCCACGTTATTGACCAAAGGATTCATCAGGATCAATAATAATGTTTATAACGAAAGCGGCTTCTCCGCGAAATTTCAGGTTCCAGAACGAGATCGAGGTCCAGAGCTTCAGAACAAGCCCTCTGCAGAGCCTCAGCAGAAAGCACGGCAAGGCTCACGATCCCAGGAAATGCCGGCTGGGTTTCCGCGGCGGCTGCCTGGAGAAGGCGTGCCGGAACCGGAAGCCGATGCTGAAGGACAGGGTGCTCTCGCGCGCCTTCTCGGAGGAGCTGGAGTCCCTGATGCACGCCGCCGGCGGCAGCCACCTCTTCTTCGACCCGCGCGGGCAGCTGATCCACCTGTGGAACAAGATCTTCCTGTCCGCCTGCCTGCTGTCGCTGTTCGTGGACCCGCTGTTCCTGTACCTGACGGGCACGCAGCGGAACACGTGCGTCGAGTTCAAGGACTCGCTGGCGCTCACGCTGTCCATGGTCCGCTCGCTGCTGGACCTCTTCTACGCCGCGCACATCCTGTTCCGCTTCCGCACCGCCTTCATCGCGCCGTCGTCGCGCGTGTTCGGGCGGGGCGAGCTCGTCATCCAGCCCTACGAGATCGCCAGGAGGTACCTTGGCCGGACGTTCTGGTTCGATCTCGTCACGGCGCTGCCCTTGCCGCAGGTCAGCTCCCGCACGCAGCCATTGATGACGTGTGTCAGTTGCGAGACTGAACAGTGAGGCTTTTGGTTTGTCATGCATGCAGTTCGTGATCTGGATCGTTATACCAAGGCTGAATGAGTACTCCCGGACGGCGAACACGAAGAACATCCTCCGGTTCAGCATCATCTTCCAGTACCTCCCGCGGTTGTTCCAGATATTCCCGCTCTCGGGGCGGATCGTCATGGCGACGGGGGTCATGACGGAGACGGCGTGGGCCGGCGCCGCGTACAACCTGATCCTCTACATGCTCGCAAGCCACGTGCTTACTTCGACCGTATATCTTTTTGCGTTGATAGATAGATGCTGATTTTTTCTTTTCCTTTTTCTTCTTTGGACGAGCACAGGTGCTGGGAGCGCTGTGGTATCTCTTCTCCGTGCAGAGGCAGGAGGCATGCTGGAGGGAGGCGTGTCTGCTCGTGAGCCCGACGTCCCAGACCATGTTCTTCGACTGCAAGGCGTTGAGCAGCAACAGGACGATCTGGTATGAGCTGAGCAACATCACAACAAGCCGGTGCACGCCTGGCAACGGCTTCTACCCGTTCGGTATCTACGAGGAGGCGCTGTACGCCAAGCTCACGTCGTCGTCTTTCACCCAGAAGTACTTCTACTGCTTCTGGTGGGGACTCAAGAACCTCAGGTATGCATGAGCGCAGGAACTGGTGCGTATTTGGTAATAGTTACATGTCTCGGTGCAAACTTTGAAGCTCAGGCCAATTTAGTACTTGTGTTTTCAATAAATAAATGGAACAGCTCCTTAGGACAGAATCTGTCGACGAGCTTGTTCATCGGTGAAATAACCTTCGCGATCGTCGTCGGCGTTCTTGGGTTAGTGCTGTTTGGCCTGCTCATCGGCAACATGCAAGTAAGCCAGCAACAACAGAGGCTAAAATCCTACTGTCTGTTATAGGATCTCTGTGATCTAACATGACCCTGCCCTGTGTCCATGCACACAGTCTTACCTCCAAGCAACGATGGTGCGGCTGGAGGAGTGGCGGACGAAGCGGACGGACATGGAGCGGTGGATGCACCACCGGCAGATCCCCCAGCCGCTGAAGCAGTGCGTCCGGAGGTACCACCAGTACCAGTGGGTGGCCACGCGCGGCGTCGACGAGGAGGCCTTGCTGCAGGACCTCCCCATGGACATCCGCCGCGACATCAAGCGCCACCTCTGCCTCGACCTCGTCCGGAGGGTGCCCCTGTTCGACGAGATGGACGAGCGGATGCTGGACGCCATCTGCGAGCGGCTGAGGCCGGCGCTGTACACGCGCGGCACGCGGCTGATGCGGGAGCTGGACCCCGTCGACTCCATGCTCTTCATCATCCGGGGCTACCTCGACTCGTACACGACGCAGGGCGGCCGCTCCGGCTTCTTCAACTCGTGCCGCATCGGCGCCGGCGAGTTCTGCGGGGAGGAGCTCCTGACGTGGGCGCTCGACCCGCGCCCCGCGGCGAAGCTGCCGCTGTCCACCCGGACCGTGCGCGCCGTGTCCGAGGTCGAGGCGTTCGCGCTCGTGGCCGACGACCTCCGCTTCGTGGCGTCGCAGTTCCGCCGCCTGCACAGCGCGCGCATCCGCCACAGGTTCCGCTTCTACTCACACCAGTGGCGCACGTGGGCCGCGTGCTTCATCCAGGCCGCCTGGCGGCGATACAAGCGGCGCCGCGCGTCCATGGAGCTCAGGGTGCGCGAGGTGCGGGCCGGAGGGAGCTTGCTGCGGTCCCGCCGCCACAGCATCGAGGGCAAGGCGTCGATTAGGAAACCCATGGAACCGGACTTCACGGTGGAAGAAGAGGACTGATCAACGAATGATTACCTAGCTAGCAGCTTTTTTTTTCCTTTC

>ZMCNGC11

CTTGCCTCCATCCACTGGCACTGATCCACCACATCCCACGCCGAGCGCGACGCCAGCAGCAGGCTAGCTCCGCTGTCCGCTCCGCCGCTCACTAGCTGCTGGACCGCGCCTCCCTCCATCGCGCCGGCGCGCGCGCCTAACTAGCGGAGCCGCCTTTTCATGGTCCCCGCCGTGGTCGGGACGAGATGCCTCGCCTCGCATTCCTCCGCCGCTCCCTCCCCGCGAGGTCCAGTCATCACTGCGAGCCTTGGAGGAACCGAGTTCCTCGAGTTTGCGCCAATGGTTTCGACTTCCGATCGGACATCGGAGTGCCTATCCTGAACCGTGGTGTCTGTTGTTTGTTTGTTTATTTCATGGGCAAAGGCTTCTCGCGCGAGCGTGTGGTGGTGGTGGTGGTGGAGACCAGGGGAGCCCGGACCAGGTGGCGCGGGACGAGGAGGCCGGAGGCAGCGGCGGAATGAGCGGGCGGTCGTCGGCGGGTGGGCCGTCCGGCGGGGAGTGCTACGCGTGCACGCAGCCCGGGGTGCCGGCGTTCCACTCCACGACGTGCGACCAGGTGCACTCGCCGGACTGGGACGCCGACGCGGGGTCCTCGCTCGTGCCGGTCCAGGGCCAGGCGCAGGCGGCGGCGGCGGCGGCGGCGCCGCGGCAGCGGCACGCGGCGCGGTGGCTGTTGGGGCCCGTGCTGGACCCGCGGAGCAGGCGCGTGCAGCGCTGGAACCGCTGGATCCTGCTGGGCCGCGCCGCCGCGCTGGCGGTGGACCCGCTCTTCTTCTACGCGCTCTCCATCGGCCGCGCTGGCCAGCCCTGCCTCTACATGGACGCCGGGCTCGCCTCCGCGGTCACGGCGCTGCGGACCTGCGCCGACGTGGCGCACCTCGCGCACGTGCTCCTGCAGCTCCGCCTCGCCTACGTCTCCCGCGAGTCCCTCGTCGTCGGGTGCGGCAAGCTCGTGTGGGACGCCCGCGCCGTCGCCGCGCACTACGCACGCTCCGTCAAGGGCCTCTGCTTCGACCTCTTCGTCATCCTCCCCATCCCGCAGGTCAGCTGCCCTGCCGACGATGGCATCCGAACTCAGCGTACCGTTTTCTTGTCACCCAGACGCTGCTGTAGCACTGTAGGTGAGGGTCAGAGGGATCAAACGCCCCCCAAAATTGTGGACATGCCTATTGCCTCGTTCTGAGACAATTGCAGCGGTGTGTAGGGCACACGAGTACGTACGCTGCAGGTACGTACAACAGCGCCCAGCAGTGAGCTGACGTGTAATGCTGGGTGATGTTCCAGTACGCTGGCATTGGCACGCATGTGTGTGGCACAGTGGCAGCAGGGAGGCTGCAGTTTCTTGAAAGATCAAGGGGGGATCCCAAGACCATGACCCGCAGTGATTGCTGGGATGGAGGAGAATTCAGGTGGCGGTAAGAGTCTCAAGGGCTTACGGAACTAATGAAGGGTACGGATCGATAATGGCGTGACGCAATGATTGGCGGCGGTAGGATTTGGAGATATATATCACCCAATCATGCGGACTTGCGGAGGGGAAAAAGAAAAAGAAACAGCATCATGGTAGGACTAGTATTTGAAAATGACCATAGTTACACTGACTGGGATAACCAGCATTGATGGGGGAAAACTGGATAGAAAACGATAGTTCAGTCCTTCAGTGACGCATTGTTCACGGGAGGGCGGCGGCCTTTTGGCATGGTAGTAATAATTGTTACGCGACGAGTTTTCGAAGCGTAAAATTCCGGTTTGTCTCCCAGCATAATACGATATATTCTCGTTTTCATATCTGCCGCTGCTCGAGAATTAAGACTAGACAGACTCTAACGTGGCTTTCGTTCTAGGGACACTTGTCTGATTCCTGTTCATGCCATTGGATTCTCTCCTCCTAACTATTTATCAGCACGCAGTCCTTGTCGCTCAGATGGTTGCGTATTTTCTTCGAGTGACAGCTAGTTTTTTTTTTGTCGCATAATACAAGAATGCGTATTCGTTTTTGTTTCGCATGATTGCACCTATCAATTTTACGTGGCTGCGAGACGAGTGTGGACAGCGCTGATTTTGGACCGACCATGGACAGAAAATTGTTAATTCTGTCCTTTTCCAAATCAAGTCATTGATATTGGGTTTTTTTTTACTCCTTGCAAAGAGCTTTGCCATGGTATGGACCATTTATTTAATAAGACCGTATGGCTAGAATCATCTGTTACTTCATATAGCAGGAGGTAACATGTGAAATATGCATTCATTTTTTCAGAAAAATACTAGTCTTTTTTTCTATATATAATTGTTGCATGCGTTACCTATTTGACATGATACAAATTGACTAGCATAACTGCCATGACAACCAAAGTGCCTGCCATGGTAGTTTTATTAGCACGTAACTTTTTATTCCCACCGAGTACTCCCTCTATCCTAATTTATAATTAGTTTATTTTCTATCATAAGTCTAACTAACTCGTCTTATTAAAAAAATGTAGAAAAATAAAAAAAATCATTGTGGTTTTGTTTATTATCAAATACACTTTACATATGACTTGATTTTTTTTTCCCATGATTTTTTAATAAGATGAGTAGTCAAAGTTAGTATTGAAAAAGTCAAATAAATTATATTTTGAAATGGAGGGTGCAGCCTGTTCGGTTCCCAACGTCAATTAGCTCACGTGCAACATGTTTTAATCTAGATTCTCTTAATTATGCCATAGTTTCAATCGAATTAACAGTGGACACGTGTAACATAGTTTAGATCGCCAAAGTTCTAAGACTCAATTCACTTGTTTTTTAATTGATTATGTAACATGACTGGTCTATAATCCATCATAACTAGTTCCTTTTGTAGTTTTGTTAAAGGGTTTCTAACCACCCCGAGTTCCTTTCTCTACATTATTTATTAAGGTTCTAACTTATTATGAAGAATCATTTGGATTGTGATCTACTACCACCTCTAGTTGAACAACACCTTTATGAGAAAAATGGAATTGTATCGAGTGCTTTAATTATCACGCCAATCATCTGCATGAAGGACCTTCAAAGCGGAATTCAATTGTTCCTTTTACATTTTTGTATGTTCTTGAGGTTCCCATTTATTTCAGGGTTCACAAAGAAATAGTGTAGATTTGTTTGACTTTATAAGTTCCGTTTTCTCAACAAGACAGTCCATCAAATTCTGCAGGTTATCTTCTGGCTGGTTATACCAAAGTTAATCAGGGAGGAGCAGGTTAAGCTTATCATGACAATACTGCTGCTCATGTTCATATTTCAATTTCTCCCCAAGGTCTACCATAGTATACACATCATGAGGAAAATGCAGAAGGTGACAGGTTACATCTTTGGATCAATATGGTGGGGATTTGGTTTAAATCTATTTGCCTATTTCATTGCTTCTCATGTGAGTATTCCCTTCCTCATTAGTTAAATACTGTAGAAAATACAGGTTAAATCGTGTGGACATGGAATCCCAGTGTCGCTCTCCTAGCATTGATATGGTTGCTGATGGCTTAGTATTGTCACTGTTGTTGATCATAATATTGCTGAACATGGAAACTGCCCCCCAACAACTAAATCATAAGGATTGATTAAGAAACTGGGTTTCTATCTAATGTCCACCAATAAACATGTCCAGTGAGTTAAAATTGTAAAAGAACAACCTTCATTTTTTTTTTTTGCCTAAATAAGAATTCCTGTCTGCAATTTTTTCGGCTCATCCTTCAACCTATAGAATTGGTACTAGACTTAAGACTAATGCAGACCGAATAATCTGGGTAATTCGATCATTCATCAATGCTCTGCCATTTCCAGATTGCGGGTGGGTGCTGGTATGTTCTTGCGATCCAGCGCATCGCTTCCTGCCTCCAGGAAGAATGCAAGAGAAACAACAGTTGCGATCTAATATCGCTAGCTTGTTCCAAGGAGATATGTTTTCACCCTCCGTGGTCATCGAATGTTAACGGATTCGCGTGCGATACGAACATGACCTCCTTTAGCCAACAAAATGTGTCTACTTGCTTGAGTGGAAAAGGTTCCTTCGCTTATGGAATCTATTTGGGGGCTCTTCCTGTTATATCGAGCAATTCGCTCGCTGTCAAAATACTCTATCCTATATTTTGGGGCCTCATGACACTCAGGTAATCACTTTTCAGATTTAATTTAATCTGCCTTTAAAACACAGTGTATGCTCTCTAGGAAACTATTGCTTTAATAAAGTTACTATATGTTCAAAGTTTCTTTCTTTCTTATACTGTTTATCTGAATAAACAAAACATCAGACTGATACCCAGTTCCACTTCAAAAAAAAAATCTAGTCTTGTTCCATGCATATGCATACTTGCATTTTTTTAATGCTGTTTTCGTATTGTGTTTGAAGCTGCATTTTTCATGAACTTATACAATTCCTTGAATTGCTCGGCAGTACTTTTGGTAACGACCTTGCCCCAACAAGCAATGGCATTGAGGTGATATTCAGCATAATCAATGTCCTCAGTGGCCTTATGCTCTTCACGTTGCTGATCGGAAACATACAGGTAAAACGATGTCGTTTTTTTTTTGGCTCTTCTGTGTGATTGTACTTGTACCACACCGATTCACTAACCAGAAATTACAACTTACTGAATCTGAATCCGCAGGTATTTCTGCACGCGGTCCTGGCAAGGAAGCGGAAGATGCAGCTGCGGTTCCGGGACATGGAATGGTGGATGAGGCGGAGGCAACTACCGTCCCGGCTGAGGCAAAGGGTGCGCAAATACGAGCGCGAACGCTGGGCCGCCGTCACGGGAGACGAGGAGATGGAGATGATCAAGGATCTGCCTGAAGGGCTGAGGCGGGACATCAAGCGCTACCTGTGCCTTGAGCTAGTTAAGCAGGTAAAATCAAGAGCTTTTAGTAAATGTTCAATGTTTTTTTACATGATGAATGAGCCATGAGATTGCTAAAAAAAAATTAACTTGCCAGGTTCCACTGTTCCATGGCATGGACGACCTGATCCTGGACAACATCTGCGACCGGCTGCGGCCTCTGGTGTTGTCCAGCGGGGAGAAGGTGATCCGGGAGGGCGACCCCGTGCAGCGCATGGTGTTCATCCTGCAGGGCAAGCTCCGGAGCACGCAGCCGCTGACCAAAGGCGTGGTGGCGACGTGCATGCTGGGCGCGGGCAGCTTCCTGGGCGACGAGCTGCTGTCGTGGTGCCTGCGCCGCCCCTTCGTGGACCGGCTCCCCGCGTCGTCGGCCACGTTCGAGTGCGTGGAAGCGGCGCAGGCGTTCTGCCTCGGCGCGCCGGACCTGCGGTTCATCACCGAGCACTTCCGGTACAACTTCGCCAACGAGAAGCTCAAGCGCACGGCGCGGTACTACTCGTCCAACTGGCGGACGTGGGCCGCCGTCAACATCCAGCTCGCGTGGCGCAGGTACAGGGCCCGGACGTCGGCGGACCTGGCGGCGCCGCCGTTGGTGGGCGGGCCCGACGACGGGGACCGGCGGCTCAGACACTACGCGGCCATGTTCATGTCGCTACGGCCGCATGACCACCTAGAGTGAGCACTGATGAGCAGGAGGGAGGGACCATCCTAGGCTCCTAGCTGTGCCGGCCGCGTCATGGTGACTGTACAGTGTGCACTGTAATAGTATATTGCTGCCATCTTTTGCGTAAGTGAATTAGTGAATTGTCAATTGCCTTTTCCCCCACAAGAATCCGAACTCTGAAATACTTCTGTTGGCCTGTCTCTCCGCGTTCGTCAGGATATTGCTGCTTCTGACCGCGCCATTGCGGCCTCAGCTGCAGGCTGCAGCTGCTGATGAACAGAAGAGATGGCAGGACCGCAGGCCAGATTCAGGCGGAGCAGAGAACACAAGTCGGCGTTCAGGTTCTCCACCACCAGATTCAGAAACCGTCTGTTCGTCCTCTCCAAAACGATTAGAATCGATAAACGCGCTCGTAAATCGTAATAACCCTAAACCAAAGCGTGAGCGAGACGCGCGAGCGCCATCAGCGGCGAAGCTCGTACACCGGGACAACTCCACAATACGGACGGATTCCAAATGCGGACCGATAGAGATTATTACTAGTTTTGCTCCTACAATTGTGTTCCAGGTTGCTCTTCTGTTGATTAGACATGGTGCTGATGCTGATGCTGAGGACAAGAGGAAGGCTACACTGTCCTTGGCGGAGCATCGAACAGCCCAAGGCCTGCACTTGTCGACGCAGCCATGGCGATGCTTGAAGGTTGATCTAGTTCTCTGCTTTAAGCCTAAACTTTCATGTCCTATCAAGAGGAATGGAAAGTTTGATAGGAGAAACTGTTCTGAGTAGAAAGTGTTGGATTTCTATATTTAGATTGATTATTACTTTCCTTGTTTAGGCTTGCATGCCACCATTCTTGCTCACGTGCATGTCTATAGTTGGAACTTGGAAGGCACTAATCAGATGATATCCTTCCTATTCCACCGGACACTGTTTCCATGAGACGGTTGGGATACGGAAGGAGTCTGGCCTATATGTATTAGCTGTTTCTCTCTATGAATCATCAATATTCGTAG

>ZMCNGC5

GAAACGGTGGGCAACGACCAAAGAGGCACATTTTTTTGGCGGTTTCTCAACGTCGATTGATGCGGTGGTGATTTGTGAGAGACTGCGACCTCTCATCTCATTGTCAGGTGATGATGTTTCGTATGATGCATGCTTGTTTCAACATCCATCGATACCGTGCTTGTTTTATTTATGATTAAAAACAACAGTGCTTGTTTTAAAACTGAATTCTTGCCGTTCATCCGGCGAATTGCCCTTTTGGTTGTGCAGGAATTTGGTCTTCGACGCAGGAGAGACGAACGACGCACTCACGAACGAAGCGCGAGTGAGCATGGCAGGATTGCTGGCGATACAATGAATGAATCGAACTGATCCGCTGGAGGCGCCTCCTTCCCCTGCATTGCATTGCTTCCATCACGAGAGGAGGGCGACTGATTGATTTCCACACGTGGTGGGTGGCGGCGCGCTCCTAGGTTTCAATCTGACGACGACGACGGCGACGACACGAGCTCGCAAGATGTCGTACGACCAGTCGGCTTTCCAGGTCGACTACATGGGCGTCGGCGCCGGCGCCGGCGTCAGCGCGTCCCGGCGGCGGTTCATGCCTTCGGAGTCGCTGGCCCGCGGCGTCATCACGCACGGCTCGGCGCAGCTGCGCACCATCGGGCGGTCGCTCCGGGCCGGCGCCACCATGGCGGCCGTGTTCCAGGAGGACCTCAAGAACACCTCCCGGCGCATCTTCGACCCGCAGGACCCGGTGCTGGTGCGCCTCAACCGCGCCTTCTTCATCTCCTGCATCGTGGCCATCGCCGTGGACCCCATGTTCTTCTACCTGCCCATGGTCACCGACGAGGGCAACCTGTGCGTGGGCATCGACCGCTGGCTCGCCATCTCCACCACCGTCGTGCGCTGCGTGGTGGACCTCTTCTTCCTGGGCCGCATCGCGCTGCAGTTCCGCACCGCCTACATCAAGCCGTCCTCCAGGGTGTTCGGGCGCGGCGAGCTCGTGATCGACACCGCGCTCATCGCCCGCCGCTACATGCGCCGCTTCTTCTCCGCGGACCTCATGTCCGTGCTCCCGCTGCCCCAGGTGGTCATCTGGAAGTTCCTGCACCGGTCCAAGGGCACCGCCGTGCTGGACACCAAGAACAGCCTGCTCTTCATCGTCTTCATCCAGTACGTCCCGCGCGTGGTGCGCATCTACCCCATCTCCTCGGAGCTCAAGCGCACCAGCGGCGTCTTCGCCGAGACCGCCTACGCCGGCGCCGCCTACTACCTCCTCTGGTACATGCTGGCCAGCCACATCGTCGGCGCCTTCTGGTACCTGCTGTCCATCGAGCGGGTCAGCGACTGCTGGAGGAACGCGTGCGACGAGTTCCCCGGGTGCAACCAGATCTACATGTACTGCGGCAACGACCGGCAGCTGGGGTTCCTGGAGTGGCGCACCATCACCCGGCAGGTGATCAACGAGACGTGCGAGCCCAAGCGGGACGGCAGCATCCCCTTCAACTACGGCATATACTCGCCGGCCGTCGTGTCGGACGTGCTCAAGTCCAAGGACACCACCTCCAAGCTGCTCTTCTGCCTCTGGTGGGGGCTGGCCAACCTGAGCACCCTCGGGCAGGGGCTCAAGACCAGCATCTACACCGGGGAGGCGCTCTTCTCCATCGCGCTCGCCATCTTCGGCCTCATCCTCATGGCCATGCTCATCGGCAACATCCAGACCTATCTCCAGTCCCTCACCGTGCGCCTGGAGGAGATGCGCGTGAAGCAGCGCGACTCGGAGCAGTGGATGCACCACCGGCTGCTGCCGCCGGAGCTGCGCGAGCGCGTCCGCCGCTACGACCAGTACAAGTGGCTCAACACCCACGGCGTCGACGAGGAGGCGCTGGTGCAAAACCTGCCCAAGGACCTCCGCCGCGACATCAAGCGCCACCTCTGCCTCGGCCTCGTCCGCCGGGTGCCGCTCTTCGCCAACATGGACGAGCGCCTCCTGGACGCCATCTGCGAGCGCCTCAAGCCCAGCCTGTGCACGGAGCACACCTACATCACCCGGGAGGGCGACCCCGTCGACCAGATGGTCTTCATCATCCGCGGCAGCCTCGAGAGCATCACCACCGACGGCGGCCGCACGGGGTTCTACAACCGCAGCCTGCTCGAGGAGGGCGACTTCTGCGGGGAGGAGCTGCTCACGTGGGCGCTCGACCCCAAGGCCGGCGCCTGCCTGCCGTCGTCCACGCGCACCGTCATGGCGCTCTCGGAGGTGGAGGCCTTCGCGCTGCACGCCGAGGAGCTCAAGTTCGTGGCGGGGCAGTTCCGCCGGATGCACAGCAAGGCGGTGCAGCACACGTTCCGGTTCTACTCCCAGCAGTGGCGCACGTGGGCAGCCACCTACATCCAGGCGGCGTGGCGGCGGCACCTCAAGCGCAGAGCGGCCGAGCTGCGGCGCAGGGAGGACGAGGAGCTGGAGGAGGACGAAGGCAAGTCCAACAGAATCAGGACCACCATACTGGTGTCGCGGTTCGCAGCCAACGCTATGCGCGGCGTGCACCGGCAGCGCTCCAGGCGGGCAGTGGCCGTGTCCGAGCTGCTGATGCCCATGCCCAAGCCGCGTGAGCCCGACTTCGGCGACGACTACTAACGCCGAGTTTGGCGGGTGTATGTACTCTACTAGTTTTATACAGGTAGCAAG

>ZMCNGC12

AATCAATGCAGATTTGAACGTCCTTCCAAGGACGCCCAAACGATCTCTGTACCACGCCTGCACGGCTGCACCTACCCCACTTGGAACTGTGGACGCCTGCTCATCTTTTTTCATCAGTAGTACCATATGTCATCAGTCAGTCAGTCATGCATGCCGCGTGCGTGACCATTTCTGTGCGTTCGCAGGTGGTCGTCTGGGTGGCGACGCCGGCGATGATACGCGCCGGGTCGACGACCGACGTCATGATCGTGCTGCTGACGGCGTTCCTGCTGGAGTACCTGCCCAAGATCTACCACGCCGTCCGCGTCCTGCGCCGGATGCAGGGCGTCTCCGGCTACCTCTTCGGCACCATCTGGTGGGGGATCGCGCTCAACCTCATGGCCTACTTCGTCGCCGCTCACGTGAGTCGTCGTCCCTCGTCCGTCCTCCCTCACTAGTCTCTCTGCATAACGACTGGTGTGACCAACCAATTTGTCCTGAATGCACGGGCATCTTACCACTAATGGCCCTCGGCGAGAGACGACGCCATGATTCTGTGACAGCTACGCATGCATGATGCCCTTTGTTGGTTGTAGGCGGTGGGCGCGTGCTGGTACCTGCTCGGCGCGCAGCGGGCCACCAAGTGCCTCAGGGAGCAGTGCGCCCAGGCCGGGAGCGGGTGCGCGCCCTGGGCGCTGGCGTGCGCGGAGCCGCTCTACTACGGCGCCACCGCCAGCAGCGTGGGGGCGGCCAGGCTCGCCTGGGCCGGCAACGCCACGGCCAGGGGCACGTGCCTCGACAGCGCCGACAACTACCAGTACGGGGCCTACCAGTGGACTGTCATGCTGGTGGCCAACCCCAGCAGGGTCGAGAGGGTTCTGCTCCCCATCTTCTGGGGGCTAATGACTCTCAGGTCGCAGATCATCGCTTCATTAATCGTTGGATCGATTCTATGCGTTTCATGGTTTCAGTAACTTAATTGGCCTGTCTACTGATGGTGTGAGTTTGTTTCACCGCTTCTGCCGGCGCCGCTCAGCACCTTTGGGAATCTGGAGAGCACGACGGAGTGGCTGGAGATCGTGTTCAACATCGTCACCATCACCGGCGGGCTGATTCTCGTGACGATGCTCATAGGGAACATCAAGGTAATGACCAGTGCACGGTCGTCGTCTTGGCCGACGGTCGACGCATCATGCGAACGGCTTATGGTGAGCTATGGGCTATGGCTGGCGGCAGCAGGTGTTCCTGAACGCGACGACGTCGAAGAAGCAGGCGATGCACACGCGGCTGCGCAGCGTGGAGCTGTGGATGAAGCGCAAGGACCTGCCCAGGAGCTACCGGCACCGGGTGCGCCAGTACGAGCGGCAGCGGTGGGCGGCCACGCGCGGCGTCGACGAGTGCCGCATCGTCCGTGACCTTCCGGAGGGGCTCCGCCGGGACATCAAGTACCACCTCTGCCTCGGCCTCGTGCGCCAGGTACGCGCGCCGTGTCGTGGCGTGACGTCCCGTAGCGCCCGTAACTGATGCGGCCGCACAACCGCGTGCAGGTGCCGCTGTTCCAACACATGGACGACCTGGTGCTCGAGAACATCTGCGACAGGGTCAAGTCCCTCATTTTCCCCAAAGGAGAAGTTGTAAGCTCAACCACCTCCAACATGCTGGTTTTCTTCTACCGTAACCGAGATTGATCTCTCGTTTATGACTCAAGATTGTCAGAGAAGGGGACCCAGTGAAGAGGATGCTGTTCATCGTGCGTGGCCACCTGCAGAGCAGCCAGGTGCTCCGCAACGGCGCCGAGAGCTGCTGCATGCTGGGGCCGGGCAACTTCAGCGGCGACGAGCTCCTGTCGTGGTGCCTGCGCCGGCCGTTCCTGGAGCGGCTGCCGGGGTCGTCTTCCACGCTGGCCACGCTGGAGAGCACGGAGGCCTTCGGGCTGGACGCCGCGGACGTCAAGTACGTCACGCAGCACTTCCGGTACACCTTCACCAACGACAAGGTGCGGCGCAGCGCGCGCTACTACTCGCCCGGGTGGCGCACGTGGGCGGCCGTGGCGGTGCAGCTCGCGTGGCGCCGCTACAAGCACCGCAAGACGCTCGCGTCGCTGTCGTTCATCCGCCCGCGCCGCCCGCTCTCGCGGTGCTCGTCGCTCGGCGAGGAGAAGCTACGCCTCTATACCGCGCTGCTCACGTCGCCCAAGCCCAACCAGGACGACCTGCTGTGACTGAGATGCATGCCTGCAGTCTGAACAACGCGCGTCTGTTCAGCGGAGTTGAAGCAAAATGCAGCGAGATGGAAGAAGCTGGTTCTCTATAGTAAAAGTGCATGTCAGAAAGAACTTGTTGGGCCGTGTCCCTCCGTGTATGTAGAGCCCAAGAGACATGTATATTTATGTGTACTGTATCAGCTGCAATAGAGAGGGTTCTAGAGACTTCAAAA

>ZMCNGC2

TATGTGTGTTTTTTACTCTTGACAATGTTTCACCCCTATTTGCAGACCTATTTGCAGTCAGCCTCTTTGAGAGTAGAAGAAATGAGAGTGAAAAGCCGTGACACTGATCAGTGGATGTCATATCGACATCTTCCTGAGAACCTCAAGGAAAGAATACGGCGTTATGAACAATATAGATGGCAAGAAACAAGCGGGGTTGATGAAGAGCAACTCCTTATGAACCTCCCCAAAGATCTTAGGAGGGATATAAAACGACATCTTTGTTTGAAACTTCTCATGAGGGCATGTTCTCATCTCCCCCTTTTATAGTACCTTGAGTTCAGCCTAGCAGAAACTTTTTTTTTATTTTGATGTATGTATTGATCTTCCTAATTTTTTTTACCTTGCTGTGTTCATCGTTTCCAGGTTCCACTGTTTGAAAATATGGACGAACAGCTTTTGGATGCCATGTGTGACTGCCTAAAGCCCATTCTGTACACAGAAGGTAGCTGCGTTATTCGCGAAGGAGATCCGGTGAACGAGATGCTCTTTGTCATGAGGGGAAACCTAATGAGCATGACGACGAATGGTGGAAGAACCGGCTTCTTTAACTCCGATGTTCTGAAGGCCGGAGATTTCTGCGGCGAAGAGCTCCTCACCTGGGCTCTTGACCCCACGTCAACATCGAGCCTCCCCAGCTCAACAAGGACGGTGAAGACGATGTCTGAAGTGGAAGCCTTTGCCTTGAGGGCTGAAGACTTGAGGTTTGTGGCCACCCAGTTCCGACGACTCCACAGCAAACAGCTCCAGCACACTTTCAGGTTCTACTCGCAGCAGTGGAGGACCTGGGCCGCCTGCTTCATCCAAGCTGCCTGGCACCGGTACTGCAGGAAGAAGATCGAGGATTCTTTGCGTGAGAAGGAGAAGAGGCTGCAGTTCGCGATTGCCAACGACAGCTCCACTTCGCTCAGCTTCATGGCAGCGCTGTATGCTTCGCGGTTCGCTGGGAATATGATACGGATCCTGAGGAGAAACGCCACGCGCAAGGCCAGGCTGCAGGAAAGAGTGCCCGCGAGACTGCTGCAGAAACCGGCTGAACCCAACTTTTCCGCAGAAGAGCAGTAGTATTTCGCTTCTCTGACTTCTCGTTTTGCAGATACAGATATGGGAAATTGTTTGCGTGACTTGCAAGCCCGAGGATGGCATGCCCAGATTAAAAAGAAGAAGAAGAAAAAGGGGAATGAAATCTCGATAGAATGACCACTGAGTGATAGTAATGTAGTGATTGATATACAGTACATTACTGTTCGTTTAAGAACGTCCAAGTGGCGTTTGTATGTGCTTGTGTAATAGCATAGTTAGATAGGAGTGATAGTAATGTAGTGATATACATTCATCCACCGAAGCTATGTGTGTATAGGGATCGACCATTTCCGTGCTTACACTCACGCACTGTAAACGCAGCTGGTGACTGCTAAGTGGCATCCATAAGTGCGCGCAAGAGATCAACAGAAACTGGCTAGGGTAGTTGGCATGTAGTGCTTGCACAGGAAGAAAGCGCTGAAATGGCTCCATGATTTCAAT

>ZMCNGC8

GGCATGTTTGGGAGCAAAGGAAATGAAGGGGATTGGAGGGGCTAAAATCCCCTTGTTATTTAAAATTGAATAGCAAGGGAATTTTAGCCCCTCCAATTCTCTCCATTTCCATTGCTCCCAAACAAGCCCTTAAACGGAAGGTAGCATGATGAACTAATGGTTTTGCCTATCGGGTGAAGATGGTCCGTGGAATCCTATGTATTATTCTTCTATTGTTACTACTTTGGTTCGAAACCACACTATTTCGACCCAAAATCATTGGTTTTTGTTGTATTTATATTTCTTGCGCTTTTGCTATTTTTCTAACTTTCTGTTTATCTTTAGGATCTTTCCCGACGAGAGACAAAATCAATCTAAGTCATTGTATCAAACTACACGGGCTGACAGATTTGGCGCAAATAGAATAGATCTGAAGAATCCTGAGAAGCTTAAGGTGTTAAATGAAAGCAACAAACCCTGGCACCAGCGTATTCTAGACCCTGGAAGTAATATTGTACTGAGATGGAACAGGGTGTACCTTGTGGCATGTTTGTTTGCTCTTTTTATAGATCCTTTTTTCTATTACCTTCCATTGATTAGACAAAATGGCAATGGATCTTCATGTGTTGCCAAGGACCAGGGACTGAGCATAAGAATCACTGTCCTACGATCACTTGCTGACTTATTTTACATGTTGAACATAGCAATCAAGTTTCATACTGCATATGTGGATCCAAAGTCCAGAGTCCTTGGAAAGGGAGAGCTTGTTGTGGATATTAAGAAGATTCAACGAAGATATATAAGAACTGATTTCTTTGTAGACATACTTGCAGCTGTGCCACTTCCACAGGTAAAGGCCTCACGAAGATTAGTGGCTGCAGCCCTTGTTGCATAATTTAATTGCTTATATTGCATTTTCTTATTTTACCATTTTTATTGGTACTACAATCACCTGTGGTATATGTTCAATAGATGCATATTATTTTGACTAAATTGATTGATTTGCGTCTATGTGGCGGTAAGATTCTCATGGTAAGCCTTTGGGTTGAGTCCATGTCATTATTTTCCTTAATATAAGTAAGTCATCCGACCTTCCTGGTGATGTTGCTATTTCCTATCCTAGGAAATTGTAGGCAGTAAACGTACAATTGCGACAACCACATTGTGACTTAAGGTGCACCCAGCTACCTTTTTACTCAGATGTATTCATGCCATATATCATCTGAATCTAATTGACATCCACTTTAGATCGTAAGAAAGAATTCAATGAAATTTATTGTAATAAGCTAATACTAGCACTGTTATTGATGCCTTATATGTATTTATATCCTGTTCTCTTGATTTTCCCCTTTTCATTAGTGGCTCATGTTGGGTTTCAACTCTAGCCTACCCAACATGGTTGGGACTAAAAGGTTTAGTGGTGGTATTGAAATTTAATCAGCTAGCTTAGCTGAATGCTTGTACATTAACTTCGCCTGTGTAGCTGAAATTTTTAAGGGGGTGTTTGGTTTGTGGGGACTAATTTTTAGTCCCTCCATTTTATTCTATTTTAGTCTGGCGTATTTGGCAATTTAGAGACTAAAATGGAATAAAATGGAGGGACTAAAAATTAGTCCCTAGAAACCAAACACCCCCTAAGTGATGATTGTTGCAAATACTGAATATGATGACTTGGTTTATTTCGTGCAAAAAAAATTTAGTTAAGAATTAATCTTTCTGGGTGATTTTGTTTTGCAGGTTACTGTGTGGTTAATTATGCCTGCGATAAAAAGCTCAGATTATAACATCCGGAACACTACATTTGCTCTCGTAATTGTAATTCAGTATGTCATAAGAATGTATCTCATCATCCCTTTAAGCAATCAGATTATCAAAGCTGTTGGAGTAGTTGCAAAGTCAGCTTGGGGGGGAGCAGCATACAATCTTCTTCTCTACATGCTTGCAAGCCATGTATGGTTATCCACTTTCAATTTCATTTACGAAATTCGTGGGGATGTTAATCCTAATTAAAATTTATTTCAGCTTCTGTTTGTTCCAAGGGACACACATGGTTTTATTTGCTATTGAGAATGGAGTCTTGTCTTCTCTTTATATGGAACCTAATATCTGTTTTCTGGTCATTTTTCTTGATTCGACAAATATATCCATTATCATGGAATGCTTTAGAATTCAGTCTTGTGTTCTGCACAAAGCAACAAAACTGTTTCAAGTAGACATGCATGTGAGCTCAATTGTGAGAAACAATGCTATCATCTTGGATCTTGTGGTTACACTTTTGTATGCGCGATACTAAAATTATGTTACTTATTACCTGTGTTCATTGCTGCAGATTACTGGTGCAATATATTACCTTCTCTCCATCGAACGGCAGATTACATGCTGGGATCAGCAGTGCGTTGCTGAGTACAATGATACACATTGCAACTTTAGTTTTATAAGCTGTGAGAATAATGGTTCTAATGATTATTCTGTGTGGGCAAATAAGACAAAAGTATTTGCCAACTGTGATGCCACGAATAGTAGTATATCATTTAACTACGGGATGTTTTCTAGTGCACTGAGTAAAGGTGCTGTATCATCTCCATTCCTTGAGAAGTATTTCTTTTGCCTATGGTGGGGCTTGCTGCAGCTTAGGTAGTGATCCACCACTATACTCTGTTACTTTGGGTTCCTTTGTATGTCTAATACAGTTACTGATGTTAGTGTTGTTCTGTGTCTGTTAACACCTTTTCAGTTCAAGCGGAAATCCTCTCGTGACAAGTGCATTTATCACAGAGAATGCATTTGCGATAGCAATTGGTGCTATCAGTCTCATACTCTTTGCTCAGTTGATTGGCAAAATGCAGGTAGTGTCATAAATTATCACTTCTGAACTACACTAAGAAGTTTTGGGGTCGATACTTTACTTTTCATGCTTGTATTACCAGTTAACTCAGCATTAGTAATTGCATTATGTGATAAATTTGGAAGCAATTTAAAAGCTGAACTAGTTTTGCAAAATTACCTTGTTATCAGTTCTATTGTGCATTCTGTGTTAGGCTATTGTCACTTTCTGTCTTTTTTTTAACGGATGCTTCAACTTCAAGCATATAAAGTATGAACTTCCATTTCATGTTTTGAGATTTCTGATAAAAATAAGGATATTGCTGTTCCTGTGTTCAGCAAGCCATTTTCCACACTGCTAACCCACTTGAAGTCTGATTCAGAGATCACTTCCTATTCTTCTTCTTGTAGACATACCTGCAGTCTATCAGTAAAAGGCTTGAAGAGTGGAGGCTGAGGCAAAGGGACATGGATGAGTGGATGAGACACCATCAACTCCCATCTCATCTTCAAGAACGTGTGCGGCGGTTCGTTCAAGTCAAATGGCTTGCTACAAGAGGAGTAGAAGAAGAATCCATCTTGCAAGCTTTGCCTGCTGATATTCGTCGGGATGTGCAGCGTCATCTTTGTTTGGACCTCGTTAGACGTGTAAGTTGTGGTTATATAACCTAGCCCCCATCTCGCAGATGATAAGTAGAGCTTTTAGTTTTCGGAAGCTTCTTGATGGTAATAAACAAGTTGACGCTCTGTTGCACCATTGCTTTTTGATTCCATTCTGCATTTCTGCAATGCAGGTACCTTTTTTCTCTGAGATGGATAACCAACTTCTCGATGCCATCTGTGAGCGGCTGGTGTCTTTCCTGTGCCCTGAGAACACGTACATCTCTCGCGAGGGTGATCCTGTGAACGAGATGCTCTTCATTATACGCGGGAAACTAGAGAGCTCAACGACAAATGGTGGCCGCAGCAACTTCTTCAACTCCATCATCCTGCGCCCCGGCGATTTCGCAGGCGAGGAGCTGCTCACGTGGGCCCTGCTCCCCAAGACCAACGTCCACTTCCCGCTCTCGACAAGGACCGTACGGAGCCACACAGAGGTGGAGGCCTTCGCTCTGCGGGCTGAGGACCTGAAGTTCGTCGCGAACCAGTTCCGTAGGCTCCACAGCAAGAAGCTCCAGCACACGTTCCGGTTCTACTCCCACCACTGGAGGACCTGGGCCGCCTGCTTCATCCAGGCCGCTTGGCGGCAGCACCAGAGGAGGAAGCTGGCCGAGAGCCTCAGCCGCTGGGAGTCGTACTCGTGGTGGTCGGCGGAGGACCACCCAACCGGCGATAAGCCGAGGCAGGAGGGCACCTCGAGCGGCGGCGGCGGCACGAGGACGATCGCTGAAGGTGCCATCGCCCATATGCACAAGCTCGCCTCTGCTTCCAGAAGGTTCCGCACCGAGGACGTCGCTATCCGCAGGCTGCAGAAGCCTGACGAGCCCGATTTCTCCGCGGACCATTTTGATTGAACCTTCTTAATATTGTGCTTCGCAGCTGGGAGTTGCATGTTGTACGAACGATCGCAGCCGTAGCATATATACTCTACTGTAATTTTACTGCACAGCCATCTTAGGTGTCGTGAATTGTAATAGAAAAAGAAGATGTATCGTGTAATTCTAAATCCAGAATCTACCCTGTATAGGCTTAACCTTTTGTGCAGTGCATAAACTTGAGTATTTCGAAGCGTCATATGTGACTTGTT

>ZMCNGC9

TGATACTGCTAGTAGTTCCTAAAGTTGGGTTATCTGCTGCAAACTATGCTAAGAATTTATTGCGTGTCACTGTTCTTCTTCAATATGTCCCCCGTATCATCAGATTCGTACCACTTCTTGATGGTCAGTCCACCAATGGATTCATATTTGAGTCAGCATGGGCTAATTTTGTGATCAACCTTCTAATGTTTATTTTGGCGGGACATGTGGTTGGTTCATGTTGGTATCTCTTTGGCTTACAGGTTAGTTGATTTTTTTGTAAGATTATTTGCAAAAATTGGTAGTGCACATTGGTTCAAATGTTCCAATGTGGATAATCGTACAAACATATCTAGTTTTTTGACGTGTTTCAATAATGAGTAATATATGCTTTACCTACTTAAATTGGAAAATGTTCAGATCCAAATACCTATGTACTATGTTAATCTGTCTGTGACATGTAACATGTTGAATTTATATATTCAATCACAAACCTATCATTTTCTTTTGTTTACCGTTTTGTCACAGAGGGTTAACCAATGTCTACGAGATGCTTGTTCTATATCGACCATTCCATATTGTGATTCTTTTATAGACTGTGGACGTGGCATTGGGAGTGGACTGTACAGACAGCAGTGGTTCAATGACTCGGGTGCAGAAGCTTGTTTTAACACTGGAAATGATGCTACTTTCCAATATGGAATTTATGAGCAGGCTGTTTTGCTCACTACAGAAGACAGTGCTGTAAAACGATATATATATTCATTATTTTGGGGGTTTCAGGTATTTGCCATCATTTGCTTGTGCCTTTCCACTTCTGTAGGTGTCTCTTTGTTGCATGTCAAATATGTGACAATCAGATTGTCATGTATGATGTTTTAGGTCGTTACTATCTAATGAGGCATGCTAACTGATGAGGTTTCATTTTATTTAAATTCAATCTAACTAGTGTTTTTACTCATAGTTCTTGGTTTGATTTCAGCAAATAAGTACCTTAGCAGGAAACCTTGTCCCGAGTTACTTTATATGGGAAGTTCTGTTCACGATGGCTATTATTGGTCTGGGACTGTTGCTTTTTGCATTGCTTATTGGAAACATGCAAAATTTTCTGCAAGCTCTTGGAAGAAGGTATGTGGTTATACTACTGCCTTATATACCATGCTACTCGCACCAAATGTAAATATAAGTTCATCTAGGTTTGTTCTAGGTCAAACCTTGTTTTACCATTGAGTATCAATACCTAAAATTTTACTATATAGATCGACAAAACAAGATTAACAGTTCTAGATTCATTCTGAACAGTACTTCTACTCCCTCCGTCTCAGAATATAAGGCGTGCTCTCTCTATGCACACGTACATCGATGCAGTGGACAGTGGTATAGAGATAATTAAATGTATTTCTTGGTCTTTGATCCAGAGGTGGTTACACCTTATATACTGGGACAGAGGGAGTATAATGTAAGCTATTCTCATTTAAAATGAGCTATTTTTGTAGATGTTGGTAGAAGTGTTGTATTGAAGATCGTCATTTTTTCCTAATTTTGATTCGAAGTATATGCTTGTACAGTCATGTACTGCGACATGTTGTGTCCTGGGTACTGAATAAGGATGTCAATGAACCGGTCTCGAGCGAGCTTGGCACCACAAGCTTGAGTTGGACTAAATTTCTAGGCAAGCTCTATCAGAGCCTGAAATCTAGAGCTTATTATAGTAATGTTTGAGCTTGTGCTAAGATGTTCGGTTTGATCAAAGCTCGAATAAGGGTCAAGCTTGGTTTTAGCAGAGGCTTGATCGTAGTCAAGAAGATGGCATATTGACATAGACAGTCGTTGGTGTTTCCCAAAACTTTGGACACTTGCTGTGTTCCAGGAGGACAGCAGTGCCTCTGAGCATGCAGTGATGGGGATTCTTGAGGGATCAAATCATTTGTGCAGCTGGCCTGCCACTGTACTCAGTGCTCATGGAGTATCCCATGGAGTCGAACTTAGGACCTGAGGAGTGCTACTCAGACTACATAACCAACTCAGTTAGAGGCTCTTTCGCTGAGGGGGGTGGGGTGGGGGCAAGAAGGAGGCGGGGCTTAGAAAAGTTGAACTCTCTGGAGGGAAGTAGTAGCAAAAGGTGAGACAATTTTATTGCGCTGTATGTGCTAGCGAGCAGTTCAAATTGAGCTCATGAAGCTCGGTGAGCTTACCTTCAATGTTGAGCTTGGTTTGATTAGCAGACAAGCCAAAGCTCAAATTGAGCTTGTAGAGAAGATCTTCGAGCCCCTTTGACAGCCTAGCTCTAGGGGTGGTAATGGTCTCTAAATTTTACACTATAAAATTTAAGGTCGGACTTTATTTCTATTCACTTTTGAACTAAAATTAGTTAAGGGCTCAAACAAATTGTGAAGAGATATTTGGGTCGTGATCCATTATCACCCCTACCTAGCACTGAATGACTGAGTACAAGCTACATGCAGTTTTTGAGGGAATTGGGTAGAGTTTAGATGGCGATTAGCATTCATTTGTGGTAGCATGAATAGCACAGATACAGCCATACATGAATTGAAGGAGCAATTGGAAGGAAGACGAAGCGCTGGTTGGTTTCTGTTCGCTCAGCTGCTGCTTGATGTCTTTGTAGCTCTCCACATTCCCAAGGCCCCTGTATACAAGCCCATTAAAGTGAAACGTGTGATGCCACACCCTTGGCTCAGGAAATCTGTTTAAGCCCCTGTATACAAGCCCATTAAAGTCATCAAACACCAGTAGTTCAATCAGTGTTCTTAAGGCGCCTAGGCGAGCAAGTGGACCAGACAGGCGCCTTAGCGCCTAGGCGGCGCCTAGGCGACGCCTAGGCGGGAATGGCGAGCAAGGCGACCAGTTTTGCCCGAGCGCCTGGACGCCTAGGCGTCACCTAGGCGACGCCTAGGCGACGCCTTAAGAACACTGAGTTCAATTCACTTAGCAGTAGTTTGAAGCGCAGTAGACACAGGAAAACACATGCGGTTGATTAACAGTTTTTGATGTGTGAACATTTCACTCCTTTTCTCTTGTGACAGGAGGTTGGAAATGCAACTCAGGCGCCGTGATGTTGAAAAGTGGATGAGCCATAGGCGATTGCCTGAAGATTTGAGAAGGTTCCTCTACAGAACTTACTTAGCAAATCTTTATCACACTTTTTCCTTCAAAAAACATAAACTTAGCTAGCATGTTTCAATGTCTAATGCAGCCATTTGCTATTATAATGACAAGCAGTAATGCCGATATGGTTGTGTTTCATGATTGACTAGGGCCCTTGTGACAACAATGATATTATACCACCCTTTGTACCAAATTATAGATTGTTTTGACTTTTCTAGGTTCATAACTTTTGCTATGCACTTAGATATACCATATGTCTAGAGTTATGTATCTAGAAATGCCGAAACAACCTATAAATTAGAACGGAGGGAGTACTTCATAAATCTACTTTGTAATCAAGAAAAAGGTGTATGCTATATTGTCAGATAGTTTGAATACGTAACAATTGTTTGAGTGGTTCAGAATTCACATTAGCAATTAGGGTTTCATATCCACAAGAGACACCATCTAGTATAGTGTTAATGAAGAAATTGAATGTGATTTGGAATGTTCTTTTGTGTATTGTAAATTTGTAATCACGGGGCTTGTTTGGTGGAGGTATTTAGAGACAAATCCTTAGCATTTAATTCAATCAATATTTAAGTATAGATACCAAAACCTTCTGATGGAACAACCTAACCCGAACAGGCAGTCATTTTTATTCTTAGTCGAAGATAACTACAGCTGTACCTTTTTTATATATGTAGGAGGGTTAGACGAGCCGAAAGGTTCACCTGGGCAGCTACTCAAGGAGTGAATGAAGAGGAGCTTTTGAGTAATTTACCTGAAGACATCCAAAGGGACATACGTCGCCACTTCTTTAGATTCCTTAATAAGGTTGGTTTGTGAGGTGCCTAATGTGACAACAAATATTATTTCAATACCTTGTGTTTCTACTCCCATCCTTTGTTTCAACTATGATTTTTGTATCTAGTTTTGTGCCTATGAAATTCTACTATTCTGAAATCTGCTACTGCTGTTTGGTTCTTAGCATTCACAACTGGTAAAGTGGTAAATGTTTTTTCGTGAACGCAGGAGAGCTGCACATCATTAAATTAAGAGAAGAAAACAGTCCAAAATGGACCAAAGTACAATACCAAAATAGACAAAACACAAGACCACCAACTCCTGCTGCGCACAAAACTGCAGCCTCCTTATGCGCCTGAAAACAGCAGTACGCAAAAGGGGACTAACAGCAAAAAGTGCCAGCAGACCACACACAACTGCTCTTCACTAATCACCCAAAAGAACCCTTGACCTAAAAATTGCAGCACCAGGACCCAAAGCCCCAAGATGTCTAGCACCGGCAAGCATCCAACACTCAACTTCATCCAAAAAAACTGTCTTTATTCCATTAATGGAAGGTCTGACACCATCAAAAACCGCCTTATTACGATGAAGCCATAAACACCGAGCGATAAAGTGGTAAATGTAACTGTTATTTAGGGAGAGAGAATGGAAAAGAACTTAATCTCCAGGGGAGAAGGGTCTGAATTGATGTGAGACCACAACCAAATGTACTGAGCTAAACTTACAGTTCAGCACTTCCAAATAAGCAATAGCACAAAATATTCTGTGCAAACCACCCATTCGGTGCAATTGTGAAAACCCCCTTGAAACACATAACTAAAAATTCCTTTTTTTTGGAAATGAACACATGTATAGATGTACATTGTTGTAAAAGCTGAGGTGCAAATTCAATGTACAAAAATTCATATGAAAATAACAACGTTCAGGTGCATGTGCACTAGAAGGCAAAGTTCCTGTGATTGTCTTCTAGCACGTACACTCCTGAAATTTGTTATTTTCATATCAATTTTCAGGAAAAGATTTTGAACGTTAGCTTTTGCGACAATATATACATCTATAGATGCACTATGGATGTGAGTCATTTCAAAACTTTGAAAACTTCTATGTATGTGTTTCAACGGGTTTTGATGATTGGACCAAAGCACCGAATAGTTTGTCATAGTGATGATAGAGACTGGAAAAGAAATTTCAGAAAAAATGTAGATATTTGTATTTTCTTCATGTTGTAGGGTTTGCCTCATTTGCATGTTTCTGCAATAAATGTTGTCTGGTAAAGGTAATTCTTACAAGTTTCAATTTAGAGTTGACTAATTTTTATCTTCTTATCTGCAGGTCCGATTATTCACCTTGATGGATTGGCCTATATTGGATGCAATATGTGACAAATTAAGACAAAACTTGTATATTAGTGGAAGTGACATTCTTTACCAAGGTGGCACTGTTGAAAAGATGGTCTTCATAGTGAGAGGGAAGCTGGAAAGCATCAGTGCAGATGGCAGCAAGGCTCCATTACATGATGGAGATGTATGTGGAGAGGAGCTCCTCACGTGGTACTTGGAACACTCTTCAGCGAACAGAGGTATGCAATTTCTGCCCTCCTGGCCGCTGTTTGTCTTTTAGTTTTGTAGTTGTCCCATCTCATGACATGAACTACAACTGTTTCAGATGGTGGGAAAATTAAATTCCAAGGTATGCGGTTGGTTGCTATACGTACAGTAAGATGTTTAACAAATGTTGAAGCTTTTGTACTCAGAGCAAGTGATCTGGAAGAAGTCACCTCGCAGTTTGCTCGATTCCTGCGTAATCCACGAGTGCAGGGAGCGATCAGGTAGGCACATATTGGTTTACATTATTATATTATTATCACATCCCTGCCCGCCAGAATAGTTAGGGCTGTTCTTATCATTGTTTTTGCTAATTCATGGCAAAATACGATATTTTTTTTGATAACCATGTATCACTGCGATTTAAGACTTGTTAAATTCAGTTCCTTCCAACTAACCTCTGTCCATAGGTATGAATCCCCCTACTGGCGAACCATTGCTGCAACTCGTATTCAGGTTGCATGGAGGTATCGTAAAAGGCGGCTGAAGCGAGCTGAGAAGTCGAGGTTGAGCGAAGAGACTTATGCCTCACTTGGATCTTGACACATGATTCTTTTCAGCGTAGACGGAGGGGATGATTGATCGCTACCTGACTAGGCACTAACTTTTCAGTTTCAGTTCAGCATCTAAAGCTTCTAGAGCTGTGAAATTTCTCCGAACATGTCTACTTGGTGGAACCTAACGGGGCAAATATTTTTGGCTTTGGTGTTCCGTGTACATAATAAGAGGAAAGTTTTTTTTTTC

>ZMCNGC1

TTTTTTAAAAAACGAGCCCGACCTTGCTCCAACTCCCTCGATTCCCTCTCCCTATTCGCGTTCCCCACTTCCGTTCGCCGCCGACACCGCGTGCGCTCTCTCCCCCCCTCCGTCTCTGTCTGCGACGACTGGGTGGGCTACCGCTGCCGCCCTACCCATGTGCTGAGGCTTCTGCCTACCTCTTCGCCGGCGAAGAGCGCCCAGCAGGTATTCCCACGTCCTCTCTTTCCTGTCTGCTATGCGAAACCGAGTTCTCAAACTTTAATTTTTGAAATTGACTTCTCAAACTCTAGGTTTAAGCTATTTGGCTGTTTGATTCCTACTAATTTCTAACTTTTTGCCAAAAATTATTGGGTGCACATGTATACACTCATGTCCCTTTACTAGATCCGTCCTCGCTTCCACGCTGCAGTTGAAGATTTGATCAGCTATCGCCTTCGATGCAGTTGAATATTTGATCAGCTATTGACTTCGCTGCTGGCCTGTTTTCTCCTTTGGAGACCAAAGAAGCCATGGCGGGCCGGGAGGAGAGATATGTGAGGTCAGTCTGGTCAGCCTTTTTTTTGAATCTGTTGTGAGAATTTCACTTCAGTGACACACGTTCAGCATGCGTCAAGAATATATTAGTGACTTGGTGTCTTATTTTTGAAAGAAAAACATCAATTGAGTGGAAATGCATGGTGCCAGATAGGGCATGTTTGGATATCAGGGCTAAAAATTACTCCACCTGTTTAAAATGGATTAACCATGGGCTAATTATAGGCTAATGAGGGCGGACGCTAACCACCAATTAGCCATCAGTAAATCTTATTAGCCTAAAATTAGCCCATGTTAAGCCTTTCTAATTGGTATTCGAAACTAGGGGCTAATTGTTAGCCCCTGGATCCAAACAGGCCCTCATTCTATGGTGTACAAAAAGTCTACAAATCCTAAGAGCCCCTTTGGCAGGGCTCTCCGAAGAGCATCTGTTGTGGCTCTGGTTCTGCCAAGAGCTTTACCGAATACCAGTGAACAAAACAGCTCCAGCCAGGGAGCTAGCAGAAGCCCTGGAAAAAAAGAACTACACGCATGGAGCTGGAAATTATGGCTTCCAACAGCTCTGTTTGTTTATCTGCTTCACAATGCATAAATTTTGAAAGAAAGCAATTTTCTTCCAAAATATGTACTGATTTTAAATCCATTTTTTTTGAATTTTTGAAAAAACCCCAACATTCTCTCCTTTAAAATTGGAACATTGGATTTTTTCTCTTGCTTGGAACATTTTTCTTTGTTTTCACACAAAGTGTGGTGCAATGTAGCAGGACGTTCTAGACATTATACACCTATTCATTCTAGAAAATACAAGAAAAGTCATTCTGGCAAGCATTTTCCAAAGAGCTTCAGCTCCACCAGAGAAGGTTCTTTTCTGAAGGAGCTAGAGCTGGAGCTGTTTTGGGAGGAATTGGAGCCCTGCCAAACGAGTCCTAAGTACTAATTTTGCTGTTATTTTTGTCATACACAGGTTTCATGACTGGAAATCAGAGCAATCTGTTTCTGTTATTTCAGATAGGGTAGTATCAGAAAAAGGGCATAACATCTTTGGCTTGTTAAAGGACAGAACAGCAGGAGCCTTTTCATTCCTGGGGAACTCTTCACATTCTGAAGCCCTAAACAAATTAGGCCTAGGGGAGAAGTCAAAAACAAAAGTTCTTGATCCTCAAGGGCCATTTTTGCAGAGATGGAACAAGATATTTGTGATATCATGTCTTTTTGCAGTTTTTGTGGACCCATTGTTCTTGTATGTCCCAGTAATTGATGGTGGCAACAACTGCCTGTACTTGGACAAGAAGTTAGAGACCACAGCAAGTATCCTGCGCTTTTTCACAGATATCTTCTATTTACTCCATATACTGTTTCAGTTCAGAACAGGCTTTATTGCTCCCTCTTCTAGAGTGTTCGGTCGGGGTGCCTTGGTTAAGGACACATTTGCAATAGCAAAGCGATATCTATCAACATTGTTCCTGGTGGATTTCTTAGCGGTTCTGCCCCTCCCTCAGGTACTTGATATGCCATCTACTCAGTGAACATTTCATAATGGAACTACTTCAATCTGATCACAACTGCTTGTATTTTTGGAAAATATCCAATCAAACTTTGAAACTTTGACCGCCAATAACTTTTATAATATTTAGTTTGAAATAACAAAAATAGTATTTGTATATATGCTTTAAAAATACTTAACAATGTCATGAATTTTGTTGTATTTTATGTATACACCCTCAGAAAATATTGTCAACAGTAAATATTGTAAACTGTCATCCAAAACACCAAGTACTTTTCTTGACTGAAGGAGTATCAAATGGCTTACAGTTGTTATGCAGTGTTTTGTATAAACATAATATATTAATACGCACACACTTCGGTTCTAGGTAATGCTTATACAAGAATGAAGGGCTATGGTAAATGTTAGCAATTTTAGGTTACTTTAGGGTGCGTTTGGTTGTAATGATAGAACAGGATGGGATATGACGTTCCTTAAATAATGTTGTTTGGTTCAGGGTGAGGGGTTGGGACAAAACTATCCCAGTGTTGTCCCTCAAAATTGGAGGGACGAGAGAGGACGCCAGAGGACGTCCCTGTCTTGCCTGTCCCCGGATGTCCCGCAACCAAACACATCCTCAATGAACTTCAATGCACCAAAGGCACATAACACATGCATGCATAAGCCATCGGTGCAACTGTGCTACAAAATGGAAATATAGAATAGGTAATTGACATTATTGTCTTTGAATTAATGCATTTCATAATTGCAAGGTTACACACATTAAAGAGTGCTGAGCATTGGAATTAACATGAGGATCATGTTTTTTGTGTGAGATTGAGAGGGAAGAGAAGAAATAATAGGTTGTGATGCAGCTAATGGGAGCTGTGCTGTCTGTCCACGAGGTCTTTATTGTTTGACCTGGACTTTCCTGAATTAATTAGCTTGGCATGGTTTCCAGTTGCTGCAGCCATGGCATGCACCACCTTATGGTGGAAATCTGCTGCCTAGAAAATGGCATCAGTGAGGGACTTTCATCTTTTGAATTATATAAGCTATGATATGTATAGCACACTCCAAAATTATCATGCTGTGTAGTGATCTAATGCTTATAATACCCAGTTACATGGTATGAACAATGATTTGAGCTCCTTGCAAACGTGCCTGAAAGGTAGATAAAATCATGGACATTCTGGTTGTGCTTTATCATGAACATTGTATGATCAATCCTTCGTTCTGCTAATGCCTGCTATCATGCTTTGCAGCCCACTTATTATTAGATTGAATTGATCTTTTATTTTCAGTTTAGATCTGTCCACTAAACTTGTAATTTCCTGTGATTTTTCATCATCAGGTGTTTGTGTTGGTGGTGCTGCCTAAGCTCCAAGGTCCTGAAATTATGAAGGCAAAAATTGTACTGTTGGTTATTATTATTTGTCAATATGTGCCTCGACTGCTCCGAATAATACCACTTTACCTTCAAATCACAAGATCTGCTGGCATACTTACAGAGACAGCATGGGCTGGTGCTGCTTTCAACCTTATAATTTATATGCTTGCCAGTCATGTAAGCCCTCTAAACGAATTATTAAACAGCGTCATCCTTGGTCTTCATTATATTGTAAACTCAAGAAAGGATGAACGATGTTATCCACGAGCACTAAATTGCTCTCCTGCTATAATCAGACAGGAGGATGAAAGTACCAATATACCCCTGCTCTTAATCTTTAAATTGGTTCCTGGTCCATATATTTGTAGTACCAGCCCACAATTCAAGTACCTTTTACTAGGTATGTTATGACCGACAGCCTAGCAGCCCTAATAAAATCAGTTAGTTTGTTTAGATGGATAGGGATAATGAGTTTGTTTAGATAGATAAGGATTTGGTTTGTTAAGATATTAGATAAGGACGACTGCTATCTCAGCCCTATAAATGTACCCCTTCAATCAATGAAAGGTTAAGCAACCAAATCATCTTGCTCGGCTTCCCCCTTTTCCTGTCTCTACTGTTCACCGAACAGTACCGCACCCCTTCCAGCCAGCAGCCCTAACCCTAGGACAACGGCGCCCAAACGTCGTGGTCCAGAGTCTCGCCCTTCTCCTCACCACTCATACCTTCGTAGAAGCTGCAACTCCAACAATCTGGTATCAGAGATGCCAGGCAACGACGACAACACCAGCGATGTCGCATCTGCTGCCGCGACCAATTCCGCCAACGCAGCGCTAGAGCAAAAGATCGACACCATGACGATGGCCCTTGTCGATCTCACCACCGCCATCACTCAAATGATCAGTAGGCCGCCAACACCACCACCGCCCTCAGCGTCCACCAGCCCTACTTCCATCCCATATGGCATGCTAGGTTATGGAGGGATACCTCCCTTGTCAAACCCCGCCGCGCCACCCATACCATCACCGCCGATCACCACCACCATAGCCGCTCCACTCCCATCCACCTCAACCAACCTTCCACCGCCAACCTTAGCCTTGCTGCCACCCACATCTACCATGCCCATACCCATCCATCAGATACCCTTCCCACACTCCCCATCACCAATCCCAGGCTTTTCCGAACCCCACCATACCGCTTACCCATCGCAGCAAAACTCTCCCAACCAGCACCACCACAACCTTCCCTTTCCCACATTCGACGACAAAGAGGATCCTGTTGGCCGGCTGTCCCGCCGTGAGAGCTTCTTCTACAATCAAGGGACGCCGGAGGTGGATAAAGTCTGGATGGCCACGTACTATCTCATGGGCACTGCACGGCTGTGGTCCGTCATGCTACGGCGCGACGAGCCCACACTACACTAGCCGCGCTTCAAGACGTTGTGCCAGTAGCGTTTTGGGCCTCCTTTGCGCACGGACACGTTGGGCGAGGTGGCAGCACCGATGTTCAAGCGCCTCACCCCAGCAGAGATGACGGAGCGACGCCGCCAAGGGCTGTGCTAAAACTGCGATGAGCCGTTTGTACGAGGCCATCACTGCCAACGTTTCTTCTACCTTGAGGTGACCGCCGACGACAACGGGGTGGCTGCGGCGGAAGATCCCCCACCCCCGTAATCATGCCTTCCAGCTCGAGGACGAGCTGTTTTTGGAGGCCGGGAGAGATGTTATGACCGACAGCCTAGCAGCCCTAATAAAATCAGTTAGTTTGTTTAGATGGATAGGGATAATGAGTTTGTTTAGATGGATAAGGATTTGGTTTATTAAGATATTAGATAAGGACGACTGCTATCTCAGCCCTATAAATGTACCCCTTCAATCAATGAAAGATTAAGCAAGCAAATCATCTTGCTCGGCTTCCCCCTTTTCCTGGCTCTACTGTTCACCGCGGGTACTGTGGTGAAGATATTAGATATTAGATAAGGACGACTGCTATCTGTGAACAGTACCGCACCCCTTCCAGCCAGCAGCCCTAACCCTAGGACAACGGCGCTCAAACGTCGTGGTCCAGAGCCTCGCCCTTCTCACCACCACTCATACCTTCTTAGAAGCCGCAGCTCCAACAAGGTACCTTTCATTTTAACTATTGGATCTGGGTAGTGGGTAATGAATGGCTATTATAATTCCCAAGCACCATAAAGATCTCTTAAATGAGATGAACAATCTCTAGATAAACTGCATTTTAATACCCATGACTCAATGTCTGTGGTGAAGATATCACAAACTGATGTTGTAACTACTTACAGGGCTTTGGAGCTCTTTGGTACATTCTTTCCATCCAGCGAGAAGACACCTGTTGGAGACAAGCATGTATCAATCAGACTGGCTGTGATCCTACATCTTTGTACTGCGGGTATCATTCACTTGCAAATAATTCTTTCTTACAAAATGCGTGCCCAACAAATAGCACTGCCAATCCAGACCCTATATTTGGAATCTTTCTACCAGCTCTCCAAAATGTTTCACAATCGACGAGTTTCTTTGAAAAACTATTCTATTGCTTTTGGTGGGGGCTACAAAATCTAAGGTTTGCTAAGAAAAAAACCCTTCCAGTTTTCTTCCTTCCAACGCACATTTATATATTGTTTGTTGGTTTATTTATTGCAGTTCCCTTGGCCAGAACATGAAAACAAGCACTAATACATTGGAGAATCTGTTTGCTGTTTTTGTCTCGACATCGGGTTTGGTTCTATTTGCACTACTTATTGGTAATGTGCAGGTACAGACACTATTTTGGCACTGTTGATATTTTCTTGTAATGTATGTTGTTCAATTAAGTGGTAACTAATTAGTATTAATAAGGCCAACATATGGAAATGAATCTTTGGATGTGCAACTGCAGTTCTAATCTGTTATGCAAAAATCTTACTGCATAATTTTGATGCATAGTCATGTGATTGTAGTCAAAATAATTGGATAAACGTGAAAATATAAGACGCAATCCTCTTGCGAGTTCGAGAAAAAACATGAAAATAGAACTGCCTATAGATTGAGCTCATTCATTTTAATATCACGCCATACTAGTAACAAATTTACAGGAACATTTTCTTTACATAATTCTCTGAAGTACAGTGCTCAAGTGTTCTGCACTAAATGCTTTTCCTTGATACACTGAAACTGAATTTGGAAAGGATCAGAAATCTTATTTTATTTATTTTTAAAGATAAGCTGTCGTTTATTACCTTCAATATTAATATTTTCCTCAAACAGTTAGTTTCATATATTATAGACCTGTATTCATAAATGTGGGGGTGGGGCTTATGCCCTGTACCAGGTCTGCAATAGTTACTAGTGCAGTTGACACAACAATCATCCCTCTAACTAGAACTGGTTCTGGCAAAACTTGGTTGTTTTGTAAAATAAAATAGGCTAACATACATGTTTAAATATTTATTTCTAAATGTATTGGTTAATCTTCTCATTGTAAGCCACAATTAATTAGCTATACTACTACATTTCCTTTATTTATCATCTCTGGTTTACCAAAATTTATCCTGCAGCGTACCATGCCATACCTGAAGTTCAGCATGTTTGAGAAAAATAAAAGACAACAGCCTCATTATTTCAGTTCGAGGCAGGAACTATTAGTGTAACCATTAATTACTTGGTTACTAACTAGTCAGCTAAGCTTTGTGCGTGCCATATTATATAAGTTTTTTTTGGTTGGTGATTGCAGACCTATTTACAGTCAGCTTCTGTGCGTATCGAAGAAATGAGAGTGAAAAGGCGTGATACAGAGCAGTGGATGGCACATAGGCTACTCCCTGAGAATCTAAAGGATCGGATTATGCGCCACGAACAATATAGGTGGCAAGAAACAAGAGGGGTTGACGAAGAGGGCCTTCTTAAAAATCTTCCAAAGGATCTTAGAAGAGAGATAAAGAGACATCTTTGTTTGTCACTTCTCATGAAGGTGCTTCCCTGAACCTCTATCTGATTTGTCTTCTCCTTGCTCATCTTGTGGTGGATGTCTGATTGGTTATGGATGCAGTAACAACACTCTCAAGATAATATTTATCTGCAGCATCCATGCAGACTGGATATTTTGACTTATTGCCATGCGTCCATAAGCTGATGATGTATAGATGCCTAGCCTTACTTGCCAAGGACCACAAGTTCTTGAAGTAGTAGGTGTTTTAAATCATTGGGCTTGTTCGCTTTGCTCTCAATCTATGTGGATTGAGTAGGTTTAAATCCTAAACAAGTCAAAATCTTTCATAATTTTTTCCAATCCCATCCAATCCACATGGGATAGGAATAACCGAACAAGACCTTAAATGGAAGCTCCTGGTTTCTTATGTTGTATGGTTTTGCTAACTGCTAAGCGTAGGATTAGCAGGTGCCAGGTGTGGTTCTGTTCTGGACTAGGCAGGCTCTTATCTGGGCTTTAGTACAACTCCTGGTTTCTTATGTTTGATGGCTCTTATTTGTGTCAGCAGGCCGGAATGTTTAATTGCCCCCATGTATTGTTTTGGACAGGCCTAATGGTAGTTTGAACTGTGTATGTGAGGTGCTTGTTCATGCCTTATGCAGAAATAATACTGTTATCATTTGACCGTCTTCATCCACTCCAACAGCACTCTGTCATGCTATATGGAGCTTGATTCAATTCTGATCGTGGAAGAGGAGGGAAAGCCCGATTAGATAATGTTCTTGGGTAATAGATATTGACAGAAGGAAATTCTGAGACTAGTTTCTTTGCTAGCCTATGCGGCATGGACGTTCACTTTCTCTTGAATCTCTGCAAAGCTAATTATACTTTACCATCTTCCTTTTTAAAAGGAAATTAAGATATTCTAGAAGGCTGCATAGGCTAGCAATTTTTAAGGTCTTGCTACAGAGCCTTCTTCATGGGGTCCATCTTCTTTGAGCCATGAAAAAGATTGTGGAAGACTTGGGCTCCCCCTAAATGCAAGTTTTTCCTTTGGTTAGCTATAAGGAATAAATGTTGGACTGCTGACAGGCTCCAAATGAGAGGATTGCAGTACTCGGTGTGTTGTTCCCTATGTGATCAGGTGCAGGAAACAATCCAACATATCCTCTGCAGTCTGTACCTGCAGTTTTACTAGACAATTTTGGCATCTCATTTTGTCGTCTATCGGGCTTGGTGACCTCACTCCGTTTGTTGCTGAACAATCCTTTGCTGAGTGGTGGGGGAAGGCTTCTAAAAAAGTGCAAAGGTGCAAGAGAAAGGGTTTCAATAGTGTCATAATTCTTGGGGCTTGGTGCTTATGTCTCACTCGTAATAAGGCTGTTTTTGATGGAGTGAGCCCTTCCATTAGCTCGATTAAAAGGTTGTTTTTAGATGAATTGATCAGTTGGAATAAGGCGGGAGCAAAGCATTTAGGCAGTCTGGGGCTTATTGTCACCTTAAATAGGGTCTAATAGGGCCTGTCAGCCCTCCCCTTTGTTTCACCTTTCTTGGGTGCTGGAGAGGAGGCTTGGGTGTTTGGTCTTGGTTTTTATTTTTGTTTTTCTTTTCTGCCCTCAGGGCGTTGTATTTCGGTCCACTTTGGACCTTTCCTCTCTTAATATAATGATGCACAGTTCTCCTGCACGTTCGAGAAAAAAAATCATCTAAATGCCAATTCACTTCAAAATGTGTCTGAATTGCCATATGATGTGTTCCAGGAAATTCCTGACATGAGCTTTTGATTTCATGACCATAGAAAGTTGAATGGCAGTTAATAACATTTGAAAGACTTCATTGAGCCGATAAATATCTGGATGGGATCTATACCTTTTATACTTGATTCAATATTATATCTTTCTCTTAATGTCATGTTTTAATGAACAGTTGCCGTAGCTTACTATGTTATTAGCATGGTTAACCTTTTGCTGCTGTTTGCAGGTTCCAATGTTTGAAAACATGGATGAACAGCTGTTGGATGCCATGTGTGATCGTCTAAAGCCTATGCTGTACACAGAAGGAAGCTGCATCATTCGCGAAGGTGATCCAGTGAATGAAATGCTCTTCATCATGAGAGGAACACTAGAGAGTACCACAACAAATGGTGGGCAAACTGGTTTCTTCAACTCTAATGTTCTAAAAGGTGGAGACTTCTGTGGTGAAGAGCTCCTCACGTGGGCCCTTGACCCCACTTCAGCTTCAAATCTTCCTGGCTCAACTAGGACAGTGAAGACGTTGTCTGAAGTCGAAGCTTTTGCTCTGAGGGCTGACGACTTGAAGTTTGTTGCCACACAATTTAGGAGGCTCCACAGCAAACAACTTCAGCATACCTTCCGGTTTTACTCACAGCAATGGAGGACCTGGGCTGCTTGCTTCATACAGGCAGCTTGGCACAGATACTGTAGGAAGAAGCTGGAAGAGGCTTTATATGAGAAGGAGAAGAGGTTACAAGCAGCAATTGTAAGTGATGGCACTACTTCGCTCAGTCTCGGTGCAGCGCTCTATGCTTCACGTTTTGCTGGCAACATGATGCGGATCTTACGGAGAAATGCCACCAGAAAGGCCCGTTTGCAGGAAAGAGTACCTGCAAGACTGTTGCAAAAGCCAGCAGAACCCAACTTCTTCGCTGAAGATAGCTGAACTTGTACCCTGTAGCAAGCAGGGATTCATGGTCCAACCGGTGAAGTTTGTGCAGAAGTTAAGATTGGATGCTGTAGATAGACAGATCAGGTGCTCTAAAGCCGTCTGGTTTAAATCACCTAATTCGCGAAAAGGCCTGGTCCGAGTTAGATTCTCCGTTGAAATAAATTTGAAGCTGACGTTAAAAAAAAAATCCGCTTTTGCTCCACGTTAAATTCTGGCTTCCATAGTGGTCGGACTAGGGTGCTGAGATAAGGGTATTGAGATTTTTTAGATCTGTGTGAAAAGATTTTCTGTAACGTAATATCTGAAGGCTGTCTTATCCTCTGTAGGTTATTTTAAAAAAAACACGTATATTTCGATTGTATACAGGTTTCATGTATTATAAACACTCTAATTTACCTTGTGCATTTGAAAAAAGAGAGAAGATGCTGTGAATATAATCGATTAATTGTTTGT

>ZMCNGC10

ATGCCTCCGCTCGCATTCCTCCGCCGCTACCTCCCCGCGAGGTTCATGCTCGCCTCCACTTCATCGCTCGAAGGAACCGAGTGTCTGTTATCGATCCTGAGTTGCGTCTGTCTGTTTGTTTCCCTGAGCAAAGGCTTCTCGCGCGAGCGTGCGATGGTGGAGTCCGGGGGAGCCCGGGCGTGGCGCGGGACGAGGAGGCCGGAGGCAGCGGCGGACTGAGCGGCCGGTCGGCGGGGGCGCCGTCCGGGGAGTGCTACGCGTGCACGCAGCCCGGGGTGCCGGCGTTCCACTCCACGGCCTGCGACCAGGTGCACTCGCCGGACTGGGACGCCGACGCGGGGTCCTCGCTGGTGCCGGTCCAGGCGCAGCAGCAGGCCCAGCCGGCGGCGGCGGCGGCGCAGCACGCGGCGCGGTGGCTGTTCGGGCCCGTGCTGGACCCGCGCAGCAAGCGCGTGCAGCGCTGGAACCGCTGGATCCTGCTCGGCCGCGCCGCCGCGCTGGCGCTGGACCCGCTCTTCTTCTACGCGCTCTCCATCGGCCGCGCCGGCCGGCCCTGCCTCTACTTGGACGCCGGCCTCGCCGCCGCGGTCACCGCGCTCCGGACCTGCGCCGACGTCGCGCACCTCGCGCACGTGCTCCTGCAGTTCCGCCTCGCCTACGTCTCCCGCGAGTCCCTCGTCGTCGGGTGCGGCAAGCTCGTCTGGGACGCCCGCGCCATCGCCGCGCACTACGCCCGCTCCGTCAAGGGCCTCTGCTTCGACCTCTTCGTCATCCTCCCCATCCCGCAGGTGAGTCAGCTGCCGACACTGGAATCCACCTGCCCCCCGAACTCACTCTACCATTTCCTTCTCACTGGAAAAAAACCCCCCCGGCAAATAAACGTTTCGAGCGTACACCCAGACGCTGTAGCACTGTAGGTGAGGGTCAGATGGATCAAACGCTCCCAAAATCATGGACATGCCCATTATCCCGTTCTGAGACAGCGCACAACGCCCTTCGCAGTCCTTGTCGGAACTGCAGCGGTGTGCAGGGCACACGACACGAGTACGTTGCAGGTACAACAGTGTCCAGCAGGGAGCTGATGTGTTAAAAAAATGCATGTTTCGGCCGCTGGAGAGTGACAGTGATGCTGGGTAATGTGCAATGCTGGCTGCGGTTTCTAGTGATTGCTGGGACGGAGAATTCAGGTGACGATAAAAGAGTCTGATGGGCTTCCGGAACGACTAGTACGGATCGATAATGGTGTGACGCAATGATTGGCGGCGGTAAGTATTACTTAAATATGACCATGGTTGCAATCATGCAGAGTGAAAAAAAAAGAAACAGCATCATTATAGTAGGACTAGTACTTGAAAATGACCATAGATACAGTGCGATAGAAAACTGGATAGAAAACAATACGTAGTAGTTCGGGACGCCGGACGCGTATCAGCAAAGTGCTCTTTGCTCATGGTAGGGTGGGCCTTTTGGCATCACAAGTGTGATATGATCCACCGGCCAACCAGATTCCTCATGGTGAGATGAGAGGGCATTTTCATTTCAGATCTGACACCGCTGGAGAGTTAGTAAGCCTAGACTCTTACGTGGCTTTTGTTCTAGCCTTCTAGGGAAACTTGTCTGATTTATGTTCATGTCATTGGATTCTTTCTCCTAACTATTCATAGTAGTCTTTGCCATTCAGATGGTCATGCGTAATTCCTTCAAGTGACAGCTAGTTTTTGTCGCATAATATAAGAATACATATTCGTTTTTGTTTCTCATGATTGCACCTATCAATTTTGTGTGGGGCATGGCTGCAAGGCGTGTGTGGACATGCTTATTTGGACCGGTCATGGGCAGAAATTGTTAACTCTATCCTTTCCACATCAATTAATGGGTTATATTATATTGTTTATCCTTACAAAAACAATTCAACTTATTATTGTTCTAAAGGGCCTAACTGATTGTTCAGGAGTCAACATGACTTGTTGTTTAGAGCTCAGCCATTCCCACATGTTCTTTTTCTTACACAAGAGTTTCAAGCCACACATGTTCCTTTCTCTACATTATTTTTCGACGCTTTCCTTACGCAGCGGCGGGTCCCGGATTCAAAACATATAGAGCGAAGCTTATTTATTTAAGTCAATTAACACAAGCATTACGCAATCAGCTATATAATTGGAAATTTTGAGAAGGGGGACTAACCGTGATTTGGCAGGAGCTGTGGATCCAGCTTTTACTTATGTTGCCACTGTCCCTACATATTTATCTTCAAAGAGAAAGAAAGAAGCCACCTCAATGTTATAAAAAAATTGATTAGTATACTATCATTACATTTTAGGCACCAGTTGAACAACACCTTTCTGAGAAAAAAAATGGAATTGTATTGCGGGCTTTAATTATCACACATGCCAATCATCTGCATGAAGGACACTGAAGGTGGAATATAACAGTTTTCTTTACGTTTTTGTATGTTCTCGAGATTTTCTTTTTATGTCAAGGTTCGCAAAGAAACATAGGGGGTGTTTGGTTTGAGGAATCACTCCATCCAAAATGTGGTGGTGCATCATGGGTACATTCTTCAAATTTGGTGGGATGACCTTATTCCTCATATTAGTACTAACTAACTAACTATAAGGAATGAGGTGATGATGGATCAACTCATTTCATTCCACAAACCAAACAAAAAAGTGAGAAATGAGAAGATGATAGACTAGCTCATTCCTCAAACCAAACAACCTAATATAGAATTGTTTTACTTTATAACTTCTGTTTTCTCAACAAGGCGCTCCATCCAATTCTGCAGGTCATCTTCTGGTTGGTTATACCAAAGTTAATTAGGGAAGAACGTGTTAGGCTTATCATGACGATACTGCTACTCATGTTCATATTTCAATTTCTCCCCAAGGTCTACCATAGTATACACATCATGAGGAAAATGCAGAAGGTGACGGGTTACATCTTTGGATCGATATGGTGGGGATTTGGTTTAAATCTATTTGCCTATTTCATTGCTTCTCATGTGAGTATTCCCTTCCTCAATAGTTATTTAAAATAATAAATTTAATCGTGTGGACATGGAATCCCAAAGTCGCTCTCATAGTATCATAGAGTGCATTGAGAGTTGTAATCGTTGATATCTGGTTGTTGATGACTTATTATTGTCAATCCTTTGTTTTTTTTAAAAAAAAAAAGAGGTGGGGGGATTGTTACAATTGCTACATATATTTCAGTAGCACAGCATATTGTTTAGGTGTACCAGACGCCACTTTTGTTTTTTCCTACATTTGTCCCCCCCCCCCCCCCCCCCAAAGTTAGTATTTGTTGATTTATCTAGCAAATATGAAGGTAATTATAAAGATGGATTCTTCCTCTCTCTCTCTCAATGATCATAATAGTGCTGAACATGAAACTGGTCAGACAGCTAAATATTAGAAGCGATTAAGAAACTGGATTTTGGTCTAATATGAACCAATAAACATATGCGGTGATTAACATTTGAAAAGAGTTTTAGCCTAAACAAGAATTATTGTCTGCAATTTTTTGGGTTTGCCCTTCATCATATAAAATTGGTATTAGACTTAAGACCAATGCGTACTGAATAAATTTGGTAAATTGATTATTAATCAACATACTGCCATTTCCAGATTGCAGGTGGGTGCTGGTATGTTCTTGCAATCCAGCGCATTGCTTCCTGCCTCCAGGAAGAATGCAAGAAAAACAATAGTTGTGATCTAATATCACTAGCTTGTTCGAAGGAGATATGCTTTCACCCTCCTTGGTCTTCGAATGTTAATGGGTTCGCATGTGATACGAACATGACCTCCTTTAGTCAACGAAATGTGTCTACTTGTTTAAGTGGTAAAGGGTCGTTTGCTTATGGAATCTATTTGGGGGCTCTTCCTGTTATATCGAGCAATTCGCTTGCTGTCAAAATTCTCTATCCTATATTTTGGGGACTCATGACACTCAGGTAATCACTTCTTAATAATCTGCTATATTCTCTCCAACACAGGGTCTGCTCTCTAGGAAACTATTGTCTTAATAATCTGCTATATTCTCAAAAGTCAAAATATTCTCTCTTTCTTATACTGTTTATCTGAATAAACATGGCATCAAACTGATACCCCAGTTCCACTTCAAAAAAAAATTAGTCTTGTTCCATGCTGCATACACATGTATTTTTTTATGCTGCATATTCATAAACTTGTACAATTCCTTGAATTGCTTGGCAGTACTTTTGGTAACGATCTTGCCCCAACAAGCAATGGTATTGAGGTGATATTCAGCATAATCAATGTCCTCAGTGGCCTGATGCTCTTCACATTGCTGATCGGAAACATACAGGTAAAACAATGCCAATTTTTTTTGTTCTTCTGTGTGATTGTATCACACCCCTTCACCAACTAAAAACCACAACTTACTGAATCCGCAGGTATTTCTGCACGCGGTCCTGGCAAGGAAGCGGAAGATGCAGCTGCGGTTCCGAGACATGGAATGGTGGATGAGACGGAGGCAGCTGCCGTCTCGGCTGAGGCAGAGGGTGCGCAAATATGAGCGCGAACGCTGGGCCGCCGTCACGGGAGACGAGGAGATGGAGATGATCAAGGATCTGCCTGAAGGACTGAGGCGGGACATCAAGCGCTACCTCTGCCTCGAGCTGGTTAAGCAGGTACAGTCATTAAAGTAACTGAAGTTCTTGGGATTTTCGCTGTCTTGTTTTTTTACATATGAATGTTCGATGCGGCGATGAAATTGTTACAATTTTTTCCCTTGCCACTTGCCAGGTTCCGCTGTTCCATGGCATGGACGATCTGATCCTGGATAACATCTGCGACCGGCTGCGGCCACTGGTGTTCTCCAGCGGGGAGAAGGTGATCCGAGAGGGCGACCCCGTGCAGCGCATGGTGTTCATCCTGCAGGGCAAGCTCCGGAGCACGCAGCCGCTGACCAAGGGCGTGGTGGCAACGTGCATGCTAGGGGCGGGCAACTTCCTAGGCGACGAGCTGCTGTCGTGGTGCCTGCGCCGCCCCTTCGTGGACCGGCTCCCCGCGTCGTCGGCCACGTTCGAGTGCGTGGAGGCGGCGCAGGCGTTCTGCCTCGACGCGCCGGACCTGCGGTTCATCACCGAGCACTTCCGCTACAAGTTCGCCAACGAGAAGCTCAGGCGCACGGCGCGGTACTACTCGTCCAACTGGCGGACGTGGGCCGCCGTCAACATCCAGCTCGCGTGGCGCAGGTATAGGGCCCGGGCATCGACGGACCTGGCGGCGATGGCCGCGCCGCCGTTGGCGGGCGGACCCGACGACGGGGACCGGCGGCTCAGACACTACGCGGCCATGTTCATGTCGCTCCGGCCGCATGACCACCTAGAGTGATCAGGAGGGGGGACGGGACCATCCTAGCTGTGCCGGCCGGGTCATGGTGTCTGTACAGTGTACACTAGTGGTATGTTGTTGTCATCTTCTGCGTGAGTGAACTGGTGGTTCGGGATTTGTCATTTAAAGAAGGTCAATAATGGAGAAATAGTTTCTTAGCCCGATTCAATTGTCTTCCTTTCACCAAGAATAAAAATTACTTCTGC

**2.1500 Up-stream sequence**

>ZMCNGC6

CGCCTCGCCCGACCCCAGGGCTCGGACTCGGGCTCAGCCCCGGAAGACGGCGAACTCCGCTCCGCCCGACCCAGGGCTCAGACTCGGGCTCAGCCCCGGAAGACGGCGAACTCCGCTCCGCCCGACCCAGGGCTCGGACTCGGGCTCAGCCCCGGAAGACGGCGAACTCCGCTCCGCCCGACCCAGGGCTCGGACTTGGGCTCAGCCCCAGAAGACGACGAACTCCGCTTCGCCCGACCCCAGGGCTCGGACTCAGCCCTGGCCTCAGCCGACGGTCTCCGCCTCGCCCGACCCAGGGGCTCGGACTCGACCACGGCCACGGAAGACAGACTCGACCTCGACCTCGGAGGAGCCTCCACATCACCCAACCTAGGGCGCGGACCGACCACGTCGACAGGAGGCGCCATCATTACCCTACCCCGAGCTGACTCAGACTACGGGGAACAAGACCGGCGTCCCATCTGGCTCGCTCCGCCAGATAGGCAATGATGGTGCCCCGCGCGCTCTGTGACGACGGCGGCTCTCGGCCCCCTTACGGAAGCAAGAGGACGTCAGCAAGGACTCGACAGCCCCAACAGCTGTCCTCTCGCCAGGCTCCAGCACTCCTCCGACGGCCACGACACCACACGAACCGGGTGCCAAACACCTCTCCGGCTGCCACGACGGCGTGTACTTAGGGCGCTAGCTCTCCTCCGCTAGACACGTAGCACTCTGCTACACTCCCCATTGTACACCTGGATCCTCTCCTTACGCCTATAAAAGGAAGGACCAGGGCCCTCTTACAGAGGGTTGGCCCGCGCGGAGAAGGACGGGACGGCGCTCGCGCAAGGCCGCTCGCTCCCACTCCCGCGTGGACGCTTGTATCCCCCTACTGCAAGCGCACCCGACTCAGGCACGGGGCTAACACGAAGGCCGCGGGATCCACTCTCTCACGTCCGCCTCTCTCCGGCTGCTTTCTTTCCCCCCTTCGCGTTCCGCCTCGCGTCGACCCATCTGGGCTGGGGCACGCGGCGACAATTTACTCGTCGGTCCAGGGACCCCCGGGGTTCAAAACACCGACAGGTATAATATCTTACTAGCACCGACTACGTGCGGGTATACTTGCAGGCACATAATTACATAAATATTATATATATATATACAATGTATAATGTACGATATGGGTAGTTCACGGGCACGAGAAAACATTCGTTGTATTGATAATTAAACCCGAACCCATGGCTATTGGTACGAATTCGCGTCCAAACTCACACCATATAAGATTTATATCTCTGGACACACAGACAAAATATGCCCGTTGCCATTTCTAATAGAGATGGTGCCACACATTTCCGACCAAAAAATAGGGACAACGCAATGGAAAAATAACATGATGAAGATTACTTAGGTTAGAGATATCATGGCACTTAAATGGATGTGAACGTCAAAAATGATCCGATCTAGTTTAAATTATTATTTTTTTTATAAATTTATGGGAATTATTGGGGATTGAAAGAATAT

>ZMCNGC4

GTGGCCGTCCTTGAGGACCTCGAGGCCCCCGACATCGTCCTGCAAGATGAGAGTGATGCAGCTGGCATCCTCGTGCTCGCTGAAGCCGATGCTCGTGCTCTCCTCCGCCGCCGCCGGGAAGTAGCGCCTCACCGTCATGAGGTCGAAGCTGCGGTCATCGTTGTAGTTCTTGAGGAAGCCCGGTGGGAGGCCCATGCACCCGTTCAGGATCTCTTGGATGAGCAATCCCAGTTCGGTGAACTTGCTGTAGCACTCCTCCACTGTCTCTCTGAAGATGAAAGATGGTTGCGTCAGTTGTTTCTTGCTAGATCACAGTATCAGGTGTTTCGTTAGTTGTATGACGAAACATTTAGCCCTCGAATCTTTTGAAATTTCTTACTAGATCAAAGCCGCTATTTGGAGTTTGGAGCCCCCTCCCCCCTAAGAATTTTACCCGATGTTATTCAATCAAAGATTCAAGAACTCATAAGGTCAGCGGGGACGGGATTTTGCCCCCGAACCCGAACTGACCTGAATCCAGCAGGACCAGCGGGGTACGCGTTGAGCCCGAGCTTCGGGTCAAATACCACCAGGTACTCGTTCTTGTCGGCCACGTGCGCCGGCTGCCGTCCGTAACCGCCTGGGAGAGGCACCTTGGACCCTTCGGCCGCCCGGACCTTGGCCTTCTCCTCGTTCGGCAGCGCGAAGAACGCGGCCGACAGCTCCAGCGCGCGCGCCATGAGCTCAACCGGCACGCGGTGGTTGACGACGCTGAAGAAGCCGTGCGTCTGGCATGCCTGGAGCACGGCCTCGCTGGCGCCGGCGATGCAGCCCTTGCCGTCTTCGGTGAAGAAAGGCGCCAGGTCCACCACGGGGAGCTCGCCGCTAGCCGCCATGTCTGTCGGCTTTCGCTTCTGTTCCCCGGTTGAAATGGCAGACTTCGATAGTAGTAAAGCCAATTAGCCAATGCCGTAAGAGCCTCGTCGTTGCGCATCGGGGCATCAATGGCTTGAGTCGGAAAAAAATGCGGTCCATCAACGTTGATTGTTTCGCCCTTTCGTTTTGACAAGAATATGAACCTTAGAACTTTTTCTGATTGCCTCACGCTCCAGTACACAACACACGCCAGACACATATTACCACATCCTATAAAGAGAAAATGCGCCTGGTACGACGGGGCCGTCCAGTTCAATGAGAAAAAGAGAACGAAGCTCCCTTGGCATCCCACCAGGTCCAGCCCACAGCTCCCTGACGGCCCACTTAGCCCCCATGCGCCGTCACGGGCCTCGACGTGCGCCGCCAGCCGCGGTCAATTTGCGGCTGCGCCTCTTGTCCAGTCGTCCCCGTCCCCGCGTTGAGCGGTTCACGGTCCGGACTCCGGAGTAACCGAAGGAAGGCAGACGGCAAAGACCTCGTTAGGCCCTGTTCGGTTGTTCCAGATTGGACCCCCGGATTAATTCCTAGCCGGATTACTTCTCTAATTTATATAGATTTTGATGAGCTAAAATGAATCCTAGCTCA

>ZMCNGC3

ACATTTAATGGATGGGTAATAGGATCATGGAATACTTGCCTTCTTCGGGGGGGGGGCGGAGGTTCGGGGTCCTCAAAAGTGGGGACCTCAGGACAATTGCAACACTCTGAATCTACTCGTGTACATACAACAAATATGGACACAAAATTAGATACAATACACTAAGCATAAAACATGGTTCAAAAATAGTCCTAGAATTATTCTATGCATTTTTACAATTTATTTGAGAATAAAAACCATATATTTGTGTTAAAAATGAATTTGTTATGAATTCAGTTTAAGCTATCAGGAGTATATTTCTAAATAACTATATCTTACAGGGTCTAAAAGTATATCAGCACTTGGATAGAACTAGAGTGGACTACGGGTTGATTTTAGCTAAATAGCGGGTCTCTTTTGAAAAGTTGGTGTTGTCAGTGCAACACTGAATAGTATCGGTGAACTAGTGTCATTTGATTACACGTGGGCGATCATGATTAAATGAAGGCATATGTGTGTGGGGATAGATATCCCCTAGGTCTCCATGGCCCAGATTTGATGCATGGGCTGTTAAAGGACCTCAATGGGCCGGGTAGATGGGCCTCGTAGGCTAATCAGCGGGACCCAACTAGAAGATGATGTGGAACTCAAAGACGTGGACATGAAGGCTTTGTAACTCCCACGCATGCAGAGGCAAAGATAAACCAACAAAGAATCAGACGCTTGCATGTTAGTTGGAGTCGGCTCTGTATTCAACCGTACTGTAACCCTAGTCTCGTCAGATATACAAGGTGAGTTAGGGACACATACAAAATGGTTGGATACTCTAATAGGCATAGCTAGAGGAAAGTTCTCATCCACTTTGTAACCTCAAACTTAAGAGCAATTCCATCTCAGTGGCACACACGATGTATGGTATTATGCATCTCAACGGCTCGAACTTGTATAGATGTTCGAGCCCTATTGTGCTTTGCATGAACCATCGAGCTAATTGTCGATCTCGCCAACCACAAAAGCACTACGTGAGACATCTCGTAGTGTGTTACTGGTGCTAAACATCGACAATGTGTGGGTGGCGCTCTGACGACGGTGATGAGCTATATGGCTCACCGGCGTAGGTTTTCTATAGCCAATAGCTTAAGGTAGCTATTCAAAAGCTAGAGCTCAACCAAACACACCTTGTTGATGCCGTTAATAGTAACTGATGCTGGTATCGGCTTGGCTATTATCAAACTCATCAAACCAGTATGGACTATTTAACCAACTTTGATCTTCGTCTGGATGAAATTCAAGTGTCATTTTTTATACAATACTGAACGAGCTAGCTATGAGAACCCATTTCTCTGTAAGAATGGCCATATAGATTTATCCTTAAAAAATGATTCCGTATGTTTTGAATTCGTTGTACTTATATGCTAGCAGTACGCAAGAGGCCATCATTTAACGAACAAGTCCGGACAACATGTTGAACTACATATATAACTAGTGTCCATGATGACGATGTTTGGCCTGATGATCAAA

>ZMCNGC7

CGCTTCATCGGCCTGGTCGTCGTGGTCACCGTTGTCCTCGCTGCTTACCGAAGATTGCCCCCCCAAACTCCCCTAAAATATAATATGTTTCCCGCTAACTAGGACAGCCGGTACATGCACAGTGGAACAGGGGACCATAACACATTGGGCGGGAAAAACCTAAACTAATCCACGTTGTCATGCAAATCATCAAAACGAACGACCTAAACCACTTAGATGAGTATTGTGCATGGTATTATTAAGGTTAGGGGGTTAAAGATCTGTTTTAAAGGACATTCTCTAATGTAGAACACCTAATCAAACAATAAGCAATTATGTAGACTTATTCCTGGTTTTTTATGACAGAAAGGTATTGGAAAGATGAAAGCCACTTGTAATGGAAATAGTATAAAAAATTTTCTTTTTCAAGGTGGGGTGTAGCGCGCCACCAGAAAATAAAAATCGTATAAAAATTGGAAAAAGTCTACATAGCCCTCAACCTTTAGAGTGTAGTCTACTTCACTATTCACATTTAAAACTGGTTGTTTAACCCCGTGAACTTTATATTACCAGACAAATAACCCTAGGGTAGTTTTTGTTGGTGGTTTTGATAACATGACATTGATTTAAGTGTCACCGGTTTTATCTATTTCAGTTATGTTATGTAGCAGTATGCAAAGTTTATATAATTTTGCACACAAATTGATAAATAAGATATTACATGTGCCCTATTCTTTTGGAATTTTAGAAGTACCGGTTTAACATTAAAAATAAAAATAAAACTACATGAATATAATTTAATATTTTTGTTCCTGATTCATTTATTATCTTCTGAAACTAAAATTTTTACTAGAGATAATTAACACTATTACTAAACTACTGCATTTTTGTTGATTTTATTAAACAATTTAAGTAACCTTTCTTCTAATTAATAACTAAAGTAATTTTACATTTGCTGTTTTAGAAACACTTAGCAAACATTAAACACAACCCTTCTCTTAGCTAAAATAAATTATACCGATATTTCTATAATATTTTGAGGACTTCAAATCTATGTTGATCAAGTAGATTGCATGTCGTCTCTGCGCAAAGAAAAGCGAAACGTGGGAGGATACGCTCGCTTATTCTGCTCTGAAGTAACTTCTCATCGACTCGCCTCAAAATTGCCCTCGCAATATGTTTGTATATCTCCTATTCCTCCAGCGGTCCAGCTCCATTAAACAAAATTATGTTTTGCTCCAACTGAAAATTTTGGAGTTACTAGGAATCGATCACTCAATATGAGTGGAGCTGGAGCATTACTTTATGTGCCCAAACAGAGGGGATCAGAAGTTACAGCATATCTGAAGAGCACCGTGCCTGTTGTTTGCTTGTCCATATGAAACTAGTGCTTTGGTGCCTTTAACTGTAATCACAAGATCCCCGTTTCAGTTAGTTGCAGATGGCCTTTTTAGTGGTCACTTTCTGTCCCATAGCTGAAGATCTATTGGCGCCGCGCCAGTTTCCACATCTATCGTAGCA

>ZMCNGC11

TGACAGCGAAACTCCTGGATAGAATCATCGCGCTGCTTCCGTAGGCCAACACACATGCTCACGAAGACCTGGAGAAGATAACAACATGATGGCATACGTTGTTGTATGTTCACATGCTCATGTGTCTCTGGCACTATCATAGGATGTTCCCGAGTCATTTGATGATCCGATCTAGGTTGCACCAGGGCATTCTTAGGGGAGTCAGAGTCGTTGACAGGACGAGTTCCAAGGTATCTTTCCACTCCTTCATCGAGAAGGGACTCGGTCACCATCTCGGTCCAAACGGACTTGGACTCGTCCTCGCGGAAGAGATCTCGTGCCGAGTCAGATTGGTACCATCTACAACAAGGTAGGGATGTTCCTATGGGCTCAAATAAAAAGTAGTGGTTATGTTAGGCATGTGGTTGGAGACATGGAGTTACTCTAAAACAATCATTTTGGCCTTCTGCAATGACTAACCAGTTGCTCATGCTTTGCTACGGTTGTTATATAATTATTATTTATTTCTAAACACTAAGGTATGTGTGGTTTGGTTGTTAGCCTCAAATTTCTGGAGCAAGGGGGTGCAGGTTTGAGTGCTTGGTTTACACTATTTTCTGCACTGTGTGGTACCTACGCGGGAGAACCAATTGGAGCGCGTGGGGCAGACCTAGAGTGTCAGTACAAAGGGGGAAGCCGTAGCACTAGGTACTCATATTAGGTTCTTAATGGGTGTGTTTGGTTGTCTGCATCTGCGCGTGCTTGCATCGCGGGATGCGGGTAGCTACTGTTTGGTTGCTGTGAGCCAGGCTATACGCGTGCGAGCTCTGTGTTTGGTTGCCTGCATGTAGATTTGGATCCAGACTCGCACGATCCTTAAAGCACCTTGGGCCAGGCTCACCAGATACGAGCAGATTGGTCGCATCTCCAGAGTCAGGCTCCAGTCATCCTGGCTCACACAACCAGGTTGGCGAGAGAAACCAACCAAACAGACCCAATATTGCCTTCTGCAGTGATTGTGAGCAACGTATCAACATTTTGTATTCTCTCTTTAAAAAGGCAGCGCAAAAATAAAAATGTAGAGCTGCAGTGCACTGACCATGTAAAAAAGTAAAAGTTTTCCGCTATATATATTAAAAAAAGATAATAGAAGCTTTCACAGATGATAACCATTTCAGGCCCAGGCAGGCTCCCGGTCGCCCCACCGTCCAGTCTGTGGACCTACCTAACCAATGCATGGACCTCGGGCTCAAGTCCCGCTGCAAAGCACCGCGGGCACCGGATCACGGTTGCCGCCTGATCGCGCCACCACAGAACGTTCGTTTCCCGTAGATCGACGTGCCGCTTAGCTATGGCTACGAGGCCCGCCCCACTACGAACAAACGCTCCCGTCAATCCGGTAGGTGTGTAGGCCGCGAAGCGAAGGGTATATCGGTGACGGTGCACGTACGCGCCACGTCGGCATGTCCTTGGGCCCCGGCACACTTGTACTTGTACCCTTCACCTCTCTCCTCTTTACTA

>ZMCNGC5

ACGTCGTCGATACGTGGCGAAGGAGGTGGCTTCGGCTTCGAAGGTTTTGGTGCACGAAGTAAAGAAGAAAAAGGCTCGGGAGGCTTGTCGAGCGTGGGAAGAAAAGAAGAAAAGATAGTCTTGCCCCTGTGGGACTTGTAAATCTTGTTTGTAAGATTCATGAACATGTTTGTAATTTCATACGAAACTGTATCTTGTGACTATAAATAGAGTAGCAATGTTATGCATTTAAGAGGCATATGGGGCTTGGAACACGGACCTACTTCGCCCTTGAAGATACTTTTGAATTCTCAAATGTGAAGTCCAAGGTATATATGTAAATATTTATCATGTTACTGAGACGAAACAAACTTTATCAAGAAGGGAAATGAGTATTCGCTCTTATTTGTCAGGGATGAAACGAACTCGAAAGGAAATGAGTGTCTAATATAAATATTTGTCACTATCCTCATCCTCGGAAGCAACACTGGCTTCCATGTCCTGTTCATCCAACTCGTCTTCGTCGTCATACTCACCATTTTCATCATCCTCTCCTACGGCACCAAATTCATCCCCAATGATTTGTTCACTGCGACTACTCCTTCAGTCTTTGCCCTTGCCCTTTCCAACCCCCTCTTTGCCCATATGCATCAAATGAATATACTGATAAGTGATAATGATACCTTATCACCATCACGGATTCACGGGTACGGTGTGCGGCAGTCAGTCAGTCAGTGTCCTCAGCTTGTAGAAAATCCCAGGGTGCCTAGCCACCACGTTCCTGAACCCCGGTGGCAGCCGGCCGCAGCGCCTCCCCAAGCTGGACCAGCACCTCCTTCTCCATCTTCTTCCACACCGTGAGACTCAGGACCTCGTGCAGCACCGCCACAGTCCTCGTCCCCGTAATGTTACTCCTTGGCGCCAGGCGCGACCCACCCTCATAGGGCGAAATGTACGGCAGCCTCTGCCAATCGTCCAGCTATTTGCGCGCCTTCTTGTGGAGCTCGAATCCTCTGGGAACGAGAGTGGGAAGGCGACCGCGTCGCCGACCTTGTAGCCGCCGGTGCGCTGCGCGTAGACCTGCATCACCGACACGGCAGGGTTCTTCCTGTAGCAGACGAGCTCTCGAGGAGGCTTCCGTCCGGGGAGAGTAGTCCGGGAAGTCCGACGCGCGGAAGAGAGTGCAGCAGCACGTCGTGCGGCGGCGGCGGCGGCGAGGAGGAACGGCACTAGGTGGTGCTCCCGCTCCACGACGTGGTCGAGGGCCCCCGGTCTCGCGCTCACGGCACGCGCGCCTCAAGGAGGCGCCGACGGTGCACGCCACACCCGCGGCAGCGGCGGCAGAGGCATTGGGAGGCAGCCAGCCGAAATGCGGCGGCGGGTGGGAACGGGGAAGAGAGTCGATCGATCGAGCCAAGGAAAGGGGAGATCGGACGGTGGTCGGTGGTGTCTATTTTTAGGTGGAGTGCCGAGAGTCTCGGTCAAATTGAGGTCCAGCAGCATGGCGCAACGGCGGGGAGT

>ZMCNGC12

ACTTTCCCGATTCCCTAGTCTGACAGCGAAAAATAAAAGCAGATAGAAGCACAAGCACAAGCTTCAGCGTCACAGGCGCGCGCGCATGCATGGCGACAAACGCCAGTTAGGTTGATTCCGTCACCAAGCACGTACGTGCGTACGCTCTTGCAACAGTAGCGGCGTATATATGCGTCCATCACGCGTCGAGTCCTGCGGATGCCGTCTCCTCTGCTCCAACACCCACATGCCACGGCCTCACCACCTGGTCAGGCCACGCGTAGCCGTCGGCACTACTCGAGTCGGTAGTCGGTAGCAGCATCCGCTCCATCGATCGGCCATGCCAGTGCCAGCCGAGCTCTCGCCGCCGTCCCCTCACGGCGCGCCGCGGACCAGAGCCGGCGAGCAGGACGACAGCACGCTTCCTCCGACGGCGGCCGCCACCGCCACGGCCACGCGGGGCGGCACTGGCAGGAAGCGCCGGCGCGGGGCGCGGGACCCGCGCGCCAGCTGGGCCTGGGAGACGGAGTGGGACCGCGCGTACCTGCTCGCCTGCGCGGCGGGGCTCCTGGTGGACCCGCTCTTCCTGTACGCCGTCGCCGTCAGCGCGCCGCTCATGTGCGTCTTCCTCGACGCCTGGTTCGCGGCCGCGGTCACCGCGCTCCGGTGCGCGGTGGACGCCATGCACGCATCCAACCTGCTGCTGCGGCTCCGAGGGGCGTGCTCGCCGAGGCGGGAAGACACGGACGAGGAGGAGGCGCAGCCGGGGCGCGACGGCGGCGTGCCGGGACGGGGGACGAGGTCCAAGGAGGGGGTTTTCTTGGACGTGCTGGTCATCCTACCGGTGATGCAGGTGCGCCGCTGGTCCCAGTCCGTGCGTGCATGGCTCTCGTCGCTCGTTCGAAAATCGAAATGCTAGCTAGCTTCATCGGTCTCTTCTTGGCCATTTGAAAGGAACCATATTTCGGATTCATCGTAGTTAGATGTGCGTGCCCGGGCTCTAAAACTTTCGTGAGCCCGGCATTTTTAGCATGAAATTTCCTCGGTGGACAATGTTCGATACTCCAGGCCCAGGGTGAGCGAGTAGTGACAGATCGGAAGAAAAACAAAGACCATTGGCAGTTGGCACAACGGAGAATTTTGGAGCCTTCTGCGACATTGTCATCGTCCATGAATACCGTGGCATTATCGCATTTCCCCATCACATTCTGCTGAGCAATAGCTGCCGCATGTGACCCAACTCCGCCATTGCCGCTCTGCGCCCAGAGTAACCTGCTGGGTGCCTGCGCTGCGACCTGGGATGCCGATTTCCAGTGGTTCTGAAGGGTCATTTCAAGCAGCGATCTGCGCACGTACGGAAGTCGCTTGCGTTCATCGGCGTGGCGTGGCGTGGCGTTGCAGAATGGGGCCATTGTGCTTGCGCAATTCTGTAGCCCTACCCGGCACCGGCAATGCATGCTGGACTGGACGAAGCAGGTTGGGCCCGTTCATCAATCGATGAACCCAAAGATGCGTTCGATC

>ZMCNGC2

ATAGTAAGTCACTAACTGACCTCCACGTCCCCTTGAGCGACCTCGGACAGTAATTGTATCATCCATCTTTGGTATAATAATGTTATGTTGGTTACAAAAGGTCTTTACTTCCTCAAACAATTCCTCCCATCCATTATCACGCACTTCACTAATATTCCGCAATGTGGCTCCAATCAATCCAACAGCAAGGACAATATTCTGATCTTTTCTTTGCAAACATTGTGAAAAATCATTAGTCTTGCCTAATAACCTCATCATGAGATGCAGAATAAGCACAAATTCAAAGCTCTCCATTTGTTTCAACAAACCAGAAGCCATATATTTCTTATCCCCACTAGAGCCATCATCAGCCACAATCTCTAGTACCTCTAGAATAGCATCCCACATGAGAACAATACGACATAAATTTTTATGATGTGAACCCCATCGAGTATCCCCAGGTTGTGCTAGGTTGGTTTTTTTTATTTTTCCCTCTACCTGAAAAAATCCCCCCTTTCCAACATGCTCACAAGTTTCTCATGGTGTTGTTGAGCCAATTGATCCCTTCTCTTACAAGAAGCATTAACACTATTCACAATCAAGTTGCACGTTTGGAAGAAATCAAATACAGAAGCGCAACACTTAGCAACTGAAACAACCACTAATTGCAATTGATGTCAATCTACCATCACAAAAATATTTAACAATCTAGTATTTAGCTCGTTGTACACTAGTAACAAAAAAGAAGTTGAGAAAGTGAGAAAATCTTTACTGCTTACAGTTAGTACTTAGTAGGACTGTTGGATACTTGGAGCAGTGGACGGAAATGTAATGGAGAATTGGAGATTGGAGGCCGACTGCCACTGCCCGACTGGGCACTAGCTGAGCCGCCGAGGGGCAAGCCGGCGCCTGACACTAGCCTTGGCCCGCTGGCCGGACCTAGACGTGCAGACTGCGGACGATGGAGGGCGGACCGCAGACGGACCACGGGCGGACGGCGGACCGCGGACGACAGACCGACGTTCAGCTTCGGCAGACGATGAACGGACACAGCCACACGGACGCCGGAGGGCGGACGGCGGACGGGACGGGGGGCGACCCGGCGCCCGACGGCTGACGCTGGCTCTGGCTGGCAGGCCGGATCCAGACGACGGAGCCGCGCAGCGGACCCAGACGGCGGACGGGCGTCGAACGACCGATGGCGACGACCACGGGCACGGGCGGACGGGTGTGCGGTTGAGGCGGCTGGCGACCGGCGGCCGCCGTCACTCTGTGTGTGGTCGTGTCAAGCGGCTGGGAGCCTGGAGGCTGGGATTAAAGGCCGCCGAGTAGCAGGCCTTTGATGCCTATTAGGGGTTTTTGGGCTAAAATTCTATACATATATAAAAATGTTTTGGGCTACGACCCGGGCCATGACCTAGGTGGCCTAGGCCCAATATCCGCCCCTGGATATAATGAGAAGCTAAATTATTGAATCGTGCAATAGAAGAGTTTGAAGTACTTTAGAGAATTCACAATTAA

>ZMCNGC8

CACTGCTGCAAACAGTTTGGTTTACAGCCCCTCACAAAAAATAATGATAGACCCCACCTGCAGTAGCAATAATACAACTACAACGTTATCTGGTGGACCTGCAGGTGGGGTCTATCTGTCTTTTTGTGAGAGGCTGCAAACAGTATGATTTGCAGCAGGGCTGCACCAAATTTTTTTCATGAGAACAGGCCGTCACCACTTGGTCCACCCCTTCGCTGTCCTCTCTGGCTAAAAAGAACCGCAGAAAGATCGTTTTTTTTCCCTGTCCCCGTTCTTGCACTCGAGTTCTTCAGTTCTTAGGAATAGTCAGACGGAACAACTCGAGTTTGCCATGGTGGTGATCTGGAACCGGGGTGTGCTGCCCCCTCTTTGATTCCCTGATTGGTCTCGCTCCAGTTCGACGCGGGATTTGGACGGGACGGGCGGAAGTTGGGAGAGCAGAGCGGCCGGCATGTTTGGGTCCAGGGTCCAGGATGAGGTGGAGATGCAGAGGAGGCCTAACAACAGGTACACATCTTGATTCCATACGGCTTCCTTTTCCACCGCGATCTATTTTAAGTTCTGACGTTTTTGGATTGCCTGCTGAGAGTCGATGAGTTGCTGTTAGAATTTTGCTGAACTGAGATTGACTTTGGGTTTGCTGAGTTTTTTTTTTGTGGGGTGGGGGGGGGGGGGGGGGGGGGAGGTTCGGATTCGATTCTAGAAGAGAGGACATAAAAGGTGGTGGTGTACCTTGGTAGGCTCTTCGTTACCTCGAGGAATCGGATAGTACCAATTTTAAGCTTTGATCCCGTTTAAGCCTGGATTCAAGTTCGTTTTGCTGGAGGGGATGAGTGACTCTAGGATGGAGAGTTGGAGACTGAGAGATCTTTATTGGAGATTAAGGCTATGTTTGAATGCACTAAAGCTAATAGCTGGTTACTAAAATTAGGTGAGGACATCCAACTAATAGTTAAGCTAGTAGCTATTTTTAACAAATTAGCTAATAGTTAGCTAGCTAATTTCACTACCAATTTTTAGCCAACTAACATTAACTCTAGTGCATTCAAACACCCCCTAACTCGTCATGTCCACAAGAACGCGTTGGTTTAATTTAGTGTGCATGAGCATGGCAGAATCGTATGATAATTTGTGAAAATGGCAGTAGAATTTGAGAGCATTACTAGATTTCATTTGTTCAAGCTTAACGTTTGTCCTGCATTTATATTCAGGTTTATGGACAATCTTCATTTCCCCCCTTTCAACTAGCCTGAATCCCATGCAATTGCATGGTAAATTGAATAGTACGGTTCTATTTGCTAAACAGTTGTAACTTGCTTAGTTTAAAAAAGAGCTGTTTTTGTCCAACAATCACGACTACTATATATCTCTGTCTTACAATTAAGAGTTAGAGACTTCTTTTGGGACCTCTGTTATAATTCTCTTCTCTATATTAGTGATGTAAGGAGAGCTTCTAGACACTTTTACTTTTTAAGGATTGTGATAATCTCTAGCCCTTAG

>ZMCNGC9

GCTCGACAACGATGACGTGGGGCGACCCCCCCTTCTTCGTGGGTTGGTCTGCTTCGAAGATGAGGCATTTGGCGGAGGATTAAGGTTTTGGAAGTCTCGCTGGTGAAGAATCGGAGCTGTCTCGAGTGGGATGTCTCGTGGCTTGGCAACGACGGCGCGCCGCAGTCTTCCTTCTCCTGTGCTGGTGTTTGTAATCGTCGTCGTGTTTCCTTTGGCCTTGTGCTGCCCGTCTTGGGAGTCGGAGCTCGTCACTTATGTTGAGCTCGGCAACGATGACTCAGGATGGGCACTGTGTGTGTGGGTTGTTTGTGCGCCCTGTATTGGTTTTGGGCCCGGTTTCCCTTAAAACTGGGTCAATTCTTTCCTTCTTAATTGAGAGGCAGAGCTCCTGCCATTACGTTCAAAAAAAATATCCTCAAATAACAAGTATTGTGAAACATTTTCTCCTGAGGTGATGGACACGCATTGCAGCAGGGAGGGAATATTTGTTTACAATAACATATGTGCTCCTTACCTTAAAAGTGTTGATTAATTTCACCAAGCATTAGTATAATGTTTTAAGCTTTGTCTTGTCAGTTGGTTTAGCATGCAACCTGTCCCTCAGCTATAGATAGGTTTCTATTTTTTAATGATCAACTATTTGTCTTGTCAAATGAAAATTGACCAACCTGCTTATGTGACTTATTTCATGAATATAATTAGATATTTGTCTTTCAGTTCAGATTGGCTTATGTTGCACCAGAGTCACGAGTGGTGGGAGCTGGAGACTTGGTTGATGAGCCAAAAAAAGTTGCTATCCATTATCTTCGTGGTTACTTTTTACTTGATTTCTTTGTTGTGCTTCCACTCCCTCAGGTAACATAAAGCTTCCGTTCGCTAATTTCTCTTGCTTTAGATATTATAAGCTGCTTCTTTTTTACTTAATGAATTCACACATCTTACTTCACTGAAATAGATATTGAAACTCTATCTGTTAGAGACCCCTTTTTGTTTAATTATGCTGTGTTTTTATTTCTGTAATGTCTGAGTAGTTTCCATTAGATTTAGGGTCAACAAGAAATAAAATAACACACAAATTACGGTTTCAATACCCTTGTATATACCAAGGCATGCCATGACTTTTTCCTGTTCCTGAACTTGTGACATGTGAGCATCTGGGAAGAACTGATGTTGATTTAGTCATACTTACATCTAACCATAATTAAGGAATATGTTGGCGATTTGTGTGTTCGTCAACAACTTGTTTCTGCCTTGCCTTTTTGCAGCTTTGCTTTATGAACTTTGTCTGTATGTATAGCACTTCATCTGATTTTACTTGCATGAATGCCATGTATCTGTCAATGCCTAAAGTTGCTTGTCTAAACTCTGAACTGATGATTGATGACATGTTCTTTTCTGATGTAGTTAAGAACAAAAAAGTGAAATAATTTTACACATTTTTCTTCACTGCACTCTTTATGCCTTTTGCTAATATTTTTCTTTGTTTTTTTATCAAGGTGA

>ZMCNGC1

TTCCAATGTACACACGATCATGATGAATATTGCAAGCTTATGTTCGAAGATTCCTATGGCTTTGGGCTCAAGCACCAACAATACCAAATGAAATAGTTACCAAGACCATGATCAAGGGGCTTTGTCCAGGACTGACGACTCAATACTTTGCTAGAAACCCCCTCAATCCTTAGAAAAGCTTCTTTAGAAGATGGATGAGTATATCAGAGGAGACAATGACTTTCGTCAGAGGAAGGAGGAGACCTTGAGATATTCTAAGATGACCAGGGGCTTCGGCAGACGGTTCAACCCAGGACATATCAGAACCATACATAATCCAAATCAGAACGAAGACAAAAATAATCACACTTAGGGACAACAAAATTAACCTCAACCTATAGGAACCCAACAACAACAACAATCAAAAAATACCTTCACACCACCGGCCCAAGAGGTGGAAGAGGAGGAAGGAGTTTTGAAGGAAGATTCAACTTTCAACCAAGGAAAATGTCCTGCCTTTTCTGTGGAGAGGACAAGGGGCATACAACAAGAACCTATCAAGTCACAATCTAGAAGCTGGAGCACGCCAGAATCAACCAAAACAAATCTTTCACACTGCTTCATACTATTCTTCATACATTCCAGAATATGTTCGTAACCAATAGCCAAGTCCACAACCCTCAGGCTCAGTTGCTTCGGCTCATTGGATTATGCCTCAGCCATTTACACTAGCACCTTCGTCATATGAGCAGTAGTTTTCTGCGTAAAACACACCATAGCGAGATGCAAGAAAACAATCTGAGGCCAGAACAGTCGATAGTACTGTATAAGAATCACATCTACTGAAACACCTGAGGGCTGGATCAAACAACCGAAGCTATTCAAAAAATCTCCAAAGATGTAAAAGCTCCAAAGTCGTTTCTAAGGGAATGCAGAGCTCAATGAAAACACCTCGTAAAAGTGTAAAAGTTCCAAAGTCGTTCATAAGAGAATGCAGAGATCAATGAAAACATCTAGTAAAGGTGTAAAAGTTCCAAAATCATTCCCTTATCCAGTGATTTGAACAGTTGGTTTTGATGTTGTTTTAAGTATGGGTGAAATTTGTCTGGTGGACTTAGTGAAGATTATAAGTGTAGTTTTGCAAAATTTAAAGTGTTGCTGCGATCTGACCGTTGATTTTGATAGCGTGTTAAGTCTAAGTGAAATTTGTCTGTGGATTTAGTGAAGGTTATGATTATAGTTTTATAAAATTTAAAGTGTTGGATTATATAGTGGTATAGATAGATAAGTTTAGGTAAAATTTATTTGGTGAATTTAGTGAATGTTATGAGTGTGAGGTGATTTGGTTAGATGAGTTTTATAAAATTCAAAGTGGTGGGTTACCCCGCACGGTTTTCGGTTTTTTTCTTTGAAGTTGTGGACTTGTGGGTCACGAGAAGCAAGAACAATCCTGTGGATTTATTTAAAAAAAACTATATATCTAAAAAATATACTTGCAATTTGGAAATGGTGGAGTAGTTT

>ZMCNGC10

CTTTCACAGATGACAACCATTTCAGGCCCAGCCAAGCTTCAGAGCTAGGCCCCACCGGCCCAGTCTGTGTACCAAGCATGCGAGGCTCCAGGCCCACATGCACCTCAGCGCCTGTAATAAGATGGCATTCCATCTCGCCCACATGGACCGAGCCCATGCACGGCGGGCTCACGGGCCACCGGGTCACGCTCGCCGCCTCATCCCGCCACATCAGACAAAAAGGGCGAAAAAAATGCATCAGTTACAGTACTTTCCTTTGCCTAATTGGTCGTCTTGGCGCGGCATCACCGTCGTAGCATTTGCGTCAAGCCCACTCTGGGCTTCTTTGGATTACAGGAATTTTGAAGGAATAATGCTGGAATAGGAAAGTTTCCTATCCTATAGTTTGGCTGGCATGATGCACCAATTATATTCCATAGGATTTTATCTCTTCATGTGTTGATTCCATAGGATTTCTAACATTCACTCAGAGCTCTTGGAAAAATTCCTACACGCGAAGCACGAGAAGTGGAATGCTAATGCTAGCAACTAATGGTCCATTATGGCTTGTCCTACCCAAATTTAAATAGAGAAAAACACATACAAAACACCCATTTGATATTGTTTTTATCCAAGCTTGACATTCATGCAAAATGAACCCTTCGTTTTTTCCTTGCCCCAACCAAACAACCTTCCACTCAATATTACTACGTTTTTCAATCCTCTGATTTGCACATACAATCCTACATTTTTCCTATTCCTATATTTTTCAATTCCTGTAATCCAAAGAAGCCCTCTCTCCGGTCTCCGCCGCACAAGAGCAGAAGGGGGAAAAAACGCAGGTGCCCTGCGTAGCTGCGGAAACACGGCCGTGTTGTAGCCATCTGGTGCCTTTTCCGTATATGGACGTGCATCTTTGCTATGGCTACTACTACATATTACCACTAGCCCATCTCATGATTCGTGTTAGGTACGTACTAACTACATACATGGTGTTAAGTGTTAACGAGAAGTGAGACAGCCATTTGTTGTTGACATCACGGGAAAATGGCAAATATAAATATCGGCGCCGGGTACAAAATCGGATCATCCCAATCGGACGCGAACCCCTAGCGCGATCATCACCATTATTGTGATGTGCCCGGCTCACCTGTGCCAGGAACCCGGTGGCCCCACTACGAACAATTGCTCGAGTCAATCCGGTAGGTGTAGCCGTGTCTGCGTAGCCGTTCCGGGGAGCCAAGGGCTCGGCACCACCGCGGCATCGGTGCACAAGGACGGCGCACGTACGCGCCACGTCCTTGGGCCCCGGCGCAGTTGTACCCTCCATCTCTGTCCTCTTTACTTGCCCCATCCATCCCACGCCGATCGCCAGGCGCCAGCAGCAGGCTAGCTCTGCTCCGCCGCTCACTGCTCGCTAGCTACTGGACCGCGCTTCCCTCTCTCCATCGCGCCGGCGCGCACGGCTAGCTCTAGCTCCCGGAGCCGCCCTTTCATGGTCCCCGCCGTGGTCGGGACGAG

**3.cDNA sequence**

>ZMCNGC6

CCACTTCAAATATTTTTTAGGCTGCTTCAAAAAACATGGTTTAGAAGTGCTCTAAATACTTCAAGTCAACTCCGTTCTAGATGAACGCTCGTTTCCCTCCCGGCGACTACTTGCATCCATTACCGGCTATTGCTTCCATTACCGTCCGCGCTTAGCACTAACCACAGTGAAAACTGCGCCACTAACCGCAGCTTTAACCCCAATTCCATCAATTAGCGGCGACTGATAAAGTAATTAAGCCGGTGGCGAAGCATCCTCGTCCGCGTCCGCATCCGCACCGATCCGGTACGGGAGTTCTCGGCTCCGACGCCCCACGCGGCCCCGCCTCGCGCTCCTTCCTCGAACACCTCGCCGCCGGCTCTCCTCAGCTCCGCCGACTCGCTTCGCTGTCCTCCTCGCCCCCAGCCTCCTCCTGATCCCAGATTCGCCTCGCCGGCCCCCGGCGCCCGCCACGAGCGCCGTCAAGCGAAGCCGTTCTTCCCTGGCGCGCGCCGCTGGCGTAGCTGCCAGGCTCCTTCCGCCGAAGCACATCGCGGCCAGCTGGCGCTCGTCGTCCACCGTCGTCGCGGCGGACTTCGTCCTCCTCCCGGAAGCACAGATCCGCACAGGCTCGGCTGCATTTCTCCACTTGCTGGTAAGGAAGCTCGCTCCGATGGATTACTCCGAGGGTTAGTTCCGCCCTGGTCTTCCGTTTCTACGCTGCCAGCAATCAAGTTCCAACAAGTCGTCGATGGACTGCGATTTGTTCGCTGCGTGGTGGAGCAGCAGCACCAGATTAGTCTCCAGGATTTTTCGGGGGTCAGCTGATGCCCCGGGCCCGTCGCCAGCGAGGCCAGCCATGCCGCTTCACCAGAAGCAGGCGGGGCTCGCTGCTAGCAAGCTGGGCGTGGGGACCTCGAAGAAACACAGGGCTTTTGTCGCAAGCGATGAGCAGTGGTACAACAAGATTTTTGATCCGTCAAGCGACTTCATCTTGACATGGAACCGCATTTTCCTCTTCTCCTGCTTCGTCGCGCTATTTATAGACCCCCTCTATTTCTATGTGCCCAAGATCAGCTACGGCAGCCCCAAATTCTGTATCGGAACAGACACCCGTTTCGCCGTCGGTGTTACATTCTTCAGATCGATTGCGGATTTATTGTATGTCCTGCACATCATAATAAAGTTCAGAACAGCATATATCAATCCAAGCTCGACTCTGAGGGTGTTTGGAAGAGGGGATCTTGTCACAAATCCCAAGGAAATTGCGTGGAAATATATCAGATCTGACTTAGCTGTTGATGTGGCAGCTGCTTTGCCTTTGCCACAGATTATTGTCTGGTTTGTGATACCAGCTATAAAGTATTCCTCTGCTGAGCACAACAATAACATTCTGGTGCTCATAGTTCTTGCTCAGTATCTTCCAAGATTGTATCTCATATTCCCCTTAACTTATGAAATTGTCAAAGCTACTGGAGTTGTTGCAAAGACTGCTTGGGAAGGAGCTGCATACAACATGGTGCTCTATCTGATAGCTAGTCATGTGCTAGGTGCACTGTGGTATTTGCTATCTGTTGATCGCCAGACATTCTGCTGGAAGACGAACTGCCTGAATGAAACTGGTTGTGATCTTAAGTACCTAGATTGTGACACGACACCAAATGCTACATGGGCGAATACGACTGCTGTCTTCAGTAATTGTAATGCTAGCGATACCAATATAAGTTTTGATTTTGGCATGTTCGAGCCTGCATTGTCTAATCAAGCCCCTGCTCAAAGTTTTGCGATGAAGTATTTCTATTCCCTCTGGTGGGGATTGCAGAATTTAAGCTGCTACGGTCAGACTCTTACTGTGAGCACCTATCTTGGCGAGACGCTGTATTGTATATTCTTGGCGGTACTTGGTCTTGTCTTGTTTGCGCATTTGATTGGAAATGTGCAGACCTACCTGCAATCTATTACTGTGAGGGTTGAGGAATGGAGATTAAAGCAAAGAGATACTGAGGAATGGATGAGACATCGTCAGCTTCCTTGTGAACTGCGGGAAAGGGTGAGACGATTTATCCAGTACAAGTGGCTTGCAACAAGAGGAGTGAACGAAGAGTCAATATTGCATGCTCTGCCTGCAGACCTTCGACGTGACATTAAGCGCCACCTTTGCCTGGGTCTTGTTCGACGGGTTCCTTTTTTCTCCCAGATGGATGATCAGCTTCTTGATGCCATCTGTGAGCGTCTTGTATCATCACTGTGCACAAAAGGCACATACATTGTCCGTGAGGGTGATCCGGTGACAGAGATGCTCTTCATCATCCGTGGAAAACTGGAAAGCTCCACAACAAATGGTGGCCGCACTGGCTTCTTCAATTCAATCACCCTGAAACCCGGTGATTTCTGTGGCGAGGAGCTTCTTGGATGGGCTCTTGTCCCCAGGCCTACTACAAATTTGCCGTCATCCACTCGGACAGTGAAGGCACTGATAGAAGTAGAGGCCTTTGCGCTCCAGGCGGAGGATCTCAAGTTTGTTGCCAGCCAGTTCAGGCGGCTGCACAGCAAGAAACTGCAGCACACTTTCCGGTACTACTCGCACCACTGGAGGACGTGGGCCTCATGCTTCATCCAAGCTGCCTGGAGACGGTACAAGCGAAGGAAGATGGCAAAGGACCTGAGTATGAGGGAGTCATTCAACTCCGTTAGATTAGACGAAGTGGATAACGAAGATGACGATTCTCCGCCCAAGAATAGCCTTGCTCTAAAATTCATAGCTAGGACTAGAAAAGTGCCTCAGAACATGAAAGAGTTGCCGAAGATAACGAAGCCAGACGAGCCAGATTTCTCAGCTGAACCCGAAGACTAAAGTTGTCTTTGCTTATGCTATCATGTACAGTGGTCATAGCTGACTATTGTGAATTGCTAGTCCTATTTGGTTCGTGATGGAGGTTACAAGCCAACTAGGTATGGTGGGTGAATTGAACTTGGGTTGACGGTGCCATCTGTGGATGTGAGGAAGGTAACTCAGAATATCAATGAAAGCGTAATGAAGCTTCTGACTGGTATATAGACTGAATGGTTGTAAGTTAACTAGTTGCTACGTTCATGTTGTATATTCAGACATCTCTGTGCCTATAGATTTCTTGAAAAAAAAAGGAAAGAAAAAACAAGAATACATCATTACATAGACTGTAATCTTAAACATCTGACATAAGGGTGTGGCTACGTTGTCCGTGGCCGTGGAGGACTGTAAATCATGAGGGTGTGGCTTCTGGTAAAATGGCTAGCACCACGCAGTGATGAGAGATATCTCGTTGAGTCATCAGGGCGCAGATTCGAACAGGGGCGGAGCTAGGCCGCGGATTCGAACAGGCGCAGAGCTAGGCCTGTTTATCGGTGTCAGCTGACACCGGCGGCTTTTTGCAAAATCAATGAGAAATATTTGATATGCGTTGTTTATCCATAGAGCTGATATCAGTGTCTAGTGGAATCGGCTCCTTCAGCTGGCTTCGCTCCGCATTTGTGAGACAAAGGTTT

>ZMCNGC4

TTCTGGTACAACCGAACAGGCCCTTAGTCGTTGACCGTCTCGCTCGCCTCCCCGTCCGCTCCGCTCCCGTGCTTTCCCCTTTGCTCGCTCGCGGACCTCCGTCCCGGACTCCGACTCCCGGCGGCGCTCTCGCGCCAGCAAGCTCGAATCTGCACGCCGCCGCCGCCGCCGCCGCCGCCCCACGCCCACGGCGCCTAGAACCAGGGTCTTTCTGGTGTGCAGGGGTGGGCGCTTGGCTTCTGAGAACATGTAACGGGTGTTGATACGAAAATAGGTTCCTGGAATAGCGGGTTCATTAGCAGTTTGGCACAACTTTGAAGCTGCAGGGGATTTCAAATGCCCTTTGTCATCAGGTCCACGGTTCCACTGAGCCAGAGGATTTTTTATTGTATTTTCAACTTCCCTGAGGATGTTTGACAGTACTCAAAAAGCTCAATACATGGATGGGCACAGAGAGAGGTTTATTAGGCTGGATGAGTCAAGTCCTAGGTCATCTGTACCTTCTGAAGTGGGAGGCAGGAGTACATTGAGGTCCAGCATGCCTGGGTTTGGTTATGGTCCATTTAATGCACTAAGGTCTTTCTTGTCTGGGGGCTCTGGAAGACTGAAGTCACTTAGACAGTCACTTACTTCTGGTGCTCCTAAGACAGCTTTTGCGGAAGATCTTAAATCATATAAGAGAACTATATTTGATCCCCAGGACAAACTTCTTTTCCGAATGAACTGGGTTTTCTTCTCGTCTTGTCTTTTTGCTGTTGCAGTGGACCCACTATTCTTCTTCCTACCCATCATCAACGATTCAAACTGCATTGGTATTGATAAAAAGTTGGCAGTGACATCAACAATAATACGTACGGTTATTGATTTTGTCTACCTTATACGCGTGTGTCTTCAATTCCGCACTGCTTATGTTGCTCCATCTTCACGCGTGTTTGGGACTGGTGAGCTTGTGATTGATCCGATGCTAATTGCAAAGCGATACATTAAAAGTTACTTTGCAATGGACTTTGTTGCATTGCTACCACTTCCACAGATTGTTGTATGGAGATACCTCCATATTCCAGATGGCCCAGATGTACTGACTACGAAAACTGCACTGGTTTGGGTTGTTTTGATCCAATACATTCCAAGGTTGCTTCGAATATTCCCTGTGATCACAGATTTGAAAAGGACAGCTGGTGTTTTTATTGAAACTGCTTGGGCTGGTGCTGCTTATTATCTTCTATGGTTTATGCTGGCTGGGCATAATGTTGGTACTTTATGGTACTTTTTAACCATAGAACGTGAAGATGATTGCTGGCATCTATACTGTGACGATCCCAACTTTGGCTTGGGATGTAATAGCAGCTACTTGTATTGCAATAATCATCATCATGGCAGCTATGATAGTTGGCTTACGAATAATAGTGCCCAAGTATTCAACATGTGCAATGGCGGTCAAGACAATCCTTTCAACTTTGGCATTTATGAGCAAGCACTGGTCTCTAAAATACTTAGTCCAGGAAATTTCATCTCAAAATTATGTTATTGCTTCTGGTGGGGATTGCAAAATCTAAGTACACTTGGTCAAGGGCTTTTGACAAGCACATACCCTGGAGAAGTCCTGTTCTCTATAGCAATATGTGTCCTTGGACTAATTCTTTTTGCTCTCCTCATTGGTAACATGCAGAGCTACCTACAATCTGTTGCTATACGCCTTGAAGAGATGAGAGTTAAGAAACGCGATGCTGAGCAGTGGATGCATCACCGTTCACTGCCACTGGACATCAGACATAGGGTGAGGAAGTATGAACGTTATCGGTGGTTGGAAACCAGGGGAGTAGACGAAGAAACTTTGGTTCAAACTCTTCCAAAAGATCTTAGGAGGGATATCAAGCGGCATCTTTGTTTGGGCTTAGTGAAAAGGGTGCCTTTGTTTGAAAATATGGATGAACGATTGTTGGATGCAATATGTGAGCGGTTAAGACCTGCACTCTACACAGAAAATGAGTTCATTTTGAGGGAAGGTGACCCGGTGGATGAGATGCACTTTATTCTTCATGGTTGTTTGGAGAGTGTAACCACTGATGGTGGACGGAGTGGGTTCTTTAACAAGGTTCAGCTAAAGGAAGGGTCGTTCTGTGGCGATGAGTTGCTCACTTGGGCATTGGATCCCAAGTCAGCTGCTAATTTTCCAGTTTCGAGCAGGACTGTCCAGGCACTCACCGAGGTTGAGGCGTTTGCCCTATGTGCAGAAGAGCTGAAATTCGTGGCCAGTCAGTTCAGGAGGCTGCACAGCAGGCAAGTGCAACACACATTCCGATTCTATTCCCAGCAGTGGAGGACTTGGGCAGCCTGCTTCATCCAAGCAGCATGGCGCCGCTACTACAAGAGGAAGATGGCAGAGCAGCGGCGCAAAGAAGAAGAGGCGGCAAGCCGGCCAAGTAGTAGCCACCCTAGCCTTGGGGCGACTATCTATGCATCTCGTTTCGCTGCCAACGCCATGCGAGGGGTTCACAGGCTAAGAAGCAAGGCTGTCCCTACCATTGTCAGGCTACCGAAACCCCCAGAACCAGATTTTGGTGTCGATGATGCTGACTAACGAGACAATCACACTGTACTCGCTTGTAATTGTACTTTACCTCATCTGTAGATTCTGTACAAGATTCAACGACAGCTGAAGAAGATAGTGTCTGTAAAGGTTTGTAACCACGCACAGACAGACCATAACGGAGGTATATCCTGCTTGACGTCTATGGAAATGCAAATCTACTTCCTTAAAACCTGAAGTGTTTTGGAGCCACCTCCGCGCTTCCGGGGTACGGGCGTACGGCATCAGTGTCACCTGCGGACGAGTGGCGGGATCGGATCTAAAAAATTTATCCACTGGAGTTGTGGGAGATGTCGTGTCTGGTGCCCTCTCCCTCCGTCCCGTGCTGCACTGCGCTTTCTTCTTTGTGATTGCTTCGCTCGCCTCCTCCACCACCAGACACCAGCCCAAACTACCTCGCCAGCTCCCTCTGCCTCCCCCTCCCTCCTCCCGTCGCCGCAGCACGCAGCTCCGCCGGTTTTCACGGCTTGGAGCAGATCTGCCACGCCACCGCCGCGTAGACCAACTTGGTGGAGGGATCCCGTCCGAATCTGATTCCTATTCTCCAGCTCGACCGCTGTAGCGAGAGGAGAGAGAGAAAGGAGCAGTGGCGACGAGGTCTATGGCATCGATGGCAGCGTACCTGGCCTGGCTCGGCGGGCGGCGCAGAAGAAGTGGGTGCACCTGCTTTACCACACGGCGCTCAACTTCCACCGCCACCTCTTCTACCAGGAAATCAGTTCCCCCAATCCTCCCCTCTTCGCCTCCCTTTTGATCTCGATCTGCCTTCCTGCTCGCGGACGGGACTGGCCTTGTGTTGTCTCTCCCGCGCAGGCGTCAGATCTCCTCGACAAGTTTGAGACCAACTGCCACGTGGTAAGGCCTCCTCCTGCTGATCCCTTTTCGCACACCTCCTCTTCCCATTTGTCTGTTAGTGGGGAGTGAATTCATGAGTTACATTTGGTGATGATGGTCTCTATGTGGTGCCGTGATTGTGAGCTAGAACAAGTTTTTTCTATCATGTTTAATTGTTAAAATGCCTCCGTTGAGACGCCAAGACTCAGCACCGTTGTGGTGTCGTCACATCCATCTCTAGCCTCCCGAGTCACCAGCGCTGCCCAAGTTAGCTGAGCTCCGTCTGTTCTGCTTTCGCGCCGCACAACACCATTGAGCTCCTCCCCGACGCCACGTCCCGAGGTCTGCCGAGCGGCCGGCTCTGCCACGCTGAGATGTGCCCTGTCCCCTGCCTAGGTCGA

>ZMCNGC3

TGTCTCCAAATTTAATTGTTTTCTTTTTCTTGGCTGGATTGAATTATTGTTGCAGACCTATCTACAATCAGCCTCGGGGCATATAGAGGAAATGAGAGTGCGAAGACGTGACATGGAGCAATGGATGTCATACAGACTACTTCCAGAGCATATCAAGGAACGAATACTGCGTCACCATCAATATCGGTGGCAAGAAACACAAGGCGTGGATGAAGAGGGCCTTCTTGTAAACCTTCCCAAGGACCTCAGGAGGGATATCAAGCGTCATCTTTGTCTGTCACTTCTCAAGAGGGTTCCAATGTTCGAAAACATGGACGACCAACTCCTGGACGCAATGTGCGACCGTGTGAAACCCATGCTGTACACAGAAGGAAGCCACATCGTTCGCGAAGGCGACCCAGTGAACGAGATGTTCTTCATCATGAGAGGGAGGCTAGAGAGCACGACAACGGACGGGGGGCGAGCGGGCTTCTTCAACTCCAACGTCCTCGAAGGCGGCGACTTCTGCGGCGAGGAGCTCCTCACGTGGGCCCTGGACCCAGCCTCGGGCTCCAACCTTCCAAGCTCGACGAGGACGGCGAGGACGCTGTCGGAGGTGGAAGGCTTCTCCCTGAGGGCTCGCCACCTGAGGTTCGTGGCCAGCCAGTACAGGCGGCTCCACAGCAAGCAGCTCCGGCACACCTTCAGGTTCTACTCCCACCAGTGGCGGACCTGGGCCGCGTGCTTCGTACAGGCGGCTTGGCACAGGTACTGCCGGAGGAGGCTGGAGGAGGGCGTGCGCGAGAAGGAGAGGATGTTCCGGGCAGCGGCCGTGACCGACATCTCCAGCTCCCGCAGCCTCGGCGCCGCGCTCTACGCTGCCCACTTCGCTCGCAACATGGTAAGGACGCTGCGGAGGAACGCCGCACGGAAGGCCCGTCTGCTGGATACAGTGTCTTCGAGGCTGTTGCAGAAGCCAGCGGAACCCAACTTTTTCGCTGAAGAAGACTGACCTTTCTTTAATTTCTACCTTGATACTGAGATGTAATTGGAGCGGATAAGGAGGTATATATTTGTTTTTATATTTTCTCTTCGGCTAATTTCGTTAATTAATCTCGTGTTC

>ZMCNGC7

CCCGTTTTTACCTAGTCTCGACGAATACCTTAACGTTTGTGACCAGAAGCTGTGAGAGATCACCATGGCATCCGGTGCTTCACGAAATGTCAGGTTCCAGAACGAGATCGAGGTCCAGAGCTTCAGAACAAGCCCTCTGCAGAGCCTCAGCAGAAAGCACGGCAAGGCTCACGATCCCAGGAAATGCCGGCTGGGTTTCCGCGGCGGCTGCCTGGAGAAGGCGTGCCGGAACCGGAAGCCGATGCTGAAGGACAGGGTGCTCTCGCGCGCCTTCTCGGAGGAGCTGGAGTCCCTGATGCACGCCGCCGGCGGCAGCCACCTCTTCTTCGACCCGCGCGGGCAGCTGATCCACCTGTGGAACAAGATCTTCCTGTCCGCCTGCCTGCTGTCGCTGTTCGTGGACCCGCTGTTCCTGTACCTGACGGGCACGCAGCGGAACACGTGCGTCGAGTTCAAGGACTCGCTGGCGCTCACGCTGTCCATGGTCCGCTCGCTGCTGGACCTCTTCTACGCCGCGCACATCCTGTTCCGCTTCCGCACCGCCTTCATCGCGCCGTCGTCGCGCGTGTTCGGGCGGGGCGAGCTCGTCATCCAGCCCTACGAGATCGCCAGGAGGTACCTTGGCCGGACGTTCTGGTTCGATCTCGTCACGGCGCTGCCCTTGCCGCAGTTCGTGATCTGGATCGTTATACCAAGGCTGAATGAGTACTCCCGGACGGCGAACACGAAGAACATCCTCCGGTTCAGCATCATCTTCCAGTACCTCCCGCGGTTGTTCCAGATATTCCCGCTCTCGGGGCGGATCGTCATGGCGACGGGGGTCATGACGGAGACGGCGTGGGCCGGCGCCGCGTACAACCTGATCCTCTACATGCTCGCAAGCCACGTGCTGGGAGCGCTGTGGTATCTCTTCTCCGTGCAGAGGCAGGAGGCATGCTGGAGGGAGGCGTGTCTGCTCGTGAGCCCGACGTCCCAGACCATGTTCTTCGACTGCAAGGCGTTGAGCAGCAACAGGACGATCTGGTATGAGCTGAGCAACATCACAACAAGCCGGTGCACGCCTGGCAACGGCTTCTACCCGTTCGGTATCTACGAGGAGGCGCTGTACGCCAAGCTCACGTCGTCGTCTTTCACCCAGAAGTACTTCTACTGCTTCTGGTGGGGACTCAAGAACCTCAGCTCCTTAGGACAGAATCTGTCGACGAGCTTGTTCATCGGTGAAATAACCTTCGCGATCGTCGTCGGCGTTCTTGGGTTAGTGCTGTTTGGCCTGCTCATCGGCAACATGCAATCTTACCTCCAAGCAACGATGGTGCGGCTGGAGGAGTGGCGGACGAAGCGGACGGACATGGAGCGGTGGATGCACCACCGGCAGATCCCCCAGCCGCTGAAGCAGTGCGTCCGGAGGTACCACCAGTACCAGTGGGTGGCCACGCGCGGCGTCGACGAGGAGGCCTTGCTGCAGGACCTCCCCATGGACATCCGCCGCGACATCAAGCGCCACCTCTGCCTCGACCTCGTCCGGAGGGTGCCCCTGTTCGACGAGATGGACGAGCGGATGCTGGACGCCATCTGCGAGCGGCTGAGGCCGGCGCTGTACACGCGCGGCACGCGGCTGATGCGGGAGCTGGACCCCGTCGACTCCATGCTCTTCATCATCCGGGGCTACCTCGACTCGTACACGACGCAGGGCGGCCGCTCCGGCTTCTTCAACTCGTGCCGCATCGGCGCCGGCGAGTTCTGCGGGGAGGAGCTCCTGACGTGGGCGCTCGACCCGCGCCCCGCGGCGAAGCTGCCGCTGTCCACCCGGACCGTGCGCGCCGTGTCCGAGGTCGAGGCGTTCGCGCTCGTGGCCGACGACCTCCGCTTCGTGGCGTCGCAGTTCCGCCGCCTGCACAGCGCGCGCATCCGCCACAGGTTCCGCTTCTACTCACACCAGTGGCGCACGTGGGCCGCGTGCTTCATCCAGGCCGCCTGGCGGCGATACAAGCGGCGCCGCGCGTCCATGGAGCTCAGGGTGCGCGAGGTGCGGGCCGGAGGGAGCTTGCTGCGGTCCCGCCGCCACAGCATCGAGGGCAAGGCGTCGATTAGGAAACCCATGGAACCGGACTTCACGGTGGAAGAAGAGGACTGATCAACGAATGATTACCTAGCTAGCAGCTTTTTTTTTCCTTTC

>ZMCNGC11

CTTGCCTCCATCCACTGGCACTGATCCACCACATCCCACGCCGAGCGCGACGCCAGCAGCAGGCTAGCTCCGCTGTCCGCTCCGCCGCTCACTAGCTGCTGGACCGCGCCTCCCTCCATCGCGCCGGCGCGCGCGCCTAACTAGCGGAGCCGCCTTTTCATGGTCCCCGCCGTGGTCGGGACGAGATGCCTCGCCTCGCATTCCTCCGCCGCTCCCTCCCCGCGAGGCTTCTCGCGCGAGCGTGTGGTGGTGGTGGTGGTGGAGACCAGGGGAGCCCGGACCAGGTGGCGCGGGACGAGGAGGCCGGAGGCAGCGGCGGAATGAGCGGGCGGTCGTCGGCGGGTGGGCCGTCCGGCGGGGAGTGCTACGCGTGCACGCAGCCCGGGGTGCCGGCGTTCCACTCCACGACGTGCGACCAGGTGCACTCGCCGGACTGGGACGCCGACGCGGGGTCCTCGCTCGTGCCGGTCCAGGGCCAGGCGCAGGCGGCGGCGGCGGCGGCGGCGCCGCGGCAGCGGCACGCGGCGCGGTGGCTGTTGGGGCCCGTGCTGGACCCGCGGAGCAGGCGCGTGCAGCGCTGGAACCGCTGGATCCTGCTGGGCCGCGCCGCCGCGCTGGCGGTGGACCCGCTCTTCTTCTACGCGCTCTCCATCGGCCGCGCTGGCCAGCCCTGCCTCTACATGGACGCCGGGCTCGCCTCCGCGGTCACGGCGCTGCGGACCTGCGCCGACGTGGCGCACCTCGCGCACGTGCTCCTGCAGCTCCGCCTCGCCTACGTCTCCCGCGAGTCCCTCGTCGTCGGGTGCGGCAAGCTCGTGTGGGACGCCCGCGCCGTCGCCGCGCACTACGCACGCTCCGTCAAGGGCCTCTGCTTCGACCTCTTCGTCATCCTCCCCATCCCGCAGGTTATCTTCTGGCTGGTTATACCAAAGTTAATCAGGGAGGAGCAGGTTAAGCTTATCATGACAATACTGCTGCTCATGTTCATATTTCAATTTCTCCCCAAGGTCTACCATAGTATACACATCATGAGGAAAATGCAGAAGGTGACAGGTTACATCTTTGGATCAATATGGTGGGGATTTGGTTTAAATCTATTTGCCTATTTCATTGCTTCTCATATTGCGGGTGGGTGCTGGTATGTTCTTGCGATCCAGCGCATCGCTTCCTGCCTCCAGGAAGAATGCAAGAGAAACAACAGTTGCGATCTAATATCGCTAGCTTGTTCCAAGGAGATATGTTTTCACCCTCCGTGGTCATCGAATGTTAACGGATTCGCGTGCGATACGAACATGACCTCCTTTAGCCAACAAAATGTGTCTACTTGCTTGAGTGGAAAAGGTTCCTTCGCTTATGGAATCTATTTGGGGGCTCTTCCTGTTATATCGAGCAATTCGCTCGCTGTCAAAATACTCTATCCTATATTTTGGGGCCTCATGACACTCAGTACTTTTGGTAACGACCTTGCCCCAACAAGCAATGGCATTGAGGTGATATTCAGCATAATCAATGTCCTCAGTGGCCTTATGCTCTTCACGTTGCTGATCGGAAACATACAGGTATTTCTGCACGCGGTCCTGGCAAGGAAGCGGAAGATGCAGCTGCGGTTCCGGGACATGGAATGGTGGATGAGGCGGAGGCAACTACCGTCCCGGCTGAGGCAAAGGGTGCGCAAATACGAGCGCGAACGCTGGGCCGCCGTCACGGGAGACGAGGAGATGGAGATGATCAAGGATCTGCCTGAAGGGCTGAGGCGGGACATCAAGCGCTACCTGTGCCTTGAGCTAGTTAAGCAGGTTCCACTGTTCCATGGCATGGACGACCTGATCCTGGACAACATCTGCGACCGGCTGCGGCCTCTGGTGTTGTCCAGCGGGGAGAAGGTGATCCGGGAGGGCGACCCCGTGCAGCGCATGGTGTTCATCCTGCAGGGCAAGCTCCGGAGCACGCAGCCGCTGACCAAAGGCGTGGTGGCGACGTGCATGCTGGGCGCGGGCAGCTTCCTGGGCGACGAGCTGCTGTCGTGGTGCCTGCGCCGCCCCTTCGTGGACCGGCTCCCCGCGTCGTCGGCCACGTTCGAGTGCGTGGAAGCGGCGCAGGCGTTCTGCCTCGGCGCGCCGGACCTGCGGTTCATCACCGAGCACTTCCGGTACAACTTCGCCAACGAGAAGCTCAAGCGCACGGCGCGGTACTACTCGTCCAACTGGCGGACGTGGGCCGCCGTCAACATCCAGCTCGCGTGGCGCAGGTACAGGGCCCGGACGTCGGCGGACCTGGCGGCGCCGCCGTTGGTGGGCGGGCCCGACGACGGGGACCGGCGGCTCAGACACTACGCGGCCATGTTCATGTCGCTACGGCCGCATGACCACCTAGAGTGAGCACTGATGAGCAGGAGGGAGGGACCATCCTAGGCTCCTAGCTGTGCCGGCCGCGTCATGGTGACTGTACAGTGTGCACTGTAATAGTATATTGCTGCCATCTTTTGCGTAAGTGAATTAGTGAATTGTCAATTGCCTTTTCCCCCACAAGAATCCGAACTCTGAAATACTTCTGTTGGCCTGTCTCTCCGCGTTCGTCAGGATATTGCTGCTTCTGACCGCGCCATTGCGGCCTCAGCTGCAGG

>ZMCNGC5

GAAACGGTGGGCAACGACCAAAGAGGCACATTTTTTTGGCGGTTTCTCAACGTCGATTGATGCGGTGGTGATTTGTGAGAGACTGCGACCTCTCATCTCATTGTCAGGAATTTGGTCTTCGACGCAGGAGAGACGAACGACGCACTCACGAACGAAGCGCGAGTGAGCATGGCAGGATTGCTGGCGATACAATGAATGAATCGAACTGATCCGCTGGAGGCGCCTCCTTCCCCTGCATTGCATTGCTTCCATCACGAGAGGAGGGCGACTGATTGATTTCCACACGTGGTGGGTGGCGGCGCGCTCCTAGGTTTCAATCTGACGACGACGACGGCGACGACACGAGCTCGCAAGATGTCGTACGACCAGTCGGCTTTCCAGGTCGACTACATGGGCGTCGGCGCCGGCGCCGGCGTCAGCGCGTCCCGGCGGCGGTTCATGCCTTCGGAGTCGCTGGCCCGCGGCGTCATCACGCACGGCTCGGCGCAGCTGCGCACCATCGGGCGGTCGCTCCGGGCCGGCGCCACCATGGCGGCCGTGTTCCAGGAGGACCTCAAGAACACCTCCCGGCGCATCTTCGACCCGCAGGACCCGGTGCTGGTGCGCCTCAACCGCGCCTTCTTCATCTCCTGCATCGTGGCCATCGCCGTGGACCCCATGTTCTTCTACCTGCCCATGGTCACCGACGAGGGCAACCTGTGCGTGGGCATCGACCGCTGGCTCGCCATCTCCACCACCGTCGTGCGCTGCGTGGTGGACCTCTTCTTCCTGGGCCGCATCGCGCTGCAGTTCCGCACCGCCTACATCAAGCCGTCCTCCAGGGTGTTCGGGCGCGGCGAGCTCGTGATCGACACCGCGCTCATCGCCCGCCGCTACATGCGCCGCTTCTTCTCCGCGGACCTCATGTCCGTGCTCCCGCTGCCCCAGGTGGTCATCTGGAAGTTCCTGCACCGGTCCAAGGGCACCGCCGTGCTGGACACCAAGAACAGCCTGCTCTTCATCGTCTTCATCCAGTACGTCCCGCGCGTGGTGCGCATCTACCCCATCTCCTCGGAGCTCAAGCGCACCAGCGGCGTCTTCGCCGAGACCGCCTACGCCGGCGCCGCCTACTACCTCCTCTGGTACATGCTGGCCAGCCACATCGTCGGCGCCTTCTGGTACCTGCTGTCCATCGAGCGGGTCAGCGACTGCTGGAGGAACGCGTGCGACGAGTTCCCCGGGTGCAACCAGATCTACATGTACTGCGGCAACGACCGGCAGCTGGGGTTCCTGGAGTGGCGCACCATCACCCGGCAGGTGATCAACGAGACGTGCGAGCCCAAGCGGGACGGCAGCATCCCCTTCAACTACGGCATATACTCGCCGGCCGTCGTGTCGGACGTGCTCAAGTCCAAGGACACCACCTCCAAGCTGCTCTTCTGCCTCTGGTGGGGGCTGGCCAACCTGAGCACCCTCGGGCAGGGGCTCAAGACCAGCATCTACACCGGGGAGGCGCTCTTCTCCATCGCGCTCGCCATCTTCGGCCTCATCCTCATGGCCATGCTCATCGGCAACATCCAGACCTATCTCCAGTCCCTCACCGTGCGCCTGGAGGAGATGCGCGTGAAGCAGCGCGACTCGGAGCAGTGGATGCACCACCGGCTGCTGCCGCCGGAGCTGCGCGAGCGCGTCCGCCGCTACGACCAGTACAAGTGGCTCAACACCCACGGCGTCGACGAGGAGGCGCTGGTGCAAAACCTGCCCAAGGACCTCCGCCGCGACATCAAGCGCCACCTCTGCCTCGGCCTCGTCCGCCGGGTGCCGCTCTTCGCCAACATGGACGAGCGCCTCCTGGACGCCATCTGCGAGCGCCTCAAGCCCAGCCTGTGCACGGAGCACACCTACATCACCCGGGAGGGCGACCCCGTCGACCAGATGGTCTTCATCATCCGCGGCAGCCTCGAGAGCATCACCACCGACGGCGGCCGCACGGGGTTCTACAACCGCAGCCTGCTCGAGGAGGGCGACTTCTGCGGGGAGGAGCTGCTCACGTGGGCGCTCGACCCCAAGGCCGGCGCCTGCCTGCCGTCGTCCACGCGCACCGTCATGGCGCTCTCGGAGGTGGAGGCCTTCGCGCTGCACGCCGAGGAGCTCAAGTTCGTGGCGGGGCAGTTCCGCCGGATGCACAGCAAGGCGGTGCAGCACACGTTCCGGTTCTACTCCCAGCAGTGGCGCACGTGGGCAGCCACCTACATCCAGGCGGCGTGGCGGCGGCACCTCAAGCGCAGAGCGGCCGAGCTGCGGCGCAGGGAGGACGAGGAGCTGGAGGAGGACGAAGGCAAGTCCAACAGAATCAGGACCACCATACTGGTGTCGCGGTTCGCAGCCAACGCTATGCGCGGCGTGCACCGGCAGCGCTCCAGGCGGGCAGTGGCCGTGTCCGAGCTGCTGATGCCCATGCCCAAGCCGCGTGAGCCCGACTTCGGCGACGACTACTAACGCCGAGTTTGGCGGGTGTATGTACTCTACTAGTTTTATACAGGTAGCAAG

>ZMCNGC12

AATCAATGCAGATTTGAACGTCCTTCCAAGGACGCCCAAACGATCTCTGTACCACGCCTGCACGGCTGCACCTACCCCACTTGGAACTGTGGACGCCTGCTCATCTTTTTTCATCAGTAGTACCATATGTCATCAGTCAGTCAGTCATGCATGCCGCGTGCGTGACCATTTCTGTGCGTTCGCAGGTGGTCGTCTGGGTGGCGACGCCGGCGATGATACGCGCCGGGTCGACGACCGACGTCATGATCGTGCTGCTGACGGCGTTCCTGCTGGAGTACCTGCCCAAGATCTACCACGCCGTCCGCGTCCTGCGCCGGATGCAGGGCGTCTCCGGCTACCTCTTCGGCACCATCTGGTGGGGGATCGCGCTCAACCTCATGGCCTACTTCGTCGCCGCTCACGCGGTGGGCGCGTGCTGGTACCTGCTCGGCGCGCAGCGGGCCACCAAGTGCCTCAGGGAGCAGTGCGCCCAGGCCGGGAGCGGGTGCGCGCCCTGGGCGCTGGCGTGCGCGGAGCCGCTCTACTACGGCGCCACCGCCAGCAGCGTGGGGGCGGCCAGGCTCGCCTGGGCCGGCAACGCCACGGCCAGGGGCACGTGCCTCGACAGCGCCGACAACTACCAGTACGGGGCCTACCAGTGGACTGTCATGCTGGTGGCCAACCCCAGCAGGGTCGAGAGGGTTCTGCTCCCCATCTTCTGGGGGCTAATGACTCTCAGCACCTTTGGGAATCTGGAGAGCACGACGGAGTGGCTGGAGATCGTGTTCAACATCGTCACCATCACCGGCGGGCTGATTCTCGTGACGATGCTCATAGGGAACATCAAGGTGTTCCTGAACGCGACGACGTCGAAGAAGCAGGCGATGCACACGCGGCTGCGCAGCGTGGAGCTGTGGATGAAGCGCAAGGACCTGCCCAGGAGCTACCGGCACCGGGTGCGCCAGTACGAGCGGCAGCGGTGGGCGGCCACGCGCGGCGTCGACGAGTGCCGCATCGTCCGTGACCTTCCGGAGGGGCTCCGCCGGGACATCAAGTACCACCTCTGCCTCGGCCTCGTGCGCCAGGTGCCGCTGTTCCAACACATGGACGACCTGGTGCTCGAGAACATCTGCGACAGGGTCAAGTCCCTCATTTTCCCCAAAGGAGAAGTTATTGTCAGAGAAGGGGACCCAGTGAAGAGGATGCTGTTCATCGTGCGTGGCCACCTGCAGAGCAGCCAGGTGCTCCGCAACGGCGCCGAGAGCTGCTGCATGCTGGGGCCGGGCAACTTCAGCGGCGACGAGCTCCTGTCGTGGTGCCTGCGCCGGCCGTTCCTGGAGCGGCTGCCGGGGTCGTCTTCCACGCTGGCCACGCTGGAGAGCACGGAGGCCTTCGGGCTGGACGCCGCGGACGTCAAGTACGTCACGCAGCACTTCCGGTACACCTTCACCAACGACAAGGTGCGGCGCAGCGCGCGCTACTACTCGCCCGGGTGGCGCACGTGGGCGGCCGTGGCGGTGCAGCTCGCGTGGCGCCGCTACAAGCACCGCAAGACGCTCGCGTCGCTGTCGTTCATCCGCCCGCGCCGCCCGCTCTCGCGGTGCTCGTCGCTCGGCGAGGAGAAGCTACGCCTCTATACCGCGCTGCTCACGTCGCCCAAGCCCAACCAGGACGACCTGCTGTGACTGAGATGCATGCCTGCAGTCTGAACAACGCGCGTCTGTTCAGCGGAGTTGAAGCAAAATGCAGCGAGATGGAAGAAGCTGGTTCTCTATAGTAAAAGTGCATGTCAGAAAGAACTTGTTGGGCCGTGTCCCTCCGTGTATGTAGAGCCCAAGAGACATGTATATTTATGTGTACTGTATCAGCTGCAATAGAGAGGGTTCTAGAGACTTCAAAA

>ZMCNGC2

TATGTGTGTTTTTTACTCTTGACAATGTTTCACCCCTATTTGCAGACCTATTTGCAGTCAGCCTCTTTGAGAGTAGAAGAAATGAGAGTGAAAAGCCGTGACACTGATCAGTGGATGTCATATCGACATCTTCCTGAGAACCTCAAGGAAAGAATACGGCGTTATGAACAATATAGATGGCAAGAAACAAGCGGGGTTGATGAAGAGCAACTCCTTATGAACCTCCCCAAAGATCTTAGGAGGGATATAAAACGACATCTTTGTTTGAAACTTCTCATGAGGGTTCCACTGTTTGAAAATATGGACGAACAGCTTTTGGATGCCATGTGTGACTGCCTAAAGCCCATTCTGTACACAGAAGGTAGCTGCGTTATTCGCGAAGGAGATCCGGTGAACGAGATGCTCTTTGTCATGAGGGGAAACCTAATGAGCATGACGACGAATGGTGGAAGAACCGGCTTCTTTAACTCCGATGTTCTGAAGGCCGGAGATTTCTGCGGCGAAGAGCTCCTCACCTGGGCTCTTGACCCCACGTCAACATCGAGCCTCCCCAGCTCAACAAGGACGGTGAAGACGATGTCTGAAGTGGAAGCCTTTGCCTTGAGGGCTGAAGACTTGAGGTTTGTGGCCACCCAGTTCCGACGACTCCACAGCAAACAGCTCCAGCACACTTTCAGGTTCTACTCGCAGCAGTGGAGGACCTGGGCCGCCTGCTTCATCCAAGCTGCCTGGCACCGGTACTGCAGGAAGAAGATCGAGGATTCTTTGCGTGAGAAGGAGAAGAGGCTGCAGTTCGCGATTGCCAACGACAGCTCCACTTCGCTCAGCTTCATGGCAGCGCTGTATGCTTCGCGGTTCGCTGGGAATATGATACGGATCCTGAGGAGAAACGCCACGCGCAAGGCCAGGCTGCAGGAAAGAGTGCCCGCGAGACTGCTGCAGAAACCGGCTGAACCCAACTTTTCCGCAGAAGAGCAGTAGTATTTCGCTTCTCTGACTTCTCGTTTTGCAGATACAGATATGGGAAATTGTTTGCGTGACTTGCAAGCCCGAGGATGGCATGCCCAGATTAAAAAGAAGAAGAAGAAAAAGGGGAATGAAATCTCGATAGAATGACCACTGAGTGATAGTAATGTAGTGATTGATATACAGTACATTACTGTTCGTTTAAGAACGTCCAAGTGGCGTTTGTATGTGCTTGTGTAATAGCATAGTTAGATAGGAGTGATAGTAATGTAGTGATATACATTCATCCACCGAAGCTATGTGTGTATAGGGATCGACCATTTCCGTGCTTACACTCACGCACTGTAAACGCAGCTGGTGACTGCTAAGTGGCATCCATAAGTGCGCGCAAGAGATCAACAGAAACTGGCTAGGGTAGTTGGCATGTAGTGCTTGCACAGGAAGAAAGCGCTGAAATGGCTCCATGATTTCAAT>ZMCNGC8

GGCATGTTTGGGAGCAAAGGAAATGAAGGGGATTGGAGGGGCTAAAATCCCCTTGTTATTTAAAATTGAATAGCAAGGGAATTTTAGCCCCTCCAATTCTCTCCATTTCCATTGCTCCCAAACAAGCCCTTAAACGGAAGGTAGCATGATGAACTAATGGTTTTGCCTATCGGGTGAAGATGGTCCGTGGAATCCTATGTATTATTCTTCTATTGTTACTACTTTGGTTCGAAACCACACTATTTCGACCCAAAATCATTGGTTTTTGTTGTATTTATATTTCTTGCGCTTTTGCTATTTTTCTAACTTTCTGTTTATCTTTAGGATCTTTCCCGACGAGAGACAAAATCAATCTAAGTCATTGTATCAAACTACACGGGCTGACAGATTTGGCGCAAATAGAATAGATCTGAAGAATCCTGAGAAGCTTAAGGTGTTAAATGAAAGCAACAAACCCTGGCACCAGCGTATTCTAGACCCTGGAAGTAATATTGTACTGAGATGGAACAGGGTGTACCTTGTGGCATGTTTGTTTGCTCTTTTTATAGATCCTTTTTTCTATTACCTTCCATTGATTAGACAAAATGGCAATGGATCTTCATGTGTTGCCAAGGACCAGGGACTGAGCATAAGAATCACTGTCCTACGATCACTTGCTGACTTATTTTACATGTTGAACATAGCAATCAAGTTTCATACTGCATATGTGGATCCAAAGTCCAGAGTCCTTGGAAAGGGAGAGCTTGTTGTGGATATTAAGAAGATTCAACGAAGATATATAAGAACTGATTTCTTTGTAGACATACTTGCAGCTGTGCCACTTCCACAGGTTACTGTGTGGTTAATTATGCCTGCGATAAAAAGCTCAGATTATAACATCCGGAACACTACATTTGCTCTCGTAATTGTAATTCAGTATGTCATAAGAATGTATCTCATCATCCCTTTAAGCAATCAGATTATCAAAGCTGTTGGAGTAGTTGCAAAGTCAGCTTGGGGGGGAGCAGCATACAATCTTCTTCTCTACATGCTTGCAAGCCATATTACTGGTGCAATATATTACCTTCTCTCCATCGAACGGCAGATTACATGCTGGGATCAGCAGTGCGTTGCTGAGTACAATGATACACATTGCAACTTTAGTTTTATAAGCTGTGAGAATAATGGTTCTAATGATTATTCTGTGTGGGCAAATAAGACAAAAGTATTTGCCAACTGTGATGCCACGAATAGTAGTATATCATTTAACTACGGGATGTTTTCTAGTGCACTGAGTAAAGGTGCTGTATCATCTCCATTCCTTGAGAAGTATTTCTTTTGCCTATGGTGGGGCTTGCTGCAGCTTAGTTCAAGCGGAAATCCTCTCGTGACAAGTGCATTTATCACAGAGAATGCATTTGCGATAGCAATTGGTGCTATCAGTCTCATACTCTTTGCTCAGTTGATTGGCAAAATGCAGACATACCTGCAGTCTATCAGTAAAAGGCTTGAAGAGTGGAGGCTGAGGCAAAGGGACATGGATGAGTGGATGAGACACCATCAACTCCCATCTCATCTTCAAGAACGTGTGCGGCGGTTCGTTCAAGTCAAATGGCTTGCTACAAGAGGAGTAGAAGAAGAATCCATCTTGCAAGCTTTGCCTGCTGATATTCGTCGGGATGTGCAGCGTCATCTTTGTTTGGACCTCGTTAGACGTGTACCTTTTTTCTCTGAGATGGATAACCAACTTCTCGATGCCATCTGTGAGCGGCTGGTGTCTTTCCTGTGCCCTGAGAACACGTACATCTCTCGCGAGGGTGATCCTGTGAACGAGATGCTCTTCATTATACGCGGGAAACTAGAGAGCTCAACGACAAATGGTGGCCGCAGCAACTTCTTCAACTCCATCATCCTGCGCCCCGGCGATTTCGCAGGCGAGGAGCTGCTCACGTGGGCCCTGCTCCCCAAGACCAACGTCCACTTCCCGCTCTCGACAAGGACCGTACGGAGCCACACAGAGGTGGAGGCCTTCGCTCTGCGGGCTGAGGACCTGAAGTTCGTCGCGAACCAGTTCCGTAGGCTCCACAGCAAGAAGCTCCAGCACACGTTCCGGTTCTACTCCCACCACTGGAGGACCTGGGCCGCCTGCTTCATCCAGGCCGCTTGGCGGCAGCACCAGAGGAGGAAGCTGGCCGAGAGCCTCAGCCGCTGGGAGTCGTACTCGTGGTGGTCGGCGGAGGACCACCCAACCGGCGATAAGCCGAGGCAGGAGGGCACCTCGAGCGGCGGCGGCGGCACGAGGACGATCGCTGAAGGTGCCATCGCCCATATGCACAAGCTCGCCTCTGCTTCCAGAAGGTTCCGCACCGAGGACGTCGCTATCCGCAGGCTGCAGAAGCCTGACGAGCCCGATTTCTCCGCGGACCATTTTGATTGAACCTTCTTAATATTGTGCTTCGCAGCTGGGAGTTGCATGTTGTACGAACGATCGCAGCCGTAGCATATATACTCTACTGTAATTTTACTGCACAGCCATCTTAGGTGTCGTGAATTGTAATAGAAAAAGAAGATGTATCGTGTAATTCTAAATCCAGAATCTACCCTGTATAGGCTTAACCTTTTGTGCAGTGCATAAACTTGAGTATTTCGAAGCGTCATATGTGACTTGTT

>ZMCNGC9

TGATACTGCTAGTAGTTCCTAAAGTTGGGTTATCTGCTGCAAACTATGCTAAGAATTTATTGCGTGTCACTGTTCTTCTTCAATATGTCCCCCGTATCATCAGATTCGTACCACTTCTTGATGGTCAGTCCACCAATGGATTCATATTTGAGTCAGCATGGGCTAATTTTGTGATCAACCTTCTAATGTTTATTTTGGCGGGACATGTGGTTGGTTCATGTTGGTATCTCTTTGGCTTACAGAGGGTTAACCAATGTCTACGAGATGCTTGTTCTATATCGACCATTCCATATTGTGATTCTTTTATAGACTGTGGACGTGGCATTGGGAGTGGACTGTACAGACAGCAGTGGTTCAATGACTCGGGTGCAGAAGCTTGTTTTAACACTGGAAATGATGCTACTTTCCAATATGGAATTTATGAGCAGGCTGTTTTGCTCACTACAGAAGACAGTGCTGTAAAACGATATATATATTCATTATTTTGGGGGTTTCAGCAAATAAGTACCTTAGCAGGAAACCTTGTCCCGAGTTACTTTATATGGGAAGTTCTGTTCACGATGGCTATTATTGGTCTGGGACTGTTGCTTTTTGCATTGCTTATTGGAAACATGCAAAATTTTCTGCAAGCTCTTGGAAGAAGGAGGTTGGAAATGCAACTCAGGCGCCGTGATGTTGAAAAGTGGATGAGCCATAGGCGATTGCCTGAAGATTTGAGAAGGAGGGTTAGACGAGCCGAAAGGTTCACCTGGGCAGCTACTCAAGGAGTGAATGAAGAGGAGCTTTTGAGTAATTTACCTGAAGACATCCAAAGGGACATACGTCGCCACTTCTTTAGATTCCTTAATAAGGTCCGATTATTCACCTTGATGGATTGGCCTATATTGGATGCAATATGTGACAAATTAAGACAAAACTTGTATATTAGTGGAAGTGACATTCTTTACCAAGGTGGCACTGTTGAAAAGATGGTCTTCATAGTGAGAGGGAAGCTGGAAAGCATCAGTGCAGATGGCAGCAAGGCTCCATTACATGATGGAGATGTATGTGGAGAGGAGCTCCTCACGTGGTACTTGGAACACTCTTCAGCGAACAGAGATGGTGGGAAAATTAAATTCCAAGGTATGCGGTTGGTTGCTATACGTACAGTAAGATGTTTAACAAATGTTGAAGCTTTTGTACTCAGAGCAAGTGATCTGGAAGAAGTCACCTCGCAGTTTGCTCGATTCCTGCGTAATCCACGAGTGCAGGGAGCGATCAGGTATGAATCCCCCTACTGGCGAACCATTGCTGCAACTCGTATTCAGGTTGCATGGAGGTATCGTAAAAGGCGGCTGAAGCGAGCTGAGAAGTCGAGGTTGAGCGAAGAGACTTATGCCTCACTTGGATCTTGACACATGATTCTTTTCAGCGTAGACGGAGGGGATGATTGATCGCTACCTGACTAGGCACTAACTTTTCAGTTTCAGTTCAGCATCTAAAGCTTCTAGAGCTGTGAAATTTCTCCGAACATGTCTACTTGGTGGAACCTAACGGGGCAAATATTTTTGGCTTTGGTGTTCCGTGTACATAATAAGAGGAAAGTTTTTTTTTTC

>ZMCNGC1

TTTTTTAAAAAACGAGCCCGACCTTGCTCCAACTCCCTCGATTCCCTCTCCCTATTCGCGTTCCCCACTTCCGTTCGCCGCCGACACCGCGTGCGCTCTCTCCCCCCCTCCGTCTCTGTCTGCGACGACTGGGTGGGCTACCGCTGCCGCCCTACCCATGTGCTGAGGCTTCTGCCTACCTCTTCGCCGGCGAAGAGCGCCCAGCAGATCCGTCCTCGCTTCCACGCTGCAGTTGAAGATTTGATCAGCTATCGCCTTCGATGCAGTTGAATATTTGATCAGCTATTGACTTCGCTGCTGGCCTGTTTTCTCCTTTGGAGACCAAAGAAGCCATGGCGGGCCGGGAGGAGAGATATGTGAGGTTTCATGACTGGAAATCAGAGCAATCTGTTTCTGTTATTTCAGATAGGGTAGTATCAGAAAAAGGGCATAACATCTTTGGCTTGTTAAAGGACAGAACAGCAGGAGCCTTTTCATTCCTGGGGAACTCTTCACATTCTGAAGCCCTAAACAAATTAGGCCTAGGGGAGAAGTCAAAAACAAAAGTTCTTGATCCTCAAGGGCCATTTTTGCAGAGATGGAACAAGATATTTGTGATATCATGTCTTTTTGCAGTTTTTGTGGACCCATTGTTCTTGTATGTCCCAGTAATTGATGGTGGCAACAACTGCCTGTACTTGGACAAGAAGTTAGAGACCACAGCAAGTATCCTGCGCTTTTTCACAGATATCTTCTATTTACTCCATATACTGTTTCAGTTCAGAACAGGCTTTATTGCTCCCTCTTCTAGAGTGTTCGGTCGGGGTGCCTTGGTTAAGGACACATTTGCAATAGCAAAGCGATATCTATCAACATTGTTCCTGGTGGATTTCTTAGCGGTTCTGCCCCTCCCTCAGGTGTTTGTGTTGGTGGTGCTGCCTAAGCTCCAAGGTCCTGAAATTATGAAGGCAAAAATTGTACTGTTGGTTATTATTATTTGTCAATATGTGCCTCGACTGCTCCGAATAATACCACTTTACCTTCAAATCACAAGATCTGCTGGCATACTTACAGAGACAGCATGGGCTGGTGCTGCTTTCAACCTTATAATTTATATGCTTGCCAGTCATGGCTTTGGAGCTCTTTGGTACATTCTTTCCATCCAGCGAGAAGACACCTGTTGGAGACAAGCATGTATCAATCAGACTGGCTGTGATCCTACATCTTTGTACTGCGGGTATCATTCACTTGCAAATAATTCTTTCTTACAAAATGCGTGCCCAACAAATAGCACTGCCAATCCAGACCCTATATTTGGAATCTTTCTACCAGCTCTCCAAAATGTTTCACAATCGACGAGTTTCTTTGAAAAACTATTCTATTGCTTTTGGTGGGGGCTACAAAATCTAAGTTCCCTTGGCCAGAACATGAAAACAAGCACTAATACATTGGAGAATCTGTTTGCTGTTTTTGTCTCGACATCGGGTTTGGTTCTATTTGCACTACTTATTGGTAATGTGCAGACCTATTTACAGTCAGCTTCTGTGCGTATCGAAGAAATGAGAGTGAAAAGGCGTGATACAGAGCAGTGGATGGCACATAGGCTACTCCCTGAGAATCTAAAGGATCGGATTATGCGCCACGAACAATATAGGTGGCAAGAAACAAGAGGGGTTGACGAAGAGGGCCTTCTTAAAAATCTTCCAAAGGATCTTAGAAGAGAGATAAAGAGACATCTTTGTTTGTCACTTCTCATGAAGGTTCCAATGTTTGAAAACATGGATGAACAGCTGTTGGATGCCATGTGTGATCGTCTAAAGCCTATGCTGTACACAGAAGGAAGCTGCATCATTCGCGAAGGTGATCCAGTGAATGAAATGCTCTTCATCATGAGAGGAACACTAGAGAGTACCACAACAAATGGTGGGCAAACTGGTTTCTTCAACTCTAATGTTCTAAAAGGTGGAGACTTCTGTGGTGAAGAGCTCCTCACGTGGGCCCTTGACCCCACTTCAGCTTCAAATCTTCCTGGCTCAACTAGGACAGTGAAGACGTTGTCTGAAGTCGAAGCTTTTGCTCTGAGGGCTGACGACTTGAAGTTTGTTGCCACACAATTTAGGAGGCTCCACAGCAAACAACTTCAGCATACCTTCCGGTTTTACTCACAGCAATGGAGGACCTGGGCTGCTTGCTTCATACAGGCAGCTTGGCACAGATACTGTAGGAAGAAGCTGGAAGAGGCTTTATATGAGAAGGAGAAGAGGTTACAAGCAGCAATTGTAAGTGATGGCACTACTTCGCTCAGTCTCGGTGCAGCGCTCTATGCTTCACGTTTTGCTGGCAACATGATGCGGATCTTACGGAGAAATGCCACCAGAAAGGCCCGTTTGCAGGAAAGAGTACCTGCAAGACTGTTGCAAAAGCCAGCAGAACCCAACTTCTTCGCTGAAGATAGCTGAACTTGTACCCTGTAGCAAGCAGGGATTCATGGTCCAACCGGTGAAGTTTGTGCAGAAGTTAAGATTGGATGCTGTAGATAGACAGATCAGGTGCTCTAAAGCCGTCTGGTTTAAATCACCTAATTCGCGAAAAGGCCTGGTCCGAGTTAGATTCTCCGTTGAAATAAATTTGAAGCTGACGTTAAAAAAAAAATCCGCTTTTGCTCCACGTTAAATTCTGGCTTCCATAGTGGTCGGACTAGGGTGCTGAGATAAGGGTATTGAGATTTTTTAGATCTGTGTGAAAAGATTTTCTGTAACGTAATATCTGAAGGCTGTCTTATCCTCTGTAGGTTATTTTAAAAAAAACACGTATATTTCGATTGTATACAGGTTTCATGTATTATAAACACTCTAATTTACCTTGTGCATTTGAAAAAAGAGAGAAGATGCTGTGAATATAATCGATTAATTGTTTGT

>ZMCNGC10

ATGCCTCCGCTCGCATTCCTCCGCCGCTACCTCCCCGCGAGGAACCGAGTGTCTGTTATCGATCCTGAGTTGCGTCTGTCTGTTTGTTTCCCTGAGCAAAGGCTTCTCGCGCGAGCGTGCGATGGTGGAGTCCGGGGGAGCCCGGGCGTGGCGCGGGACGAGGAGGCCGGAGGCAGCGGCGGACTGAGCGGCCGGTCGGCGGGGGCGCCGTCCGGGGAGTGCTACGCGTGCACGCAGCCCGGGGTGCCGGCGTTCCACTCCACGGCCTGCGACCAGGTGCACTCGCCGGACTGGGACGCCGACGCGGGGTCCTCGCTGGTGCCGGTCCAGGCGCAGCAGCAGGCCCAGCCGGCGGCGGCGGCGGCGCAGCACGCGGCGCGGTGGCTGTTCGGGCCCGTGCTGGACCCGCGCAGCAAGCGCGTGCAGCGCTGGAACCGCTGGATCCTGCTCGGCCGCGCCGCCGCGCTGGCGCTGGACCCGCTCTTCTTCTACGCGCTCTCCATCGGCCGCGCCGGCCGGCCCTGCCTCTACTTGGACGCCGGCCTCGCCGCCGCGGTCACCGCGCTCCGGACCTGCGCCGACGTCGCGCACCTCGCGCACGTGCTCCTGCAGTTCCGCCTCGCCTACGTCTCCCGCGAGTCCCTCGTCGTCGGGTGCGGCAAGCTCGTCTGGGACGCCCGCGCCATCGCCGCGCACTACGCCCGCTCCGTCAAGGGCCTCTGCTTCGACCTCTTCGTCATCCTCCCCATCCCGCAGGTCATCTTCTGGTTGGTTATACCAAAGTTAATTAGGGAAGAACGTGTTAGGCTTATCATGACGATACTGCTACTCATGTTCATATTTCAATTTCTCCCCAAGGTCTACCATAGTATACACATCATGAGGAAAATGCAGAAGGTGACGGGTTACATCTTTGGATCGATATGGTGGGGATTTGGTTTAAATCTATTTGCCTATTTCATTGCTTCTCATATTGCAGGTGGGTGCTGGTATGTTCTTGCAATCCAGCGCATTGCTTCCTGCCTCCAGGAAGAATGCAAGAAAAACAATAGTTGTGATCTAATATCACTAGCTTGTTCGAAGGAGATATGCTTTCACCCTCCTTGGTCTTCGAATGTTAATGGGTTCGCATGTGATACGAACATGACCTCCTTTAGTCAACGAAATGTGTCTACTTGTTTAAGTGGTAAAGGGTCGTTTGCTTATGGAATCTATTTGGGGGCTCTTCCTGTTATATCGAGCAATTCGCTTGCTGTCAAAATTCTCTATCCTATATTTTGGGGACTCATGACACTCAGTACTTTTGGTAACGATCTTGCCCCAACAAGCAATGGTATTGAGGTGATATTCAGCATAATCAATGTCCTCAGTGGCCTGATGCTCTTCACATTGCTGATCGGAAACATACAGGTATTTCTGCACGCGGTCCTGGCAAGGAAGCGGAAGATGCAGCTGCGGTTCCGAGACATGGAATGGTGGATGAGACGGAGGCAGCTGCCGTCTCGGCTGAGGCAGAGGGTGCGCAAATATGAGCGCGAACGCTGGGCCGCCGTCACGGGAGACGAGGAGATGGAGATGATCAAGGATCTGCCTGAAGGACTGAGGCGGGACATCAAGCGCTACCTCTGCCTCGAGCTGGTTAAGCAGGTTCCGCTGTTCCATGGCATGGACGATCTGATCCTGGATAACATCTGCGACCGGCTGCGGCCACTGGTGTTCTCCAGCGGGGAGAAGGTGATCCGAGAGGGCGACCCCGTGCAGCGCATGGTGTTCATCCTGCAGGGCAAGCTCCGGAGCACGCAGCCGCTGACCAAGGGCGTGGTGGCAACGTGCATGCTAGGGGCGGGCAACTTCCTAGGCGACGAGCTGCTGTCGTGGTGCCTGCGCCGCCCCTTCGTGGACCGGCTCCCCGCGTCGTCGGCCACGTTCGAGTGCGTGGAGGCGGCGCAGGCGTTCTGCCTCGACGCGCCGGACCTGCGGTTCATCACCGAGCACTTCCGCTACAAGTTCGCCAACGAGAAGCTCAGGCGCACGGCGCGGTACTACTCGTCCAACTGGCGGACGTGGGCCGCCGTCAACATCCAGCTCGCGTGGCGCAGGTATAGGGCCCGGGCATCGACGGACCTGGCGGCGATGGCCGCGCCGCCGTTGGCGGGCGGACCCGACGACGGGGACCGGCGGCTCAGACACTACGCGGCCATGTTCATGTCGCTCCGGCCGCATGACCACCTAGAGTGATCAGGAGGGGGGACGGGACCATCCTAGCTGTGCCGGCCGGGTCATGGTGTCTGTACAGTGTACACTAGTGGTATGTTGTTGTCATCTTCTGCGTGAGTGAACTGGTGGTTCGGGATTTGTCATTTAAAGAAGGTCAATAATGGAGAAATAGTTTCTTAGCCCGATTCAATTGTCTTCCTTTCACCAAGAATAAAAATTACTTCTGC

**4.CDS sequence**

>ZMCNGC6

ATGGACTGCGATTTGTTCGCTGCGTGGTGGAGCAGCAGCACCAGATTAGTCTCCAGGATTTTTCGGGGGTCAGCTGATGCCCCGGGCCCGTCGCCAGCGAGGCCAGCCATGCCGCTTCACCAGAAGCAGGCGGGGCTCGCTGCTAGCAAGCTGGGCGTGGGGACCTCGAAGAAACACAGGGCTTTTGTCGCAAGCGATGAGCAGTGGTACAACAAGATTTTTGATCCGTCAAGCGACTTCATCTTGACATGGAACCGCATTTTCCTCTTCTCCTGCTTCGTCGCGCTATTTATAGACCCCCTCTATTTCTATGTGCCCAAGATCAGCTACGGCAGCCCCAAATTCTGTATCGGAACAGACACCCGTTTCGCCGTCGGTGTTACATTCTTCAGATCGATTGCGGATTTATTGTATGTCCTGCACATCATAATAAAGTTCAGAACAGCATATATCAATCCAAGCTCGACTCTGAGGGTGTTTGGAAGAGGGGATCTTGTCACAAATCCCAAGGAAATTGCGTGGAAATATATCAGATCTGACTTAGCTGTTGATGTGGCAGCTGCTTTGCCTTTGCCACAGATTATTGTCTGGTTTGTGATACCAGCTATAAAGTATTCCTCTGCTGAGCACAACAATAACATTCTGGTGCTCATAGTTCTTGCTCAGTATCTTCCAAGATTGTATCTCATATTCCCCTTAACTTATGAAATTGTCAAAGCTACTGGAGTTGTTGCAAAGACTGCTTGGGAAGGAGCTGCATACAACATGGTGCTCTATCTGATAGCTAGTCATGTGCTAGGTGCACTGTGGTATTTGCTATCTGTTGATCGCCAGACATTCTGCTGGAAGACGAACTGCCTGAATGAAACTGGTTGTGATCTTAAGTACCTAGATTGTGACACGACACCAAATGCTACATGGGCGAATACGACTGCTGTCTTCAGTAATTGTAATGCTAGCGATACCAATATAAGTTTTGATTTTGGCATGTTCGAGCCTGCATTGTCTAATCAAGCCCCTGCTCAAAGTTTTGCGATGAAGTATTTCTATTCCCTCTGGTGGGGATTGCAGAATTTAAGCTGCTACGGTCAGACTCTTACTGTGAGCACCTATCTTGGCGAGACGCTGTATTGTATATTCTTGGCGGTACTTGGTCTTGTCTTGTTTGCGCATTTGATTGGAAATGTGCAGACCTACCTGCAATCTATTACTGTGAGGGTTGAGGAATGGAGATTAAAGCAAAGAGATACTGAGGAATGGATGAGACATCGTCAGCTTCCTTGTGAACTGCGGGAAAGGGTGAGACGATTTATCCAGTACAAGTGGCTTGCAACAAGAGGAGTGAACGAAGAGTCAATATTGCATGCTCTGCCTGCAGACCTTCGACGTGACATTAAGCGCCACCTTTGCCTGGGTCTTGTTCGACGGGTTCCTTTTTTCTCCCAGATGGATGATCAGCTTCTTGATGCCATCTGTGAGCGTCTTGTATCATCACTGTGCACAAAAGGCACATACATTGTCCGTGAGGGTGATCCGGTGACAGAGATGCTCTTCATCATCCGTGGAAAACTGGAAAGCTCCACAACAAATGGTGGCCGCACTGGCTTCTTCAATTCAATCACCCTGAAACCCGGTGATTTCTGTGGCGAGGAGCTTCTTGGATGGGCTCTTGTCCCCAGGCCTACTACAAATTTGCCGTCATCCACTCGGACAGTGAAGGCACTGATAGAAGTAGAGGCCTTTGCGCTCCAGGCGGAGGATCTCAAGTTTGTTGCCAGCCAGTTCAGGCGGCTGCACAGCAAGAAACTGCAGCACACTTTCCGGTACTACTCGCACCACTGGAGGACGTGGGCCTCATGCTTCATCCAAGCTGCCTGGAGACGGTACAAGCGAAGGAAGATGGCAAAGGACCTGAGTATGAGGGAGTCATTCAACTCCGTTAGATTAGACGAAGTGGATAACGAAGATGACGATTCTCCGCCCAAGAATAGCCTTGCTCTAAAATTCATAGCTAGGACTAGAAAAGTGCCTCAGAACATGAAAGAGTTGCCGAAGATAACGAAGCCAGACGAGCCAGATTTCTCAGCTGAACCCGAAGACTAA

>ZMCNGC4

ATGTTTGACAGTACTCAAAAAGCTCAATACATGGATGGGCACAGAGAGAGGTTTATTAGGCTGGATGAGTCAAGTCCTAGGTCATCTGTACCTTCTGAAGTGGGAGGCAGGAGTACATTGAGGTCCAGCATGCCTGGGTTTGGTTATGGTCCATTTAATGCACTAAGGTCTTTCTTGTCTGGGGGCTCTGGAAGACTGAAGTCACTTAGACAGTCACTTACTTCTGGTGCTCCTAAGACAGCTTTTGCGGAAGATCTTAAATCATATAAGAGAACTATATTTGATCCCCAGGACAAACTTCTTTTCCGAATGAACTGGGTTTTCTTCTCGTCTTGTCTTTTTGCTGTTGCAGTGGACCCACTATTCTTCTTCCTACCCATCATCAACGATTCAAACTGCATTGGTATTGATAAAAAGTTGGCAGTGACATCAACAATAATACGTACGGTTATTGATTTTGTCTACCTTATACGCGTGTGTCTTCAATTCCGCACTGCTTATGTTGCTCCATCTTCACGCGTGTTTGGGACTGGTGAGCTTGTGATTGATCCGATGCTAATTGCAAAGCGATACATTAAAAGTTACTTTGCAATGGACTTTGTTGCATTGCTACCACTTCCACAGATTGTTGTATGGAGATACCTCCATATTCCAGATGGCCCAGATGTACTGACTACGAAAACTGCACTGGTTTGGGTTGTTTTGATCCAATACATTCCAAGGTTGCTTCGAATATTCCCTGTGATCACAGATTTGAAAAGGACAGCTGGTGTTTTTATTGAAACTGCTTGGGCTGGTGCTGCTTATTATCTTCTATGGTTTATGCTGGCTGGGCATAATGTTGGTACTTTATGGTACTTTTTAACCATAGAACGTGAAGATGATTGCTGGCATCTATACTGTGACGATCCCAACTTTGGCTTGGGATGTAATAGCAGCTACTTGTATTGCAATAATCATCATCATGGCAGCTATGATAGTTGGCTTACGAATAATAGTGCCCAAGTATTCAACATGTGCAATGGCGGTCAAGACAATCCTTTCAACTTTGGCATTTATGAGCAAGCACTGGTCTCTAAAATACTTAGTCCAGGAAATTTCATCTCAAAATTATGTTATTGCTTCTGGTGGGGATTGCAAAATCTAAGTACACTTGGTCAAGGGCTTTTGACAAGCACATACCCTGGAGAAGTCCTGTTCTCTATAGCAATATGTGTCCTTGGACTAATTCTTTTTGCTCTCCTCATTGGTAACATGCAGAGCTACCTACAATCTGTTGCTATACGCCTTGAAGAGATGAGAGTTAAGAAACGCGATGCTGAGCAGTGGATGCATCACCGTTCACTGCCACTGGACATCAGACATAGGGTGAGGAAGTATGAACGTTATCGGTGGTTGGAAACCAGGGGAGTAGACGAAGAAACTTTGGTTCAAACTCTTCCAAAAGATCTTAGGAGGGATATCAAGCGGCATCTTTGTTTGGGCTTAGTGAAAAGGGTGCCTTTGTTTGAAAATATGGATGAACGATTGTTGGATGCAATATGTGAGCGGTTAAGACCTGCACTCTACACAGAAAATGAGTTCATTTTGAGGGAAGGTGACCCGGTGGATGAGATGCACTTTATTCTTCATGGTTGTTTGGAGAGTGTAACCACTGATGGTGGACGGAGTGGGTTCTTTAACAAGGTTCAGCTAAAGGAAGGGTCGTTCTGTGGCGATGAGTTGCTCACTTGGGCATTGGATCCCAAGTCAGCTGCTAATTTTCCAGTTTCGAGCAGGACTGTCCAGGCACTCACCGAGGTTGAGGCGTTTGCCCTATGTGCAGAAGAGCTGAAATTCGTGGCCAGTCAGTTCAGGAGGCTGCACAGCAGGCAAGTGCAACACACATTCCGATTCTATTCCCAGCAGTGGAGGACTTGGGCAGCCTGCTTCATCCAAGCAGCATGGCGCCGCTACTACAAGAGGAAGATGGCAGAGCAGCGGCGCAAAGAAGAAGAGGCGGCAAGCCGGCCAAGTAGTAGCCACCCTAGCCTTGGGGCGACTATCTATGCATCTCGTTTCGCTGCCAACGCCATGCGAGGGGTTCACAGGCTAAGAAGCAAGGCTGTCCCTACCATTGTCAGGCTACCGAAACCCCCAGAACCAGATTTTGGTGTCGATGATGCTGACTAA

>ZMCNGC3

GTCTCCAAATTTAATTGTTTTCTTTTTCTTGGCTGGATTGAATTATTGTTGCAGACCTATCTACAATCAGCCTCGGGGCATATAGAGGAAATGAGAGTGCGAAGACGTGACATGGAGCAATGGATGTCATACAGACTACTTCCAGAGCATATCAAGGAACGAATACTGCGTCACCATCAATATCGGTGGCAAGAAACACAAGGCGTGGATGAAGAGGGCCTTCTTGTAAACCTTCCCAAGGACCTCAGGAGGGATATCAAGCGTCATCTTTGTCTGTCACTTCTCAAGAGGGTTCCAATGTTCGAAAACATGGACGACCAACTCCTGGACGCAATGTGCGACCGTGTGAAACCCATGCTGTACACAGAAGGAAGCCACATCGTTCGCGAAGGCGACCCAGTGAACGAGATGTTCTTCATCATGAGAGGGAGGCTAGAGAGCACGACAACGGACGGGGGGCGAGCGGGCTTCTTCAACTCCAACGTCCTCGAAGGCGGCGACTTCTGCGGCGAGGAGCTCCTCACGTGGGCCCTGGACCCAGCCTCGGGCTCCAACCTTCCAAGCTCGACGAGGACGGCGAGGACGCTGTCGGAGGTGGAAGGCTTCTCCCTGAGGGCTCGCCACCTGAGGTTCGTGGCCAGCCAGTACAGGCGGCTCCACAGCAAGCAGCTCCGGCACACCTTCAGGTTCTACTCCCACCAGTGGCGGACCTGGGCCGCGTGCTTCGTACAGGCGGCTTGGCACAGGTACTGCCGGAGGAGGCTGGAGGAGGGCGTGCGCGAGAAGGAGAGGATGTTCCGGGCAGCGGCCGTGACCGACATCTCCAGCTCCCGCAGCCTCGGCGCCGCGCTCTACGCTGCCCACTTCGCTCGCAACATGGTAAGGACGCTGCGGAGGAACGCCGCACGGAAGGCCCGTCTGCTGGATACAGTGTCTTCGAGGCTGTTGCAGAAGCCAGCGGAACCCAACTTTTTCGCTGAAGAAGACTGA

>ZMCNGC7

ATGGCATCCGGTGCTTCACGAAATGTCAGGTTCCAGAACGAGATCGAGGTCCAGAGCTTCAGAACAAGCCCTCTGCAGAGCCTCAGCAGAAAGCACGGCAAGGCTCACGATCCCAGGAAATGCCGGCTGGGTTTCCGCGGCGGCTGCCTGGAGAAGGCGTGCCGGAACCGGAAGCCGATGCTGAAGGACAGGGTGCTCTCGCGCGCCTTCTCGGAGGAGCTGGAGTCCCTGATGCACGCCGCCGGCGGCAGCCACCTCTTCTTCGACCCGCGCGGGCAGCTGATCCACCTGTGGAACAAGATCTTCCTGTCCGCCTGCCTGCTGTCGCTGTTCGTGGACCCGCTGTTCCTGTACCTGACGGGCACGCAGCGGAACACGTGCGTCGAGTTCAAGGACTCGCTGGCGCTCACGCTGTCCATGGTCCGCTCGCTGCTGGACCTCTTCTACGCCGCGCACATCCTGTTCCGCTTCCGCACCGCCTTCATCGCGCCGTCGTCGCGCGTGTTCGGGCGGGGCGAGCTCGTCATCCAGCCCTACGAGATCGCCAGGAGGTACCTTGGCCGGACGTTCTGGTTCGATCTCGTCACGGCGCTGCCCTTGCCGCAGTTCGTGATCTGGATCGTTATACCAAGGCTGAATGAGTACTCCCGGACGGCGAACACGAAGAACATCCTCCGGTTCAGCATCATCTTCCAGTACCTCCCGCGGTTGTTCCAGATATTCCCGCTCTCGGGGCGGATCGTCATGGCGACGGGGGTCATGACGGAGACGGCGTGGGCCGGCGCCGCGTACAACCTGATCCTCTACATGCTCGCAAGCCACGTGCTGGGAGCGCTGTGGTATCTCTTCTCCGTGCAGAGGCAGGAGGCATGCTGGAGGGAGGCGTGTCTGCTCGTGAGCCCGACGTCCCAGACCATGTTCTTCGACTGCAAGGCGTTGAGCAGCAACAGGACGATCTGGTATGAGCTGAGCAACATCACAACAAGCCGGTGCACGCCTGGCAACGGCTTCTACCCGTTCGGTATCTACGAGGAGGCGCTGTACGCCAAGCTCACGTCGTCGTCTTTCACCCAGAAGTACTTCTACTGCTTCTGGTGGGGACTCAAGAACCTCAGCTCCTTAGGACAGAATCTGTCGACGAGCTTGTTCATCGGTGAAATAACCTTCGCGATCGTCGTCGGCGTTCTTGGGTTAGTGCTGTTTGGCCTGCTCATCGGCAACATGCAATCTTACCTCCAAGCAACGATGGTGCGGCTGGAGGAGTGGCGGACGAAGCGGACGGACATGGAGCGGTGGATGCACCACCGGCAGATCCCCCAGCCGCTGAAGCAGTGCGTCCGGAGGTACCACCAGTACCAGTGGGTGGCCACGCGCGGCGTCGACGAGGAGGCCTTGCTGCAGGACCTCCCCATGGACATCCGCCGCGACATCAAGCGCCACCTCTGCCTCGACCTCGTCCGGAGGGTGCCCCTGTTCGACGAGATGGACGAGCGGATGCTGGACGCCATCTGCGAGCGGCTGAGGCCGGCGCTGTACACGCGCGGCACGCGGCTGATGCGGGAGCTGGACCCCGTCGACTCCATGCTCTTCATCATCCGGGGCTACCTCGACTCGTACACGACGCAGGGCGGCCGCTCCGGCTTCTTCAACTCGTGCCGCATCGGCGCCGGCGAGTTCTGCGGGGAGGAGCTCCTGACGTGGGCGCTCGACCCGCGCCCCGCGGCGAAGCTGCCGCTGTCCACCCGGACCGTGCGCGCCGTGTCCGAGGTCGAGGCGTTCGCGCTCGTGGCCGACGACCTCCGCTTCGTGGCGTCGCAGTTCCGCCGCCTGCACAGCGCGCGCATCCGCCACAGGTTCCGCTTCTACTCACACCAGTGGCGCACGTGGGCCGCGTGCTTCATCCAGGCCGCCTGGCGGCGATACAAGCGGCGCCGCGCGTCCATGGAGCTCAGGGTGCGCGAGGTGCGGGCCGGAGGGAGCTTGCTGCGGTCCCGCCGCCACAGCATCGAGGGCAAGGCGTCGATTAGGAAACCCATGGAACCGGACTTCACGGTGGAAGAAGAGGACTGA

>ZMCNGC11

ATGCCTCGCCTCGCATTCCTCCGCCGCTCCCTCCCCGCGAGGCTTCTCGCGCGAGCGTGTGGTGGTGGTGGTGGTGGAGACCAGGGGAGCCCGGACCAGGTGGCGCGGGACGAGGAGGCCGGAGGCAGCGGCGGAATGAGCGGGCGGTCGTCGGCGGGTGGGCCGTCCGGCGGGGAGTGCTACGCGTGCACGCAGCCCGGGGTGCCGGCGTTCCACTCCACGACGTGCGACCAGGTGCACTCGCCGGACTGGGACGCCGACGCGGGGTCCTCGCTCGTGCCGGTCCAGGGCCAGGCGCAGGCGGCGGCGGCGGCGGCGGCGCCGCGGCAGCGGCACGCGGCGCGGTGGCTGTTGGGGCCCGTGCTGGACCCGCGGAGCAGGCGCGTGCAGCGCTGGAACCGCTGGATCCTGCTGGGCCGCGCCGCCGCGCTGGCGGTGGACCCGCTCTTCTTCTACGCGCTCTCCATCGGCCGCGCTGGCCAGCCCTGCCTCTACATGGACGCCGGGCTCGCCTCCGCGGTCACGGCGCTGCGGACCTGCGCCGACGTGGCGCACCTCGCGCACGTGCTCCTGCAGCTCCGCCTCGCCTACGTCTCCCGCGAGTCCCTCGTCGTCGGGTGCGGCAAGCTCGTGTGGGACGCCCGCGCCGTCGCCGCGCACTACGCACGCTCCGTCAAGGGCCTCTGCTTCGACCTCTTCGTCATCCTCCCCATCCCGCAGGTTATCTTCTGGCTGGTTATACCAAAGTTAATCAGGGAGGAGCAGGTTAAGCTTATCATGACAATACTGCTGCTCATGTTCATATTTCAATTTCTCCCCAAGGTCTACCATAGTATACACATCATGAGGAAAATGCAGAAGGTGACAGGTTACATCTTTGGATCAATATGGTGGGGATTTGGTTTAAATCTATTTGCCTATTTCATTGCTTCTCATATTGCGGGTGGGTGCTGGTATGTTCTTGCGATCCAGCGCATCGCTTCCTGCCTCCAGGAAGAATGCAAGAGAAACAACAGTTGCGATCTAATATCGCTAGCTTGTTCCAAGGAGATATGTTTTCACCCTCCGTGGTCATCGAATGTTAACGGATTCGCGTGCGATACGAACATGACCTCCTTTAGCCAACAAAATGTGTCTACTTGCTTGAGTGGAAAAGGTTCCTTCGCTTATGGAATCTATTTGGGGGCTCTTCCTGTTATATCGAGCAATTCGCTCGCTGTCAAAATACTCTATCCTATATTTTGGGGCCTCATGACACTCAGTACTTTTGGTAACGACCTTGCCCCAACAAGCAATGGCATTGAGGTGATATTCAGCATAATCAATGTCCTCAGTGGCCTTATGCTCTTCACGTTGCTGATCGGAAACATACAGGTATTTCTGCACGCGGTCCTGGCAAGGAAGCGGAAGATGCAGCTGCGGTTCCGGGACATGGAATGGTGGATGAGGCGGAGGCAACTACCGTCCCGGCTGAGGCAAAGGGTGCGCAAATACGAGCGCGAACGCTGGGCCGCCGTCACGGGAGACGAGGAGATGGAGATGATCAAGGATCTGCCTGAAGGGCTGAGGCGGGACATCAAGCGCTACCTGTGCCTTGAGCTAGTTAAGCAGGTTCCACTGTTCCATGGCATGGACGACCTGATCCTGGACAACATCTGCGACCGGCTGCGGCCTCTGGTGTTGTCCAGCGGGGAGAAGGTGATCCGGGAGGGCGACCCCGTGCAGCGCATGGTGTTCATCCTGCAGGGCAAGCTCCGGAGCACGCAGCCGCTGACCAAAGGCGTGGTGGCGACGTGCATGCTGGGCGCGGGCAGCTTCCTGGGCGACGAGCTGCTGTCGTGGTGCCTGCGCCGCCCCTTCGTGGACCGGCTCCCCGCGTCGTCGGCCACGTTCGAGTGCGTGGAAGCGGCGCAGGCGTTCTGCCTCGGCGCGCCGGACCTGCGGTTCATCACCGAGCACTTCCGGTACAACTTCGCCAACGAGAAGCTCAAGCGCACGGCGCGGTACTACTCGTCCAACTGGCGGACGTGGGCCGCCGTCAACATCCAGCTCGCGTGGCGCAGGTACAGGGCCCGGACGTCGGCGGACCTGGCGGCGCCGCCGTTGGTGGGCGGGCCCGACGACGGGGACCGGCGGCTCAGACACTACGCGGCCATGTTCATGTCGCTACGGCCGCATGACCACCTAGAGTGA

>ZMCNGC5

ATGTCGTACGACCAGTCGGCTTTCCAGGTCGACTACATGGGCGTCGGCGCCGGCGCCGGCGTCAGCGCGTCCCGGCGGCGGTTCATGCCTTCGGAGTCGCTGGCCCGCGGCGTCATCACGCACGGCTCGGCGCAGCTGCGCACCATCGGGCGGTCGCTCCGGGCCGGCGCCACCATGGCGGCCGTGTTCCAGGAGGACCTCAAGAACACCTCCCGGCGCATCTTCGACCCGCAGGACCCGGTGCTGGTGCGCCTCAACCGCGCCTTCTTCATCTCCTGCATCGTGGCCATCGCCGTGGACCCCATGTTCTTCTACCTGCCCATGGTCACCGACGAGGGCAACCTGTGCGTGGGCATCGACCGCTGGCTCGCCATCTCCACCACCGTCGTGCGCTGCGTGGTGGACCTCTTCTTCCTGGGCCGCATCGCGCTGCAGTTCCGCACCGCCTACATCAAGCCGTCCTCCAGGGTGTTCGGGCGCGGCGAGCTCGTGATCGACACCGCGCTCATCGCCCGCCGCTACATGCGCCGCTTCTTCTCCGCGGACCTCATGTCCGTGCTCCCGCTGCCCCAGGTGGTCATCTGGAAGTTCCTGCACCGGTCCAAGGGCACCGCCGTGCTGGACACCAAGAACAGCCTGCTCTTCATCGTCTTCATCCAGTACGTCCCGCGCGTGGTGCGCATCTACCCCATCTCCTCGGAGCTCAAGCGCACCAGCGGCGTCTTCGCCGAGACCGCCTACGCCGGCGCCGCCTACTACCTCCTCTGGTACATGCTGGCCAGCCACATCGTCGGCGCCTTCTGGTACCTGCTGTCCATCGAGCGGGTCAGCGACTGCTGGAGGAACGCGTGCGACGAGTTCCCCGGGTGCAACCAGATCTACATGTACTGCGGCAACGACCGGCAGCTGGGGTTCCTGGAGTGGCGCACCATCACCCGGCAGGTGATCAACGAGACGTGCGAGCCCAAGCGGGACGGCAGCATCCCCTTCAACTACGGCATATACTCGCCGGCCGTCGTGTCGGACGTGCTCAAGTCCAAGGACACCACCTCCAAGCTGCTCTTCTGCCTCTGGTGGGGGCTGGCCAACCTGAGCACCCTCGGGCAGGGGCTCAAGACCAGCATCTACACCGGGGAGGCGCTCTTCTCCATCGCGCTCGCCATCTTCGGCCTCATCCTCATGGCCATGCTCATCGGCAACATCCAGACCTATCTCCAGTCCCTCACCGTGCGCCTGGAGGAGATGCGCGTGAAGCAGCGCGACTCGGAGCAGTGGATGCACCACCGGCTGCTGCCGCCGGAGCTGCGCGAGCGCGTCCGCCGCTACGACCAGTACAAGTGGCTCAACACCCACGGCGTCGACGAGGAGGCGCTGGTGCAAAACCTGCCCAAGGACCTCCGCCGCGACATCAAGCGCCACCTCTGCCTCGGCCTCGTCCGCCGGGTGCCGCTCTTCGCCAACATGGACGAGCGCCTCCTGGACGCCATCTGCGAGCGCCTCAAGCCCAGCCTGTGCACGGAGCACACCTACATCACCCGGGAGGGCGACCCCGTCGACCAGATGGTCTTCATCATCCGCGGCAGCCTCGAGAGCATCACCACCGACGGCGGCCGCACGGGGTTCTACAACCGCAGCCTGCTCGAGGAGGGCGACTTCTGCGGGGAGGAGCTGCTCACGTGGGCGCTCGACCCCAAGGCCGGCGCCTGCCTGCCGTCGTCCACGCGCACCGTCATGGCGCTCTCGGAGGTGGAGGCCTTCGCGCTGCACGCCGAGGAGCTCAAGTTCGTGGCGGGGCAGTTCCGCCGGATGCACAGCAAGGCGGTGCAGCACACGTTCCGGTTCTACTCCCAGCAGTGGCGCACGTGGGCAGCCACCTACATCCAGGCGGCGTGGCGGCGGCACCTCAAGCGCAGAGCGGCCGAGCTGCGGCGCAGGGAGGACGAGGAGCTGGAGGAGGACGAAGGCAAGTCCAACAGAATCAGGACCACCATACTGGTGTCGCGGTTCGCAGCCAACGCTATGCGCGGCGTGCACCGGCAGCGCTCCAGGCGGGCAGTGGCCGTGTCCGAGCTGCTGATGCCCATGCCCAAGCCGCGTGA

>ZMCNGC12

ATGCATGCCGCGTGCGTGACCATTTCTGTGCGTTCGCAGGTGGTCGTCTGGGTGGCGACGCCGGCGATGATACGCGCCGGGTCGACGACCGACGTCATGATCGTGCTGCTGACGGCGTTCCTGCTGGAGTACCTGCCCAAGATCTACCACGCCGTCCGCGTCCTGCGCCGGATGCAGGGCGTCTCCGGCTACCTCTTCGGCACCATCTGGTGGGGGATCGCGCTCAACCTCATGGCCTACTTCGTCGCCGCTCACGCGGTGGGCGCGTGCTGGTACCTGCTCGGCGCGCAGCGGGCCACCAAGTGCCTCAGGGAGCAGTGCGCCCAGGCCGGGAGCGGGTGCGCGCCCTGGGCGCTGGCGTGCGCGGAGCCGCTCTACTACGGCGCCACCGCCAGCAGCGTGGGGGCGGCCAGGCTCGCCTGGGCCGGCAACGCCACGGCCAGGGGCACGTGCCTCGACAGCGCCGACAACTACCAGTACGGGGCCTACCAGTGGACTGTCATGCTGGTGGCCAACCCCAGCAGGGTCGAGAGGGTTCTGCTCCCCATCTTCTGGGGGCTAATGACTCTCAGCACCTTTGGGAATCTGGAGAGCACGACGGAGTGGCTGGAGATCGTGTTCAACATCGTCACCATCACCGGCGGGCTGATTCTCGTGACGATGCTCATAGGGAACATCAAGGTGTTCCTGAACGCGACGACGTCGAAGAAGCAGGCGATGCACACGCGGCTGCGCAGCGTGGAGCTGTGGATGAAGCGCAAGGACCTGCCCAGGAGCTACCGGCACCGGGTGCGCCAGTACGAGCGGCAGCGGTGGGCGGCCACGCGCGGCGTCGACGAGTGCCGCATCGTCCGTGACCTTCCGGAGGGGCTCCGCCGGGACATCAAGTACCACCTCTGCCTCGGCCTCGTGCGCCAGGTGCCGCTGTTCCAACACATGGACGACCTGGTGCTCGAGAACATCTGCGACAGGGTCAAGTCCCTCATTTTCCCCAAAGGAGAAGTTATTGTCAGAGAAGGGGACCCAGTGAAGAGGATGCTGTTCATCGTGCGTGGCCACCTGCAGAGCAGCCAGGTGCTCCGCAACGGCGCCGAGAGCTGCTGCATGCTGGGGCCGGGCAACTTCAGCGGCGACGAGCTCCTGTCGTGGTGCCTGCGCCGGCCGTTCCTGGAGCGGCTGCCGGGGTCGTCTTCCACGCTGGCCACGCTGGAGAGCACGGAGGCCTTCGGGCTGGACGCCGCGGACGTCAAGTACGTCACGCAGCACTTCCGGTACACCTTCACCAACGACAAGGTGCGGCGCAGCGCGCGCTACTACTCGCCCGGGTGGCGCACGTGGGCGGCCGTGGCGGTGCAGCTCGCGTGGCGCCGCTACAAGCACCGCAAGACGCTCGCGTCGCTGTCGTTCATCCGCCCGCGCCGCCCGCTCTCGCGGTGCTCGTCGCTCGGCGAGGAGAAGCTACGCCTCTATACCGCGCTGCTCACGTCGCCCAAGCCCAACCAGGACGACCTGCTGTGA

>ZMCNGC2

TATGTGTGTTTTTTACTCTTGACAATGTTTCACCCCTATTTGCAGACCTATTTGCAGTCAGCCTCTTTGAGAGTAGAAGAAATGAGAGTGAAAAGCCGTGACACTGATCAGTGGATGTCATATCGACATCTTCCTGAGAACCTCAAGGAAAGAATACGGCGTTATGAACAATATAGATGGCAAGAAACAAGCGGGGTTGATGAAGAGCAACTCCTTATGAACCTCCCCAAAGATCTTAGGAGGGATATAAAACGACATCTTTGTTTGAAACTTCTCATGAGGGTTCCACTGTTTGAAAATATGGACGAACAGCTTTTGGATGCCATGTGTGACTGCCTAAAGCCCATTCTGTACACAGAAGGTAGCTGCGTTATTCGCGAAGGAGATCCGGTGAACGAGATGCTCTTTGTCATGAGGGGAAACCTAATGAGCATGACGACGAATGGTGGAAGAACCGGCTTCTTTAACTCCGATGTTCTGAAGGCCGGAGATTTCTGCGGCGAAGAGCTCCTCACCTGGGCTCTTGACCCCACGTCAACATCGAGCCTCCCCAGCTCAACAAGGACGGTGAAGACGATGTCTGAAGTGGAAGCCTTTGCCTTGAGGGCTGAAGACTTGAGGTTTGTGGCCACCCAGTTCCGACGACTCCACAGCAAACAGCTCCAGCACACTTTCAGGTTCTACTCGCAGCAGTGGAGGACCTGGGCCGCCTGCTTCATCCAAGCTGCCTGGCACCGGTACTGCAGGAAGAAGATCGAGGATTCTTTGCGTGAGAAGGAGAAGAGGCTGCAGTTCGCGATTGCCAACGACAGCTCCACTTCGCTCAGCTTCATGGCAGCGCTGTATGCTTCGCGGTTCGCTGGGAATATGATACGGATCCTGAGGAGAAACGCCACGCGCAAGGCCAGGCTGCAGGAAAGAGTGCCCGCGAGACTGCTGCAGAAACCGGCTGAACCCAACTTTTCCGCAGAAGAGCAGTAG

>ZMCNGC8

ATGTATTATTCTTCTATTGTTACTACTTTGGTTCGAAACCACACTATTTCGACCCAAAATCATTGGTTTTTGTTGTATTTATATTTCTTGCGCTTTTGCTATTTTTCTAACTTTCTGTTTATCTTTAGGATCTTTCCCGACGAGAGACAAAATCAATCTAAGTCATTGTATCAAACTACACGGGCTGACAGATTTGGCGCAAATAGAATAGATCTGAAGAATCCTGAGAAGCTTAAGGTGTTAAATGAAAGCAACAAACCCTGGCACCAGCGTATTCTAGACCCTGGAAGTAATATTGTACTGAGATGGAACAGGGTGTACCTTGTGGCATGTTTGTTTGCTCTTTTTATAGATCCTTTTTTCTATTACCTTCCATTGATTAGACAAAATGGCAATGGATCTTCATGTGTTGCCAAGGACCAGGGACTGAGCATAAGAATCACTGTCCTACGATCACTTGCTGACTTATTTTACATGTTGAACATAGCAATCAAGTTTCATACTGCATATGTGGATCCAAAGTCCAGAGTCCTTGGAAAGGGAGAGCTTGTTGTGGATATTAAGAAGATTCAACGAAGATATATAAGAACTGATTTCTTTGTAGACATACTTGCAGCTGTGCCACTTCCACAGGTTACTGTGTGGTTAATTATGCCTGCGATAAAAAGCTCAGATTATAACATCCGGAACACTACATTTGCTCTCGTAATTGTAATTCAGTATGTCATAAGAATGTATCTCATCATCCCTTTAAGCAATCAGATTATCAAAGCTGTTGGAGTAGTTGCAAAGTCAGCTTGGGGGGGAGCAGCATACAATCTTCTTCTCTACATGCTTGCAAGCCATATTACTGGTGCAATATATTACCTTCTCTCCATCGAACGGCAGATTACATGCTGGGATCAGCAGTGCGTTGCTGAGTACAATGATACACATTGCAACTTTAGTTTTATAAGCTGTGAGAATAATGGTTCTAATGATTATTCTGTGTGGGCAAATAAGACAAAAGTATTTGCCAACTGTGATGCCACGAATAGTAGTATATCATTTAACTACGGGATGTTTTCTAGTGCACTGAGTAAAGGTGCTGTATCATCTCCATTCCTTGAGAAGTATTTCTTTTGCCTATGGTGGGGCTTGCTGCAGCTTAGTTCAAGCGGAAATCCTCTCGTGACAAGTGCATTTATCACAGAGAATGCATTTGCGATAGCAATTGGTGCTATCAGTCTCATACTCTTTGCTCAGTTGATTGGCAAAATGCAGACATACCTGCAGTCTATCAGTAAAAGGCTTGAAGAGTGGAGGCTGAGGCAAAGGGACATGGATGAGTGGATGAGACACCATCAACTCCCATCTCATCTTCAAGAACGTGTGCGGCGGTTCGTTCAAGTCAAATGGCTTGCTACAAGAGGAGTAGAAGAAGAATCCATCTTGCAAGCTTTGCCTGCTGATATTCGTCGGGATGTGCAGCGTCATCTTTGTTTGGACCTCGTTAGACGTGTACCTTTTTTCTCTGAGATGGATAACCAACTTCTCGATGCCATCTGTGAGCGGCTGGTGTCTTTCCTGTGCCCTGAGAACACGTACATCTCTCGCGAGGGTGATCCTGTGAACGAGATGCTCTTCATTATACGCGGGAAACTAGAGAGCTCAACGACAAATGGTGGCCGCAGCAACTTCTTCAACTCCATCATCCTGCGCCCCGGCGATTTCGCAGGCGAGGAGCTGCTCACGTGGGCCCTGCTCCCCAAGACCAACGTCCACTTCCCGCTCTCGACAAGGACCGTACGGAGCCACACAGAGGTGGAGGCCTTCGCTCTGCGGGCTGAGGACCTGAAGTTCGTCGCGAACCAGTTCCGTAGGCTCCACAGCAAGAAGCTCCAGCACACGTTCCGGTTCTACTCCCACCACTGGAGGACCTGGGCCGCCTGCTTCATCCAGGCCGCTTGGCGGCAGCACCAGAGGAGGAAGCTGGCCGAGAGCCTCAGCCGCTGGGAGTCGTACTCGTGGTGGTCGGCGGAGGACCACCCAACCGGCGATAAGCCGAGGCAGGAGGGCACCTCGAGCGGCGGCGGCGGCACGAGGACGATCGCTGAAGGTGCCATCGCCCATATGCACAAGCTCGCCTCTGCTTCCAGAAGGTTCCGCACCGAGGACGTCGCTATCCGCAGGCTGCAGAAGCCTGACGAGCCCGATTTCTCCGCGGACCATTTTGATTGA

>ZMCNGC9

ATACTGCTAGTAGTTCCTAAAGTTGGGTTATCTGCTGCAAACTATGCTAAGAATTTATTGCGTGTCACTGTTCTTCTTCAATATGTCCCCCGTATCATCAGATTCGTACCACTTCTTGATGGTCAGTCCACCAATGGATTCATATTTGAGTCAGCATGGGCTAATTTTGTGATCAACCTTCTAATGTTTATTTTGGCGGGACATGTGGTTGGTTCATGTTGGTATCTCTTTGGCTTACAGAGGGTTAACCAATGTCTACGAGATGCTTGTTCTATATCGACCATTCCATATTGTGATTCTTTTATAGACTGTGGACGTGGCATTGGGAGTGGACTGTACAGACAGCAGTGGTTCAATGACTCGGGTGCAGAAGCTTGTTTTAACACTGGAAATGATGCTACTTTCCAATATGGAATTTATGAGCAGGCTGTTTTGCTCACTACAGAAGACAGTGCTGTAAAACGATATATATATTCATTATTTTGGGGGTTTCAGCAAATAAGTACCTTAGCAGGAAACCTTGTCCCGAGTTACTTTATATGGGAAGTTCTGTTCACGATGGCTATTATTGGTCTGGGACTGTTGCTTTTTGCATTGCTTATTGGAAACATGCAAAATTTTCTGCAAGCTCTTGGAAGAAGGAGGTTGGAAATGCAACTCAGGCGCCGTGATGTTGAAAAGTGGATGAGCCATAGGCGATTGCCTGAAGATTTGAGAAGGAGGGTTAGACGAGCCGAAAGGTTCACCTGGGCAGCTACTCAAGGAGTGAATGAAGAGGAGCTTTTGAGTAATTTACCTGAAGACATCCAAAGGGACATACGTCGCCACTTCTTTAGATTCCTTAATAAGGTCCGATTATTCACCTTGATGGATTGGCCTATATTGGATGCAATATGTGACAAATTAAGACAAAACTTGTATATTAGTGGAAGTGACATTCTTTACCAAGGTGGCACTGTTGAAAAGATGGTCTTCATAGTGAGAGGGAAGCTGGAAAGCATCAGTGCAGATGGCAGCAAGGCTCCATTACATGATGGAGATGTATGTGGAGAGGAGCTCCTCACGTGGTACTTGGAACACTCTTCAGCGAACAGAGATGGTGGGAAAATTAAATTCCAAGGTATGCGGTTGGTTGCTATACGTACAGTAAGATGTTTAACAAATGTTGAAGCTTTTGTACTCAGAGCAAGTGATCTGGAAGAAGTCACCTCGCAGTTTGCTCGATTCCTGCGTAATCCACGAGTGCAGGGAGCGATCAGGTATGAATCCCCCTACTGGCGAACCATTGCTGCAACTCGTATTCAGGTTGCATGGAGGTATCGTAAAAGGCGGCTGAAGCGAGCTGAGAAGTCGAGGTTGAGCGAAGAGACTTATGCCTCACTTGGATCTTGA

>ZMCNGC1

ATGGCGGGCCGGGAGGAGAGATATGTGAGGTTTCATGACTGGAAATCAGAGCAATCTGTTTCTGTTATTTCAGATAGGGTAGTATCAGAAAAAGGGCATAACATCTTTGGCTTGTTAAAGGACAGAACAGCAGGAGCCTTTTCATTCCTGGGGAACTCTTCACATTCTGAAGCCCTAAACAAATTAGGCCTAGGGGAGAAGTCAAAAACAAAAGTTCTTGATCCTCAAGGGCCATTTTTGCAGAGATGGAACAAGATATTTGTGATATCATGTCTTTTTGCAGTTTTTGTGGACCCATTGTTCTTGTATGTCCCAGTAATTGATGGTGGCAACAACTGCCTGTACTTGGACAAGAAGTTAGAGACCACAGCAAGTATCCTGCGCTTTTTCACAGATATCTTCTATTTACTCCATATACTGTTTCAGTTCAGAACAGGCTTTATTGCTCCCTCTTCTAGAGTGTTCGGTCGGGGTGCCTTGGTTAAGGACACATTTGCAATAGCAAAGCGATATCTATCAACATTGTTCCTGGTGGATTTCTTAGCGGTTCTGCCCCTCCCTCAGGTGTTTGTGTTGGTGGTGCTGCCTAAGCTCCAAGGTCCTGAAATTATGAAGGCAAAAATTGTACTGTTGGTTATTATTATTTGTCAATATGTGCCTCGACTGCTCCGAATAATACCACTTTACCTTCAAATCACAAGATCTGCTGGCATACTTACAGAGACAGCATGGGCTGGTGCTGCTTTCAACCTTATAATTTATATGCTTGCCAGTCATGGCTTTGGAGCTCTTTGGTACATTCTTTCCATCCAGCGAGAAGACACCTGTTGGAGACAAGCATGTATCAATCAGACTGGCTGTGATCCTACATCTTTGTACTGCGGGTATCATTCACTTGCAAATAATTCTTTCTTACAAAATGCGTGCCCAACAAATAGCACTGCCAATCCAGACCCTATATTTGGAATCTTTCTACCAGCTCTCCAAAATGTTTCACAATCGACGAGTTTCTTTGAAAAACTATTCTATTGCTTTTGGTGGGGGCTACAAAATCTAAGTTCCCTTGGCCAGAACATGAAAACAAGCACTAATACATTGGAGAATCTGTTTGCTGTTTTTGTCTCGACATCGGGTTTGGTTCTATTTGCACTACTTATTGGTAATGTGCAGACCTATTTACAGTCAGCTTCTGTGCGTATCGAAGAAATGAGAGTGAAAAGGCGTGATACAGAGCAGTGGATGGCACATAGGCTACTCCCTGAGAATCTAAAGGATCGGATTATGCGCCACGAACAATATAGGTGGCAAGAAACAAGAGGGGTTGACGAAGAGGGCCTTCTTAAAAATCTTCCAAAGGATCTTAGAAGAGAGATAAAGAGACATCTTTGTTTGTCACTTCTCATGAAGGTTCCAATGTTTGAAAACATGGATGAACAGCTGTTGGATGCCATGTGTGATCGTCTAAAGCCTATGCTGTACACAGAAGGAAGCTGCATCATTCGCGAAGGTGATCCAGTGAATGAAATGCTCTTCATCATGAGAGGAACACTAGAGAGTACCACAACAAATGGTGGGCAAACTGGTTTCTTCAACTCTAATGTTCTAAAAGGTGGAGACTTCTGTGGTGAAGAGCTCCTCACGTGGGCCCTTGACCCCACTTCAGCTTCAAATCTTCCTGGCTCAACTAGGACAGTGAAGACGTTGTCTGAAGTCGAAGCTTTTGCTCTGAGGGCTGACGACTTGAAGTTTGTTGCCACACAATTTAGGAGGCTCCACAGCAAACAACTTCAGCATACCTTCCGGTTTTACTCACAGCAATGGAGGACCTGGGCTGCTTGCTTCATACAGGCAGCTTGGCACAGATACTGTAGGAAGAAGCTGGAAGAGGCTTTATATGAGAAGGAGAAGAGGTTACAAGCAGCAATTGTAAGTGATGGCACTACTTCGCTCAGTCTCGGTGCAGCGCTCTATGCTTCACGTTTTGCTGGCAACATGATGCGGATCTTACGGAGAAATGCCACCAGAAAGGCCCGTTTGCAGGAAAGAGTACCTGCAAGACTGTTGCAAAAGCCAGCAGAACCCAACTTCTTCGCTGAAGATAGCTGA

>ZMCNGC10

ATGCCTCCGCTCGCATTCCTCCGCCGCTACCTCCCCGCGAGGAACCGAGTGTCTGTTATCGATCCTGAGTTGCGTCTGTCTGTTTGTTTCCCTGAGCAAAGGCTTCTCGCGCGAGCGTGCGATGGTGGAGTCCGGGGGAGCCCGGGCGTGGCGCGGGACGAGGAGGCCGGAGGCAGCGGCGGACTGAGCGGCCGGTCGGCGGGGGCGCCGTCCGGGGAGTGCTACGCGTGCACGCAGCCCGGGGTGCCGGCGTTCCACTCCACGGCCTGCGACCAGGTGCACTCGCCGGACTGGGACGCCGACGCGGGGTCCTCGCTGGTGCCGGTCCAGGCGCAGCAGCAGGCCCAGCCGGCGGCGGCGGCGGCGCAGCACGCGGCGCGGTGGCTGTTCGGGCCCGTGCTGGACCCGCGCAGCAAGCGCGTGCAGCGCTGGAACCGCTGGATCCTGCTCGGCCGCGCCGCCGCGCTGGCGCTGGACCCGCTCTTCTTCTACGCGCTCTCCATCGGCCGCGCCGGCCGGCCCTGCCTCTACTTGGACGCCGGCCTCGCCGCCGCGGTCACCGCGCTCCGGACCTGCGCCGACGTCGCGCACCTCGCGCACGTGCTCCTGCAGTTCCGCCTCGCCTACGTCTCCCGCGAGTCCCTCGTCGTCGGGTGCGGCAAGCTCGTCTGGGACGCCCGCGCCATCGCCGCGCACTACGCCCGCTCCGTCAAGGGCCTCTGCTTCGACCTCTTCGTCATCCTCCCCATCCCGCAGGTCATCTTCTGGTTGGTTATACCAAAGTTAATTAGGGAAGAACGTGTTAGGCTTATCATGACGATACTGCTACTCATGTTCATATTTCAATTTCTCCCCAAGGTCTACCATAGTATACACATCATGAGGAAAATGCAGAAGGTGACGGGTTACATCTTTGGATCGATATGGTGGGGATTTGGTTTAAATCTATTTGCCTATTTCATTGCTTCTCATATTGCAGGTGGGTGCTGGTATGTTCTTGCAATCCAGCGCATTGCTTCCTGCCTCCAGGAAGAATGCAAGAAAAACAATAGTTGTGATCTAATATCACTAGCTTGTTCGAAGGAGATATGCTTTCACCCTCCTTGGTCTTCGAATGTTAATGGGTTCGCATGTGATACGAACATGACCTCCTTTAGTCAACGAAATGTGTCTACTTGTTTAAGTGGTAAAGGGTCGTTTGCTTATGGAATCTATTTGGGGGCTCTTCCTGTTATATCGAGCAATTCGCTTGCTGTCAAAATTCTCTATCCTATATTTTGGGGACTCATGACACTCAGTACTTTTGGTAACGATCTTGCCCCAACAAGCAATGGTATTGAGGTGATATTCAGCATAATCAATGTCCTCAGTGGCCTGATGCTCTTCACATTGCTGATCGGAAACATACAGGTATTTCTGCACGCGGTCCTGGCAAGGAAGCGGAAGATGCAGCTGCGGTTCCGAGACATGGAATGGTGGATGAGACGGAGGCAGCTGCCGTCTCGGCTGAGGCAGAGGGTGCGCAAATATGAGCGCGAACGCTGGGCCGCCGTCACGGGAGACGAGGAGATGGAGATGATCAAGGATCTGCCTGAAGGACTGAGGCGGGACATCAAGCGCTACCTCTGCCTCGAGCTGGTTAAGCAGGTTCCGCTGTTCCATGGCATGGACGATCTGATCCTGGATAACATCTGCGACCGGCTGCGGCCACTGGTGTTCTCCAGCGGGGAGAAGGTGATCCGAGAGGGCGACCCCGTGCAGCGCATGGTGTTCATCCTGCAGGGCAAGCTCCGGAGCACGCAGCCGCTGACCAAGGGCGTGGTGGCAACGTGCATGCTAGGGGCGGGCAACTTCCTAGGCGACGAGCTGCTGTCGTGGTGCCTGCGCCGCCCCTTCGTGGACCGGCTCCCCGCGTCGTCGGCCACGTTCGAGTGCGTGGAGGCGGCGCAGGCGTTCTGCCTCGACGCGCCGGACCTGCGGTTCATCACCGAGCACTTCCGCTACAAGTTCGCCAACGAGAAGCTCAGGCGCACGGCGCGGTACTACTCGTCCAACTGGCGGACGTGGGCCGCCGTCAACATCCAGCTCGCGTGGCGCAGGTATAGGGCCCGGGCATCGACGGACCTGGCGGCGATGGCCGCGCCGCCGTTGGCGGGCGGACCCGACGACGGGGACCGGCGGCTCAGACACTACGCGGCCATGTTCATGTCGCTCCGGCCGCATGACCACCTAGAGTGA

**5. Protein sequence of maize**

>ZMCNGC3

VSKFNCFLFLGWIELLLQTYLQSASGHIEEMRVRRRDMEQWMSYRLLPEHIKERILRHHQ

YRWQETQGVDEEGLLVNLPKDLRRDIKRHLCLSLLKRVPMFENMDDQLLDAMCDRVKPMLYTEGSHIVREGDPVNEMFFIMRGRLESTTTDGGRAGFFNSNVLEGGDFCGEELLTWALDPASGSNLPSSTRTARTLSEVEGFSLRARHLRFVASQYRRLHSKQLRHTFRFYSHQWRTWAACFVQAAWHRYCRRRLEEGVREKERMFRAAAVTDISSSRSLGAALYAAHFARNMVRTLRRNAARKARLLDTVSSRLLQKPAEPNFFAEED

>ZMCNGC7

MASGASRNVRFQNEIEVQSFRTSPLQSLSRKHGKAHDPRKCRLGFRGGCLEKACRNRKPMLKDRVLSRAFSEELESLMHAAGGSHLFFDPRGQLIHLWNKIFLSACLLSLFVDPLFLYLTGTQRNTCVEFKDSLALTLSMVRSLLDLFYAAHILFRFRTAFIAPSSRVFGRGELVIQPYEIARRYLGRTFWFDLVTALPLPQFVIWIVIPRLNEYSRTANTKNILRFSIIFQYLPRLFQIFPLSGRIVMATGVMTETAWAGAAYNLILYMLASHVLGALWYLFSVQRQEACWREACLLVSPTSQTMFFDCKALSSNRTIWYELSNITTSRCTPGNGFYPFGIYEEALYAKLTSSSFTQKYFYCFWWGLKNLSSLGQNLSTSLFIGEITFAIVVGVLGLVLFGLLIGNMQSYLQATMVRLEEWRTKRTDMERWMHHRQIPQPLKQCVRRYHQYQWVATRGVDEEALLQDLPMDIRRDIKRHLCLDLVRRVPLFDEMDERMLDAICERLRPALYTRGTRLMRELDPVDSMLFIIRGYLDSYTTQGGRSGFFNSCRIGAGEFCGEELLTWALDPRPAAKLPLSTRTVRAVSEVEAFALVADDLRFVASQFRRLHSARIRHRFRFYSHQWRTWAACFIQAAWRRYKRRRASMELRVREVRAGGSLLRSRRHSIEGKASIRKPMEPDFTVEEED

>ZMCNGC11

MPRLAFLRRSLPARLLARACGGGGGGDQGSPDQVARDEEAGGSGGMSGRSSAGGPSGGECYACTQPGVPAFHSTTCDQVHSPDWDADAGSSLVPVQGQAQAAAAAAAPRQRHAARWLLGPVLDPRSRRVQRWNRWILLGRAAALAVDPLFFYALSIGRAGQPCLYMDAGLASAVTALRTCADVAHLAHVLLQLRLAYVSRESLVVGCGKLVWDARAVAAHYARSVKGLCFDLFVILPIPQVIFWLVIPKLIREEQVKLIMTILLLMFIFQFLPKVYHSIHIMRKMQKVTGYIFGSIWWGFGLNLFAYFIASHIAGGCWYVLAIQRIASCLQEECKRNNSCDLISLACSKEICFHPPWSSNVNGFACDTNMTSFSQQNVSTCLSGKGSFAYGIYLGALPVISSNSLAVKILYPIFWGLMTLSTFGNDLAPTSNGIEVIFSIINVLSGLMLFTLLIGNIQVFLHAVLARKRKMQLRFRDMEWWMRRRQLPSRLRQRVRKYERERWAAVTGDEEMEMIKDLPEGLRRDIKRYLCLELVKQVPLFHGMDDLILDNICDRLRPLVLSSGEKVIREGDPVQRMVFILQGKLRSTQPLTKGVVATCMLGAGSFLGDELLSWCLRRPFVDRLPASSATFECVEAAQAFCLGAPDLRFITEHFRYNFANEKLKRTARYYSSNWRTWAAVNIQLAWRRYRARTSADLAAPPLVGGPDDGDRRLRHYAAMFMSLRPHDHLE

>ZMCNGC5

MSYDQSAFQVDYMGVGAGAGVSASRRRFMPSESLARGVITHGSAQLRTIGRSLRAGATMAAVFQEDLKNTSRRIFDPQDPVLVRLNRAFFISCIVAIAVDPMFFYLPMVTDEGNLCVGIDRWLAISTTVVRCVVDLFFLGRIALQFRTAYIKPSSRVFGRGELVIDTALIARRYMRRFFSADLMSVLPLPQVVIWKFLHRSKGTAVLDTKNSLLFIVFIQYVPRVVRIYPISSELKRTSGVFAETAYAGAAYYLLWYMLASHIVGAFWYLLSIERVSDCWRNACDEFPGCNQIYMYCGNDRQLGFLEWRTITRQVINETCEPKRDGSIPFNYGIYSPAVVSDVLKSKDTTSKLLFCLWWGLANLSTLGQGLKTSIYTGEALFSIALAIFGLILMAMLIGNIQTYLQSLTVRLEEMRVKQRDSEQWMHHRLLPPELRERVRRYDQYKWLNTHGVDEEALVQNLPKDLRRDIKRHLCLGLVRRVPLFANMDERLLDAICERLKPSLCTEHTYITREGDPVDQMVFIIRGSLESITTDGGRTGFYNRSLLEEGDFCGEELLTWALDPKAGACLPSSTRTVMALSEVEAFALHAEELKFVAGQFRRMHSKAVQHTFRFYSQQWRTWAATYIQAAWRRHLKRRAAELRRREDEELEEDEGKSNRIRTTILVSRFAANAMRGVHRQRSRRAVAVSELLMPMPKPR

>ZMCNGC12

MHAACVTISVRSQVVVWVATPAMIRAGSTTDVMIVLLTAFLLEYLPKIYHAVRVLRRMQGVSGYLFGTIWWGIALNLMAYFVAAHAVGACWYLLGAQRATKCLREQCAQAGSGCAPWALACAEPLYYGATASSVGAARLAWAGNATARGTCLDSADNYQYGAYQWTVMLVANPSRVERVLLPIFWGLMTLSTFGNLESTTEWLEIVFNIVTITGGLILVTMLIGNIKVFLNATTSKKQAMHTRLRSVELWMKRKDLPRSYRHRVRQYERQRWAATRGVDECRIVRDLPEGLRRDIKYHLCLGLVRQVPLFQHMDDLVLENICDRVKSLIFPKGEVIVREGDPVKRMLFIVRGHLQSSQVLRNGAESCCMLGPGNFSGDELLSWCLRRPFLERLPGSSSTLATLESTEAFGLDAADVKYVTQHFRYTFTNDKVRRSARYYSPGWRTWAAVAVQLAWRRYKHRKTLASLSFIRPRRPLSRCSSLGEEKLRLYTALLTSPKPNQDDLL

>ZMCNGC2

YVCFLLLTMFHPYLQTYLQSASLRVEEMRVKSRDTDQWMSYRHLPENLKERIRRYEQYRWQETSGVDEEQLLMNLPKDLRRDIKRHLCLKLLMRVPLFENMDEQLLDAMCDCLKPILYTEGSCVIREGDPVNEMLFVMRGNLMSMTTNGGRTGFFNSDVLKAGDFCGEELLTWALDPTSTSSLPSSTRTVKTMSEVEAFALRAEDLRFVATQFRRLHSKQLQHTFRFYSQQWRTWAACFIQAAWHRYCRKKIEDSLREKEKRLQFAIANDSSTSLSFMAALYASRFAGNMIRILRRNATRKARLQERVPARLLQKPAEPNFSAEEQ

>ZMCNGC8

MYYSSIVTTLVRNHTISTQNHWFLLYLYFLRFCYFSNFLFIFRIFPDERQNQSKSLYQTTRADRFGANRIDLKNPEKLKVLNESNKPWHQRILDPGSNIVLRWNRVYLVACLFALFIDPFFYYLPLIRQNGNGSSCVAKDQGLSIRITVLRSLADLFYMLNIAIKFHTAYVDPKSRVLGKGELVVDIKKIQRRYIRTDFFVDILAAVPLPQVTVWLIMPAIKSSDYNIRNTTFALVIVIQYVIRMYLIIPLSNQIIKAVGVVAKSAWGGAAYNLLLYMLASHITGAIYYLLSIERQITCWDQQCVAEYNDTHCNFSFISCENNGSNDYSVWANKTKVFANCDATNSSISFNYGMFSSALSKGAVSSPFLEKYFFCLWWGLLQLSSSGNPLVTSAFITENAFAIAIGAISLILFAQLIGKMQTYLQSISKRLEEWRLRQRDMDEWMRHHQLPSHLQERVRRFVQVKWLATRGVEEESILQALPADIRRDVQRHLCLDLVRRVPFFSEMDNQLLDAICERLVSFLCPENTYISREGDPVNEMLFIIRGKLESSTTNGGRSNFFNSIILRPGDFAGEELLTWALLPKTNVHFPLSTRTVRSHTEVEAFALRAEDLKFVANQFRRLHSKKLQHTFRFYSHHWRTWAACFIQAAWRQHQRRKLAESLSRWESYSWWSAEDHPTGDKPRQEGTSSGGGGTRTIAEGAIAHMHKLASASRRFRTEDVAIRRLQKPDEPDFSADHFD

>ZMCNGC9

ILLVVPKVGLSAANYAKNLLRVTVLLQYVPRIIRFVPLLDGQSTNGFIFESAWANFVINLLMFILAGHVVGSCWYLFGLQRVNQCLRDACSISTIPYCDSFIDCGRGIGSGLYRQQWFNDSGAEACFNTGNDATFQYGIYEQAVLLTTEDSAVKRYIYSLFWGFQQISTLAGNLVPSYFIWEVLFTMAIIGLGLLLFALLIGNMQNFLQALGRRRLEMQLRRRDVEKWMSHRRLPEDLRRRVRRAERFTWAATQGVNEEELLSNLPEDIQRDIRRHFFRFLNKVRLFTLMDWPILDAICDKLRQNLYISGSDILYQGGTVEKMVFIVRGKLESISADGSKAPLHDGDVCGEELLTWYLEHSSANRDGGKIKFQGMRLVAIRTVRCLTNVEAFVLRASDLEEVTSQFARFLRNPRVQGAIRYESPYWRTIAATRIQVAWRYRKRRLKRAEKSRLSEETYASLGS

>ZMCNGC1

MAGREERYVRFHDWKSEQSVSVISDRVVSEKGHNIFGLLKDRTAGAFSFLGNSSHSEALNKLGLGEKSKTKVLDPQGPFLQRWNKIFVISCLFAVFVDPLFLYVPVIDGGNNCLYLDKKLETTASILRFFTDIFYLLHILFQFRTGFIAPSSRVFGRGALVKDTFAIAKRYLSTLFLVDFLAVLPLPQVFVLVVLPKLQGPEIMKAKIVLLVIIICQYVPRLLRIIPLYLQITRSAGILTETAWAGAAFNLIIYMLASHGFGALWYILSIQREDTCWRQACINQTGCDPTSLYCGYHSLANNSFLQNACPTNSTANPDPIFGIFLPALQNVSQSTSFFEKLFYCFWWGLQNLSSLGQNMKTSTNTLENLFAVFVSTSGLVLFALLIGNVQTYLQSASVRIEEMRVKRRDTEQWMAHRLLPENLKDRIMRHEQYRWQETRGVDEEGLLKNLPKDLRREIKRHLCLSLLMKVPMFENMDEQLLDAMCDRLKPMLYTEGSCIIREGDPVNEMLFIMRGTLESTTTNGGQTGFFNSNVLKGGDFCGEELLTWALDPTSASNLPGSTRTVKTLSEVEAFALRADDLKFVATQFRRLHSKQLQHTFRFYSQQWRTWAACFIQAAWHRYCRKKLEEALYEKEKRLQAAIVSDGTTSLSLGAALYASRFAGNMMRILRRNATRKARLQERVPARLLQKPAEPNFFAEDS

>ZMCNGC10

MPPLAFLRRYLPARNRVSVIDPELRLSVCFPEQRLLARACDGGVRGSPGVARDEEAGGSGGLSGRSAGAPSGECYACTQPGVPAFHSTACDQVHSPDWDADAGSSLVPVQAQQQAQPAAAAAQHAARWLFGPVLDPRSKRVQRWNRWILLGRAAALALDPLFFYALSIGRAGRPCLYLDAGLAAAVTALRTCADVAHLAHVLLQFRLAYVSRESLVVGCGKLVWDARAIAAHYARSVKGLCFDLFVILPIPQVIFWLVIPKLIREERVRLIMTILLLMFIFQFLPKVYHSIHIMRKMQKVTGYIFGSIWWGFGLNLFAYFIASHIAGGCWYVLAIQRIASCLQEECKKNNSCDLISLACSKEICFHPPWSSNVNGFACDTNMTSFSQRNVSTCLSGKGSFAYGIYLGALPVISSNSLAVKILYPIFWGLMTLSTFGNDLAPTSNGIEVIFSIINVLSGLMLFTLLIGNIQVFLHAVLARKRKMQLRFRDMEWWMRRRQLPSRLRQRVRKYERERWAAVTGDEEMEMIKDLPEGLRRDIKRYLCLELVKQVPLFHGMDDLILDNICDRLRPLVFSSGEKVIREGDPVQRMVFILQGKLRSTQPLTKGVVATCMLGAGNFLGDELLSWCLRRPFVDRLPASSATFECVEAAQAFCLDAPDLRFITEHFRYKFANEKLRRTARYYSSNWRTWAAVNIQLAWRRYRARASTDLAAMAAPPLAGGPDDGDRRLRHYAAMFMSLRPHDHLE

**5. Protein sequence of Arabidopsis and rice**

>AT5G53130

MNFRQEKFVRFQDWKSDKTSSDVEYSGKNEIQTGIFQRTISSISDKFYRSFESSSARIKLFKRSYKSYSFKEAVSKGIGSTHKILDPQGPFLQRWNKIFVLACIIAVSLDPLFFYVPIIDDAKKCLGIDKKMEITASVLRSFTDVFYVLHIIFQFRTGFIAPSSRVFGRGVLVEDKREIAKRYLSSHFIIDILAVLPLPQMVILIIIPHMRGSSSLNTKNMLKFIVFFQYIPRFIRIYPLYKEVTRTSGILTETAWAGAAFNLFLYMLASHVFGAFWYLFSIERETVCWKQACERNNPPCISKLLYCDPETAGGNAFLNESCPIQTPNTTLFDFGIFLDALQSGVVESQDFPQKFFYCFWWGLQNLSSLGQNLKTSTYIWEICFAVFISIAGLVLFSFLIGNMQTYLQSTTTRLEEMRVKRRDAEQWMSHRLLPENLRKRIRRYEQYKWQETRGVDEENLLSNLPKDLRRDIKRHLCLALLMRVPMFEKMDEQLLDALCDRLQPVLYTEESYIVREGDPVDEMLFIMRGKLLTITTNGGRTGFLNSEYLGAGDFCGEELLTWALDPHSSSNLPISTRTVRALMEVEAFALKADDLKFVASQFRRLHSKQLRHTFRYYSQQWKTWAACFIQAAWRRYIKKKLEESLKEEENRLQDALAKEACGSSPSLGATIYASRFAANILRTIRRSGSVRKPRMPERMPPMLLQKPAEPDFNSDD

>AT3G48010

MSNLHLYTSARFRNFPTTFSLRHHHNDPNNQRRRSIFSKLRDKTLDPGGDLITRWNHIFLITCLLALFLDPLYFYLPIVQAGTACMSIDVRFGIFVTCFRNLADLSFLIHILLKFKTAFVSKSSRVFGRGELVMDRREIAIRYLKSEFVIDLAATLPLPQIMIWFVIPNAGEFRYAAHQNHTLSLIVLIQYVPRFLVMLPLNRRIIKATGVAAKTAWSGAAYNLILYLLVSHVLGSVWYVLSIQRQHECWRRECIKEMNATHSPSCSLLFLDCGSLHDPGRQAWMRITRVLSNCDARNDDDQHFQFGMFGDAFTNDVTSSPFFDKYFYCLWWGLRNLSSYGQSLAASTLSSETIFSCFICVAGLVFFSHLIGNVQNYLQSTTARLDEWRVRRRDTEEWMRHRQLPDELQERVRRFVQYKWLTTRGVDEEAILRALPLDLRRQIQRHLCLALVRRVPFFAQMDDQLLDAICERLVPSLNTKDTYVIREGDPVNEMLFIIRGQMESSTTDGGRSGFFNSITLRPGDFCGEELLTWALVPNINHNLPLSTRTVRTLSEVEAFALRAEDLKFVANQFRRLHSKKLQHAFRYYSHQWRAWGTCFIQAAWRRYMKRKLAMELARQEEEDDYFYDDDGDYQFEEDMPESNNNNGDENSSNNQNLSATILASKFAANTKRGVLGNQRGSTRIDPDHPTLKMPKMFKPEDPGFF

>AT2G23980

MFDTCGPKGVKSQVISGQRENFVRLDSMDSRYSQSSETGLNKCTLNIQGGPKRFAQGSKASSGSFKKGFRKGSEGLWSIGRSIGLGVSRAVFPEDLEVSEKKIFDPQDKFLLLCNKLFVASCILAVSVDPLFLYLPFINDKAKCVGIDRKLAIIVTTIRTVIDSFYLFHMALRFRTAYVAPSSRVFGRGELVIDPAQIAKRYLQQYFIIDLLSVLPVPQIIVWRFLYTSRGANVLATKQALRYIVLVQYIPRFLRMYPLSSELKRTAGVFAETAWAGAAYYLLLYMLASHIVGALWYLLALERNNDCWSKACHNNQNCTRNFLFCGNQNMKGYAAWDNIKVSYLQLKCPVNVPEDEEPPFDFGIYLRALSSGIVSSKNFVSKYFFCLWWGLQNLSTLGQGLETSTYPGEVIFSITLAIAGLLLFALLIGNMQTYLQSLTIRLEEMRVKRRDSEQWMHHRMLPPELRERVRRYDQYKWLETRGVDEENLVQNLPKDLRRDIKRHLCLALVRRVPLFENMDERLLDAICERLKPCLFTEKSYLVREGDPVNEMLFIIRGRLESVTTDGGRSGFYNRSLLKEGDFCGDELLTWALDPKSGSNLPSSTRTVKALTEVEAFALIADELKFVASQFRRLHSRQVQHTFRFYSQQWRTWAACFMQAAWRRYIKRKKLEQLRKEEEEEEAAAASVIAGGSPYSIRATFLASKFAANALRSVHKNRTAKSTLLLSSTKELVKFQKPPEPDFSAEDH

>AT2G24610

MEFKRDNTVRFYGDEKQTIEVGEKRVPLFKSTTAPFMKQEVLPKKSKTRLKIPRFGRFKVFPENFEIERDKILDPGGDAVLQWNRVFLFWCLVALYVDPLFFFLSSVKRIGRSSCMTTDLKLGIVITFFRTLADLFYVLHIVIKFRTAYVSRTSRVFGRGELVKDPKLIARRYLRSDFIVDLIACLPLPQIVSWFILPSIRSSHSDHTTNALVLIVLVQYIPRLYLIFPLSAEIIKATGVVTTTAWAGAAYNLLQYMLASHILGSAWYLLSIERQATCWKAECHKESVPLQCVTDFFDCGTLHRDDRNNWQNTTVVFSNCDPSNNIQFTFGIFADALTKNVVSSPFLEKYLYCLWFGLQNLSSYGQNLSTSTSVLETMFAILVAIFGLVLFALLIGNMQTYLQSITVRLEEWRLKRRDTEEWMGHRLLPQNLRERVRRFVQYKWLATRGVDEETILHSLPADLRRDIQRHLCLDLVRRVPLFAQMDDQLLDAICERLASSLSTQGNYIVREGDPVTEMLFIIRGKLESSTTNGGRTGFFNSITLRPGDFCGEELLAWALLPKSTVNLPSSTRTVRALEEVEAFALQAGDLKFVANQFRRLHSKKLQHTFRYYSHQWRTWAACFVQVAWRRYKRKKLAKSLSLAESFSSYDEEEAVAVAATEEMSHEGEAQSGAKARHHTSNVKPHFAATILASRFAKNTRRTAHKLKDVEIPMLPKPDEPDFSVDD

>AT5G54250

MATEQEFTRASRFSRDSSSVGYYSEEDNTEEEDEEEEEMEEIEEEEEEEEEEDPRIGLTCGGRRNGSSNNNKWMMLGRILDPRSKWVREWNKVFLLVCATGLFVDPLFLYTLSVSDTCMCLLVDGWLALTVTALRSMTDLLHLWNIWIQFKIARRWPYPGGDSDGDTNKGGGTRGSTRVAPPYVKKNGFFFDLFVILPLPQVVLWVVIPSLLKRGSVTLVVSVLLVTFLFQYLPKIYHSIRHLRRNATLSGYIFGTVWWGIALNMIAYFVAAHAAGACWYLLGVQRSAKCLKEQCENTIGCDLRMLSCKEPVYYGTTVMVLDRARLAWAQNHQARSVCLDINTNYTYGAYQWTIQLVSSESRLEKILFPIFWGLMTLSTFGNLESTTEWSEVVFNIIVLTSGLLLVTMLIGNIKVFLHATTSKKQAMHLKMRNIEWWMKKRHLPIGFRQRVRNYERQRWAAMRGVDECEMVQNLPEGLRRDIKYHLCLDLVRQVPLFQHMDDLVLENICDRVKSLIFTKGETIQKEGDAVQRMLFVVRGHLQSSQLLRDGVKSCCMLGPGNFSGDELLSWCLRRPFVERLPPSSSTLVTLETTEAFGLDAEDVKYVTQHFRYTFVNEKVKRSARYYSPGWRTWAAVAVQLAWRRYKHRLTLTSLSFIRPRRPLSRCASLGEDKLRLYAAILTSPKPNPDDFDDY

>AT2G28260

MGYGNSRSVRFQEDQEVVHGGESGVKLKFKINGTQINNVKMMSKGKFLKAKVLSRVFSEDLERVKTKILDPRGQTIRRWNKIFLIACLVSLFVDPLFFFLPVMRNEACITIGVRLEVVLTLIRSLADAFYIAQILIRFRTAYIAPPSRVFGRGELVIDSRKIAWRYLHKSFWIHLVAALPLPQVLIWIIIPNLRGSPMTNTKNVLRFIIIFQYVPRMFLIFPLSRQIIKATGVVTETAWAGAAYNLMLYMLASHVLGACWYLLAVERQEACWRHACNIEKQICQYRFFECRRLEDPQRNSWFEWSNITTICKPASKFYEFGIFGDAVTSTVTSSKFINKYFYCLWWGLKNLSSLGQNLATSTYAGEILFAIIIATLGLVLFALLIGNMQTYLQSTTMRLEEWRIRRTDTEQWMHHRQLPPELRQAVRKYDQYKWLATRGVDEEALLISLPLDLRRDIKRHLCFDLVRRVPLFDQMDERMLDAICERLKPALCTEGTFLVREGDPVNEMLFIIRGHLDSYTTNGGRTGFFNSCLIGPGDFCGEELLTWALDPRPVVILPSSTRTVKAICEVEAFALKAEDLQFVASQFRRLHTKQLRHKFRFYSHQWRTWAACFIQAAWRRHRKRKYKTELRAKEEFHYRFEAATARLAVNGGKYTRSGSDSGMMSSIQKPVEPDFSSE

>AT5G15410

MPSHPNFIFRWIGLFSDKFRRQTTGIDENSNLQINGGDSSSSGSDETPVLSSVECYACTQVGVPAFHSTSCDQAHAPEWRASAGSSLVPIQEGSVPNPARTRFRRLKGPFGEVLDPRSKRVQRWNRALLLARGMALAVDPLFFYALSIGRTTGPACLYMDGAFAAVVTVLRTCLDAVHLWHVWLQFRLAYVSRESLVVGCGKLVWDPRAIASHYARSLTGFWFDVIVILPVPQAVFWLVVPKLIREEKVKLIMTILLLIFLFQFLPKIYHCICLMRRMQKVTGYIFGTIWWGFALNLIAYFIASHVAGGCWYVLAIQRVASCIRQQCMRTGNCNLSLACKEEVCYQFVSPTSTVGYPCLSGNLTSVVNKPMCLDSNGPFRYGIYRWALPVISSNSLAVKILYPIFWGLMTLSTFANDLEPTSNWLEVIFSIVMVLSGLLLFTLLIGNIQVFLHAVMAKKRKMQIRCRDMEWWMKRRQLPSRLRQRVRRFERQRWNALGGEDELELIHDLPPGLRRDIKRYLCFDLINKVPLFRGMDDLILDNICDRAKPRVFSKDEKIIREGDPVQRMIFIMRGRVKRIQSLSKGVLATSTLEPGGYLGDELLSWCLRRPFLDRLPPSSATFVCLENIEAFSLGSEDLRYITDHFRYKFANERLKRTARYYSSNWRTWAAVNIQMAWRRRRKRTRGENIGGSMSPVSENSIEGNSERRLLQYAAMFMSIRPHDHLE

>AT1G15990

MYKSQYISGQREKFVRLDDIDSSSSPATGMMMQRNCFGFNLKNRGGEKKKASKSFREGVKKIRSEGLITIGKSVTRAVFPEDLRITEKKIFDPQDKTLLVWNRLFVISCILAVSVDPLFFYLPIVDNSGSSCIGIDTKLAVTTTTLRTIVDVFYLTRMALQFRTAYIAPSSRVFGRGELVIDPAKIAERYLTRYFVVDFLAVLPLPQIAVWKFLHGSKGSDVLPTKTALLNIVIVQYIPRFVRFIPLTSELKKTAGAFAEGAWAGAAYYLLWYMLASHITGAFWYMLSVERNDTCWRFACKVQPDPRLCVQILYCGTKFVSSGETEWIKTVPELLKSNCSAKADDSKFNYGIYGQAISSGIVSSTTFFSKFCYCLWWGLQNLSTLGQGLQTSTFPGEVLFSIAIAIAGLLLFALLIGNMQTYLQSLTVRLEEMRIKRRDSEQWMHHRSLPQNLRERVRRYDQYKWLETRGVDEENIVQSLPKDLRRDIKRHLCLNLVRRVPLFANMDERLLDAICERLKPSLFTESTYIVREGDPVNEMMFIIRGRLESVTTDGGRSGFFNRGLLKEGDFCGEELLTWALDPKAGSNLPSSTRTVKALTEVEAFALEAEELKFVASQFRRLHSRQVQQTFRFYSQQWRTWASCFIQAAWRRYSRRKNAELRRIEEKEEELGYEDEYDDESDKRPMVITRSESSSRLRSTIFASRFAANALKGHRLRSSESSKTLINLQKPPEPDFDAE

>AT4G30560

MLDCGKKAVKSQVISGRLEKFVRLDSMDSRYSQTSDTGLNRCTLNLQGPTRGGGAQGNNVSSGSFKKGFRKGSKGLWSIGRSIGLGVSRAVFPEDLKVSEKKIFDPQDKFLLLCNKLFVTSCILAVSVDPLFLYLPFVKDNEKCIGIDRKLAIIATTLRTVIDAFYLFHMALRFRTAFVAPSSRVFGRGELVIDPAQIAKRYLQQYFIIDFLSVLPLPQIVVWRFLYISKGASVLATKRALRSIILVQYIPRFIRLYPLSSELKRTAGVFAETAWAGAAYYLLLYMLASHIVGAIWYLLALERYNGCWTKVCSNSSLDCHRNFLFCGNEKMDGYAAWTTIKDSVLQLNCPVNTTDNPPFDFGIYLRALSSGIVSSKSFVSKYFFCLWWGLQNLSTLGQGLETSTYPGEVIFSIALAIAGLLLFALLIGNMQTYLQSLTIRLEEMRVKRRDSEQWMHHRMLPPELRERVRRYDQYKWLETRGVDEENLVQNLPKDLRRDIKRHLCLALVRRVPLFENMDERLLDAICERLKPCLYTESSYLVREGDPVNEMLFIIRGRLESVTTDGGRSGFFNRSLLKEGDFCGEELLTWALDPKSGSNLPSSTRTAKALTEVEAFALIADELKFVASQFRRLHSRQVQHTFRFYSQQWRTWAAIFIQAAWRRYVKKKKLEQLRKEEEEGEGSVTSIRATFLASKFAANALRKVHKNRIEAKSTIELVKYQKPSEPDFSADDTS

>AT5G57940

MAGKRENFVRVDDLDSRLPSSSVAFQQNYASNFSGQLHPIHASNETSRSFKKGIQKGSKGLKSIGRSLGFGVYRAVFPEDLKVSEKKIFDPQDKFLLYCNKLFVASCILSVFVDPFFFYLPVINAESKCLGIDRKLAITASTLRTFIDVFYLAHMALQLRTAYIAPSSRVFGRGELVIDPAQIAKRYLQRWFIIDFLSVLPLPQIVVWRFLQSSNGSDVLATKQALLFIVLVQYIPRFLRVLPLTSELKRTAGVFAETAWAGAAYYLLLYMLASHIVGAFWYLLALERNDACWQEACIDAGNCSTDFLYCGNQNMDGYAVWNRAKESVLKSKCRADLDDNNPPFDFGIYTQALSSGIVSSQNFIVKYCYCLWWGLQNLSTLGQGLETSTYPMEIIFSISLAISGLILFALLIGNMQTYLQSLTIRLEEMRVKRRDSEQWMHHRMLPQDLRERVRRYDQYKWLETRGVDEEYLVQNLPKDLRRDIKRHLCLALVRRVPLFKSMDDKLLDAICMRLKPCLFTESTYLVREGDPVDEMLFIIRGRLESVTTDGGRSGFFNRSLLKEGEFCGEELLTWALDPKSGVNLPSSTRTVKALTEVEAFALTSEELKFVASQFRRLHSRQVQHTFRFYSHQWRTWAACFIQAAWRRYCKRKKMEEAEAEAAAVSSSTAGPSYSIGAAFLATKFAANALRTIHRNRNTKIRDLVKLQKPPEPDFTAD

>AT3G17700

MASHNENDDIPMLPISDPSSRTRARAFTSRSRSVSLSNPTSSIEGFDTSTVVLGYTGPLRTQRRPPLVQMSGPLTSTRKHEPLFLPHPSSDSVGVSSQPERYPSFAALEHKNSSEDEFVLKHANLLRSGQLGMCNDPYCTTCPSYYNRKAAQIPTSRVSALFDSTFHNALYDDAKGWARRFASSVNRYLPGIMNPHAKEVQTWTKFFALSCLLAIFIDPLFFFLIKVQEQNKCIMIDWPMTKAFVAVRSVTDVIFTMNILLQFRLAYVARESTVVGAGQLVSHPKKIALHYLKGKFFLDLFIVMPLPQILILWIIPAHLGASGANYAKNLLRAAVLFQYIPKLYRLLPFLAGQTPTGFIFESAWANFVINLLTFMLAGHVVGSCWYLFGLQRVNQCLRNACGNFGRECQDLIDCGNGNSSVLVRATWKDNASANACFQEDGFPYGIYLKAVNLTNHSNLFTRYSYSLFWGFQQISTLAGNQVPSYFLGEVFFTMGIIGLGLLLFALLIGNMQNFLQALGKRNLEMTLRRRDVEQWMSHRRLPDGIRRRVREAERFNWAATRGVNEELLFENMPDDLQRDIRRHLFKFLKKVRIFSLMDEPILDAIRERLKQRTYIGSSTVLHRGGLVEKMVFIVRGEMESIGEDGSVLPLYEGDVCGEELLTWCLERSSVNPDGTRIRMPSKGLLSSRNVRCVTNVEAFSLSVADLEDVTSLFSRFLRSHRVQGAIRYDSPYWRLRAARQIQVAWRYRRRRLHRLCTPQSSYSL

>AT1G01340

MILFRFKDEGKPLSSEYGYGRKARPSLDRVFKNVKWGFKKPLSFPSHKDPDHKETSSVTRKNIINPQDSFLQNWNKIFLFACVVALAIDPLFFYIPIVDSARHCLTLDSKLEIAASLLRTLIDAFYIIHIVFQFRTAYIAPSSRVFGRGELVDDAKAIALKYLSSYFIIDLLSILPLPQIVVLAVIPSVNQPVSLLTKDYLKFSIIAQYVPRILRMYPLYTEVTRTSGIVTETAWAGAAWNLSLYMLASHVFGALWYLISVEREDRCWQEACEKTKGCNMKFLYCENDRNVSNNFLTTSCPFLDPGDITNSTIFNFGIFTDALKSGVVESHDFWKKFFYCFWWGLRNLSALGQNLQTSKFVGEIIFAISICISGLVLFALLIGNMQKYLESTTVREEEMRVRKRDAEQWMSHRMLPEDLRKRIRRYEQYRWQETRGVEEETLLRNLPKDLRRDIKRHLCLDLLKKVPLFEIMDEQLLDAVCDRLRPVLYTENSYVIREGDPVGEMLFVMRGRLVSATTNGGRSGFFNAVNLKASDFCGEDLLPWALDPQSSSHFPISTRTVQALTEVEAFALTAEDLKSVASQFRRLHSKQLQHTFRFYSVQWRTWSVSFIQAAWRRYCRRKLAKSLRDEEDRLREALASQDKEHNAATVSSSLSLGGALYASRFASNALHNLRHNISNLPPRYTLPLLPQKPTEPDFTANHTTDP

>AT1G19780

MYKSQYISGHREKFVRLDDTDSRVSMSSNATGMKKRSCFGLFNVTSRGGGKTKNTSKSFREGVKIGSEGLKTIGKSFTSGVTRAVFPEDLRVSEKKIFDPQDKTLLLWNRMFVISCILAVSVDPLFFYLPIVDNSKNCIGIDSKLAVTTTTLRTIIDVFYLTRMALQFRTAYIAPSSRVFGRGELVIDPAKIAERYLTRYFIVDFLAVLPLPQIAVWKFLHGSKGTDVLPTKQALLHIVITQYIPRFVRFIPLTSELKKTAGAFAEGAWAGAAYYLLWYMLASHITGAFWYMLSVERNDTCLRSACKVQPDPKVCVQILYCGSKLMSSRETDWIKSVPDLFKNNCSAKSDESKFNYGIYSQAVSSGIVSSTTFFSKFCYCLWWGLQNLSTLGQGLQTSTYPGEVLFSIAIAVAGLLLFALLIGNMQTYLQSLTVRLEEMRIKRRDSEQWMHHRSLPQNLRERVRRYDQYKWLETRGVDEENIVQSLPKDLRRDIKRHLCLNLVRRVPLFANMDERLLDAICERLKPSLYTESTYIVREGDPVNEMLFIIRGRLESVTTDGGRSGFFNRGLLKEGDFCGEELLTWALDPKAGSNLPSSTRTVKALTEVEAFALEAEELKFVASQFRRLHSRQVQQTFRFYSQQWRTWAACFIQAAWRRHLRRKIAELRRKEEEEEEMDYEDDEYYDDNMGGMVTRSDSSVGSSSTLRSTVFASRFAANALKGHKLRVTESSKSLMNLTKPSEPDFEALDTDDLN

>AT2G46450

MNHRRSKFARIDSMGVDGKLKSVRGRLKKVYGKMKTLENWRKTVLLACVVALAIDPLFLFIPLIDSQRFCFTFDKTLVAVVCVIRTFIDTFYVIHIIYYLITETIAPRSQASLRGEIVVHSKATLKTRLLFHFIVDIISVLPIPQVVVLTLIPLSASLVSERILKWIILSQYVPRIIRMYPLYKEVTRAFGTVAESKWAGAALNLFLYMLHSYVFGAFWYLSSIERKSKCWRAACARTSDCNLTVTDLLCKRAGSDNIRFLNTSCPLIDPAQITNSTDFDFGMYIDALKSGVLEVKPKDFPRKFVYCFWWGLRNISALGQNLETSNSAGEIFFAIIICVSGLLLFAVLIGNVQKYLQSSTTRVDEMEEKRRDTEKWMSYRVIPEYLKERIRRFEDYKWRETKGTEEEALLRSLPKDLRLETKRYLYLDMLKRVPWLNIMDDGWLLEAVCDRVKSVFYLANSFIVREGHPVEEMLIVTRGKLKSTTGSHEMGVRNNCCDLQDGDICGELLFNGSRLPTSTRTVMTLTEVEGFILLPDDIKFIASHLNVFQRQKLQRTFRLYSQQWRSWAAFFIQAAWRKHCKRKLSKTRDNENIPQGTQLNLASTLYVSRFVSKALQNRRKDTADCSSSPDMSPPVPHKPADLEFAKAEA

>AT4G30360

MELRKDKLLMFYSEGKESKEAKWAVNDPMSKSYKLSLPSALRPDNLLPGNRLRYTDASKSKSSKVSWYKTILDPGSEIVLKWNWVFIVSCMVALFIDPLYFFVPAIGGDKNYPCARTDTSLSILVTFFRTIADLFYLLHIFIKFRTGFIAPNSSTRVFGRGELVMDPKAIAWRYIKSDFIIDLIATLPLPQIVIWFVISTTKSYRFDHNNNAIALIVLLQYIPRFYLIIPLSSQIVKATGVVTKTAWAGAAYNLLLYMLASHVLGAAWYILSVDRYTSCWKSRCNGEAGQVNCQLYYLDCDSMYDNNQMTWANVTKVFKLCDARNGEFKYGIFGNAITKNVVSSQFFERYFYCLWWGLQQLSSYGQNLSTTMFMGETTFAVLIAIFGLVLFAHLIGNMQTYLQSLTVRLEEWRLKKRDTEEWMRHRQLPEELRNRVRRYEQYKWLATRGVDEEVLLQSLPTDLRRDIQRHLCLDLVRRVPFFSQMDDQLLDAICERLVSSLCTEGTYLVREGDLISEMLFIIRGRLESSTTNGGRTGFFNSIILRPGDFCGEELLSWALLPKSTLNLPSSTRTVRALVEVEAFALRAEDLKFVANQFRRLHSKKLQHTFRFYSHHWRTWAACFIQAAWRRYKRRVMENNLTAIESMENEEGEVGEELVVVEEEECVEESPRTKMNLGVMVLASRFAANTRRGVAAQRVKDVELPRFKKPEEPDFSAEHDD

>AT5G14870

MNKIRSLRCLLPETITSASTAASNRGSDGSQFSVLWRHQILDPDSNIVTYWNHVFLITSILALFLDPFYFYVPYVGGPACLSIDISLAATVTFFRTVADIFHLLHIFMKFRTAFVARSSRVFGRGELVMDSREIAMRYLKTDFLIDVAAMLPLPQLVIWLVIPAATNGTANHANSTLALIVLVQYIPRSFIIFPLNQRIIKTTGFIAKTAWAGAAYNLLLYILASHVLGAMWYLSSIGRQFSCWSNVCKKDNALRVLDCLPSFLDCKSLEQPERQYWQNVTQVLSHCDATSSTTNFKFGMFAEAFTTQVATTDFVSKYLYCLWWGLRNLSSYGQNITTSVYLGETLFCITICIFGLILFTLLIGNMQSSLQSMSVRVEEWRVKRRDTEEWMRHRQLPPELQERVRRFVQYKWLATRGVDEESILHSLPTDLRREIQRHLCLSLVRRVPFFSQMDDQLLDAICGCLVSSLSTAGTYIFREGDPVNEMLFVIRGQIESSTTNGGRSGFFNSTTLRPGDFCGEELLTWALMPNSTLNLPSSTRSVRALSEVEAFALSAEDLKFVAHQFKRLQSKKLQHAFRYYSHQWRAWGACFVQSAWRRYKRRKLAKELSLHESSGYYYPDETGYNEEDEETREYYYGSDEEGGSMDNTNLGATILASKFAANTRRGTNQKASSSSTGKKDGSSTSLKMPQLFKPDEPDFSIDKEDV

>AT4G01010

MAFGRNNRVRFRDWISEGTEYGYGRNKARPSLNTVLKNVRRGLKKPLSFGSHNKKRDSNSSTTTQKNIINPQGSFLQNWNKIFLFASVIALAIDPLFFYIPIVDGERHCLNLHRNLEIAASVLRTFIDAFYIIHIVFQFRTAYISPSSRVFGRGELVDDPKAIAIKYLSSYFIIDLLSILPLPQLVVLAVIPNVNKPVSLITKDYLITVIFTQYIPRILRIYPLYTEVTRTSGIVTETAWAGAAWNLSLYMLASHVFGALWYLISVEREDRCWREACEKIPEVCNFRFLYCDGNSSVRNDFLTTSCPFINPDDITNSTVFNFGIFTDALKSGIVESDDFWKKFFYCFWWGLRNLSALGQNLNTSKFVGEIIFAVSICISGLVLFALLIGNMQKYLESTTVREEEMRVRKRDAEQWMSHRMLPDDLRKRIRRYEQYKWQETRGVEEENLLRNLPKDLRRDIKRHFCLDLLKKVPLFEIMDEQLLDAVCDKLKPVLYTENSYAIREGDPVEEMLFVMRGKLMSATTNGGRTGFFNAVYLKPSDFCGEDLLTWALDPQSSSHFPISTRTVQALTEVEAFALAADDLKLVASQFRRLHSKQLQHTFRFYSVQWRTWGASFIQAAWRRHCRRKLARSLTEEEDRFRNAITKRERNAASSSSLVATLYASRFASNALRNLRTNNLPLLPPKPSEPDFSLRNP

>AT3G17690

MAHTRTFTSRNRSVSLSNPSFSIDGFDNSTVTLGYTGPLRTQRIRPPLVQMSGPIHSTRRTEPLFSPSPQESPDSSSTVDVPPEDDFVFKNANLLRSGQLGMCNDPYCTTCPSYYNRQAAQLHTSRVSASRFRTVLYGDARGWAKRFASSVRRCLPGIMNPHSKFVQVWTRVLAFSSLVAIFIDPLFFFLLLIQQDNKCIAIDWRATKVLVSLRSITDLIFFINILLQFRLAYVAPESRIVGAGQLVDHPRKIARHYFRGKFLLDMFIVFPIPQIMILRIIPLHLGTRREESEKQILRATVLFQYIPKLYRLLPLLAGQTSTGFIFESAWANFVINLLTFMLAGHAVGSCWYLSALQRVKKCMLNAWNISADERRNLIDCARGSYASKSQRDLWRDNASVNACFQENGYTYGIYLKAVNLTNESSFFTRFSYSLYWGFQQISTLAGNLSPSYSVGEVFFTMGIIGLGLLLFARLIGNMHNFLQSLDRRRMEMMLRKRDVEQWMSHRRLPEDIRKRVREVERYTWAATRGVNEELLFENMPDDLQRDIRRHLFKFLKKVRIFSLMDESVLDSIRERLKQRTYIRSSTVLHHRGLVEKMVFIVRGEMESIGEDGSVLPLSEGDVCGEELLTWCLSSINPDGTRIKMPPKGLVSNRNVRCVTNVEAFSLSVADLEDVTSLFSRFLRSHRVQGAIRYESPYWRLRAAMQIQVAWRYRKRQLQRLNTAHSNSNR

>AT2G46430

MMNPQRNKFVRFNGNDDEFSTKTTRPSVSSVMKTVRRSFEKGSEKIRTFKRPLSVHSNKNKENNKKKKILRVMNPNDSYLQSWNKIFLLLSVVALAFDPLFFYIPYVKPERFCLNLDKKLQTIACVFRTFIDAFYVVHMLFQFHTGFITPSSSGFGRGELNEKHKDIALRYLGSYFLIDLLSILPIPQVVVLAIVPRMRRPASLVAKELLKWVIFCQYVPRIARIYPLFKEVTRTSGLVTETAWAGAALNLFLYMLASHVFGSFWYLISIERKDRCWREACAKIQNCTHAYLYCSPTGEDNRLFLNGSCPLIDPEEITNSTVFNFGIFADALQSGVVESRDFPKKFFYCFWWGLRNLSALGQNLKTSAFEGEIIFAIVICISGLVLFALLIGNMQKYLQSTTVRVEEMRVKRRDAEQWMSHRMLPDDLRKRIRKYEQYKWQETKGVEEEALLSSLPKDLRKDIKRHLCLKLLKKVPWFQAMDDRLLDALCARLKTVLYTEKSYIVREGEPVEDMLFIMRGNLISTTTYGGRTGFFNSVDLVAGDFCGDLLTWALDPLSSQFPISSRTVQALTEVEGFLLSADDLKFVATQYRRLHSKQLRHMFRFYSVQWQTWAACFIQAAWKRHCRRKLSKALREEEGKLHNTLQNDDSGGNKLNLGAAIYASRFASHALRNLRANAAARNSRFPHMLTLLPQKPADPEFPMDET

>AT2G46440

MNLQRRKFVRLDSTGVDGKLKSVRGRLKKVYGKMKTLENWRKTVLLACVVALAIDPLFLFIPLIDSQRFCFTFDKTLVAVVCVIRTFIDTFYVIHIIYYLITETIAPRSQASLRGEIVVHSKATLKTRLLFHFIVDIISVLPIPQVVVLTLIPLSASLVSERILKWIILSQYVPRIIRMYPLYKEVTRAFGTVAESKRVGAALNFFLYMLHSYVCGAFWYLSSIERKSTCWRAACARTSDCNLTVTDLLCKRAGSDNIRFLNTSCPLIDPAQITNSTDFDFGMYIDALKSGVLEVKPKDFPRKFVYCFWWGLRNISALGQNLETSNSAGEIFFAIIICVSGLLLFAVLIGNVQKYLQSSTTRVDEMEEKKRDTEKWMSYREIPEYLKERIRRFEDYKWRRTKGTEEEALLRSLPKDLRLETKRYLFLKLLKKVPLLQAMDDQLLDALCARLKTVHYTEKSYIVREGEPVEDMLFIMRGNLISTTTYGGRTGFFNSVDLIAGDSCGDLLTWALYSLSSQFPISSRTVQALTEVEGFVISADDLKFVATQYRRLHSKQLQHMFRFYSLQWQTWAACFIQAAWKRHCRRKLSKALREEEGKLHNTLQNDDSGGNKLNLGAAIYA

>LOC_Os06g33570

MMMGREDKYVRFEDWRSEQSVMSPRRHNALSSLKERTAGVFAFLGNLVHSETLKRLVLHERKLTTRTLHPQGPFLQSWNKIFVLSCIFAVSVDPLFFYIPVINDNNTCWYLDKKLEITASVLRFFTDIFYILHIIFQFRTGYIASSLTTFGRGVLVEDRYAIAKRYLSTYFLIDVFAVLPLPQVVILVVLPNLGGSEVTKAKNILMFIVICQYVPRLIRIRPLYLQITRSAGVITETPWAGAVLNLLIYLLASHVLGALWYLLSIERKDACWRDMCSNNSTVCNQAYLYCGDKENSILRTACLPIDSNDIDPNFGIYVPALNNVSQSTNFLAKLFYCVWWGLQNLSSLGQNLKTSTYAWENLFAVFVSISGLVLFALLIGNVQTYLQSAHLREEEMRVKSRDTDQWMSYRLLPENLKERIRRHEKYRWHQTSGVDEELLLMNLPKDLRRAIKRHLCLSLLMRVPMFENMDDQLLNALCDRLKPVLYTEGSCIIREEDPVNEMLFIMRGNLMSMTTNGGRTGFFNSDVLKGGDFCGEELLTWALDPTSVSSLPSSTRTVKTMSEVEAFALRAEDLKFVATQFRRLHSKQLQHTFKFYSQHWRTWAACFIQAAWHRYCRKKIEDSLREKEKRLQFAIVNDGATTLSFRAAIYASRFAGNMMRILRRNATRKARLKESVPARLLQKPAEPNFAAEEQ

>LOC_Os06g33610

MRVKSRDTDQWMSYRLLPENLKERIRRHEKYRWHQTSGVDEELLLMNLPKDLRRAIKRHLCLSLLMRVPMFENMDDPLLDALCDHLKPVLYTEGSCIIREEDPVYEMLFIMRGNLMSMTTDGGITGFFKSDVLKGGDFCGEELLTWALDPTSVSRLPSSTRTVETMSEVEAFALTAEDLKFVATQFRRLYRKQLRHTFRAPLNCSSEIVEEQENTLF

>LOC_Os03g44440

MSYASGGGGGGELATKRSAFHIDYGGGVSLRRLAQPEALARGMITQGSAQLRTLGRSLRTGAAMAVVFQEDLKNTSRKIFDPQDRLLVRLNRSFVVSCIVSIAVDPVFFYAPQVTANGGNLCVGISRDLAISASVVRTVVDLFFAARIVLQFRTAYIAPSSRVFGRGELVIDTAQIAARYFRRFFAADLLSVLPLPQIVIWKFLHRSKGAAVLSTKDALLIIVFLQYIPRVVRIYPLSSELKRTSGAFAESAYAGAAYYLLWYMLASHIVGASWYLLSIERVSDCWKKACNEFPGCNKIYMYCGNDHQKGFLEWRTITRQYINETCEPRDGVMPFNYGIYTPAVRSDVIKSNDFTSKLLYCLWWGLANLSTLGQGLQTSIYTGEALFSIFLATFGLILMAMLIGNIQTYLQSMTVRLEEMRVKRRDSEQWMHHRLLPQELRERVRRYDAYKWVNTRGVDEEVLVANLPKDLRRDIKRHLCLGLVRRVPLFANMDERLLDAICERLRPALYTERTFIIREGDPVDQMLFIIRGCLESITTDGGRSGFFNRSLLEESDFCGEELLTWALDPKAGLSLPSSTRTVRALSEVEAFALHSDELKFVAGQFRRMHSKQVQHTFRFYSQQWRTWAATYIQAAWRRHLKRRAAELRRREEEEEEAAAIRSSTGLKTTMLVSRFAANAMRGVHRQRSRRADEVLMMPMPKPSEPDFGADY

>LOC_Os12g28260

MFGSCGGGYRTQTINGRKGTFVRLEQQEDQERQPAATYTMDGSGGGGRVQHVMDSYFSSAPKIRTRSVRMAAAGVMSIGGYRAERLKSIGRVFQEDLTNMSQKIFDPQDAFLVRMNRLFVMACIVSVAVDPLFFYLPAVTATDSNTCIGFDRGLATGATAVRSAIDLFYLARIALQFRTAYIAPSSRVFGRGELVIDPAAIARRYVRRFFVVDLLSVLPLPQIPIWNFLHRPKGADLLPTKNALLFIVLVQYIPRLVRFYPITSELKRTTGVFAETAFAGAAYYLLLYMLASHMVGAFWYLLSIERLDDCWRENCRVLKFHQCKKYMYCGGGNLGQSGFLEWRTMIRQVLVMECAPADEAGTGFQYGIFTTAIQSGVVSTTNLVAKVLFCLWWGLQNLSTVGQGLKTTHYKGEALFAIFLAVFGLILMALLIGNMQTYLQSMTLRLEEMRLRRRDSEQWMRHRVLPVDLQERVWRHDQYRWLETRGVDEDSLVRSLPKDLRRDVKRHLCLRLVRRVPLFANMDERLLDAICERLKPSLCTEATYILREGDPVDEMLFIIRGRLESSTTDGGRMGFFNRGLLKEGDFCGEELLTWALDPKAAANLPLSTRTVKAISEVEAFALHADELKFVAGQFRRLHSKQLQQTFRFYSQQWRTWASCFIQAAWRRHLKRRAAEQRRREEEEEEEAASASSSCQITTTVLVSRFAKNAMRGAQRQRSRRDANLIVLPKPPEPDFQTMEY

>LOC_Os04g55080

MFDSAHKAQYIDGQREMFKRLDESSPRSSVPSEVGGRSTLKFSMPSFGYDSFNPVRSFLSGVRKGSGRLKSLRQSLTSGAPKTAFAEDLKSFKKTIFDPQEKFLFQMNWFCFLSCVFAVAVDPLFFFLPIIDGDDKSSCIGIDKKLAVTSTIIRTILDLVYLIRVFLQFRTAYVAPSSRVFGTGELVIDPMRIAIRYLKSYFVMDFFALLPLPQIVVWRYLHTLDGPDVPSTKNALVWVVLFQYIPRLLRIFPVTKDLKRTAGVFIETAWLGAAYYLLWFMLAGHNVGTLWYFLTIEREDSCWRSNCHSNDGCNKSYLYCSDNHTGNYTSWLSKRTELLSACSTNSFQFGIFEQALVSGILRPGNFISKICYCFWWGLQNLSTLGQGLQTSIYPGEVLFSIAICVIGLILFALLIGNMQTYLQSVAIRLEEMRVKKRDAEQWMHHRSLPPQIRERVRRYERYRWLETRGVDEENLVQTLPKDLRRDIKRHLCLGLVKRVPLFENMDERLLDAICERLRPTLYTENEYILREGDPVDEMHFILHGCLESETTDGGRSGFFNKVQLKEGAFCGDELLTWALDPKSAANFPASTRTVKALTEVEAFALCAEELKFVASQFRRLHSRQVQHTFRFYSQHWRTWAACFIQAAWRRYYKRKMAEQHRKEEEAANRQSSSSHHPSLAATIYASRFAANALRGVHRLRSRASPTIVRLPKPPEPDFAVDEAD

>LOC_Os02g41710

MACNGSRAVRFQNDMELPHWKTSSVPECTSSSRSTKHGKAQHQQQQHHDPRKWRRGGGGGGSLKDRVLSRAFSEELESLMSSGANHLFFDPRGQLIHLWSKIFLAACLASLFVDPLFLYLTGTRQNMCIELKYSLAFTLSMIRSLLDLFYAAHIFFRFRTAFIAPSSRVFGRGELVIQPCKIARRYLAGTFWFDLVTALPLPQFVIWIVIPKLKESATANRKNILRFSIIFQYLPRLFQIFPLSRQIVMATGVMTETAWAGAAYNLILYMLASHVLGALWYLFSVQRQEACWREACHVEGPSCQTLFFDCKTVSSNRTMWYELSNITSLCTPSNGFYQFGIYGEALDNGLTSSSFTQKYFYCFWWGLKNLSCLGQNLSTSLFIGEITFATVIGVLGLVLFALLIGNMQATMVRLEEWRTKRTDMERWMNHRQIPQPLKQCVRRYHQYKWLATRGVDEEALLEDLPMDIRRDIKRHLCLDLVRRVPLFDEMDERMLEAICERLRPALYTRGTRLVRELDPVDSMLFIIRGYLDSYTTQGGRSGFFNSCRIGAGEFCGEELLPWALDPRPAASLPLSTRTVRAVSEVEAFALVADDLRFVASQFRRLHSARIRHRFRFYSHQWRTWAACFIQAAWRRNKRRRASMELRMREGGEARPGGSVRCRRHSCDGKALIKKPMEPDFTVEEED

>LOC_Os12g06570

MDMQIGVGVTAVRTVADLFYLAHMILKFRTAFVAPSSRVFGRGELVRDPDQIAIRYLKNDFIIDLAAMLPIPQVIIWFVIPAVNNSSANHTNNTLSMIVLIQYIPRVFLIVSLNSKIVKSSGVVTRTAWAGAAYNLLLYTLASHVLGALWYLLSIERQYTCWMDVCTRENGTNPAIPKCYMSYLDCKTLEDPIRMDWHSRSEIDHQCLLPEATYVYGLFADALNLDVAKVNFWDKYLYCLWWGFRNLSSYGQNLENSTYRGETIFCILICIMGLVFFSHLIGNMQTYLQSMTVRLEEWRVKRRDIEEWMRHRQLPLELQERVRRFFQYKWLATRGVDEESILQSLPLDLRREIQRHLCLALVRRVPFFSQMDEQLLDAICERLVSSLSTKDAYIVREGDPVSEMLFVIRGELESSTTDGGRTNFFSSITLRPGDFCGEELLTWALMPNPSLNFPQSTRTVRSVTEVEAFALRAEDLKYVANQFKRLHSKRLQHAFRYYSHQWRSWGACFVQGAWRRYKKRKLARELSKQEELYYMQGQGGDDGDGHDDSDSAPLLGAGVGAGGDHRDGAAAGAAHLGATFLASKFAKNTKKSAAAHHGKARMEDVSSIKFPKLAKPDEPDFSLSSDDVL

>LOC_Os09g38580

MFGSRVQDEVEMQRRTTNRIFPDERQDQFKLPFQAARADRFGVNRIDAKTTEKIKVISEGNIPWHRRILDPGSSMVLMWNRVFLGSCLFALFIDPFFYYLPLVHVLDESTNRSCIAKDRRLSITITVLRTFADLFYMLNIMVKFHTAYVDPKSRVLGKGELVLDLKKIQRRYLRTDFFIDLLATIPLPQVTVWIIMPSIKNSDYNIRNTTFALVIMIQYIFRMYLIVPLSNQIIKAAGVVAKSAWLGAAYNLLYYMLASHITGAIYYLLSIERQITCWNQQCLNESCSFNFISCDNTGSSSYLTWGKNTSIFDNCDPNRNSSANPPPFNYGMFSTALSKGAVSAPFLEKYFFCLWWGLLQLSSSGNPLQTSAYIAENTFAIAIGALSLVLFAQLIGNMQTYLQSISKRLEEWRLRQRDMEEWMRHHQLPDELQDRVRRFVQVKWLATRGVEEESILQALPADIRRDVQRHLCLDLVRRVPFFSEMDYQLLDAICERLVSFLCPERTYISREGDPVNEMLFVIRGKLESSTTNGGRSNFFNSIILRPGDFAGEELLTWALLPKTNVHFPLSTRTVQSLTEVEAFALRAEDLKFVANQFRRLHSKKLQHTFRFYSHHWRTWAACFIQAAWRQHQRRKLAESLSRWESYSWWPEEHPPADKPKQEGTSSSTKTIAESAIAQMHKFASASRRFRADDTAIRRLQKPDEPDFSADHFD

>LOC_Os02g54760

MFGAGKVDDEMALKRQRTVRFYDEKAKPTIPTHQKQAGFAASKLGVASSGKNKIFVPGEELWYKRILDPSSDFILTWNHIFLFSCFVALFIDPLYFYVPKISYGTPNSCIGTDRHLAITVTFFRSISDLLYFTHIIIKFRTAYINPSSTMRVFGRGDLITDPKEIAWQYLRSDFVVDAVAALPLPQILIWFVIPAIKYSTDEHNNNILVLIVLAQYFPRLYLIFPLTYEIVKTTGVVAKTAWQGAAYNMLLYMIASHVLGALWYLLSVDRQTACWKSNCKNETGCDIKFLDCDVIPNQNWASKTAIFNTCDATNTSISFDYGMFQPALFNQAPGQRFLMKYFYSLWWGLQNLSCYGQTITVSTYIGETLYCIFLAVLGLVLFAHLIGNVQTYLQSITVRVEEWRLKQRDTEEWMRHRQLPHELRERVRRFIQYKWLATRGVNEESILQALPADLRRDIKRHLCLGLVRRVPFFSQMDNQLLDAICERLVSSLCTQGTYIVREGDPVTEMLFIIRGKLESSTTNGGRTGFFNSTTLKSGDFCGEELLGWALVPKPTVNLPSSTRTVKALIEVEAFALQAEDLKFVANQFRRLHSKRLQHTFRYYSHHWRTWASCFIQAAWRRYKRRKMARDLSMRESFCSMRSDDSNGEDDSPPKQNLAMKIMSGSRKGPQNMKELPKLRKPDEPDFSAEPCE

>LOC_Os06g08850

MFGSRRVKDEMELRKQRTVRFHEERAKPTIPTHQKQAGLATSKLGLGISEKNKIFLAGNELWYKKIIDPSSDFILTWNYVLRIACFVALFMDPLYFYVPKIYYGTPNSCIGRDTRLAIIVTVFRSITDLFYVLQIIIKFRTAYINPSSTLGVFSRGDLVTDPGNIAKHYLRSSFVVDLVASLPLPQIIIWSVIPSVKYSLSEHDDDILLLIALFQYVLRLYLVFSLNSKIVEVTGAFSKTAWQGAAYNLLLYMIASHVLGALWYLLSVDRQTACWEKYCSKEAGCQNRYLACDIQSDSNWKISTAIFNKCDATNKTIDFDFGMFTPLLSNQAPDQGFLKKFFYCLWWGLQNLSCYGQTLTVSTYIGETLYAIFLAVLGLVLFAHLIGNVQTYLQSITARVEEWRIKQRDTEEWMRHRQLPQKLRERVRRFVHYKWLATRGVDEESILKALPADLRRDIKRHLCLDLVCRVPFFSQMDGQLLDAICERLVSSLSTVGTYIVREGDPVTEMLFIIRGKLESSTTDGGRTGFFNSITLKTGDFCGEELLGWALVPKPTVNLPSSTRTVKTIVEVEAFALRAEDLKFVASQFRRLHSRKLQHTFRYYSHHWRTWAACFIQAAWRRYKRRRLAKDLSIRESFFSRRSFEDDGSPEHSLVLNAVRKGAHIIKELPKFRKPSEPDFSAEHDD

>LOC_Os02g53340

MSDQERDDIPMLLRNVELPTFPPRSTSMCIPVRDDEYEEDTFVPHTGPLFVQPPTQTAAAGIPFTNTPDMPPRPPQGKQVNKPHAIMPEEIGGNRWSYSGNVPKNEHLMMSGPLGQCDDPDCVNCPPACKNKRHFHRGSSTLDSKFHNFLCEHGGGWKKEIERFLSRIPVMNPHAKVVQQWNQFFVISCLVAIFIDPLFFFLLSVQKDNKCIVLNWHFATALAVVRSVTDAIYFLHMLLQFRLAYVAPESRVVGAGDLVDEPKKIAVRYLRGYFLLDFFVVLPLPQVMILLVIPKYVGLSTANYAKNLLRITVLLQYVPRIIRFVPLLGGQSDSSANGFIFESAWANFVINLLMFVLAGHVVGSCWYLFGLQRVNQCLRNACSASKIPSCDGFIDCGRGINIGKQNQLSRQQWFNDSASTACFDTGDNGFHYGIYEQAVLLTTEDNAVKRYIYSLFWGFQQISTLAGNLVPSYFAWEVLFTMAIIGLGLLLFALLIGNMQNFLQALGRRRLEMQLRRRDVEQWMSHRRLPEDLRRRVRRAERFTWAATQGVNEEELLSNLPEDIQRDIRRHFFRFLNKVRLFTLMDWPILDAICDKLRQNLYISGSDILYQGGPVEKMVFIVRGKLESISADGSKAPLHEGDVCGEELLTWYLEHSSANRDGGRMRFHGMRLVAIRTVRCLTNVEAFVLRASDLEEVTSQFSRFLRNPRVQGAIRYESPYWRTIAATRIQVAWRYRNRRLKRAGMSKLNDQSYNSALERGARECDARQHGRV

>LOC_Os06g10580

MSGQERDDVPMLELQRFPTRSVSMCIPVRDDIYEDSIISHSGPIFTPAPTQYTSVAIPSGNRDMLDKLPRPKVKSKPHVVTPEEVGISNWPYDQHVPKNKHLMMYSEPLGLCDNPDCVDCPRACKNKRHFQRSLAPFDNKFHNILYGYGDRWKKKAGHYLSYIPIMKPHDKAVHRWNQFFVISCLLAIFNDPLFFFLLSVDKDYKCIVFNWNFAIALAVGRSVTDAIYFLHMLLQFRLAYVAPESRVVGTGDLVDEPMKIAMRYLRGFFVLDLFVVLPLPQVMILLVIPKYVGLSSANYAKNLLRATVLLQYVPRIIRFVPLLGGQSTNGFIFESAWSTFVINLLMFVLAGHVVGSCWYLFGLQRVNQCLRDSCAASNISKALCNNCTDCGITGINRTNWLNNSDLTGCFDTKSGNFPYGIYQQAVLLTTEPGLKRYIYSLFWGFQQISTLAGNLIPSYFVWEVIFTMAIIGLGLLLFALLIGSMQNFLQALGKRRLEMQLRRRDVEQWMSHRRLPEDLRRRVRSAERFSWVATRGVNEEELLSNLPEDIQRGIRRHFFGFLKKVRLFNLMDNATWDAICDKLRQNLYITGSDILYQGGPVEKMVFIVRGRLESISADGNKSPLQEGDVCGEELLSWYLEQSSVNRDGGKIKLHGMRLVAIRTVRCLTNVEAFVLRARDLEEVTSQFSRFLRNPLVLGTIRYESPYWKNLAANRIQVAWRYRKRRLKRAEMQRLQ

>LOC_Os03g55100

MPSLSFLRFLSGRSLADVCDGVKRRLGLGDDEGRDEEAGLAGGSSRPAAAAAVAGPPGECYACTQPGVPSFHSTTCDQVHSPDWDADAGSSLVPVQAQPSAAHHAAAAAARWVFGPVLDPRSKRVQRWNRWILLARAAALAVDPLFFYALSIGRAGQPCVYMDAGLAAAVTALRTAADLAHLAHVLLQFRVAYVSRESLVVGCGKLVWDPRAIAAHYARSLKGLWFDLFVILPIPQVIFWLVIPKLIREEQIKLIMTMLLLLFLLQFLPKVYHSIYIMRKMQKVTGYIFGTIWWGFGLNLFAYFIASHIAGGCWYVLAIQRVASCLQEECKIKNTCNLTSLACSKEMCFHLPWSDKNGLACNLTSFGQQNIPDCLSGNGPFAYGIYKGALPVISSNSLAVKILYPIFWGLMTLSTFGNDLEPTSNWLEVIFSIINVLSGLMLFTLLIGNIQVFLHAVLARKRKMQLRFRDMEWWMRRRQLPSRLRQRVRKYERERWAAITGDEEMEMIKDLPEGLRRDIKRYLCLELVKQVPLFHGMDDLILDNICDRLRPLVFSSGEKVIREGDPVQRMVFVLQGKLRSTQPLAKGVVATCMLGAGNFLGDELLSWCLRRPSLDRLPASSATFECVETAQAFCLDAPDLRFITEQFRYKFANEKLKRTARYYSSNWRTWAAVNIQLAWRRYKARTTTDLASAAQPPSAGGPDDGDRRLRHYAAMFMSLRPHDHLE

>LOC_Os01g57370

MASSSAAAASSAHGVGVVQRLWLEEQERKPPPKRGGGKRRWAWAPLEPRRAGWWAREWDRAYLLACAAGLMVDPLFLYAVSVSGPLMCVFLDGWFAAAVTVLRCTVDAMHAWNLLMRLRAAVRPPEEDDGADEEVAAERGAGGNGGGPAPAQVARPVSRKGLMLDMFVILPVMQVIVWVAAPAMIRAGSTTAVMTVLLVSFLFEYLPKIYHAVRLLRRMQNTYVFGTIWWGIALNLMAYFVAAHAVGACWYLLGAQRATKCLKEQCAQGGSGCAPGALACAAPLYYGGAVGGVGADRLAWALDASARGTCLDSGDNYQYGAYKWTVMLVANPSRLEKILLPIFWGLMTLSTFGNLASTTEWLEIVFNIITITGGLILVTMLIGNIKVFLNAATSKKQAMQTRLRGVEWWMKRKKLPQSFRHRVRQHERQRWAATRGVDECRIVRDLPEGLRRDIKYHLCLDLVRQVPLFQHMDDLVLENICDRVKSLVFPKGEIIVREGDPVQRMLFIVRGHLQSSQVLRTGATSCCTLGPGNFSGDELLSWCMRRPFLERLPASSSTLVTMESTEAFGLEAADVKYVTQHFRYTFTNDRVRRSARYYSHGWRTWAAVAVQLAWRRYKHRKTLASLSFIRPRRPLSRCSSLGEEKLRLYTAILTSPKPNPNQDDLV

>LOC_Os05g42250

MSGELSTRASTSSSSSSPPGDARGPEHGGTPRGEVSSKRRLVLRRRQRWRRLGGGAAASWAAADPRARWVREWNRAYLLACAAGLMVDPLFLYAVSLSGPLMCVFLDGWLAAAVTALRCMVDAMHAWNIVTQLRVSRAGRERACAAGPDEEQPEAEAAAPAPAADADAAASNKLRDHGRYRKWLVLDFFVILPVMQVVVWVAAPAMIRAGSTTAVMTVMLVAFMLEYLPKIYHSVVFLRRMQNQSGHIFGTIWWGIALNLIAYFVAAHAVGACWYLLGVQRATKCLKEQCLLAGLPACASSTAAVACVDPLYYGAAVASVGGDRLAWGGNATARNVCLSSGDNYQYGAYKWTVMLVSNPSRLEKMLLPIFWGLMTLSTFGNLESTTEWVEIVFNIMTITGGLILVTMLIGNIKVFLNATTSKKQAMQTRLRGLEWWMEHKGVPHGFRQRVRQFERQRWAATRGVDECQIVRDLPEGLRRDIKYHLCLDLVRQVPLFHHMDDLVLENICDRVKSLIFPKGEIIVREGDPVQRMLFIVRGHLQCSQVMRNGATSWCTLGPGNFSGDELLSWCMRRPFMERLPASSSTLVTAESTEAFGLEAGDVKYVTQHFRYTFTSDKVRRSARYYSHGWRTWAAVAVQLAWRRYKHRKTLASLSFIRPRRPLSRCSSLGEEKLRLYTAILTSPKPNQDDDF
